# The design, delivery and evaluation of ‘Human Perspectives VR’: An immersive educational programme designed to raise awareness of contributory factors for a traumatic childbirth experience and PTSD

**DOI:** 10.1371/journal.pone.0276263

**Published:** 2022-11-02

**Authors:** Stephanie Heys, Soo Downe, Mick McKeown, Gill Thomson

**Affiliations:** 1 Maternity Learning and Development Lead, Consultant Midwife, The Northwest Ambulance Service, The University of Central Lancashire, Preston, United Kingdom; 2 Professor of Midwifery Studies, The University of Central Lancashire, Preston, United Kingdom; 3 Professor of Democratic Mental Health, The University of Central Lancashire, Preston, United Kingdom; 4 Professor of Perinatal Health, The University of Central Lancashire, Preston, United Kingdom; Lausanne University Hospital: Centre Hospitalier Universitaire Vaudois (CH), SWITZERLAND

## Abstract

**Background:**

A traumatic childbirth experience affects ~30% of women each year, with negative impacts on maternal, infant, and family wellbeing. Women classified as vulnerable or marginalised are those more likely to experience a psychologically traumatising birth. A key contributory factor for a traumatic childbirth experience is women’s relationships with maternity care providers.

**Aims:**

To develop, design and evaluate an immersive educational programme for maternity care providers to raise awareness of traumatic childbirth experiences amongst vulnerable groups, and ultimately to improve women’s experiences of childbirth.

**Methods:**

A critical pedagogical approach that utilised virtual reality (VR) underpinned the design and development of the educational programme. This involved: a) collecting vulnerable/disadvantaged women’s experiences of birth via interviews; b) analysing data collected to identify key hotspots for traumatic experiences within interpersonal patient–provider relationships to develop a script; c) filming the script with professional actors creating a first person perspective via VR technology; d) using existing literature to inform the theoretical and reflective aspects of the programme; e) conducting an evaluation of the education programme using pre-and post-evaluation questionnaires and a follow-up focus group.

**Findings:**

Human Perspective VR was very well received. Participants considered the content to have enhanced their reflective practice and increased their knowledge base regarding contributory factors associated with a traumatic childbirth experience. A need for further work to implement learning into practice was highlighted.

**Conclusion:**

While further research is needed to evaluate the impact of the programme, Human Perspective VR programme offers an innovative approach to reflective education and to enhance participants’ care practices.

## Introduction

Each year it is estimated that ~30% of women experience a psychologically traumatising birth [1, 2]. A traumatic childbirth experience is defined as:

‘A traumatic childbirth experience refers to a woman’s experience of interactions and/or events directly related to childbirth that caused overwhelming distressing emotions and reactions; leading to short and/ or long-term negative impacts on a woman’s health and wellbeing’ [3].

Traumatic childbirth experiences have been associated with a wide range of negative impacts for women and their families, such as, low self-esteem, early breastfeeding cessation, and relationship issues [4]. Similar to other forms of trauma, a traumatic birth can lead to post-traumatic stress disorder (PTSD), a trauma- and stressor-related disorder, with 4.7% of women developing PTSD post birth [5].

A meta-ethnography focused on women’s experiences of a traumatic birth highlighted disrespectful care practices and women experiencing a loss of control as the main contributory factors to a distressing birth experience [6, 7]. More recently, a comparative systematic review and meta-analysis explored the prevalence and risk factors of birth-related posttraumatic stress among parents, highlighting elevated rates of PTSD in targeted samples (those with a potential risk status) such as mothers of young age, those with pregnancy complications and women with a history of childhood trauma [6]. Other research has also identified the contribution of social aspects of birth to a self-perceived traumatic birth such as a lack of understanding from health care professionals regarding the individual needs of women, feelings of stigma, lack of trust in staff providing care and communication barriers [7–11]. Women’s experiences of poor maternity care are reported globally, including care in the UK [12–15]. An international knowledge mapping exercise focused on training provision for women following a traumatic birth, acknowledge a traumatic birth experience as a key public health concern, calling for formalised care providing and training for care providers [16].

A traumatic birth, if left untreated, can lead to post-traumatic stress disorder, a recognised complex and serious mental health condition that affects approximately 4.7% of women in general community samples [5] and 19% in high-risk groups, such as women with a previous mental health illness, previous PTSD, preterm birth [17]. These statistics indicate that women from more complex vulnerable and disadvantages communities may be disproportionally affected.

### Disadvantaged and vulnerable women’s experiences of maternity care

Disadvantaged and vulnerable women have been found to be more likely to have poor access to healthcare due to issues such as mistrust of professionals [18–23], social stressors such as lack of support and complex life factors [24], communication barriers [25], health literacy [26] and fear of stigma and judgments [27, 28]. Black, Asian and minority ethnic (BAME) women and those from disadvantaged and vulnerable backgrounds have a higher risk of preterm, low birth weight babies [29, 30], are at a greater risk of poor mental health such as depression, anxiety and stress and are more likely to die during childbirth [31–34]. A recent meta-ethnographic synthesis of disadvantaged and vulnerable women’s negative experiences of maternity care in high-income countries also highlighted how disadvantaged women’s vulnerability was compounded by complex life factors, judgmental and stigmatizing attitudes by health professionals, and differential care provision [28]. Such findings highlight the need for increased awareness of contributory factors to such experiences amongst women accessing maternity care.

Additionally, women from vulnerable backgrounds and those with complex life factors are at an increased risk of perinatal mental health difficulties [35–38] and higher rates of morbidity and mortality, in particular amongst black and ethnic minority women [32]. In recent years there has been significant investment in perinatal mental health following the work of the Maternal Mental Health Alliance who highlighted the disparity in specialist provision across the UK, and commissioned an economic evaluation that highlighted the costs of poor perinatal mental health at 8.1 billion per annum [39]. However, a lack of knowledge and skills amongst midwives regarding traumatic birth and PTSD still exist [40, 41]. Furthermore, as a multi-cultural society, with high levels of deprivation and inequalities, maternity professionals are increasingly required to provide services to women from diverse backgrounds. Notwithstanding this, provision in maternity education is lacking resources and knowledge to support maternity professionals to deliver an equitable service [42]. Global and national initiatives call for the identification of enhanced approaches to address issues of disrespectful, inequitable and biased maternity care [43–46], acknowledging opportunities to harness digital solutions and technologies to support this [47].

### Innovative digital tools and approaches in healthcare

A recent quality improvement innovation in health care is the use of Virtual Reality (VR) to enhance educational approaches. The following definition is proposed, providing a detailed description of VR;

‘Virtual reality incorporates computer-generated, interactive and highly vivid environments that enable the user to achieve a state of immersion through the ultimate experience of telepresence, and facilitate engagements in human encounters that are multi-sensorial, dynamic and resemble the user’s perception and understanding of the real world’ [48].

VR has been reported as useful in redirecting patients’ attention during painful treatments and in exposure therapy for the treatment of phobias and PTSD, by creating safe imaginative spaces to encounter and overcome fears and phobias [49–53]. It has been used to address eating disorders and obesity, encouraging individuals to improve body image perceptions and adopt healthier eating habits [48, 54]. Its use also spans multiple health disciplines including patient motor rehabilitation, aiding patients to reacquire specific skills and improve body movement in virtual environments [55]. Several authors have reported an increase in knowledge retention when using VR tech as opposed to conventional teaching methods [56–58]. A recent scoping review focused on the use of VR as an application to assist pregnant women identified how this technology has different applications in pregnancy, from reducing anxiety and pain to exercise training [59]. VR has also been used to provide midwifery students with a virtual, internal anatomical view of pregnancy, its physiological progression and fetal and placental positions [60]. More recently a cross-sectional survey and observational study included an evaluation of VR within a midwifery curriculum with findings suggesting VR enhances engagement, creates authentic active learning experiences and supports students to visualise and better understand abstract concepts [61].

Drawing on the utilisation of VR as an embodiment experience to enhance sensory experience offers a unique approach to midwifery training and education, allowing professionals to experience care from the perspective of the woman. This study capitalised on this approach to develop, design and evaluate an immersive educational programme utilising VR to raise awareness of traumatic birth experiences amongst vulnerable and disadvantaged population groups for maternity care providers.

## Methods

A critical pedagogical (CP) framework underpinned the design, development, and evaluation of the immersive education programme. CP allow participants to recognize connections between individual problems and experiences and the social contexts in which they are embedded [62]. Realizing one’s consciousness ‘(‘*conscientization*‘) is the first step in achieving ‘*praxis*’, defined as the ability and knowledge to take action against oppression through liberating education [63]. Within the context of this educational programme, CP was used to facilitate midwives ‘*conscientization*‘ via illuminating situations of power within the social space of birth. The social space relates to the international interactions between women and healthcare providers. As detailed within a secondary discourse analysis focused on experiences of women who have had a traumatic birth [64], interpersonal interactions play a key role in how women experience childbirth. In our study, it was considered that helping midwives to identify how these interpersonal situations can contribute towards a traumatic birth would facilitate *‘praxis’*.

A three-phase interlinking model based on the writings of Freire helped to frame the CP approach undertaken; ‘listening and naming’, ‘dialogue and reflection’ and ‘promoting of transformative social action [62]. Full details of work undertaken in each of the three phases to design, develop and deliver the educational programme is illustrated in Table 1 and described as follows:

a) *‘Listening and naming’* relates to learning the problems, issues and real-world experience of the learners. In the case of this study—the contributory factors to traumatic birth within the social space of birth. This work involved three stages:
  i) Identifying triggers: Similar to work undertaken by Wallerstein & Bernstien [65], the educational programme was designed to present real life situations of traumatic birth from within women’s accounts. This stage involved a systematic review and meta-synthesis of available literature exploring the birth experiences of disadvantaged and vulnerable women [28] and a further 10 interviews undertaken in North-West UK (June-August 2017) to ensure the insights from the review were still relevant. This information was synthesised using a qualitative content analysis approach to identify the *‘meaning units’*, defined as *‘the constellation of words or statements that relate to the same central meaning’* [66]. The focus here was to identify ‘triggers’ that created situations of traumatic birth within the social context of childbirth (see Table 1 for overview of key triggers identified).

**Table 1:** Key triggers identified following a synthesis of primary and secondary sources.

Triggers for a traumatic birth within the social context of childbirth	Description
A lack of emotional support	A lack of emotional support refers to how women felt they were not supported emotionally by healthcare professionals.
Poor information giving	Poor information giving highlights how women felt they were not provided with sufficient information and were unsure of what was happening and why.
Poor use of language	Poor use of language related to inappropriate language used.
Unconsented interventions	Unconsented interventions were explicitly and implicitly reported by the women highlighting issues around consent and choice during birth.
Submissive interactions	Submissive interactions related to women being shouted at or not feeling that they were able to voice their concerns.
Judgemental attitudes	Judgemental attitudes related to professionals’ negative preconceptions based on women’s social, cultural and ethnic backgrounds.

This evidence, as well as wider research into traumatic birth and PTSD onset [1, 2, 17, 67, 68] was used to develop a theoretical PowerPoint presentation on traumatic birth and particular issues faced by vulnerable/disadvantaged women to be used within the educational programme.

ii) Script development: This stage included developing a script to depict one woman’s birth experience that included all identified triggers. A script writer (and screenplay director) was employed to support the process with all included authors providing input and feedback. Clinician input on drafts of the script was also provided with various stakeholder engagement and feedback.
iii) Filming the scenario: This aspect included hiring three professional actors (who acted the roles of a woman in labour, a midwife and a doctor) who were filmed using 360-degree camera technology. The screenplay director led this work with actors performing the birth scenario within one NHS maternity Trust in North-West UK. Elsasser & Hagener (2015) highlight the importance of creating a narrative field that tells a story with the props, content and set alongside the narrative and actors [69]. Filming within an NHS maternity hospital provided scenarios with a familiar environment and culture to facilitate critical reflection [65, 70]. The completed film was 7 minutes in length (see S1 File for script narrative).

Participants used VR headsets to enable 360 viewing of the filmed scenario with audio. The VR viewing provided a first-person experience of care, allowing for spatial and sensory reflective experience.

**Fig 1:**
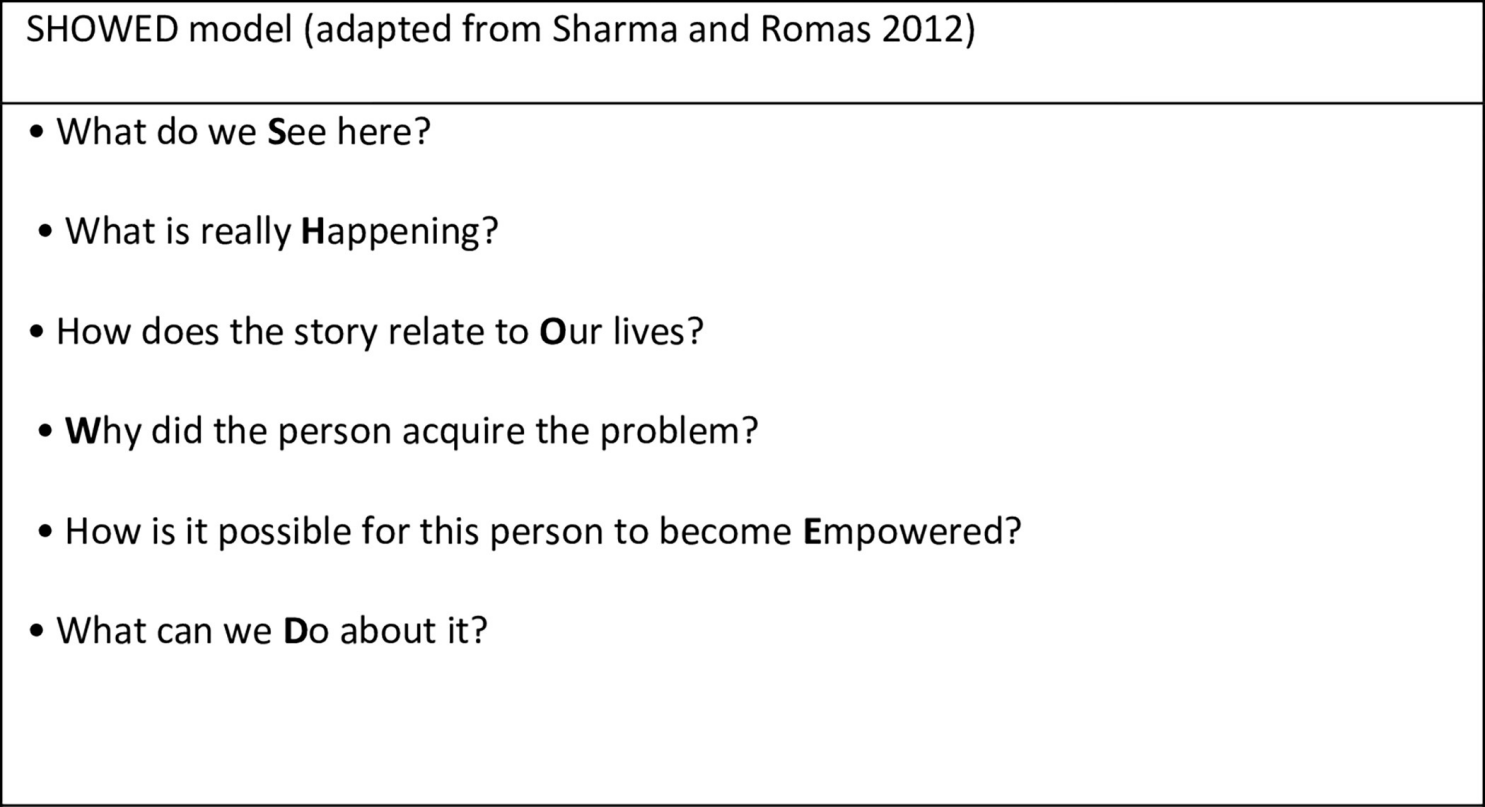
SHOWED model.

b) ***‘Dialogue and reflection’*** allow for a problem-posing approach, encouraging critical thinking amongst participants. This stage involves learners being encouraged to think critically, enabling them to focus on learning as opposed to outcomes. We drew on the SHOWED model for this phase (see Fig 1). This model was adapted from Freirean techniques [71] and provided a practical guide for the inclusion of critical questioning in sessions with learners after they had viewed the VR content.

Through dialogue in education, people can name, interrogate, and re-imagine their reality [72]. Dialogue in this section encouraged a deeper look at the social space of birth and factors that impact upon women’s experience and the potential for a traumatic birth. This included discussing care practices from different viewpoints to facilitate critical consciousness.

The SHOWED approach was used at two points. First, after the learners had watched the filmed scenario as part of a critical discussion. Second, after additional information was provided about the woman (named as Emma in the script).

*‘Emma is new to the area, she is originally from London fleeing from an abusive relationship. She is 17 years old and had a history of sexual abuse and drug use. She spent some time prior to arriving in the area in a women’s refuge*, *this is her first baby’*

This process relates to ‘culture circles’ [73] in terms of how revisiting a familiar environment with a critical eye allows participants to open up dialogue on dehumanising aspects of a scenario. Participants were given this additional information and encouraged to discuss interactions within the scenario that may have further impacted upon Emma’s experience, as well as a wider reflection on practices that may cause situations of trauma for women who may have complex life situations.

c) ***‘Promoting of transformative social action’***: The final phase in the Freirean model is the promotion of transformative social action, or the critical action phase [71]. This process is where those taking part in the programme take part in the continuous process of action and reflection to facilitate praxis [74]. Critical action has been identified as having three different forms: campaigning, awareness raising and education [75]. Campaigning or convincing an organisation or government to change policy could be at one end of the spectrum of possible change [71]. On the other end, this can relate to equipping learners with the tools they need to identify inequality and injustice so that they can seek change if that is what they desire [75]. It is this ‘education phase’ that was targeted in the programme–to promote transformative action by influencing how people think and react [70].

Wallerstein and Bernstein (1988) describe this step of a CP as a group effort to identify problems, through the process of critically assessing the social and historical roots of the problem to be addressed and then developing strategies to improve current practices [65]. Within the education programme it encouraged participants to reflect on practice from the woman’s midwife’s, and doctor’s perspective within the shared video, and to write ‘*practice points’*. The aim was to consolidate learning into a shareable format for knowledge translation and to facilitate conscientization. As a final activity in the programme, participants were asked to work as a group to identify and name five key points for practice that could have a personal impact upon a woman’s childbirth experience, and to document these on a pre-prepared template poster to be displayed in their clinical areas. This was described to participants as actions that could be shared with colleagues (i.e. once the poster is displayed it can be a focus for continued discussions/conversations and prompt actions of others) and individually implemented when caring for women during labour and birth.

### Evaluation elements

Evaluation was undertaken in two stages. First, bespoke pre- and post- training questionnaires were developed that included Likert scales and open-ended questions. The initial questionnaire gathered a baseline of participant’s knowledge of traumatic birth and PTSD, knowledge of risk factors associated with traumatic birth and PTSD. These issues were then revisited within the post-training questionnaire to provide an assessment of how the programme had influenced knowledge and attitudes. Attendees were also asked additional questions at this juncture to explore their experiences of the training. They were asked to indicate how useful the training programme was in terms of raising awareness of traumatic birth/PTSD following childbirth and how to improve women’s birth experiences measured on a scale of 1 –very useful to 5- not useful at all. An open question of *‘Do you have any thoughts on the use of VR*?’ was also included (see S2 File for questionnaires).

Field notes of discussions were undertaken on the day by GT to capture critical reflections. Participants were also re-contacted ~6 weeks after the educational programme had been delivered to the impact of attendance upon participants knowledge and practice, alongside exploring how participants found sharing learning *(practice points)* in their work areas.

### Running the event

The immersive educational programme was delivered in an NHS Trust in the North West of England on the 10^th^ of April 2018. The session was booked six weeks in advance through liaising with the practice education midwife and in close collaboration with the delivery suite coordinator. Fostering relationships with clinicians was important to ensure that delivery did not adversely impact upon clinical practice, as some participants attended during work hours. The programme was organised in the education centre at the host Trust, booked for 12 mid-day with food and refreshments provided to the participants. All participants were provided with an information sheet about the programme and associated evaluation. A reminder email was sent to each participant the week before, providing details of the location and time. At the start of the day, participants were asked to introduce themselves, were provided with an overview of the planned activities and then asked to sign a consent form (see Table 2 for overview of all activities).

**Table 2:** Overview of delivery of educational programme mapped to Matthews (2014) three step model.

Segments of the educational programme	Time spent on activity	Description of the activity	Links to three step model of a critical pedagogy (Table 14)
Introductions	5 MINS	Introductions, an overview of the day to ensure participants were clear about the programme and participants provided with an opportunity to ask any questions.	
Consent	5 MINS	Consent obtained.	
Pre-questionnaire	5 MINS	Questionnaires issued and completed	
VR scenario viewing	15 MINS	Participants were supported in the use of the VR google cardboards and how to view the scenario. The VR scenario lasted 7 minutes.	**Listening & Naming**
Discussion	15 MINS	Participants encouraged to reflect on the scenario from the woman, midwife and doctor’s perspective using the SHOWED model.	**Dialogue & Reflection**
Theoretical presentation on traumatic birth and PTSD	15 MINS	Delivery of a theoretical presentation to address issues associated with traumatic birth and PTSD onset, including information on contributory factors that can place some women at a higher risk.	**Listening & Naming**
Return to Emma’s story adding additional factors	15 MINS	Additional context on Emma’s situation provided, and participants encouraged to discuss how observed interactions may have impacted on Emma’s experience.	**Dialogue & Reflection**
Practice points group work	10 MINS	Five practice point identified during a group discussion.	**The promoting of transformative social action**
Post questionnaire	5 MINS	Questionnaires issued and completed.	
Closing and any final questions	5 MINS	Participants given opportunity to ask any final questions.	
Follow up assessment	Text responses via email	Participants re-contacted for follow up feedback	

### Ethics approval and consent to participate

Ethics approval was gained from Health Research Authority (Integrated Research Application System number (blinded for review) and (blinded for review)). Written consent was gained from participants and all methods were carried out in accordance with the Health Research Authority guidance for researchers and ethics committees and the Declaration of Helsinki.

### Data analysis

Quantitative data captured within the Likert scales were analysed descriptively using SPSS. A thematic approach was undertaken to analyse the open text answers, field notes, and email follow-up comments. Braun and Clarke’s (2006) approach of reading and re-reading, organising the data into codes and then into themes that represented the whole data set was first undertaken by SH and GT with final interpretations shared with all authors [76].

## Results

In total 10 participants attended the immersive educational training programme. Participants included two student midwives (n = 2) (who were also qualified nurses). The remaining participants’ clinical backgrounds included roles working on the local midwife-led birth centres (n = 3), joint research and clinical roles (n = 2) and centralised hospital labour ward (n = 3).

Based on the baseline questionnaire, all participants stated that they had received no training in relation to traumatic birth / PTSD throughout the course of their midwifery career. Answers to the same questions asked at both stages to assess influence of the programme on knowledge and attitudes, overall demonstrated a positive shift.

These data identified that while only three (30%) participants agreed they had ‘good’ understanding of how traumatic birth/PTSD onset following childbirth was caused at baseline, all participants either agreed or strongly agreed with this question after delivery of the programme. Similar baseline scores were recorded for the question ‘do you feel able to recognise women at risk of traumatic birth/PTSD onset. In terms of being able to impact on women’s experiences, baseline and endline measures were all positive.

In the following section, we report on five key themes to emerge from the field notes, qualitative comments to open-text questions in the questionnaires (n = 10) and email contacts (n = 10) collected ~6 week after delivery of the educational programme. Key themes identified were *‘Identifying and meeting knowledge gaps’*, *‘Becoming the other’*, *‘Nurturing professional empathy and understanding’* and ‘*Understanding and adopting a critical social lens’*. These themes are presented below.

### Identifying and meeting knowledge gaps

As reflected within the baseline questionnaire data, midwives spoke of the lack of training about traumatic birth/PTSD:

‘I’d never have thought it would have been that prevalent, because you don’t really hear about PTSD as a midwife, its more postnatal depression, I’ve never had any training on PTSD so I would not have known what to look out for’ (P1)

N = 10 participants demonstrated knowledge gaps in identifying factors that contribute to traumatic birth experiences for women, and a lack of confidence in identifying PTSD post-birth. For example, one believed traumatic births to be only attributed to physical trauma rather than it being a complex phenomenon that can include psychological trauma:

‘Birth trauma is when the woman experiences trauma such as an instrumental birth or a grade one section, it can also be when a woman had a tear or a shoulder dystocia and may need a debrief about her experience’ (P1)

Participants also reflected on situations contributing to trauma emphasising emergency situations, or an intervention-based birth, rather than it being a subjective experience that can occur irrespective of how a woman gives birth. For example, one midwife reported:

‘Birth trauma can happen when there is an emergency and a woman is scared for her life, for example a grade 1 section or a forceps delivery’ (P8)

Positive shifts however were noted in these knowledge gaps within the post-evaluation questionnaire with participants listing a range of relevant factors such as previously disclosed/undisclosed trauma, poor interpersonal interactions and poor information giving. All bar one participant felt the training programme had been ‘very useful’ (remaining participant recording ‘useful’) in terms of raising awareness of traumatic birth/PTSD following childbirth and how to improve women’s birth experiences. All respondents in follow-up email correspondence stated that attending the training has enhanced their understanding of a traumatic birth and PTSD:

‘I feel I am more knowledgeable about risk factors for birth trauma and PTSD following the training’ (P2)

All participants highlighted a need for training of this nature in the follow up emails.

### Becoming the other

Insights from participants reflected on the experience of ‘becoming the other’ in the VR scenario and how powerful this was for learning and reflection. One of the midwives reflected:

‘Wow that was powerful, you really become the other, I felt like I was in the room’ (P3)

Midwives indicated that the feelings and thoughts the film evoked enabled them to appreciate how *‘helpless’* women must feel:

‘It was interesting to be looking up all the time because I was on the bed, that must be really uncomfortable and intimidating, you just forget that dynamic of the situation in practice’ (P6)

And the lack of communication between Emma and the midwife/doctor:

‘The silences in the room were unnerving for me being the woman, I wanted her [the midwife] to ask me if I had understood what the doctor had said I thought why is she not talking to me’ (P10)

Another highlighted the sense of reality VR methodology invoked, in which she was searching for support around her within the 360 environment:

‘The worst bit for me was when everyone left the room, I was looking around to see if I had a birthing partner’ (P5)

Overall, the VR stimulated operational and emotional critical reflections about how women in labour must feel. This included how social interactions had the potential for women to feel alone and excluded from care decisions. The participants highlighted (in follow-up email contacts) the difficulty in explaining to others the psychosocial impacts this had upon them. This suggested how the immersive nature of the education programme was a crucial facet:

‘It was hard to explain the programme to someone else other than it was really useful and powerful. I also don’t think you can get across to people the emotion attached to the session or viewing the scenario, they need to attend themselves, I think that would be better’ (P5)

### Nurturing professional empathy and understanding

Participants referred to their own practice in identifying with the midwife in the film but also highlighted potential issues, e.g. *‘we can be very paternal*, *but that’s not empowering’* (P5) and ‘*two* [midwife/doctor] *people against one*, *that this is what we are going to do to you’* (P2). A risk-based approach to care was highlighted during the discussions with one participant empathising with the midwife in the scenario:

‘I don’t think she was being purposefully uncaring she was just very task orientated as she had a lot to do and probably wanted to make sure everything was safe before they went to theatre, she did ask if she had anyone to go with her, so she was thinking about the woman needing support’ (P6)

This comment generated a critical debate around the use of language and exploring effective communication and information giving. For instance, one participant reported:

‘Yes, but then she never followed it up when the women said no, she could have said don’t worry I will be with you in theatre and I will support you’ (P7)

Reflections were made on the impact of prescriptive care on a woman’s ability to advocate for herself, thereby highlighting issues surrounding choice and consent:

‘It was all ‘we are going to do this’, there was not much chance for the woman to disagree or ask questions and although they were doing what needed to be done I think they could have put it to the woman a bit better’ (P8)

Participants critically discussed the practices they saw within the scenario. They framed these responses from the woman’s perspective, but also considered real-life difficulties of keeping the woman at the centre in everyday practice. This encouraged participants to consider how the situation may have impacted upon the ability of the midwife in the scenario to provide good care. One stated:

‘It’s horrible to see but we have all been there, I feel that the midwife could have done with some support herself to better support the woman’ (P1)

With another midwife responding:

‘It’s easy to forget that there may be a midwife in another room who needs supporting and feeling like that when your busy with your own work, makes you think twice’ (P6)

These comments highlight how the programme appeared to nurture professional empathy and to view care from multiple perspectives; *‘It really saddens me to read those* [women’s quotes shared during delivery of the presentation] *I’d hate to think that I’ve ever made a woman feel that way’* (P7).

### Understanding and adopting a critical social lens

During the discussions, participants discussed frustrations of working in a system that did not facilitate individualised care, leaving them feeling overworked and under resourced. One midwife expressed her frustrations at feeling under scrutiny in situations such as the one presented in the scenario:

‘I mean she’s trying her best, she’s in task mode, she needs to get everything sorted before she goes to theatre or they will probably shout at her for not having the right paperwork, you know what it’s like ‘(P8)

This was an interesting take in which the participant empathised with the midwife in the scenario as she identified the pressures and oppressive environment in which she worked. When the discussion moved to consideration of how complex factors may influence women’s care, some referred to how they observed judgemental care in practice:

‘Women who come in who may have had other children removed, you hear others label those women as selfish and irresponsible for having a baby if they can’t look after it’ (P8)

Others highlighted how health environments fostered a culture of stigma and discrimination:

‘That happens in healthcare full stop, even in nursing people will say that those accessing A+E with drug problems, alcohol issues etc., people will stand there and say that they are draining the system’ (P3)

The VR film did, however, stimulate critical consideration of the environment on women’s vulnerability; *‘It’s strange as I’ve never been in that position myself*, *but I felt vulnerable and I didn’t like how it felt looking at my legs in stirrups*, *I felt exposed’* (P5), as well as the need to share risk factors and to minimise adversity for women. This was in relation to the vaginal examination performed within the scenario, and how this may have been traumatic for the woman with a complex history:

‘She could have a history of sexual abuse and the vaginal examination could have traumatised her, it was a man too and the midwife never asked if the woman was ok with that, she [midwife] probably didn’t have any notes she could check either as she was out of area’ [3]

With this conversation stimulating a further valuable point of reflection of how women may choose not to disclose prior abuse due to a lack of trusting relationship with their midwife; ‘*not many women are going to disclose sensitive information like that in the 15 minutes they get seeing a midwife’* (P7). Adding the critical context about Emma appeared to enhance the midwives’ ability to make connections between interpersonal interactions and the possibility of contributing to a traumatic experience.

### Reflections on educational programme

All participants provided positive feedback to the training and considered it should be offered on a mandatory basis:

‘It’s so difficult working full time and keeping up to date with CPD [continual professional development] stuff, I think it would be great to having something like this in mandatory training, it’s definitely needed’ (P5)

The most valuable aspect was around the use of VR as it afforded an experience that could not be achieved using traditional methods:

‘It’s one thing being told how to be and what not to do by someone stood at the front of a classroom, it’s another thing having those things happen to you [via VR] and you feeling helpless to do anything about it’ (P10)

VR offered a unique immersive tool to place midwives in a unique and empathic position to connect and understand women’s realities, as well as how the ‘private’ nature of the immersive experience helped to enhance reflection:

‘I thought it was great because it’s good for looking at your own behaviours and reflecting upon them, usually in training everyone feels they have to put on their best performance because everyone is watching your every move, with this [the VR training] it’s more of a private thing because you’re watching thinking ‘oh I do that’, ‘maybe I should change the way I think about that’ (P3)

Suggestions for development of the programme included to use the VR concept to train for emergency situations such as neonatal resuscitation and obstetric emergencies and filming a positive birth experience to demonstrate how care could be enhanced.

### ‘Stimulating praxis’

Following on from discussions during delivery, critical considerations of social aspects of care were also transposed into five agreed practice points that emphasised openness, empathy and continuity, namely ‘*Inclusive handover’;* ‘*Be considerate of disclosed and undisclosed history of trauma’;* ‘*One to one care in labour’;* ‘*Use positive language’* and *‘Put yourself in the woman’s shoes*.*’* Although as all respondents (in email follow-up) stated that sharing practice points was difficult, this suggests that further work is needed (and considered further in the discussion).

## Discussion

This study has provided the field of midwifery education with the potential to create reflective and immersive tools to enhance interpersonal care delivery. However, as yet VR remains an unexplored method to instigate behavioural change amongst healthcare professionals, where other approaches have so far been used to observe behaviour, not to demonstrate behavioural change [77]. As far as we are aware, there are no prior studies utilising VR that were designed to enhance reflective practices and raise awareness of interpersonal interactions during maternity care delivery. In a UK midwifery context, the findings of this study suggest that framing such approaches within a critical pedagogy can influence critical and reflexive thinking, with the potential to enable midwives, and, potentially, other health care professionals, to improve problem solving and the identification of and resistance to oppressive aspects of care [78]. Additionally, VR in healthcare education could be used as a vehicle for behavioural change, acknowledging the strong emotional experience of those who participated in this study.

Critical pedagogies aim to illuminate dehumanisation, placing emphasis on the importance of being critical of how things are and respecting the agency and capabilities of participants [79]. In this study, these insights emerged organically during reflective discussion with midwives, as they noticed and reflected on dehumanising aspects of the scenario during labour and birth. Instead of midwives receiving, filing, and storing deposits of learning material delivered, praxis was encouraged through the facilitation of reflection, theory and action embedded with the critical pedagogical approach [74]. The digital educational resources focused on enhancing relational, respectful, and humanistic care during labour and birth, and appeared to stimulate acknowledgment of political, social and inequality structures that impact upon maternity care experiences.

The adoption of technology is often faced with resistance from professionals. Critical factors include perceived ease of use; less time to interact with patients; issues with navigating complex interconnected systems; and a concern that technology will replace the human interaction in caring professions [80, 81]. In contrast, Benjamin and Jennings (2010) proposed that technological advances may sweep away oppressive aspects of a technocratic culture, pointing out the progressive possibilities in new technologies of cultural production [82]. The use of technology to enhance the user experience has been supported in the arts, especially within film, radio, and photography [49, 83, 84]. Benjamin & Jennings (2010) concur, stating how new and contemporary forms of art that utilize technology maintain their cultural power through the aura of the authentic and original [82]. Evaluation data gathered from Human Perspectives VR suggested that a key value of the education programme was its use of VR technology to stimulate affective response. The sensory experience enabled emancipatory praxis, demonstrating how technology can contribute to progressive forms of education in midwifery aimed at improving care experiences.

Immersive scenarios offer an innovative and engaging way of presenting evidence-based feedback as part of a co-collaborative approach to knowledge production and translation. First-person reflections and feedback from women in maternity care should not just serve as a vehicle to capture comfort measures but become fully integrated into the system to drive change, learning and quality improvement strategies. This novel approach to engaging participants with performative content opens up unprecedented opportunities to use immersive narratives in maternity education to improve women’s birth experiences, through the technological embodiment of the ‘other’. The creation of a private learning space within which empathic reflexivity can occur offers a powerful moment of conscientisation for facing and challenging unhelpful self-other distinctions.

It is important that innovators, researchers and educators approach the use of technology as an emancipatory endeavour with care and thought, ensuring that the correct ethical and moral frameworks are considered [85]. It is also worth acknowledging potential negative effects of VR and a resistance to its use, with considerations warranted in exploring the emotional and physical impacts associated. Digital innovations are at the forefront of strategies deployed by the DOH and NHS suggesting that technology has the power to transform services and save the NHS billions in cost and reduce health inequalities [47]. That said, caution must be applied ensuring that technology is utilized within a humanistic framework to ensure emancipatory, as opposed to exclusionary, outcomes [86]. Freire’s (2018) Pedagogical theory provides a valued reference point for considering the views and consequent behaviours that people express, and to stimulate challenge through the process of problematisation and conscientisation [64]. Recommendation for future studies include follow up interviews with participants to evaluate if their raised awareness of birth trauma and PTSD also resulted in an improvement in care delivery.

This paper has highlighted how immersive critical pedagogies could help illuminate how power structures filter into relational care practices, both in terms of how this process affects organisational cultures and how it affects communication and interpersonal interaction between maternity professionals and women during birth.

## Conclusion

The findings of this study demonstrate that Human Perspectives VR can provide an experiential form of creating and delivering immersive content for midwives, and, potentially, other health professionals such as doctors and obstetricians whose training differs from midwifery based educational programmes. It can enable participants to experience context, dialogue, spatial awareness, and emotional response within the virtual space of labour and birth. Becoming the ‘other’ was a powerful tool to stimulate educational reflective content, enhancing participating midwives’ critical awareness of practices that could cause situations of traumatic birth and subsequent PTSD. Findings from the delivery of the programme highlight the need for adequate training for midwives on traumatic birth and PTSD and a consideration for developing further immersive educational programmes. A critical approach to delivering maternity education in practice offers a space to reflect upon and challenge a nexus of power, institutional norms and practices that may lead to oppressive and impersonal care experiences, for women, birthing people, and for health care practitioners.

## Supporting information

S1 File Conscientization for practice: The design and delivery of an immersive educational programme to sensitise maternity professionals to the potential for traumatic birth experiences amongst disadvantaged and vulnerable women. (PDF)

S2 File (PDF)

S3 File (DOCX)

S4 File (DOCX)

## List of abbreviations

VR: Virtual Reality
PTSD: Post Traumatic Stress Disorder
CP: Critical Pedagogical

## References

[1] Ayers S, Bond R, Bertullies S, Wijma K. The aetiology of post-traumatic stress following childbirth: a meta-analysis and theoretical framework. Psychol Med. 2016 Apr;46(6):1121–34. doi: 10.1017/S0033291715002706 26878223

[2] Grekin R, O’Hara MW. Prevalence and risk factors of postpartum posttraumatic stress disorder: a meta-analysis. Clin Psychol Rev. 2014 Jul;34(5):389–401. doi: 10.1016/j.cpr.2014.05.003 24952134

[3] Leinweber J., Fontein‐Kuipers Y., Thomson G., Karlsdottir S. I., Nilsson C., Ekström‐Bergström A., et al. (2022). Developing a woman‐centered, inclusive definition of traumatic childbirth experiences: A discussion paper. *Birth*. 10.1111/birt.12634 35403241

[4] Fenech G, Thomson G. Tormented by ghosts from their past’: a meta-synthesis to explore the psychosocial implications of a traumatic birth on maternal well-being. Midwifery. 2014 Feb;30(2):185–93. doi: 10.1016/j.midw.2013.12.004 24411664

[5] Heyne C. S., Kazmierczak M., Souday R., Horesh D., Lambregtse-van den Berg M., Weigl T., et al. (2022). Prevalence and risk factors of birth-related posttraumatic stress among parents: A comparative systematic review and meta-analysis. *Clinical Psychology Review*, 94, 102157. doi: 10.1016/j.cpr.2022.102157 35584590

[6] Elmir R, Schmied V, Wilkes L, Jackson D. Women’s perceptions and experiences of a traumatic birth: a meta-ethnography. J Adv Nurs. 2010 Oct;66(10):2142–53. doi: 10.1111/j.1365-2648.2010.05391.x 20636467

[7] McKelvin G, Thomson G. Defence against trauma: women’s use of defence mechanisms following childbirth-related trauma. Journal of Reproductive and Infant Psychology. 2015 Apr 17;33.

[8] Redshaw M, Heikkilä K. Delivered With Care. A National Survey of Women’s Experience of Maternity Care 2010. 2010.

[9] Jayaweera H, Quigley MA. Health status, health behaviour and healthcare use among migrants in the UK: evidence from mothers in the Millennium Cohort Study. Soc Sci Med. 2010 Sep;71(5):1002–10. doi: 10.1016/j.socscimed.2010.05.039 20624665

[10] WHEC. Enablers and barriers to wellbeing–experiences of BME women in Manchester [Internet]. 2013. (85. Womens Health and Quality Consortium (WHEC) (2013) Briefing). Available from: http://www.whec.org.uk/wordpress/wp-content/uploads/downloads/2013/03/WHEC-briefing-local-roadshow-events-March-2013.pdf Last accessed 15/4/2016

[11] Psarros A. Women’s voices on health: addressing barriers to accessing primary care [Internet]. Maternity Action. 2014 [cited 2022 Mar 19]. Available from: https://maternityaction.org.uk/2014/05/womens-voices-on-health-addressing-barriers-to-accessing-primary-care/

[12] Bohren MA, Vogel JP, Hunter EC, Lutsiv O, Makh SK, Souza JP, et al. The Mistreatment of Women during Childbirth in Health Facilities Globally: A Mixed-Methods Systematic Review. PLoS Med. 2015 Jun;12(6):e1001847; discussion e1001847. doi: 10.1371/journal.pmed.1001847 26126110 PMC4488322

[13] Hodnett ED. Pain and women’s satisfaction with the experience of childbirth: a systematic review. Am J Obstet Gynecol. 2002 May;186(5 Suppl Nature):S160–172. doi: 10.1067/mob.2002.121141 12011880

[14] Feder GS, Hutson M, Ramsay J, Taket AR. Women exposed to intimate partner violence: expectations and experiences when they encounter health care professionals: a meta-analysis of qualitative studies. Arch Intern Med. 2006 Jan 9;166(1):22–37. doi: 10.1001/archinte.166.1.22 16401807

[15] Furber CM, McGowan L. A qualitative study of the experiences of women who are obese and pregnant in the UK. Midwifery. 2011 Aug;27(4):437–44. doi: 10.1016/j.midw.2010.04.001 20483513

[16] Thomson G., Diop M. Q., Stuijfzand S., & Horsch A. (2021). Policy, service, and training provision for women following a traumatic birth: an international knowledge mapping exercise. *BMC health services research*, 21(1), 1–10. 34742293 10.1186/s12913-021-07238-x PMC8571982

[17] Yildiz PD, Ayers S, Phillips L. The prevalence of posttraumatic stress disorder in pregnancy and after birth: A systematic review and meta-analysis. J Affect Disord. 2017 Jan 15;208:634–45. doi: 10.1016/j.jad.2016.10.009 27865585

[18] Dixon-Woods M, et al. Vulnerable groups and access to health care: a critical interpretive review [Internet]. 2014 [cited 2022 Mar 19]. Available from: https://www.menshealthforum.org.uk/vulnerable-groups-and-access-health-care-critical-interpretive-review

[19] Marryat L, Martin C. Growing Up In Scotland: Maternal mental health and its impact on child behaviour and development [Internet]. 2010 [cited 2022 Mar 19]. Available from: http://www.gov.scot/publications/growing-up-scotland-maternal-mental-health-impact-child-behaviour-development/

[20] De Schepper S, Vercauteren T, Tersago J, Jacquemyn Y, Raes F, Franck E. Post-Traumatic Stress Disorder after childbirth and the influence of maternity team care during labour and birth: A cohort study. Midwifery. 2016 Jan;32:87–92. doi: 10.1016/j.midw.2015.08.010 26410818

[21] Hajizadeh K, Vaezi M, Meedya S, Mohammad Alizadeh Charandabi S, Mirghafourvand M. Prevalence and predictors of perceived disrespectful maternity care in postpartum Iranian women: a cross-sectional study. BMC Pregnancy and Childbirth. 2020 Aug 14;20(1):463. doi: 10.1186/s12884-020-03124-2 32795326 PMC7427776

[22] Kassa ZY, Tsegaye B, Abeje A. Disrespect and abuse of women during the process of childbirth at health facilities in sub-Saharan Africa: a systematic review and meta-analysis. BMC Int Health Hum Rights. 2020 Sep 7;20(1):23. doi: 10.1186/s12914-020-00242-y 32894127 PMC7487593

[23] Mayra K, Matthews Z, Padmadas S. Why do some care providers abuse women during childbirth? Drivers of respect, disrespect & abuse during childbirth from midwife’s perspectives & experiences. 2020.

[24] Kramer MS, Demissie K, Yang H, Platt RW, Sauvé R, Liston R. The contribution of mild and moderate preterm birth to infant mortality. Fetal and Infant Health Study Group of the Canadian Perinatal Surveillance System. JAMA. 2000 Aug 16;284(7):843–9. doi: 10.1001/jama.284.7.843 10938173

[25] Raine R, Cartwright M, Richens Y, Mahamed Z, Smith D. A qualitative study of women’s experiences of communication in antenatal care: identifying areas for action. Matern Child Health J. 2010 Jul;14(4):590–9. doi: 10.1007/s10995-009-0489-7 19554436

[26] Hughson J-A, Marshall F, Daly JO, Woodward-Kron R, Hajek J, Story D. Health professionals’ views on health literacy issues for culturally and linguistically diverse women in maternity care: barriers, enablers and the need for an integrated approach. Aust Health Rev. 2018 Feb;42(1):10–20. doi: 10.1071/AH17067 29081348

[27] Jakobsen S, Overgaard C. “They’ll be judging us” A qualitative study of pregnant women’s experience of being offered participation in a supportive intervention. Midwifery. 2018 Feb 1;61. 10.1016/j.midw.2018.02.017 29579695

[28] Heys S, Downe S, Thomson G. ‘I know my place’; a meta-ethnographic synthesis of disadvantaged and vulnerable women’s negative experiences of maternity care in high-income countries. Midwifery. 2021 Dec 1;103:103123. doi: 10.1016/j.midw.2021.103123 34425255

[29] NICE. Pregnancy and complex social factors: a model for service provision for pregnant women with complex social factors [Internet]. NICE; [cited 2022 Mar 19]. Available from: https://www.nice.org.uk/guidance/cg110

[30] Ncube CN, Enquobahrie DA, Burke JG, Ye F, Marx J, Albert SM. Transgenerational Transmission of Preterm Birth Risk: The Role of Race and Generational Socio-Economic Neighborhood Context. Matern Child Health J. 2017 Aug;21(8):1616–26. doi: 10.1007/s10995-016-2251-2 28084576 PMC5509521

[31] Say L, Chou D, Gemmill A, Tunçalp Ö, Moller A-B, Daniels J, et al. Global causes of maternal death: a WHO systematic analysis. Lancet Glob Health. 2014 Jun;2(6):e323–333. doi: 10.1016/S2214-109X(14)70227-X 25103301

[32] MBRRACE. Saving Lives Improving Mothers’ Care—Lessons learned to inform maternity care from the UK and Ireland Confidential Enquiries into Maternal Deaths and Morbidity 2015–17 [Internet]. 2018 [cited 2022 Mar 19]. Available from: https://www.npeu.ox.ac.uk/mbrrace-uk/presentations/saving-lives-improving-mothers-care#saving-lives-improving-mothers-care-lessons-learned-to-inform-maternity-care-from-the-uk-and-ireland-confidential-enquiries-into-maternal-deaths-and-morbidity-2015%E2%80%9317

[33] Knight M. The findings of the MBRRACE-UK confidential enquiry into Maternal Deaths and Morbidity. Obstetrics, Gynaecology and Reproductive Medicine. 2019 Jan 1;29(1):21–3.

[34] Vilda D, Wallace M, Dyer L, Harville E, Theall K. Income inequality and racial disparities in pregnancy-related mortality in the US. SSM Popul Health. 2019 Dec;9:100477. doi: 10.1016/j.ssmph.2019.100477 31517017 PMC6734101

[35] Austin M-P, Hadzi-Pavlovic D, Saint K, Parker G. Antenatal screening for the prediction of postnatal depression: validation of a psychosocial Pregnancy Risk Questionnaire. Acta Psychiatr Scand. 2005 Oct;112(4):310–7. doi: 10.1111/j.1600-0447.2005.00594.x 16156839

[36] Fisher J, Cabral de Mello M, Patel V, Rahman A, Tran T, Holton S, et al. Prevalence and determinants of common perinatal mental disorders in women in low- and lower-middle-income countries: a systematic review. Bull World Health Organ. 2012 Feb 1;90(2):139–149H. doi: 10.2471/BLT.11.091850 22423165 PMC3302553

[37] Fellmeth G, Fazel M, Plugge E. Migration and perinatal mental health in women from low- and middle-income countries: a systematic review and meta-analysis. BJOG. 2017 Apr;124(5):742–52. doi: 10.1111/1471-0528.14184 27320110

[38] Howard LM, Khalifeh H. Perinatal mental health: a review of progress and challenges. World Psychiatry. 2020 Oct;19(3):313–27. doi: 10.1002/wps.20769 32931106 PMC7491613

[39] Bauer A, Parsonage M, Knapp M, Iemmi V, Adelaja B. The costs of perinatal mental health problems. 2014.

[40] Ayers S, Eagle A, Waring H. The effects of childbirth-related post-traumatic stress disorder on women and their relationships: a qualitative study. Psychol Health Med. 2006 Nov;11(4):389–98. doi: 10.1080/13548500600708409 17129916

[41] Patterson J, Hollins Martin C, Karatzias T. PTSD post-childbirth: a systematic review of women’s and midwives’ subjective experiences of care provider interaction. J Reprod Infant Psychol. 2019 Feb;37(1):56–83. doi: 10.1080/02646838.2018.1504285 30114935

[42] Thomson G, Schmied V. Psychosocial Resilience and Risk in the Perinatal Period: Implications and Guidance for Professionals. 1st ed. London; 2017.

[43] White Ribbon Alliance. Respectful Maternity Care Charter [Internet]. White Ribbon Alliance. 2011 [cited 2022 Mar 19]. Available from: https://www.whiteribbonalliance.org/respectful-maternity-care-charter/

[44] Afulani PA, Moyer CA. Accountability for respectful maternity care. Lancet. 2019 Nov 9;394(10210):1692–3. doi: 10.1016/S0140-6736(19)32258-5 31604661

[45] Bohren MA, Tunçalp Ö, Miller S. Transforming intrapartum care: Respectful maternity care. Best Pract Res Clin Obstet Gynaecol. 2020 Aug;67:113–26. doi: 10.1016/j.bpobgyn.2020.02.005 32245630

[46] Asefa A. Unveiling respectful maternity care as a way to address global inequities in maternal health. BMJ Glob Health. 2021 Jan;6(1):e003559. doi: 10.1136/bmjgh-2020-003559 33509839 PMC7845670

[47] NHS England. NHS England» Maternity Transformation Programme [Internet]. 2017 [cited 2022 Mar 19]. Available from: https://www.england.nhs.uk/mat-transformation/

[48] Krueger M. W. (1993). An easy entry artificial reality. In *Virtual Reality* (pp. 147–161). Academic Press.

[49] Shingleton RM, Richards LK, Thompson-Brenner H. Using technology within the treatment of eating disorders: a clinical practice review. Psychotherapy (Chic). 2013 Dec;50(4):576–82. doi: 10.1037/a0031815 23527906 PMC3735837

[50] Serdar K, Kelly NR, Palmberg AA, Lydecker JA, Thornton L, Tully CE, et al. Comparing online and face-to-face dissonance-based eating disorder prevention. Eat Disord. 2014;22(3):244–60. doi: 10.1080/10640266.2013.874824 24456277

[51] Fairburn CG, Patel V. The impact of digital technology on psychological treatments and their dissemination. Behav Res Ther. 2017 Jan;88:19–25. doi: 10.1016/j.brat.2016.08.012 28110672 PMC5214969

[52] Rizzo A ‘Skip’, Shilling R. Clinical Virtual Reality tools to advance the prevention, assessment, and treatment of PTSD. Eur J Psychotraumatol. 2017 Jan 16;8(sup5):1414560. 29372007 10.1080/20008198.2017.1414560 PMC5774399

[53] Platkin C, Link AR, Kwan A. The digital revolution and its potential impact on detection and treatment of depressive disorders. In: Public Health Perspectives on Depressive Disorders. 2017. p. 380–410.

[54] VRMC. The potential for virtual reality to improve health care. [Internet]. Virtual Reality Medical Center. 2016 [cited 2022 Mar 19]. Available from: https://vrphobia.com/

[55] Weiss P, Sveistrup H, Rand D, Kizony R. Video capture virtual reality: A decade of rehabilitation assessment and intervention. Physical Therapy Reviews. 2009 Oct 1;14:307–21.

[56] Alverson D, Caudell T, Goldsmith T. Creating virtual reality medical simulations: a knowledge-based design and assessment approach. In 2015. p. 411–23.

[57] Ekstrand C, Jamal A, Nguyen R, Kudryk A, Mann J, Mendez I. Immersive and interactive virtual reality to improve learning and retention of neuroanatomy in medical students: a randomized controlled study. CMAJ Open. 2018 Feb 23;6(1):E103–9. doi: 10.9778/cmajo.20170110 29510979 PMC5878950

[58] Olmos Raya E, Cavalcanti J, Contero M, Castellanos M, Alice I, Chicchi Giglioli I, et al. Mobile Virtual Reality as an Educational Platform: A Pilot Study on the Impact of Immersion and Positive Emotion Induction in the Learning Process. Eurasia Journal of Mathematics, Science and Technology Education. 2018 Feb 27;14.

[59] Hajesmaeel-Gohari S, Sarpourian F, Shafiei E. Virtual reality applications to assist pregnant women: a scoping review. BMC Pregnancy and Childbirth. 2021 Mar 25;21(1):249. doi: 10.1186/s12884-021-03725-5 33765969 PMC7993522

[60] Jones D, Fealy S. Immersive technology in maternity care: Anatomy in the Digital Age. In 2020 [cited 2022 Mar 19]. Available from: https://www.youtube.com/watch?v=U2TyG4_nG7Y&feature=youtu.be

[61] Hardie P, Darley A, Carroll L, Redmond C, Campbell A, Jarvis S. Nursing & Midwifery students’ experience of immersive virtual reality storytelling: an evaluative study. BMC Nurs. 2020;19:78. 32821245 10.1186/s12912-020-00471-5 PMC7433077

[62] Diemer MA, McWhirter EH, Ozer EJ, Rapa LJ. Advances in the Conceptualization and Measurement of Critical Consciousness. Urban Rev. 2015 Dec 1;47(5):809–23.

[63] Laros A, Fuhr T, Taylor E. Transformative Learning Meets Bildung: An International Exchange. 2017.

[64] Kuipers Y. J., Thomson G., Goberna-Tricas J., Zurera A., Hresanová E., Temesgenová N., et al. (2022). The social conception of space of birth narrated by women with negative and traumatic birth experiences. *Women and Birth*. doi: 10.1016/j.wombi.2022.04.013 35514007

[65] Wallerstein N, Bernstein E. Empowerment education: Freire’s ideas adapted to health education. Health Educ Q. 1988;15(4):379–94. doi: 10.1177/109019818801500402 3230016

[66] Graneheim UH, Lundman B. Qualitative content analysis in nursing research: concepts, procedures and measures to achieve trustworthiness. Nurse Educ Today. 2004 Feb;24(2):105–12. doi: 10.1016/j.nedt.2003.10.001 14769454

[67] Alcorn KL, O’Donovan A, Patrick JC, Creedy D, Devilly GJ. A prospective longitudinal study of the prevalence of post-traumatic stress disorder resulting from childbirth events. Psychol Med. 2010 Nov;40(11):1849–59. doi: 10.1017/S0033291709992224 20059799

[68] Thomson G, Downe S. Emotions and support needs following a distressing birth: Scoping study with pregnant multigravida women in North-West England. Midwifery. 2016 Sep;40:32–9. doi: 10.1016/j.midw.2016.06.010 27428096

[69] Elsaesser T, Hagener M. Film Theory: An Introduction through the Senses - 2nd Edition—Thomas [Internet]. 2015 [cited 2022 Mar 19]. Available from: https://www.routledge.com/Film-Theory-An-Introduction-through-the-Senses/Elsaesser-Hagener/p/book/9781138824300

[70] Sharma M, Romas J. Theoretical Foundations of Health Education and Health Promotion. Jones & Bartlett Publishers. 2012.

[71] Matthews C. Critical pedagogy in health education. Health Education Journal. 2014 Sep 1;73:600–9.

[72] Nyirenda J. The relevance of Paulo Freire’s contributions to education and development in present day Africa. undefined [Internet]. 1996 [cited 2022 Mar 19]; Available from: https://www.semanticscholar.org/paper/The-relevance-of-Paulo-Freire%27s-contributions-to-in-Nyirenda/f084df7013cfd94c78df0f501ba631567d972e73

[73] Ledwith M. Emancipatory Action Research as a Critical Living Praxis: From Dominant Narratives to Counternarratives. In 2017. p. 49–62.

[74] Freire P. Pedagogy of the oppressed. New York: Herder: Myra Bergman Ramos; 1968.

[75] Andreotti V, Souza L. Global learning in the “knowledge society”. Four tools for discussion. Zep Zeitschrift Fur Internationale Bildungsforschung Und Entwicklungspadagogik [Internet]. 2008 Jan 1 [cited 2022 Mar 19]; Available from: https://www.academia.edu/25832540/Global_learning_in_the_knowledge_society_Four_tools_for_discussion

[76] Braun V, Clarke V. Using thematic analysis in psychology. Qualitative Research in Psychology. 2006 Jan 1;3:77–101.

[77] Fertleman C, Aubugeau-Williams P, Sher C, Lim A-N, Lumley S, Delacroix S, et al. A Discussion of Virtual Reality As a New Tool for Training Healthcare Professionals. Front Public Health. 2018 Feb 26;6:44. doi: 10.3389/fpubh.2018.00044 29535997 PMC5834832

[78] Halman M, Baker L, Ng S. Using critical consciousness to inform health professions education. Perspect Med Educ. 2017 Feb;6(1):12–20. 28050879 10.1007/s40037-016-0324-y PMC5285284

[79] Rosenberger C. Beyond Empathy: Developing Critical Consciousness Through Service Lear [Internet]. 2014 [cited 2022 Mar 19]. Available from: https://www.taylorfrancis.com/chapters/edit/10.4324/9781410606051-9/beyond-empathy-developing-critical-consciousness-service-learning-cynthia-rosenberger

[80] Gagnon M-P, Ngangue P, Payne-Gagnon J, Desmartis M. m-Health adoption by healthcare professionals: a systematic review. J Am Med Inform Assoc. 2016 Jan;23(1):212–20. doi: 10.1093/jamia/ocv052 26078410 PMC7814918

[81] Piwek L, Ellis DA, Andrews S, Joinson A. The Rise of Consumer Health Wearables: Promises and Barriers. PLoS Med. 2016 Feb 2;13(2):e1001953. doi: 10.1371/journal.pmed.1001953 26836780 PMC4737495

[82] Benjamin W, Jennings M. The Work of Art in the Age of Its Technological Reproducibility [First Version]. Grey Room. 2010;(39):11–38.

[83] Jameson F. Postmodernism, or The Cultural Logic of Late Capitalism. New Left Rev. 1984 Aug 1;(I/146):53–92.

[84] McRobbie A. Postmodernism and Popular Culture [Internet]. 2003 [cited 2022 Mar 19]. Available from: https://www.routledge.com/Postmodernism-and-Popular-Culture/McRobbie-Mcrobbie/p/book/9780415077132

[85] Huda M. Empowering application strategy in the technology adoption: Insights from professional and ethical engagement. Journal of Science and Technology Policy Management. 2018 Mar 9;10.

[86] Yaldren J, Van Loo M. Technology and inclusivity. British Journal of Cardiac Nursing. 2018 Feb 21.

