# Supplementary material for: The design, delivery and evaluation of ‘Human Perspectives VR’: An immersive educational programme designed to raise awareness of contributory factors for a traumatic childbirth experience and PTSD
Source: PLoS One. 2022 Nov 2;17(11):e0276263. doi: 10.1371/journal.pone.0276263 (PMC9629609; doi:10.1371/journal.pone.0276263)
Supplement: S1 File — (PDF) [file pone.0276263.s001.pdf]

Conscientization for practice: The design and delivery of an immersive educational programme to sensitise maternity professionals to the potential for traumatic birth experiences amongst disadvantaged and vulnerable women.

By

Stephanie Heys

A thesis submitted in partial fulfilment for the requirements for the degree of Doctor of Philosophy at the University of Central Lancashire

June 2019

Type of Award **Doctor of Philosophy**

School **School of Community Health & Midwifery**

1. **Concurrent registration for two or more academic awards**

I declare that while registered as a candidate for the research degree, I have not been a registered candidate or enrolled student for another award of the University or other academic or professional institution

____

2. **Material submitted for another award**

I declare that no material contained in the thesis has been used in any other submission for an academic award and is solely my own work

____

3. **Collaboration**

Where a candidate's research programme is part of a collaborative project, the thesis must indicate in addition clearly the candidate's individual contribution and the extent of the collaboration. Please state below:

____

4. Use of a Proof-reader

No proof-reading service was used in the compilation of this thesis.

Signature of Candidate ____

Print name: ____

ABSTRACT

Birth is an important time in a woman's life. While the journey into motherhood can be a transformational and liminal experience, unfortunately, this is not the case for every woman. It is estimated that approximately 30 % of women experience childbirth as a traumatic event, with up to 4% of women in community samples developing Post Traumatic Stress Disorder (PTSD) following childbirth. It is also highlighted that women who are vulnerable and disadvantaged, due to complex life situations such as poor mental health, poverty and social isolation, are more likely to experience birth trauma and PTSD onset. Recent research highlights that women's subjective experience of birth is one of the most important factors in determining birth trauma, and that negative interactions with health care professionals are a key contributor to its development. The aim of this study was to develop and evaluate a training programme for maternity care providers to raise awareness of birth trauma amongst disadvantaged and vulnerable women. A critical pedagogical approach was adopted so that the design of the programme would aid reflection, critical thinking and conscientization.

This study includes a meta-ethnographic review, empirical interviews and the design and delivery of a tailored educational programme within an NHS Trust. Firstly, a meta-ethnography was undertaken to explore disadvantaged and vulnerable women's negative experiences of maternity care in high income countries. Noblit & Hare's (1988) meta-ethnographic approach was used and four themes were identified through the synthesis of eighteen studies; '*Depersonalisation*' '*Dehumanisation*', '*Them & us'* and '*No care in the care'*. Secondly, ten local disadvantaged and vulnerable women in North West of England were recruited and interviewed, exploring their negative experiences of birth. A framework analysis was used to interpret the data, identifying key triggers for birth trauma, focused on interpersonal interactions with maternity healthcare professionals. These findings were then compared against studies included in the meta-ethnography. Following these stages an innovative educational programme focused on birth trauma and PTSD was developed and evaluated. Key findings from the meta-ethnography and the empirical interviews informed the content of a filmed childbirth scenario that was embedded within a critical pedagogical framework. The scenario was delivered to participants' using virtual reality (VR) technology, forming part of a 90-minute educational programme, in which maternity professionals view the scenario

from a first-person perspective. Other elements of the education programme involved providing statistical evidence on birth trauma and PTSD, a presentation of qualitative data collected during empirical phases, critical reflections and the development of actionable practice points to change/influence care practice, for self and others. Ten maternity professionals participated in the evaluation, with pre/post questionnaires and a follow-up session used to assess participants attitudes, knowledge and experiences prior, during and following attendance. Findings suggest the immersive educational programme increased participants understanding and knowledge of birth trauma and PTSD, with the use of VR as a tool for knowledge translation found to enhance critical reflection and facilitate praxis. While further research to test the efficacy of the educational programme on women's birth experiences is needed, simulated first person realities, embedded within a critical pedagogical framework, offer a unique and innovative approach to addressing interpersonal care in maternity and wider health-related contexts of care.

CONTENTS

ABSTRACT ii
ACKNOWLEDGMENTS xii
EPIGRAPH xiii
LIST OF TABLES xiv
LIST OF FIGURES xiv
LIST OF IMAGES xv
ABRIEVIATIONS xv
CHAPTER 1: OVERVIEW OF THE RESEARCH 17
1.1 The research problem 17
1.2 Research funding 18
1.3 Research aim 18
1.3.1 Research objectives 19
1.4 Thesis overview 19
1.5 Organisation of thesis 20
CHAPTER 2: BACKGROUND CHAPTER 23
2.1 Introduction 23
2.2 Birth trauma 23
2.2.1 Impact of a traumatic birth 24
2.2.2 Incidence and prevalence of traumatic birth experiences & PTSD 26
2.2.3 Risks & predictors of traumatic birth experiences & PTSD 27
2.2.4 Theoretical basis for PTSD 29
2.3 Disadvantaged and vulnerable women in maternity care 30
2.3.1 Health inequalities amongst disadvantaged and vulnerable women 32
2.4 Health inequalities in midwifery care: A global perspective 33
2.5 Neoliberalism and social determinants of health amongst childbearing women 34
2.6 The impact of austerity upon provisions of maternity care 36
2.7 Othering in maternity care 37
2.7.1 Intersectionality in a midwifery context 38
2.8 The physical and psychological impact of poor interpersonal care. 39
2.8.1. Iatrogenic impact of birth 39
2.9 Specialist care for disadvantaged and vulnerable women 40
2.10 Current training on birth trauma and complex issues 42
2. 11 Addressing cultural and organisational failings impacting upon maternity care 43

2. 12 Trauma informed approaches to maternity services 44
2.13 A co-productive approach to service design: listening to women's voices 45
2. 14 Digital innovation in education 46
2.15 Current advances in digital education 47
2.16 Rationale for study 48
2. 17 Conclusion 49
CHAPTER 3: META-ETHNOGRAPHY 51
3.1 Introduction 51
3.2 Aim of the systematic review 51
3.3 Research question 52
3.4 What is a systematic review? 52
3.5 Methodology 52
3.5.1 Meta-ethnography 52
3.6 The search (phase 1) 53
3.7 Enhancing retrieval methods in databases 55
3.7.1 Berry picking 56
3.8 Methods used during the search 56
3.8.1 Database searches 56
3.8.2 Area scanning / journal run 57
3.8.3 Footnote / reference chasing 57
3.8.4 Citation tracking 57
3.8.5 Author run 57
3.8.6 Personal knowledge resources 57
3.9 Deciding what is relevant to the initial interest (Phase 2) 57
3.9.1 Full text screening 59
3.9.2 Quality appraisal 59
3.10 Findings 61
3.11 Initial reading and familiarisation (phase 3) 63
3.12 Determining how studies are related (phase 4) translating studies (phase 5) synthesising translations (phase 6) 64
3.13 Expressing the synthesis (Phase 7) 66
3.14 'Depersonalization' 66
3.14.1 Disengagement 67
3.14.2 Lack of culturally contextual care 67
3.15 'No care in the care' 69
3.15.1 Poor emotional connections 69

3.15.2 Submissive interactions 70
3.16 'Dehumanisation' 72
3.16.1 Demoralising interactions 72
3.16.2 Absence of human rights in maternity care 73
3.17 'Them and us' 74
3.17.1 Judgemental attitudes 75
3.17.2 Inferiority complex as a result of interactions 76
3.18 Line of argument synthesis 78
3.19 Summary of key findings 78
3.20 Conclusion 78
CHAPTER 4: THEORETICAL CHAPTER 80
4.1 Introduction 80
4.2 SECTION 1 80
4.2.1 Epistemological and ontological perspectives. 80
4.2.2 Social constructionism 81
4.2.3 Addressing issues of power through a social constructionist lens 82
4.2.4 Interpretivist approaches to research inquiry 84
4.2.5 Otherness 85
4.2.6 Social abjection 86
4.2.7 Testimonial & hermeneutic injustice and power 88
4.3 SECTION 2 89
4.3.1 Introduction 89
4.3.2 Critical social theory 90
4.3.3 Hegemony 92
4.3.4 Counter Hegemony 94
4.3.5 From Gramsci to Freire: A critical pedagogical focus for midwifery education 95
4.4 SECTION 3 96
4.4.1 Introduction 96
4.4.2 Paulo Freire 96
4.4.3 Critical pedagogies 97
4.4.4 Critical consciousness 'conscientization' 100
4.4.5 Critical pedagogies in healthcare education 100
4.4.6 Culture circles 102
4.4.7 Theoretical synergies: Using critical consciousness to inform midwifery education 102
4.5 Reflections 103
4.6 Conclusion 104

CHAPTER 5: METHODS 106
5.1 Introduction 106
5.2 Critical methods 106
5.3 Interviews with disadvantaged and vulnerable women 107
5.3.1 Engaging participants 107
5.3.2 Sampling methods 107
5.3.3 Sample size / recruitment process 108
5.3.4 Inclusion criteria 108
5.3.5 Recruitment 108
5.3.6 Data collection 110
5.3.7 Style of engagement 111
5.3.8 Transcribing and storing the data 113
5.4 Data analysis 113
5.5 The framework method 113
5.6 The benefits of using a framework approach 114
5.7 Stage 1 Transcription 115
5.8 Stage 2 Familiarization with the interviews 115
5.9 Stage 3 Coding 115
5.10 Stage 4 Developing a working analytical framework 116
5.11 Stage 5 Applying the analytical framework 117
5.12 Stage 6 Charting data into the framework matrix 117
5.13 Step 6.1 Additional step to cross reference triggers for birth trauma across studies within the meta-ethnography 118
5.14 Step 7 Interpreting the data 119
5.15 Limitations of framework approach 119
5.16 The design and delivery of an immersive education programme for midwives using VR technology 120
5.16.1 Choosing an approach to design a critical pedagogy 120
5.11 Overview of the three-phase critical pedagogy model 122
5.12 Step 1 listening and naming 123
5.12.1 Codification 124
5.12.2 Using narratives with a critical pedagogy 124
5.12.3Writing a script 125
5.12.4 Considering perspectives 126
5.12.5 Filming - Technical aspects 127
5.12.6 Choosing actors 128
5.13 Step 2 Dialogue and reflection 129

5.13.1 Theory based session. Adding context 130
5.14 Step 3 The promotion of transformative social action 130
5.14.1 Practice points 131
5.15 Recruitment of participants to attend the immersive educational programme 131
Introduction 131
5.15.1 Study site 132
5.15.2 Sample strategy 132
5.16 Addressing rigour 133
5.16.1 Credibility 133
5.16.2 Confirmability 134
5.16.3 Transferability 134
5.16.4 Dependability 135
5.17 Methods for evaluation of the educational programme 136
5.17.1 Pre and post questionnaires 136
5.17.2 Focus group follow up 137
5.17.3 Data analysis 137
5.18 Ethics 137
5.18.1 Ethical principles 138
5.18.2 Ethical considerations 138
5.19 Conclusion 140
CHAPTER 6. FINDINGS 141
6.1 Introduction 141
6.2 Participants 141
6.3 Reflections on recruitment and data collection 142
6.4 Identifying triggers for traumatic experiences across the data set 143
6.4.1. Trigger 1: A lack of emotional support 144
6.4.2. Trigger 2: Poor information giving 146
6.4.3. Trigger 3 Poor use of language 147
6.4.4. Trigger 4: Unconsented interventions 147
6.4.5. Trigger 5 Submissive interactions 148
6.4.6. Trigger 6: Judgemental attitudes 149
6.5 Positive aspects of the data 150
6.6 Mapping key triggers within papers identified in the meta-ethnography 151
6.7 Conclusion 153
CHAPTER 7: DEVELOPING AN IMMERSIVE EDUCATION PROGRAMME 154
7.1 Introduction 154

7.2 Script & filming. Mapping triggers for trauma within the narrative 154
7.3 The script....... 155
7.4 Filming....... 155
7.5 Directing the day....... 156
7.6 The film 156
7.6.1 Scene 1: Introduction to the film 158
7.6.2 Scene 2. Summary of content. Discussion with the midwife following a vaginal examination. 158
Image 5 scene 2 synopsis 158
7.6.3 Scene 3. Summary of content midwife and doctor conversation. 158
7.6.4 Scene 4. Summary of content. Discussion with doctor. 159
7.6.5 Scene 5. Summary of content. Cannulation and reduction in fetal heart rate. 159
7.6.6. Scene 6. Summary of content. Reviewing the CTG. 160
7.6.7. Scene 7. Summary of content. Vaginal examination. 160
7.6.8. Scene 8. Summary of content decision for a caesarean section, preparing for theatre. 160
7.6.9. Scene 9. Summary of content alone in the room. End of film. 161
7.7 Overview of Educational Programme 161
7.7 Conclusion....... 163
CHAPTER 8: FINDINGS FROM EVALUATION PHASE. THE DELIVERY OF AN IMMERSIVE CRITICAL PEDAGOGY....... 164
8.1 Introduction 164
8.2 Running the event....... 164
8.4 Participants 166
8.5 Venue 166
8.6 Delivering a critical pedagogy, ‘*Building trust’* 166
8.7 Reflective thoughts ‘*Becoming the other’* 167
8.8 Professional reflections and sharing experiences ‘*Nurturing professional empathy and understanding*’ 169
8.9 Providing the theory ‘*What, why, when’* 171
8.10 Addressing the context ‘*Critical thinking’* 172
8.11 Critiquing the system *‘Fit for purpose?’* 175
8.12 Creating critical knowledge *‘Practice Points’* 177
8.13 Pre/post questionnaire findings....... 179
8.14 Exploring the experience of VR 181
8.15 Follow up questions 182
8.16 Conclusion....... 183

CHAPTER 9: DISCUSSION 185
9.1 Introduction 185
9.2 Summary of study 185
9.3 Unique contribution to knowledge 186
9.4 Central interpretive findings 187
9.5 '*Them & us'* a neoliberal crisis in maternity care? 188
9.6 Deconstructing the *'other'* in maternity care 190
9.7 Epistemic injustices in maternity care practices 192
9.8 Collective efficacy – a critical approach to change 193
9.9 Tensions between policy and practice 194
9.10 The value of narratives in conscientization 195
9.11 Complexities of technological contributions to critical pedagogy 196
9.12 VR - a tool for 'Conscientization'? 198
9.13 Conclusion 199
9.14 Implications of study 200
9.14.1 Implications for policy and practice 200
9.14.2 Implications for future research 201
9.14.3 An emerging framework for creating immersive educational resources in healthcare 202
9.14.4 Strengths of the study 203
9.14.5 Limitations of the study 204
CHAPTER 10: TRANSLATING ACADEMIC OUTPUTS INTO TOOLS FOR IMPACT 207
10.1 Introduction 207
10.2 Dissemination: developing a campaign and marketing strategy 207
10.3 Working across-disciplines in healthcare innovation: developing a module descriptor 209
10. 4 Business school presentations 212
10. 5 Summary 212
10. 6 Conclusion 213
CHAPTER 11: PERSONAL REFLECTIONS 214
11.1 Introduction 214
11.2 Personal journey: Becoming an academic 214
11.3 Climbing the ladder 215
11.4 Professional identity and conflicting feelings 216
11.5 Final thoughts 217
REFERENCES 219
APPENDICES **Error! Bookmark not defined.**

Appendix 1 Database searches .... **Error! Bookmark not defined.**

Appendix 2 Initial screen full text papers................................. **Error! Bookmark not defined.**

Appendix 3 Quality assessment. Study codes quality grades and characteristics of included studies .... **Error! Bookmark not defined.**

Appendix 4 Participant information sheet for women's interviews .... **Error! Bookmark not defined.**

Appendix 5 Sociodemographic form for women during recruitment phase .. **Error! Bookmark not defined.**

Appendix 6 Contact information sheet .... **Error! Bookmark not defined.**

Appendix 7 Consent form .... **Error! Bookmark not defined.**

Appendix 8 Interview guide .... **Error! Bookmark not defined.**

Appendix 9 Example of conversational transcript of interview . **Error! Bookmark not defined.**

Appendix 10 Script 'Emma's Story' .... **Error! Bookmark not defined.**

Appendix 11 Prototype explanation .... **Error! Bookmark not defined.**

Appendix 12 Participant information sheet for participation in the VR educational programme .... **Error! Bookmark not defined.**

Appendix 13 Contact information sheet .... **Error! Bookmark not defined.**

Appendix 14 Consent form for participation in the VR educational programme .... **Error! Bookmark not defined.**

Appendix 15 Pre-intervention questionnaire .... **Error! Bookmark not defined.**

Appendix 16 Post intervention questionnaire .... **Error! Bookmark not defined.**

Appendix 17 Practice point template .... **Error! Bookmark not defined.**

Appendix 18 Focus group schedule .... **Error! Bookmark not defined.**

Appendix 19 The University of Central Lancashire ethics approval .... **Error! Bookmark not defined.**

Appendix 20 The Health Research Authority HRA approval letter .... **Error! Bookmark not defined.**

Appendix 21 The University of Central Lancashire Ethics approval .... **Error! Bookmark not defined.**

Appendix 22 Module descriptor .... **Error! Bookmark not defined.**

Appendix 23 Winning marketing and campaign strategy presentation ... **Error! Bookmark not defined.**

Appendix 24 Winning marketing and campaign strategy report. .... **Error! Bookmark not defined.**

ACKNOWLEDGMENTS

My gratitude and thanks go firstly to the women who kindly shared their stories with me. This gratitude extends to all women around the world who speak out in the quest to help others, thank you.

I would like to acknowledge my supervisor Dr Gill Thomson. Gill, I will be eternally grateful for the compassion, guidance and support you have given me throughout this journey. Your belief in me gave me the courage to begin this journey and your encouragement has helped me to finish it, thank you. I would also like to thank my other supervisors, Professor Soo Downe for being the ultimate role model in midwifery research, your dedication and passion for midwifery is undeniable, you are a true inspiration and most importantly, kind. My thanks extend to the wonderful Sheena Byrom for her guidance, support and love. I feel so lucky to have such a dynamic and inspiring group of women to guide me as I begin my research career. Professor Mick McKeown, Mick, I know you joined the team late, but your presence has been invaluable both on an academic and a personal level, a true comrade, thank you. I would also like to thank Jeanette Fegan for helping me through the difficult times.

A special thanks also goes to those involved in the planning and design of the immersive scenario, Alex Keelan, Luke Nelson, David Gregan, Jade Golding, Amy Forrest, Cathy Atherton and Emma Ashton. I would also like to thank my fellow PhD friends, Louise, Anam, Claire, Naoimh, Flo, Sam and Anna. You have all lent me a listening ear and kind word when I needed them the most and for that I am so very grateful, I couldn't have done it without you all.

This thesis would also not have been possible without the support from my partner Liam. You joined me on this journey as a friend and ended up my long-suffering other half. You have supported me all the way, thank you for reminding me to laugh amidst all the study and for providing me with perspective when I needed it the most. Lastly, and most importantly, I would like to dedicate this thesis and all I do to my kind, loving and beautiful daughter Abigail. You are my best friend. Your love, patience and kindness has given me strength when I thought I had nothing left to give. This one's for you Kid.

EPIGRAPH

'Human existence cannot be silent, nor can it be nourished by false words, but only by true words, with which people transform the world. To exist, humanly, is to name the world, to change it. Once named, the world in its turn reappears to the name's as a problem and requires of them a new naming. People are not built in silence, but in word, in work, in action-reflection' (Freire 1972, p. 143)

LIST OF TABLES

Table 1	Classification of disadvantaged and vulnerable characteristics amongst childbearing women	p. 15
Table 2	PEO framework and search terms	p. 37
Table 3	Inclusion exclusion criteria	p. 40
Table 4	Walsh & Downes' scoring criterial for quality appraisal	p. 42
Table 5	Study codes. Summary of characteristics and grades	p. 44
Table 6	Synthesis interpretations	p. 47
Table 7	Example of interview transcription coding undertaken on interview transcripts	p. 97
Table 8	The application of codes to interview transcripts	p. 98
Table 9	Refining and reconceptualising the codes	p. 99
Table 10	Characteristics of women interviewed	p. 122
Table 11	Themes emerging from the data using the framework method	p. 125
Table 12	Triggers mapped against papers included in the meta-ethnography	p. 132
Table 13	Triggers for birth trauma mapped to each scene of the film	P. 138
Table 14	Overview of the immersive educational programme mapped to the three-step model of designing a critical pedagogy	p. 149
Table 15	Evaluation stages mapped to stages within the immersive educational programme	p. 152
Table 16	Pre and post questionnaire results	p. 167
Table 17	PESTEL tool used to map the study within a marketing and business framework	p. 194

LIST OF FIGURES

Figure 1	Noblit & Hare (1988) seven phase meta-ethnography	p. 35
Figure 2	Prisma diagram	p. 44
Figure 3	Overview of the immersive educational programme	p. 102
Figure 4	Stages in the development of the script	p. 106

Figure 5	The SHOWED model	p. 110
Figure 6	List of key components included within immersive critical pedagogies	P. 195

LIST OF IMAGES

Image 1	Prototype testing	p. 108
Image 2	The birthing room	p. 137
Image 3	Observing and directing	p. 138
Image 4	Scene 1 synopsis. Introduction to the film	p. 140
Image 5	Scene 2 synopsis. Discussion with the midwife following vaginal examination.	p. 141
Image 6	Scene 3 synopsis. Midwife and doctor conversation	p. 142
Image 7	Scene 4 synopsis. Discussion with doctor	p. 143
Image 8	Scene 5 synopsis. Cannulation and reduction of fetal heart rate	p. 144
Image 9	Scene 6 synopsis. Reviewing the CTG	p. 145
Image 10	Scene 7 synopsis. Vaginal examination	p. 146
Image 11	Scene 8 synopsis. Decision for a caesarean section; preparing for theatre	p. 147
Image 12	Scene 9 synopsis. Alone in the room	p. 148
Image 13	Evaluation of programme location and room layout	p. 154

ABRIEVIATIONS

DOH	The Department of Health
NMC	The Nursing and Midwifery Council
CLAHRC	Collaboration for Leadership in Applied Health Research and Care
NIHR	National Institute for Health Research
WHO	World Health Organization
NHS	The National Health Service

PTSD	Post-Traumatic Stress Disorder
DSM	American Psychiatric Association's Diagnostic and Statistical Manual
BAME	Black Asian and Minority Ethnic
FA	Framework Analysis
POV	Point of View
HEE	Health Education England
CTG	Cardiotocography
LMIC	Low- and middle-income counties
AHCP	Allied Health Care Professionals
AHSN	Academic Health Science Networks
CCG	Clinical Commissioning Groups

CHAPTER 1: OVERVIEW OF THE RESEARCH

1.1 The research problem

Despite government initiatives, health inequalities persist in the National Health Service (NHS). In 2012 the Health and Social Care Act placed a duty on the Secretary of State, NHS England and Clinical Commissioning Groups to address inequalities in health (Health and Social Care Act, 2012). However, recent statistics reveal that teenage mothers, those from socioeconomically disadvantaged areas and Black, Asian and Minority Ethnic (BAME) communities are more likely to have mental health issues, have a higher mortality rate and have a higher rate of long-term conditions associated with their lifestyle (Office for National Statistics 2011, Department of Health 2012a, 2012b, Public Health England 2014). In 2007 The World Health Organization (WHO) also identified that those from disadvantaged socioeconomic backgrounds were more likely to receive poor care practices from maternity providers and to experience a traumatic birth (Houweling et al., 2007). As women who need support following a traumatic birth can feel unable to access help due to their experiences of the health care system (Gamble et al., 2002, Ayers, Claypool & Eagle 2006), this can create a wider inequality gap amongst disadvantaged and vulnerable women (Miller et al., 2016).

As society becomes more multi-racial and multi-cultural, maternity professionals are increasingly required to provide maternity services to women from diverse backgrounds and to those who have complex needs. The recent NHS England Maternity Service Review committed to delivering equitable and culturally sensitive care in maternity services to reduce healthcare disparities (NHS England, 2016). However, a lack of educational resources to address these issues has left NHS Trusts unequipped to deal with complex and diverse minority groups (Ross-Davie et al., 2006, Jomeen et al., 2009). This research aims to increase the resources available to staff in this area by developing a feasible and acceptable educational programme for maternity professionals to reduce the number of disadvantaged and vulnerable women experiencing a traumatic birth.

To achieve this, I have adopted a critical approach, engaging with critical theories when exploring the lived experiences of disadvantaged and vulnerable women and during the design and delivery of an immersive educational programme for maternity professionals. An underpinning principle of this thesis relates to *'conscientization'*. Also known as critical consciousness, conscientization is an educational and social concept

developed by Paulo Freire, a Brazilian critical educationalist and philosopher (Freire, 1972). Conscientization is concerned with achieving an in depth understanding of the world by illuminating social and political contradictions. This practice includes acting against oppressive elements and challenging inequalities of treatment or opportunity. Conscientization involves being aware of these conditions to emancipate the oppressed. Using the works and theories of critical thinkers and critical educationalists, critiques of power are at the centre of the analysis within this study.

1.2 Research funding

My doctoral studies were funded by the North West Coast Collaboration for Leadership in Applied Health Research and Care (CLAHRC). In 2008 the National Institute for Health Research (NIHR) created nine regional CLAHRC's across the UK to undertake research activities, enhance the implementation of findings into practice and build capacity across organisations for generating and utilising evidence (Martin et al., 2011).

The CLAHRC North West Coast (NWC) aims to reduce health inequalities in the region in relation to poor health outcomes and chronic issues faced by the socioeconomically deprived population in the North West. Fifty percent of the poorest neighbourhoods are found in the North West suffering considerably higher numbers of deprivation, health inequalities and poor health (Popay et al., 2017). The CLAHRC NWC received funding over a five-year period to conduct research focused on key issues faced in the region to build relationships with NHS organisations and to increase the availability of high-quality health care by investing and working with local and national initiatives (Harvey et al., 2011). The CLHARC's have nurtured collaborative relationships between clinical practice and academic institutions to enhance the uptake of evidence-based interventions and data from specifically focused and targeted research activity (Currie et al., 2013). To date these interventions have had a positive impact on the wellbeing of local communities and encouraged and implemented patient focused research, engaging the public in influencing health outcomes through empowerment and education.

1.3 Research aim

The main aim of this study was to develop and deliver a feasible and acceptable educational programme for maternity professionals to reduce the number of disadvantaged and vulnerable women experiencing a traumatic birth and the development of post-traumatic stress disorder (PTSD).

1.3.1 Research objectives

- To undertake a literature review and a meta-ethnography focused on disadvantaged and vulnerable women's experiences of maternity care in high income countries, with a focus on negative birth experiences.

- To explore disadvantaged and vulnerable women's negative experiences of maternity professionals during childbirth within a locality, using semi-structured interviews.

- To use key findings from the meta-ethnography and interviews to inform the design of a tailored educational programme to reduce birth trauma experiences and potential PTSD amongst disadvantaged and vulnerable women.

- To explore maternity professionals' views and experiences of a tailored educational programme in terms of the comprehensibility, acceptability, utility, ease of use, and feasibility.

- To explore the impact of the programme on participants awareness, knowledge and critical thinking regarding contributory factors to birth trauma and how the programme may or may not influence practices.

1.4 Thesis overview

During phase one of the study a meta-ethnography was undertaken of 18 studies which explored interpersonal interactions between disadvantaged and vulnerable women and maternity health care professionals in high-income countries, during the childbearing continuum. Four third-order interpretations were developed: *'Depersonalisation', 'No care in the care', 'Dehumanisation'* and '*Them and us*'.

Phase two of the study consisted of semi-structured interviews with ten disadvantaged and vulnerable women who had experienced a traumatic birth in the North West of England. Framework analysis was used to identify key triggers for birth trauma during interpersonal interactions with maternity healthcare professionals, with these findings then mapped against studies included in the meta-ethnography. Issues faced by disadvantaged and vulnerable women were identified through these stages. These

included an identification of judgements and biases in relation to women's social positioning when accessing maternity care, alongside a lack of cultural and social awareness from healthcare professionals.

Phase three of the study included the design of a tailored educational programme using innovative educational approaches to enhance the learning experience. A three-step model for designing critical pedagogies was adopted during the design phase. Triggers for trauma identified during empirical stages (meta-ethnography / interviews) were used to develop a filmed immersive scenario that was presented to participants via virtual reality (VR) technology. Other elements of the training programme included reflective discussions, group work, a presentation and the development of action points for practice, forming part of a 90-minute immersive educational programme.

In the last phase of this study, an evaluation of the programme was undertaken within an NHS maternity Trust in the North West of England. The delivery included a consideration of the practical application and acceptability of using a critical pedagogy in maternity education, whilst exploring the use of VR as an experiential approach to enhancing critical reflections. Pre and post questionnaires and a follow up email questionnaire six week after the programme were used to assess participants' experiences and knowledge following attendance. Findings suggest the programme raised awareness of contributory factors associated with the development of birth trauma and PTSD. Participants reported positive experiences of using virtual reality as a learning tool, expressing how this enhanced their critical awareness of the social space of birth and the impact of interpersonal interactions on women's birth experiences. Following delivery, a cross collaborative approach was taken working with the University of Central Lancashire's Business School to develop a campaign and marketing strategy for the finished programme to aid its adoption in practice.

1.5 Organisation of thesis

An overview of each of the chapters is provided as follows:

Chapter 2 provides an overview of current research and debates surrounding each strand of this thesis. Topics relating to the thesis within this section include birth trauma and PTSD amongst disadvantaged and vulnerable women, the social space of birth,

current training for maternity professionals and innovative approaches to the development and delivery of specialised programmes in maternity education. This chapter concludes with a question that is not currently addressed within the literature.

Chapter 3 presents findings from a systematic review and meta-ethnographic synthesis focused on the negative experiences of maternity care amongst disadvantaged and vulnerable women in high income countries. Four themes are presented and discussed.

Chapter 4 outlines the research context with regards to underpinning theory and methodology. The rationale for the selection of a critical theoretical approach is presented. This is followed by an outline of the seminal work of Paulo Freire; '*Pedagogy of The Oppressed*', a text concerned with the design of critical pedagogies in education to aid *'Conscientization'*.

Chapter 5 describes and discusses the methods chosen for the study. This chapter details how disadvantaged and vulnerable women were identified, recruited, data collection methods chosen, consideration of ethical issues, as well as the modified framework method used to analyse the data. The design of the educational programme is also discussed, in line with the principles of designing a critical pedagogy.

Chapter 6 presents the findings from the empirical interviews undertaken with disadvantaged and vulnerable women. The sociodemographic details of the women are provided, followed by personal reflections on recruitment and the interview process. Six key triggers for birth trauma, focused on negative interpersonal interactions are presented and discussed, together with exemplar quotes, mapped to the wider literature.

Chapter 7 details how key findings from empirical stages informed the design of the immersive educational programme. This chapter includes a detailed description of the design and development of the script used in the filming of a 360 VR scenario. The key triggers for birth trauma are mapped against exemplar extracts from the script to depict how each particular trigger was expressed within the film. Visual stills and a synopsis from each key scene are detailed, followed by a table detailing each stage and activity of the programme, mapped to the critical pedagogical framework adopted within this study.

Chapter 8 presents evaluation findings following delivery of the immersive educational programme within an NHS Trust in the North West of England. This chapter details how the day was run, including a detailed discussion on each stage of the process. The evaluation findings are presented detailing how the programme impacted upon participants' knowledge, understanding and reflections of birth trauma and PTSD and their thoughts on the use of VR as a tool for critical reflection.

Chapter 9 presents the discussion and conclusions to this thesis. The study is situated within the current literature using a critical lens to illuminate key findings. The unique contributions generated by my study are detailed, together with recommendations for practice, policy and further research. The strengths and limitations of the research are also discussed. Conclusions are drawn on the impact of the programme, the use of critical pedagogies in maternity education, the potential of VR as a method for critical reflection and a tool for knowledge translation.

Chapter 10 presents insights into a collaboration with the University of Central Lancashire's business school that involved developing a campaign and marketing strategy for the immersive educational programme. This chapter details how academic outputs from the study were translated into tools for impact through a cross collaborative partnership.

Chapter 11 is a reflexivity chapter. Here I offer a reflection on the difficulties, issues and complications encountered on my journey to completing this thesis. Within this chapter I discuss how these have influenced my perceptions, thoughts and development as a researcher and as a human being.

CHAPTER 2: BACKGROUND CHAPTER

2.1 Introduction

This chapter aims to give an overview of the current literature concerned with birth trauma and the development of PTSD. Firstly, this chapter discusses the impact of birth trauma and PTSD, providing information on incidence, prevalence and impact upon childbearing women. Birth trauma and PTSD is then discussed amongst disadvantaged and vulnerable groups of women in maternity care. Risk factors and predictors of birth trauma are presented. A specific focus is placed on examining the current research concerned with interpersonal interactions between women and their health care professionals (HCP's) in maternity care. A focus on the social space of birth is then presented, including the impact of poor interpersonal interactions during birth. This includes examining how such interactions may impact upon the experience of birth for women. This chapter also explores current training provisions available to midwives who work with disadvantaged and vulnerable women, including current recommendations and key documents that have focused on enhancing maternity care for these women. In the final section I discuss the call for innovative solutions to address interpersonal care within healthcare. Digital technology is discussed within a midwifery context, exploring the possibilities of its use within midwifery education.

2.2 Birth trauma

Childbirth is reported to be a significant, liminal experience in a woman's life as she transitions from woman to mother. Research has identified how a positive birth experience can enhance a woman's self-efficacy, sense of achievement, capability and mastery (Broussard & Weber-Breaux, 1994, Lavender et al., 1999, Jomeen, 2010, Thomson & Schmied, 2017, Hill & Firth, 2018). A positive birth can also help foster emotional bonds with offspring, increase breastfeeding rates and reduce the risk of postnatal depression (Simkin 1991, Broussard & Weber-Breaux, 1994, Nichols & Gennaro, 2000, Nillson et al., 2013, Thomson & Schmied, 2017), highlighting the empowering capabilities of childbirth. However, some women have a traumatic and distressing experience of birth with short- and long-term implications for women, infant and their families (Ayers, Eagle & Waring, 2006, Beck & Watson, 2008, Jomeen, 2010, Reid, 2011, Fenech & Thomson, 2014).

A recent concept analysis undertaken by Greenfield, Jomeen & Glover (2016) provides a definition of birth trauma, acknowledging contrasting views on its aetiology and definition. Within this study, the definition of birth trauma was examined defined and re phrased as '*Traumatic Birth'*:

> *'Traumatic birth' is a complex concept which is used to describe a series of related experiences of and negative psychological responses to childbirth. Physical trauma in the form of injury to the baby or mother may be involved, but is not a necessary condition'* (Greenfield et al., 2016 p. 3).

This is the definition used within this current study, since, while birth trauma may or may not relate to physical complications, it is the psychological aspects of birth which are paramount and of interest within this thesis. As Beck (2004a) states:

> *'Birth trauma is in the eye of the beholder'* (Beck 2004a p. 32).

2.2.1 Impact of a traumatic birth

When childbirth is experienced as traumatic, it can have a profound negative impact upon on the lives of mothers, infants, fathers and wider families (Allen, 1998, Beck, 2004a, Beck 2004b, Ayers, Eagle & Wearing, 2006, Nicholls & Ayers, 2007). Undiagnosed and untreated, negative experiences of childbirth can affect women for years post birth (Forssen, 2012). Mothers who have experienced a traumatic birth can also go on to experience serious mental health problems (Forssen, 2012, Beck, 2004a), negative impacts upon marital relationships (Ayers Eagle & Wearing, 2016) and maternal infant bonding issues (Nicholls & Ayers, 2007, Renfrew et al., 2014). A recent meta-synthesis also highlighted how a traumatic birth can result in women experiencing devastating emotional turmoil with long-term negative repercussions on self-identity and relationships (Fenech & Thomson, 2014), highlighting the all-encompassing impact of birth experiences for women. Such an experience can also have implications upon women's future reproductive choices, with some women choosing a termination or sterilisation to avoid negative experiences associated with birth (Fenech & Thomson, 2014, Greenfield Jomeen & Glover, 2019).

One of the key implications of a traumatic birth is the risk for Post-Traumatic Stress Disorder (PTSD) onset following childbirth (Beck & Watson 2010, Pratt-Eriksson et al., 2014, Watson, 2016, Macias-Konstantopoulos, 2017)

Post-Traumatic Stress Disorder (PTSD) is defined as:

> *'An anxiety disorder associated with serious traumatic events and characterized by such symptoms as survivor guilt, reliving the trauma in dreams, numbness and lack of involvement with reality, or recurrent thoughts and images'* (PTSD. (n.d.) Collins English Dictionary – Complete and Unabridged, 12th Edition, 2014).

A diagnosis of PTSD is based on criteria defined by the American Psychiatric Association's Diagnostic and Statistical Manual (DSM) of Mental Disorders (American Psychiatric Association, 1980). The most recent DSM (version 5) defines this condition as an individual having been exposed to a traumatic event (directly or indirectly) when they feel their own or another's life is threatened, and who experience symptoms that cause significant distress from four different symptom clusters: Intrusion, avoidance, negative alterations in cognition and mood and alterations in arousal and reactivity (American Psychiatric Association, 1994). Symptoms of PTSD also need to be present for over a month period to qualify for a diagnosis. Within a midwifery context, childbirth poses as a particular risk for fear of life and the development of PTSD (Czarnocka et al., 2000, Olde et al., 2005, Grekin O'Hara et al., 2014).

There is also evidence highlighting that up to 75% of women who had PTSD after birth also had depression, resulting in the possible misdiagnosis of postpartum depression and an underrepresentation of cases (White et al., 2006, Parfitt & Ayers, 2008). Although postpartum depression has been extensively described (O'Hara & McCabe, 2013), data suggest that women following childbirth may also exhibit a post-traumatic stress response induced by the childbirth experience and may suffer from birth related PTSD without existing clinical depression (Olde et al., 2006, Grekin and O'Hara, 2014). These findings highlight the importance of understanding contributory factors for PTSD, alongside a recognition of its presentation so that maternity professionals can best support women presenting with symptoms.

Current maternity care practices also fail to account for the development of trauma, or the re-traumatization of women during childbirth despite trauma exposure increases the risk of a range of vulnerabilities (Herringa, 2017). These include mental health problems like PTSD alongside depression, excessive hostility, generalized anxiety, substance abuse, physical health problems, interpersonal struggles, eating disorders

and suicidality, among many others (Steencamp et al., 2015, Herringa, 2017). Within current practices, a lack of awareness, knowledge and screening tools tailored for childbearing women at risk of trauma related experiences, risks maternity services neglecting the needs of women who may be at risk of birth trauma (Ayers Eagle & Wearing, 2016, Balaam et al., 2017). Alongside a lack of understanding and pathways to prevent traumatizing women, maternity professionals remain unaware of the definitions, classifications and symptoms of PTSD in childbearing women (Patterson et al., 2018) presenting a gap in maternity education.

2.2.2 Incidence and prevalence of traumatic birth experiences & PTSD

Research evidence highlights that approximately 30 % of women experience childbirth as a traumatic event (Creedy Shochet & Horsfall 2000, Soet Brack & Dilorio 2003, Ayers et al., 2009, Alcorn et al., 2010) with up to one in three women presenting with symptoms associated with PTSD development (Creedy et al., 2000, Soet et al., 2003, Maggioni et al., 2006, Ayers et al., 2009, Grekin & Ohara, 2014). A recent meta-analysis reported that approximately 4% of women in community samples developed PTSD following childbirth (95%, CI 2.77–5.71) (Yildiz Ayers & Phillips, 2017). Women in high-risk groups (e.g. history of poor mental health, previous trauma, premature birth) were identified as at a greater risk of developing PTSD, with a mean prevalence of 18.95% (95%, CI 10.62–31.43) in pregnancy and 18.5% (95%, CI 10.6–30.38) after birth (Yildiz et al., 2017). At a diagnostic level PTSD profiles are also usually higher when measured soon after the birth. Unsurprisingly higher rates of PTSD are also found amongst women who have had late pregnancy loss or stillbirth (Engelhard et al., 2001, Turton et al., 2001). A study by Ayers and Pickering also found that prevalence rates decreased from 2.8% at six weeks to 1.5% at six months postpartum (Ayers & Pickering, 2001), highlighting potential discrepancies in the accurate reporting of women who experience birth trauma and PTSD.

Despite a breath of studies focused on measuring the prevalence of birth trauma and PTSD, the reporting of these vary considerably within the literature, depending on where studies have been undertaken. For instance, 2.1% of women were reported to have developed PTSD postpartum in a study undertaken in the Netherlands (Olde, Van Der Hart et al., 2006) compared to 17. 2% reported in a study undertaken in Iran (Shaban et al., 2013). However, it is important to note that variations in rates may be attributed to

time at diagnosis, measurement tools used, level of maternity care provided, and population studied. These statistics are also likely to be an under representation as women may feel unable to seek help due to feeling stigmatized, culture differences, concerns over negative reprisals and a mistrust of professionals (Dixon–Woods et al., 2005, Marryat & Martin., 2011, De Schepper et al., 2016). It has also been noted that confusion and uncertainty exist amongst maternity professionals on how to identify and separate risk, or presence, of PTSD, postpartum depression, anxiety and hormonal changes when caring for women (Bromley et al., 2017). This results in many women who may be suffering from a traumatic birth and PTSD remaining undetected and not supported, often presenting later with issues related to previous experiences (De Schepper et al., 2016). While beyond the remit of my study, this research highlights the need for standardised practices in measuring the incidence of birth trauma and PTSD to gather more representative and accurate statistics on incidence and prevalence to best support women at risk.

2.2.3 Risks & predictors of traumatic birth experiences & PTSD

Two meta-analyses (Grekin & Ohara, 2014, Ayers et al., 2016) report pre-birth risk factors for PTSD onset following childbirth as pre-existing depression, fear of birth, poor health and risk of complications, a history of PTSD and a need for counselling in pregnancy. Specific intrapartum related risk factors were identified as: operative birth, negative experiences of childbirth and lack of support and reported experiences of disassociation[1] (Van Son et al., 2005). A recent meta-analysis identified five categories of risk factors, these included negative perception of childbirth, maternal mental health, trauma history, PTSD, delivery mode and complications and low social support (Dekel Stuebe & Dishy, 2017). Negative perceptions of childbirth and low social support received the highest weighted score and were identified as the most potent predictors of PTSD (Dekel et al., 2017), raising questions about the impact adverse childhood experiences[2] and complex life factors that may place women at a higher risk for developing PTSD.

[1] Dissociation is a psychology term used to describe a wide array of experiences from mild detachment from immediate surroundings to more severe detachment from physical and emotional experiences. The major characteristic of all dissociative phenomena involves a detachment from reality.

[2] Adverse Childhood Experiences (ACEs) are stressful events occurring in childhood. These include, but are not exhaustive; domestic violence, parental abandonment through separation or divorce, being the victim of abuse (physical, sexual and/or emotional), a

According to Ayers outcomes and incidences of birth trauma and PTSD for women depend on the interplay between pre-birth vulnerability factors, risk factors in birth, and factors after birth (Ayers et al., 2016). Factors such as pre-existing maternal psychiatric problems, a previous negative birth experience and a lack of social support, have been consistently reported as risk factors for birth trauma and PTSD (Andersen et al., 2012), similar to factors associated with PTSD development in different types of trauma (Ozer et al., 2003). Other research has also identified how a lack of understanding of the individual needs of women, loss of trust in staff providing care and women-professional communication barriers all contribute to a self-perceived traumatic birth (Jayaweera & Quigley 2010, Redshaw & Heikkila 2010, Women's Health and Quality Consortium, 2013, Garthus-Niegel et al., 2013, Psarros, 2014). A lack of effective care and poor support and advocacy between women and their health care providers have also been noted as key issues for women who report negative experiences during childbirth (Harris & Ayers 2012). A qualitative meta-synthesis by Elmir et al (2010) exploring women's experience of birth trauma highlighted disrespectful care practices and women experiencing a loss of control as the main contributory factors for birth trauma.

These findings illuminate the importance of possible stressors associated with the risk for PTSD and the role maternity professionals play in contributing to women experiencing a traumatic birth. Thomson & Feeley (2019) reinforce this notion, noting that when trust-based relationships are absent during birth (either as a result of care provided by multiple care providers and/or when care does not align with the woman's needs) this can destroy a woman's' feelings of safety during labour and childbirth, ultimately increasing negative emotions. Whilst risk and predictive factors are important to identify women who may be more likely to develop PTSD, other research has highlighted how subjective experiences of childbirth are most important (Garthus-Niegel et al., 2013). A study by Thomson & Downe (2010) highlighted how negative or positive birth experiences were not associated with a particular type of birth, or model of care, emphasising it's not *what* happens in childbirth but *how* it happens that is

member of the household being in prison, being the victim of neglect (physical and emotional), a parent with a mental health condition or growing up in a household in which there are adults experiencing alcohol and drug use problems.

important. The need to address relationship-based care as a means to prevent trauma and PTSD onset for women in maternity care is evident and requires urgent attention.

2.2.4 Theoretical basis for PTSD

Several theoretical models/frameworks have been developed to help understand the onset of PTSD. A recent study by Dikmen-Yildiz Ayers and Phillips (2018) focused on long term trajectories of PTSD after birth and associated risk factors using the diathesis stress model[3], helping identify women at risk of PTSD. This model identified that poor satisfaction with health professionals was associated with chronic-PTSD and delayed-PTSD, with less social support and negative accounts of childbirth 4–6 weeks after birth predictors for chronic recovery trajectories (Dikmen-Yildiz et al., 2018). The diathesis stress model also considered that negative responses (such as those associated with PTSD) occur due to a combination of an individual's vulnerability and when the level of stress experienced exceeds a certain threshold held by an individual (Dikmen-Yildiz et al., 2018). Janoff-Bulman (2010) suggests that people generally hold beliefs and assumptions about themselves and their life-world: beliefs that they will not be subject to adversity and that the world is '*meaningful*' and '*benevolent*'. When these beliefs are challenged or '*shattered*' through a traumatic experience (e.g. a traumatic birth) this then leads to fundamental and negative alterations of self-image and interactions with others. Ehlers & Clark (2000) developed a cognitive model that suggests PTSD onset occurs when a traumatic event is experienced in such a way that is presents a sense of current threat, with this sense of threat linked to an individual's level of vulnerability, including beliefs, prior experiences, coping abilities, the negative assessment of the traumatic event and possible disturbed memory processes.

These theoretical standpoints help in understanding how the onset of PTSD may occur, highlighting the importance of identifying existing levels of vulnerability women may have prior to childbirth and how these may impact upon their risk of experiencing trauma and developing PTSD.

[3] The diathesis–stress model is a psychological theory that attempts to explain a disorder, or its trajectory, as the result of an interaction between a predisposition of vulnerability and a stress caused by life experiences.

2.3 Disadvantaged and vulnerable women in maternity care

Several studies have identified socio-demographic risk factors for PTSD. These include young age, low income, primiparity, multiparity, adverse childhood experiences and mental health problems (Zambaldi et al., 2011, Abedian et al., 2013, Boorman et al., 2014, Vossbeck-Elsebusch et al., 2014, Yildiz et al., 2017). Terms such as disadvantaged, vulnerable and/or marginalized are often used interchangeably when describing people who are excluded from social, economic and/or educational opportunities enjoyed by others in their community due to numerous factors beyond their control. These include factors at the social level (such as economic inequality, violence, stigma, racism, and migration), family level (including neglect and abuse) and individual level (e.g. disability, minority ethnicity) (The World Health Organization WHO, 2017). Disadvantaged and vulnerable groups include women who are immigrants or refugees; sexual minorities; those living in poverty and the socioeconomically deprived; incarcerated; those who have run away or been turned out of their homes following neglect and/or abuse; those who are trafficked and those who belong to a stigmatized indigenous, minority ethnic, tribal or religious group (WHO, 2007).

Recent studies also reveal that teenage mothers, those from socioeconomically disadvantaged areas and Black, Asian and Minority Ethnic (BAME) communities are more likely to have mental health issues, have a higher mortality rate and have a higher rate of long term conditions associated with lifestyle (Office for National Statistics, 2011, Department of Health 2012a, 2012b, Public Health England, 2014), highlighting the range of vulnerabilities amongst these groups of women. As these population groups have been identified as suffering the worst health outcomes in maternity care, alongside being at an increased risk of experiencing birth trauma and PTSD (CEMACH, 2008, Marmot, 2010, Knight 2019), it was decided that this study would focused on exploring birth trauma experiences amongst this group of women. Firstly, it was important that key classifications of disadvantaged and vulnerable were identified for this study.

When exploring terms within the literature, questions regarding what constitutes a classification of disadvantaged or vulnerable has led to debate within research due to different interpretations of each concept. For example, some authors argue for a broader approach to classifications of vulnerable, stating that every research protocol that focuses on participant vulernablilites needs to be assessed on its different features

or layers, emphasising context specific considerations that may impact upon researchers' interpretations (Luna., 2009, Lange et al., 2013). Such interpretations range from researchers own experiences, demographics of participants and/or the location of research endevours, to the historical and political influences upon research contexts impacting upon ones understanding and analysis of a particular phenomena (ref xxx). A recent concept analysis provides an example of such an approach, clarifying how the term vulnerability is defined in relation to pregnancy, birth and the postnatal period, concluding that vulnerability should be viewed as a complex phenomenon rather than a singular concept when applied to childbearing women (Briscoe, Lavender, McGowan 2016). Van der Zande (2017) explored this concept further during the chiddbearing continuum, categorising features of vulnerability during pregnancy which included Informed consent, susceptibility to coercion, higher exposure to risk due to a lack of scientific knowledge and vulnerability of the fetus. Alongside vulnerabilities relating to care delivery, research has also highlighted that individual vulnerabilities amongst disadvantaged and vulnerable women such as mental health difficulties, domestic violence and self-harm are increased during pregnancy (Burch & Gallup, 2004, National Institute for Health and Clinical Excellence NICE., 2010, Higgins et al., 2016, Orsolini et al., 2016). In light of such complex situations that women may find themselves in during pregnancy, it was important to ensure that women who self-defined as vulnerable were represented within the meta ethnography. It was decided that abstracts and papers would also be screened for women who fell into this category to ensure representation.

When developing a comprehensive meta ethnography exploring definitions of disadvantaged and vulnerable provided by key organizations concerned with the health and wellbeing of women provided a logical starting point. These key organizations included the World Health Organization, the United nations Population fund, The White Ribbon Alliance, The International Federation of Gynaecology and Obstetrics, The National Institute for Health Research. Alongside searching organisation definitions, numerous midwifery-based research studies focused on disadvantaged and vulnerable women were also searched (Grote et al., 2015, Rayment-Jones et al., 2015). Key documents identified within the background chapter of the thesis were also examined to identify how authors themselves defined disadvantaged and vulnerable within their studies. Through these stages I was able to compile a thorough and comprehensive list

regarding the classifications of *'disadvantaged'* and *'vulnerable'* amongst childbearing women. The most common and most cited within the literature were complied and listed as seen in table 1, as to provide the review with a comprehensive list during screening.

Table 1 Classification of disadvantaged and vulnerable characteristics amongst childbearing women

Teenage mothers
Asylum seeking women
Victims/survivors of domestic abuse/sexual abuse
Substance (drugs & alcohol) abusers
Living in poverty/extreme financial hardship
Excluded from education
Specific ethnic minority groups
Travellers
Poor mental health
Homeless / Living in temporary accommodation
Learning disabilities / physical disabilities
Known to child protection services
LBGTQ women
Women living in deprived areas
Women in prison
Recently arrived migrants
Refugee women
Women that self-define as vulnerable

2.3.1 Health inequalities amongst disadvantaged and vulnerable women

Despite government initiatives and reports focused on improving outcomes and access to care for disadvantaged and vulnerable groups (Marmot 2005, Department of Health 2006, Department of Health 2012b, Marmot & Allen, 2014) health inequalities amongst these groups still persist in the NHS (Cookson et al., 2017, Donkin & Marmot, 2017, Mattheys et al., 2018, Bhui et al., 2018). Alongside these facts, disadvantaged and vulnerable women are more likely to have poor access to healthcare due to issues such as mistrust of professionals (Dixon–Woods et al, 2005., De Schepper et al., 2006, Marryat & Martin, 2010), social stressors such as lack of support and complex relationships (Kramer et al., 2000, Mackenbach et al., 2008, Knight et al., 2009), communication barriers (Raine et al, 2010) and fear of stigma and judgements (Jakobsen & Overgaard, 2018). BAME women and those from disadvantaged and vulnerable backgrounds also have a higher risk of maternal mortality (Drapper et al., 2018),

highlighting complex and interwoven biopsychosocial[4] influences amongst these groups of women. These facts highlight the need for services to provide specialised and individualized care for vulnerable groups in order to improve outcomes and reduce inequalities in care provisions.

2.4 Health inequalities in midwifery care: A global perspective

Women's experiences of poor maternity care have been identified globally, including in the UK (Hodnett, 2002, Feder et al., 2006, Bohren et al., 2016). Browser and Hill (2010) conducted a landscape report in which they described seven major contributors of disrespectful and abusive practices in maternity services, including individual and community, national law and policies, human rights and ethics, governance and leadership and service delivery and providers. Bohren et al (2016) expanded upon this report by conducting a mixed method review to highlight issues across high, middle and low-income countries, reporting that disadvantaged and vulnerable women were at a higher risk than more privileged women for negative experiences of intrapartum care. This raises important questions about inequalities within maternity provisions that may be contributing to traumatic birth experiences and PTSD.

Within their study, Bohren and colleagues highlighted specific issues relating to care delivery, including physical abuse, non-consented clinical care, non-confidential care, non-dignified care, discrimination, abandonment and sociodemographic influences (Bohren et al., 2016). A systematic review of women's satisfaction with childbirth was undertaken by Hodnett (2002) concluding that women's expectations of caregiver support generally overrides socio-demographic influences that may contribute to negative experiences, placing a greater emphasis on the interpersonal impact of care delivery over that of sociodemographic factors. Similarly, the impact of sociodemographic influences should not be separated from the analyses of negative birth experiences, but placed in context, allowing researchers to take a whole systems approach, accounting for the biopsychosocial influences upon women's experiences of care and subsequent associated mental health issues (Buultjens et al., 2013). Bohren et al (2016) also identified that while disrespect and abuse in maternity care was a

[4] Biopsychosocial refers to the interconnection between biology, psychology, and socio-environmental factors. Examining how these aspects play a role in health and disease.

universal phenomenon, difficulties arise when addressing traumatic experiences of care amongst diverse groups due to structural, cultural and variations in maternity provisions across low- and middle-income countries.

As part of the recent Lancet Midwifery series, Horton & Astudillo (2014) called for a '*system level shift'*, highlighting the variations of midwifery provisions globally that impact upon women's experiences of maternity care. Renfrew et al (2014) urge stakeholders to acknowledge the political, influential and social pressures upon maternity services globally to address inadequate care provisions for women and babies, furthermore, Freedman & Cruk (2014) call for quality and accountability agendas within maternity care to be challenged, using the issue of disrespectful and abusive care practices in these settings as an example of failing systems. Although these findings highlight the role interpersonal care may play in the experience of disrespectful and abusive care practices, these must also be placed in context with political, social and structural aspects of care delivery.

2.5 Neoliberalism and social determinants of health amongst childbearing women

Political influences on healthcare services has long been an area of contention, especially within the UK and the delivery of a publicly funded NHS. Sturgeon (2014) highlights the issues faced in the UK due to the rise of a neoliberal consumer culture and the commodification in the provision of healthcare. Neoliberalism is an ideology and political model that emphasizes the value of free market competition (Venugopal, 2015). Although a political theory, its application has directly impacted upon social determents of health by promoting a society that is based on competition and meritocracy[5] (Littler, 2017). The neoliberal agenda became influential in the UK in the 70's during the prime ministership of Margaret Thatcher, when the term '*Thatcherism*'[6] was born. This approach to political reform welcomed a free market economy, deregulation and privatization. This reform was seen to be the answer to social justice and equality by driving competition and a society built on merit and a small state mind

[5]Meritocracy: A political system in which economic goods and/or political power are vested in individual people on the basis of talent, effort, and achievement, rather than factors such as heredity or wealth.

[6] Thatcherism has been used to describe the principles of the British government under Margaret Thatcher. Thatcherism represented a systematic, decisive rejection and reversal of the post-war consensus instead favouring the free market and privatisations of state funded industries.

set (Fuchs, 2016). It rejects the idea that people's future is determined by their socioeconomic positioning believing that through hard work, belief in their abilities and accessing all the support available, everyone can improve their social position seamlessly (Labonté & Stuckler, 2016). Unfortunately, however, this ideology ignores the impact of poverty, hunger, ethnicity and gender on many, with theorists argue that this approach has fuelled a growing inequality gap in the UK from the 70's onwards (Scott-Samuel et al., 2014).

Neoliberalism's impact upon healthcare began with Thatcher introducing several policy initiatives that set the NHS on a course from which it has not deviated since (Scott-Samuel et al., 2014). These changes led the NHS into a form of managerialism and the introduction of a quasi-market in healthcare centred on competition and choice, resulting is disparities in healthcare delivery across the UK. Many also argue that national and international neoliberalism as an ideology has played a role in many of the crises that have taken place in the last 20 years, including the financial collapse of 2008[7] (Kotz, 2009). From a health perspective it is also argued that neoliberalism has contributed towards the collapse of public health, a resurgent in child poverty, a rise in homelessness, loneliness, depression and chronic mental health issues (Labonté & Stuckler, 2016). Mercer and Flynn (2017) believe that neoliberalism has played a part in the deregulation and consumer approach adopted within the NHS, resulting in widening inequalities for those accessing care and inappropriate provisions of care for those with complex needs. I argue within this thesis that these significant changes should not be viewed as merely a political issues, but acknowledged as arising from the same ideology (neoliberalism) which has now become so engrained within daily life, that many are unaware of its influence, existence and impact upon a service that is use by most women in the UK at some point in their lives.

Some would argue that the current political approaches to managing the healthcare service in terms of efficiency and improving health outcomes, tends to undermine local social determents of health (Maynard & Williams, 2018, Barker et al., 2018). With others refuting this belief stating that austerity measures are vital in rebuilding and

[7] The financial crisis of 2007–2008 is also known as the global financial crisis and the 2008 financial crisis. It is considered amongst economists to have been the most serious financial crisis since the Great Depression of the 1930s.

strengthening healthcare service in the NHS, including the privatization of some services across the country (Gamble, 2014). Russell and colleagues have shown how the postcode lottery of healthcare across the UK is resulting in fragmented services that are unable to cope with the demands of an increasingly diverse society in terms of health, wealth and opportunities (Russell et al., 2013), further exacerbating the difficulties faced by disadvantaged and vulnerable groups needing to access healthcare in the UK.

2.6 The impact of austerity upon provisions of maternity care

Klein explains how neoliberal theorists have historically advocated the use of crises to impose unpopular government policies (Klien, 2015). This is relatable to the current climate in which cuts to NHS funding, privatisation of essential services, liquidation of Sure Start centres[8], removal of specialists' posts supporting vulnerable women, and a lack of funding invested in perinatal mental health services are widespread across the UK (Robertson et al., 2017). Roberts argues that governments consistently insist that austerity cuts to healthcare services in the UK will improve overall quality, yet whilst the nation is led to believe that these approaches are necessary, corporations and governments are working in their own best interest as they steadily deregulate and privatise services for corporate gain (Roberts et al., 2012). Austerity approaches to improving healthcare are regularly reinforced through the media, citing topical issues of health tourism, alcohol abuse, drug related incidents, teenage pregnancy and the benefits system as placing unnecessary strain upon the NHS's ability to provide quality care (Lewis et al., 2018).This approach reinforces a merit-based rhetoric, in which issues such as underfunded healthcare services and socioeconomic influences on health and poverty are replaced by tales of patients misusing vital services with self-perpetuating conditions such as drug and alcohol abuse (Meier, 2016).

Neoliberalism disregards the human endeavour of the NHS, which is inherently a political act of social justice (Vetter, 2003). Unfortunately, neoliberal narratives have

[8] 'Sure Start' was an area-based initiative introduced in 1998 by the government. The initiative aimed to give children the best possible start in life through the improvement of childcare, early education, health and family support, with an emphasis on outreach and community development. Chancellor Gordon Brown announced that the Government would provide funding for 2,500 Children's Centres by 2008. In 2017 the evidence concerning the effectiveness of Sure Start from both the NESS and the ECCE studies was summarised by a briefing paper that was written for members of Parliament. The value-for-money analysis concluded that most services provided a net financial loss to Government. Research published in April 2018 reveals that as many as 1,000 Sure Sure Start children's centres in England may have closed since August 2009, with local authorities cited financial pressures as a reason for cuts (Puffett, 2018).

created a discontent and tension within the UK healthcare system, impacting upon belief and attitudes that dominate social spaces in healthcare (Abassi, 2018). Mold (2015) argues that these beliefs have created divisions in British society, impacting upon perceptions of entitlement towards those who access care with complex needs. These issues could be seen to further exacerbate complexities for disadvantaged and vulnerable women in maternity care, impacting upon how they interact, access, engage and experience maternity services. It is acknowledged however that all may share this particular take on the crisis within the NHS, although for the purpose of this study, this viewpoint provides an inevitable backdrop for investigating the wider social and political influences that impact upon women's care experiences.

2.7 Othering in maternity care

While maternity care systems have long acknowledged the diversities of those assessing maternity care, a deeper layer of inequality exists that impacts upon the segregation of certain groups, through a process that sociologists have termed *'Othering'*[9]. Othering is the process of labelling an individual or a group as the other, founding one's own identity through opposition and a criticism of this *'other'* (Canales, 2000). Mead established that social identities are created through social interaction with other people and our consequent self-reflection about who we think we are, according to these social exchanges (Mead, 1934). The notion of *'otherness'* is also seen as central to the way a society establishes an identity (Bauman, 2013). It must also be acknowledged, however, that otherness is not exclusive to maternity care and needs to be understood in the context of how certain groups/people are viewed as different, or, not fitting into predefined boxes of what constitutes normal within a society (Eriksson, 2015). In the context of this study, women with existing mental health conditions, those dealing with complex life situations and minority ethnic women are at risk of being othered in maternity care due to falling outside of the realms of a *'normal'* childbearing state (Thomson & Schmied, 2017), creating a possible crisis of inequality.

Although maternity services acknowledge that disadvantaged and vulnerable women require specialised support during pregnancy, provisions to support these women are

[9] The idea of '*Othering*' is central to sociological analyses of how majority and minority identities are constructed. This is because the representation of different groups within any given society is controlled by groups that have greater political power.

consistently being cut, leaving women at risk of poor mental health, maternal mortality and traumatic experiences of care (Draper et al., 2018, Firth & Haith Cooper, 2018). Acknowledging the diversity of those engaging with maternity services is a vital step in improving outcomes and experiences, although this requires an acknowledgement of the beliefs, criticisms and ideas held about the *'other'* by maternity professionals in the NHS.

2.7.1 Intersectionality in a midwifery context

Intersectionality factors may also impact upon maternity care experiences amongst disadvantaged and vulnerable women. Intersectionality as a concept considers that various forms of social stratification, such as class, race, sexual orientation, age, religion, creed, disability and gender do not exist as separate identities, but are interwoven together (Collins & Bilge, 2016). The awareness of intersectionality factors encourages researchers to be mindful of the role *'structure'* (recurrent patterned arrangements which influence or limit the choices and opportunities available) and *'agency'* (the capacity of individuals to act independently and to make their own free choices) play in influencing the deterministic limits of women's ability to act with agency as a result of interwoven social strata's (Weber & Parra-Medin, 2003). As disadvantaged and vulnerable women are at a greater risk of having past trauma related experiences, there is an increased risk that these women may experience interactions with maternity care professionals as threatening, hostile and unsupportive due to a lack of resilience and agency during birth (Thomson & Schmied, 2017).

A number of researchers have found that service users from vulnerable population groups continue to say that maternity services in the UK do not meet their needs (Thomson et al., 2013, McLeish & Redshaw, 2019), further exacerbating dissatisfaction with care experiences for these women. To prevent birth trauma experiences, some research suggests that amongst these population of women, resilience could play a protective role against birth trauma (Yildiz et al., 2017), although focusing on resilience as a protective factors attributes blame and responsibility on the individual, whilst minimising and devaluing the role that others may play in contributing to this experience.

2.8 The physical and psychological impact of poor interpersonal care.

Lived human relations are concerned with relationships, communications and connections we make with others during life events (Van Manen, 2016). Whilst these relations are ever-present in our lives, childbirth presents a potentially isolating, vulnerable experience in which relationships with others in the social space of birth are crucial to positive experiences (Thomson & Downe, 2010). A Cochrane Review undertaken in 2015 concluded that women require improved emotional support during birth from their care providers to reduce the risk of trauma (Bastos et al., 2015). A study by Thomson and Downe (2008) identified that birth trauma was related to fractured interpersonal relationships with caregivers, with women feeling disconnected, helpless and isolated during birth. Following a mixed methods study of women's experiences of childbirth trauma in relation to care provider interactions, Reed et al (2017) suggests that maternity service provision needs to prioritise the physical *and* emotional needs of women to improve experiences of care, as whilst midwives may be focused on the processing of birth, and clinical indicators, the subjective experience of women must be considered.

2.8.1. Iatrogenic impact of birth

Research over several years has also identified the link between maternal distress and iatrogenic harm. These studies report the links between maternal distress and higher levels of cortisol during the childbearing continuum, placing the mother and baby at increased risk of premature birth, immunosuppression of the neonate, fetal neurodevelopment and obstetric intervention (Stewart et al., 2015, Su, Zhang et al., 2015). Heightened levels of stress such as that experienced during a negative and traumatic birth could contribute to iatrogenic harm (Dahlen et al., 2013), compounded amongst disadvantaged and vulnerable women due to existing social and psychological stressors (Schetter & Tannner, 2012). With other research identifying that some women may experience interpersonal trauma that stems from the negative attitudes of health care professionals during birth (Hodnett, 2002, Bohren et al., 2016, Yildiz et al., 2017). Reducing maternal stress through positive experiences of care could help reduce the risk factors associated with infant mortality, unnecessary interventions and traumatic birth experiences.

2.9 Specialist care for disadvantaged and vulnerable women

The recent Maternity Mental Health Alliance MMHA *'Everyone's Business Campaign'* highlighted shocking gaps in UK perinatal mental health services, with the costs of PMH exceeding eight billion per one-year birth cohort (Bauer et al., 2014). The Due North Study also highlights a widening inequality gap between the North of England and the rest of the UK (Department of Health DOH, 2014), with the MMHA mapping exercise of perinatal mental health services revealing how the most deprived areas in the UK have notably less spent on mental health services for women (MIND, 2014). This mapping exercise also revealed that PMH services in North West UK (an area with a high socioeconomically deprived communities) fall short of national standards.

An increased awareness of the impact of poor maternal mental health and improved financial investment in the area has led to the rapid expansion of perinatal mental health provision across the UK (NHS England, 2017). In 2015, an additional £375 million funding was announced to align national perinatal services with agreed standards (Maternal Mental Health Alliance, 2016, Mental Health Taskforce, 2016), with perinatal mental health investment an identified area of growth and is expected to expand by 250% in the fields of nursing and midwifery by 2021 (Health Education England HEE, 2017). To date, some NHS Trusts have invested in specialised services for vulnerable women, yet barriers to accessing these services include multiple factors such as lack of awareness about mental ill health, cultural expectations, ongoing stigma, culturally insensitive and fragmented health services and interactions with culturally incompetent and dismissive health providers (Baldwin et al., 2018, Watson et al., 2019). Despite Thomson & Balaam (2018) highlighting that support provided by a specialist perinatal midwifery service / team improved the maternity and parenting experiences of vulnerable/marginalised women, authors argue how more work needs to be done to reduce unwarranted variation and inequity in specialist mental health services in maternity care provision across the UK (Baron et al., 2016). Evidence also highlights how current services are unable to cope with the increasing number of pregnant women seeking help with issues such as depression, anxiety, suicidal intents and PTSD (The Royal College of Obstetricians and Gynaecologists (RCOG), 2017), the need for preventative interventions focused on perinatal mental illness is timely.

A recent meta synthesis undertaken by Smith et al (2019) identified that barriers to accessing mental health services amongst women with perinatal mental illness involve complex, interlinking, multilevel barriers, highlighting the need for stakeholders to address individual, organisational, sociocultural and structural-level barriers at different stages of the care pathway. In May 2018 NHS England, NHS Improvement, National Collaborating Centre for Mental Health (2018) collaborated to produce guidance on perinatal mental health pathways for practitioners in the primary and secondary care The guidance highlights significant opportunities to deliver better value, evidence-based perinatal mental health care, supported by an investment of £365 million between 2015/16 and 2020/21. However, a recent evaluation on current UK perinatal community mental health services found that there is considerable variability within the provision of services offered across the UK, reflecting differences in service user needs, in local mental health and statutory services provision, in staff skills and training, and in the developmental stage and staffing provision of the team (Davies et al., 2018). This is mirrored in the lack of acute care services to support vulnerable pregnant women in the NHS (NHS England, 2016), highlighting gaps in both acute and primary care settings.

Currently, investment in perinatal mental health resides predominantly in treatment and interventions for women, opposed to preventative measures and education. A recent integrative review focused on barriers to addressing mental health issues in a midwifery setting identified a lack of training, lack of clarity regarding the scope of practice and time constraints as common provider level barriers to addressing mental health issues from identification to management (Bayrampour et al., 2018), with maternity staff report feeling unequipped to deal with mental health issues amongst complex and diverse minority groups (Ross-Davie et al., 2006). Staff have also expressed feeling unsure of how to broach sensitive subjects, and how at times they avoid raising these issues for fear of causing offence or due to feeling ill-equipped to help if problems are disclosed (Somerville, 2015, Lindenmeyer et al., 2016, Cole 2017). These issues could also contribute to health professionals feeling a lack of satisfaction in their roles due to being unable to deliver care to meet women's psychosocial needs (Byrom & Downe 2015). Findings highlight the need for specific training aimed at supporting maternity professionals in caring for women with complex needs to support both women and

healthcare providers and a potential preventative approach to addressing issues such as birth trauma and PTSD development amongst childbearing women.

2.10 Current training on birth trauma and complex issues

To date there is no mandatory training delivered in the UK focused on birth trauma and PTSD for maternity professionals. Evidence shows that the quality and quantity of mental health focused education currently received by maternity care professionals is failing women (Ross et al., 2006, Rowan et al., 2010, Saving Mothers Lives, 2011). While the MMHA highlights the need for better health care provision for mental health in maternity services (Bauer et al, 2014), others have argued that this needs to be approached collaboratively with maternity staff, including a specific focus on education (Beck, 2004b., Downe Finlayson & Flemming, 2010, Grekin & O'Hara, 2014). The recent Maternity Service Review also highlighted this need, calling for training and sharing of best practice to reduce variation and to standardise care delivery for disadvantaged and vulnerable women across the country (NHS England, 2016). Recommendations from this includes a report on education delivery in maternity services suggesting that a scaling up of midwifery education across disciplines in health could enhance working relationships, improve staff confidence and support those accessing care with complex needs (NHS England, 2016). The Birth Trauma Association (BTA) the MMHA and Thomson and Schmied call for research into maternity staff training to assess education delivery, identify and address contributory factors to birth trauma and implement changes to help bridge the inequality gap (Bauer et al., 2014, Thomas & Schmied 2017, The Birth Trauma Association 2018). Currently, there appears to be no clear evidence or guidance for policy makers into the level, content or length of training required. An approach that is gaining ground in a mental ill-health preventative context is trauma informed care (Muskett, 2014), offering a framework within which maternity services can adopt preventative measures against traumatic birth experiences and PTSD.

Harris & Ayers (2012) agree calling for better educational resources to reduce the number of women experiencing trauma, alongside supporting midwives in recognising and supporting women at risk. Unfortunately, staff shortages, inadequate funding and a lack of professional training opportunities hinder post qualifying educational opportunities for staff (Jarosova et al., 2016). Given the variations and lack of effective resources for maternity staff caring for women with complex needs, the need for

adequate support and enhanced training focused on birth trauma and PTSD is pressing (Ayers et al, 2016).

2. 11 Addressing cultural and organisational failings impacting upon maternity care

The Kings Fund identified that core problems related to failings in the NHS are a result of organisational cultures and processes (West, 2016). This report proposed that when focusing on potential solutions, organisations must focus on logistical, strategic and organisational causes rather than focusing on specific professional failings, highlighting how the latter may result in disregard and inaction around managerial and structural deficiencies that impact upon care delivery (West, 2016). In contrast, other research stipulates that a greater focus should be placed on reconnecting midwives to the innate and intuitive skills and aspects of maternity care by returning to a humanistic framework for care delivery (Ménage, 2017, Pearson, 2018). Midwifery staff are continuously faced with a contradiction between expected high levels of intuition and empathy within their caring role and pressures upon them in practice due to increasing workloads. Despite these issues, NHS midwives in the UK currently receive little to no formal post qualifying education focused on compassionate, culturally contextual or humanistic care delivery (Good & Officer, 2012, Hall, 2013, Bray et al., 2014). The ability of midwives to pay close attention to women, practice empathetically, and take intelligent action to help is known to be affected by high and chronic levels of stress (Byrom & Downe 2015, Wright et al., 2018). These issues highlight the difficulties faced when attempting to change practices without an appreciation for the complex and multifactorial processes within healthcare institutions.

Recent reports highlighting failings in the NHS (Francis, 2013, Kirkup, 2015) continually touching upon the instinctual essence of caring, calling for staff to reconnect with core values in their roles. Dealing with women with complex needs in maternity care calls for a higher level of emotional intuitiveness and awareness to adapt, understand and deliver care that meets the needs of an individual (Hyde & Roche-Reid 2014, Power, 2016). It has also been reported that, in maternity care, the neoliberal managerialist imperative of a *'one size fits all'* approach can result in women feeling processed, dehumanised and under-supported placing them at risk of birth trauma and subsequent PTSD (Thomson & Schmied 2017). This situation is also compounded by a lack of dedicated support services for disadvantaged and vulnerable women and uncertainties

amongst maternity professionals regarding contributory factors, recognition, treatment, and impact of birth trauma PTSD amongst these groups of women. The Birth Trauma Association (BTA) the MMHA (Bauer et al., 2014) and Thomson & Schmid (2017) all call for research into maternity staff training to assess education delivery, identify and address contributory factors to birth trauma and implement changes to help bridge the inequality gap. Currently, however, there appears to be no clear evidence or guidance for policy makers into the level, content or length of training required. An approach that is gaining ground in a mental ill-health preventative context is trauma informed care (Muskett, 2014) offering a framework within which maternity Trusts can adopt preventative measures against traumatic birth experiences and PTSD.

2. 12 Trauma informed approaches to maternity services

Trauma informed care is an approach to healthcare delivery for people with complex needs and is defined as:

> *'A strengths-based framework that is grounded in an understanding of and responsiveness to the impact of trauma, that emphasizes physical, psychological, and emotional safety for both providers and survivors, and that creates opportunities for survivors to rebuild a sense of control and empowerment'* (Hopper et al., 2010 p. 56).

It is a structured treatment framework that involves understanding, recognising, and responding to the effects of trauma (Guarino, 2015). The practice is guided by six principles: safety; trustworthiness and transparency; peer support; collaboration and mutuality; empowerment, voice and choice and cultural, historical and gender issues (Bowen & Murshied, 2016). Suggestions on implementing trauma informed approaches in maternity care include adopting a tiered or stepped approach recognising that supportive interventions that decrease one or more stressors for women could result in better outcomes (Seng & Taylor, 2015). A trauma informed approach is also associated with decreased stress levels and improved social, psychological and physical wellbeing (Muskett, 2014). It is also acknowledged that the intersectionality of trauma, PTSD, mental health issues and complex life factors that play a part in their development amongst childbearing women is complex and difficult to treat (Ayers et al., 2016). Rather than attempting to identify and design interventions that specialise in the treatment of

birth trauma and PTSD, an intervention focused on prevention could provide a practical approach to reducing the numbers of women suffering. A trauma informed approach to the design of maternity education could provide maternity services with a sensitive approach to addressing these complex factors involved in the aetiology of birth trauma and PTSD.

Attempting to address risks faced by childbearing women first requires an informed approach to identifying existing risk factors and vulnerabilities. Statistics show that one in five women have a history of childhood mistreatment, one in five have been subjected to severe violence in intimate relationships and one in five have suffered from sexual assault (WHO, 2016), all risk factors for the development of trauma and PTSD. These facts shed light on the need for maternity services to update their schema for maternal mental health and how services are provided. Trauma informed approaches emphasize physical and psychological safety and help survivors rebuild a sense of control and empowerment (Muskett, 2014). The development and monitoring of trauma-informed interventions and policies was found to provide a counterbalance to social and environmental risk factors for trauma for disadvantaged obese pregnant women in a recent study (Tuck et al., 2017).

Beckett et al (2017) suggests that delivering care through a trauma-informed lens leads to increased patient satisfaction with care and greater professional satisfaction for those delivering care. Trauma-informed approaches to designing and delivering services could provide maternity care providers with an approach to empowering women that can help them to maintain sense of trust and control in the birth environment. First this requires an acknowledgement of the importance of women's voices and experiences, to ensure that any new solutions are relevant to all, and co-produced with those they are designed to support.

2.13 A co-productive approach to service design: listening to women's voices

The recent NHS Maternity Service Review (NHS England, 2016) placed emphasis on a co productive approach to quality maternity care, placing women's experiences at the forefront of its recommendations, alongside a multidisciplinary approach to care delivery. This vision calls for maternity services to become safer, more personalised, kinder, professional and more family friendly; where every woman has access to information to enable her to make decisions about her care; and where she and her

baby can access support that is centred on their individual needs and circumstances (NHS England, 2016). In the UK the Maternity Voices Partnership (MVP) meetings held across the UK include teams of women and their families, commissioners and providers aimed at capturing the essence of this call by working together in the development of local maternity care and the implementation of the *Better Births* agenda (Newburn & Fletcher, 2015). Although the report is a step in the right direction, a recent study highlighted the underrepresentation of disadvantaged and vulnerable women's voices in maternity research and engagement due to difficulties in accessing hard to reach women (Moreton et al., 2016). This issue is echoed by Ebert et al (2014) who acknowledge that women with complex life situations are also less likely to highlight poor care experiences through existing channels due to a mistrust of the system. Consequently, these women may decide not to engage with services in future.

Pairman (2006) believes that the key changes needed to tackle poor experiences of midwifery care begin with the co-production of services that adopt a bottom up approach to reform and design, led by and for women and their families. Mackintosh also notes the need to encourage policy makers to involve women in a meaningful way when designing and implementing programmes to enhance maternity care, ensuring that these are representative for all women (Mackintosh et al., 2018). Barley (2017) argues that policy makers must first acknowledge their part in reformative changes in healthcare reiterating the importance of self-reflection, individual impact and a collaborative endeavour towards a shared goal to facilitate change. In their *'Going Digital'* report the RCM recently summarised recommendations from the Maternity Service Review, highlighting the need for services to utilise digital innovation to maximise impact, enhance access to information and improve the levels of satisfaction for women and families (RCM, 2016). However, within this document less attention has been given to the utilization of digital solutions and innovations in maternity education and the role this may play in providing meaningful learning experiences for those delivering maternity care in the NHS.

2. 14 Digital innovation in education

The need for digital innovation to revolutionize the NHS is paramount (Honeyman et al., 2016, NHS England, 2016). This calls for the development, design and implementation of cutting-edge solutions to enhance efficiency, empower service users and transform

current systems. The emergence of advanced digital technologies and the widespread use of smartphones opens unprecedented opportunities for innovation in healthcare education (Honeyman et al., 2016). The Department of Health released a report in 2010 calling for innovative and sustainable interventions to improve outcomes, with the patient experience at the core of its vision (DOH, 2010). However, rather than focusing on transforming processes through innovation, Wadmann & Hoeyer suggest that the focus should shift to transforming realities and current systems opposed to investments in digital reforms (Wadmann & Hoeyer, 2018).

Visser et al (2018) believes that if the digitalization of services is not approached with an equality lens, healthcare systems risk widening inequalities due to unequal consumer engagement. It has also been argued that digital solutions in healthcare such as computer enhanced systems, paperless record keeping and electronic auditing tools have largely been used within a scientific paradigm to enhance processes, streamline systems and used diagnostically within healthcare (Agarwal et al., 2010, Hollis et al., 2015, Crispi et al., 2018). That said, some argue that digital technology is not necessarily about commercialisation, nor is it necessarily about scientific bureaucracy, stating its use can be deeply emancipatory (Topol 2012, Lupton, 2013). Goodman et al (2015) believe that the primary use of healthcare technologies should be to enhance efficiency of systems so that healthcare staff have more time to spend on human aspects of care. In addition, the rise of digital humanities reflects how the printed word is no longer the main medium for knowledge production and distribution and thereby opens a whole new playing field for innovators in educational delivery (Wald et al., 2018). As maternity services move into the digital age, findings encourage educationalists to ensure innovations and interventions are designed using humanistic frameworks focused on enhancing self-actualization and emotional wellbeing, helping women achieve positive birth experiences and to support the psychological wellbeing of those delivering care.

2.15 Current advances in digital education

While it may be argued that the adoption of digital technologies is slow and disparate in the NHS (Collins, 2018), a recent systematic review identified that many healthcare institutions have successfully implemented digital technology for impact (Dascal et al., 2017) yet reported the need for larger, well-controlled studies to assess if these approaches are cost-effective. One digital application that is of interest within this study

is the use of virtual reality (VR) to enhance user perceptions. VR has been reported as useful in redirecting patients' attention during painful treatments by reducing anxiety, discomfort, or unpleasantness (Wiederhold & Wiederhold, 2007). VR has also been utilized in many studies to distract patients (mainly children) during burns care, and in exposure therapy for the treatment of phobias and PTSD, by creating alternative imaginative feared environments (Shingleton et al., 2013, Serdar et al., 2014, Faireburn & Patel 2017, Rizzo & Shilling, 2017, Platkin Link & Kwan, 2017).

As a distraction technique and educational tool, VR has also been used to address eating disorders and obesity, encouraging patients to improve body image perceptions and adopt healthier eating habits (Wiederhold, 2016). Its use also spans across multiple disciplines in heath including patient motor rehabilitation, aiding patients to reacquire specific skills and improve body movement in virtual environments (Weiss et al., 2009). While current evidence suggests that VR may be used as a therapeutic training tool, its application as a reflective tool is unexplored. Given the lack of robust studies exploring the use of VR in maternity education the gap in the literature provides researchers with unprecedented opportunities to develop such applications in this field.

Several authors have reported an increase in knowledge retention when using VR tech as opposed to conventional teaching methods (Alverson et al., 2015, Ekstrand et al., 2018, Olmos-Raya et al., 2018). VR offers an alternative approach to learning, such as providing participants with immersive simulated environments to raise critical awareness of oppressive elements of care and to facilitate reflections upon practices encouraging professionals to engage in humanistic, woman-led care provision.

2.16 Rationale for study

Women's experiences of childbirth are integral to the nurturing and development of competent, confident and independent mothers. Women who are disadvantaged and marginalised due to complex life factors, such as poverty, mental health are at an increased risk of experiences a traumatic birth and suffer from PTSD. While maternity professionals are required to provide maternity care to women from diverse backgrounds, current maternity education does not address birth trauma/PTSD and lacks the resources and knowledge to support them to deliver an equitable service for marginalised women. Initiatives to combat perinatal mental health problems in the UK also tend to be focused on the treatment of trauma and PTSD as opposed to adopting

preventative measures, which if addressed, could transform care practices and experiences for women during childbirth.

A culturally sensitive and trauma informed approach to maternity care needs to be introduced into continuous professional development requirements of maternity professionals to ensure care delivery is respectful, equitable and sensitive. The application of digital humanities via immersive technology in maternity education could provide an effective platform for the recognition, treatment and knowledge of birth trauma and PTSD amongst midwifery staff to improve outcomes and reduce the number of women experiencing a traumatic birth. The use of a virtual reality medium for knowledge translation could play an important role in enhancing perceptions, promoting critical reflection and raising awareness of the difficulties that these women face within the social space of birth.

Therefore, the aim of my study was to explore traumatic birth experiences amongst this group of women to help design a tailored educational programme to sensitise maternity professionals to the potential for traumatic birth experiences amongst disadvantaged and vulnerable women. This includes the acknowledgment of digital advances in education, presenting opportunities to utilize and adopt innovative approached to the dehiscing of educational resources.

2. 17 Conclusion

In this section, I have drawn on insights from the wider literature to provide justification for my study - the design and evaluation of an immersive educational programme focused on raising awareness of birth trauma and PTSD amongst maternity professional. While disrespectful and abusive care practices amongst disadvantaged and vulnerable women in maternity care have been reported in low to middle income countries, less is known about how such women experience childbirth in high income countries. Such insights are important to identify possible additional context specific factors associated with negative experiences of care for specific groups as a basis for designing any potentially effective interventions.

The next chapter discusses steps taken in identifying and synthesising existing literature focused on disadvantaged and vulnerable women’s experiences of maternity care in high-income countries. This step is required prior to exploring the design of an

educational programme to identify key issues reported by women from this targeted group.

CHAPTER 3: META-ETHNOGRAPHY

3.1 Introduction

The previous chapter positioned negative experiences of birth as detrimental to maternal and fetal wellbeing. It also identified a lack of education amongst maternity professionals on how to care for women with complex needs during the childbearing continuum. In this chapter I present the methodology and findings of a systematic review and meta-ethnography synthesis that focuses on exploring disadvantaged and vulnerable women's experiences of maternity care. The aim was to identify any existing literature undertaken in high income countries to help inform the empirical phase of my study (i.e. interviews with local women) and ultimately to inform the educational programme. In the following sections I provide an overview of the methods, methodology and synthesised findings from a meta-ethnography into vulnerable women's experiences of antenatal and intrapartum maternity care in high income countries.

3.2 Aim of the systematic review

As identified in chapter two, poor relations with health care providers is one of the main contributory factors to women experiencing their birth as traumatic. While existing reviews have highlighted women's poor experiences of maternity care (Browser & Hill, 2010, Bohren et al., 2016), most of the included literature was from low and middle-income countries. The transferability of these findings to high income contexts is problematic due to differences in healthcare delivery and availability of maternity care globally. Furthermore, current literature lacks a focus on the interpersonal factors and interactions associated with poor experiences of care. As my study aims to design an educational programme to raise awareness of birth trauma and PTSD in maternity professionals, a first key stage was to explore and synthesise existing literature of vulnerable women's experiences of maternity care, with a specific focus on key interpersonal factors. The focus could have been purely to identify research that explored disadvantaged women's accounts of birth trauma, as vulnerable women are known to be more likely to have negative experiences. However, it was felt that a more inclusive approach would lead to a better understanding of their experiences of maternity care.

The aim of this systematic review was to gather, quality assess, synthesise and interpret the current literature that explored the maternity care experiences of disadvantaged and vulnerable women in high income countries – with a specific focus on interpersonal interactions between women and their maternity care providers.

3.3 Research question

'What are the lived experiences of maternity care interactions for disadvantaged and vulnerable women in high income countries?'

3.4 What is a systematic review?

A systematic review aims to locate, appraise and synthesize the best available research (Booth et al., 2016). It aims to identify the existing evidence that fits pre-specified inclusion criteria relating to the research question and is more robust than traditional methods that are open to bias and subjectivity (Campbell et al., 2015). Systematic reviews can provide informative and evidence-based answers to a specific research question, as well as highlighting areas where further research is required (Boland et al., 2017). In line with the trademark steps of a systematic review in the following sections I have documented all the decisions and methods adopted, thereby providing a clear and transparent audit trail for reproducibility purposes (Thomas & Harden, 2008, Barnett-Page & Thomas, 2009).

3.5 Methodology

3.5.1 Meta-ethnography

First, as the purpose of the review was to identify women's views and experiences of maternity care, as opposed to, e.g. a focus on outcomes, it was necessary to adopt a qualitative based approach. Noblit and Hare's (1988) meta-ethnography method was chosen due to its ability to explore a variety of qualitative studies focused on a phenomenon to form new interpretations of a research field. The aim of a meta-ethnography is not to condense and aggregate the findings such as in a meta-analysis, but rather to allow for an enhanced interpretation of the data set (Walsh & Downe 2005). In this review I followed Noblit and Hare's seven-step framework as seen in figure 1.

A fundamental tenet of the meta-ethnographic approach is the translation of studies which can be undertaken in three ways dependent upon the data identified (Noblit & Hare 1988). First, reciprocal translation, whereby similarities are identified across the

included studies. Second is refutational translation, which involves exploring and explaining contradictions. Finally, the line of argument synthesis involves building up a picture of the whole through a new conceptualisation of the data set (Noblit & Hare, 1988).

Figure 1 Noblit & Hares (1988) seven phase meta-ethnography.

1. Getting started (the search)

2. Deciding what is relevant to the initial interest

3. Reading studies and extracting data

4. Determining how studies are related (identifying common themes and concepts)

5. Translating studies (checking first and/or second order concepts and themes against each other)

6. Synthesising translations (attempting to create new third order constructs)

7. Expressing the synthesis.

The quality and conduct of a meta-ethnographic approach has been criticised by several researchers (Thomas & Harden 2008, Campbell et al., 2011, France et al., 2014). A recent systematic review of meta- ethnographies highlighted flaws in 66% percent of the 32 included papers due to methodological errors (France et al., 2014). Within the review 13% did not address the research aim specified, only 31% clearly described how they analysed data from primary studies and only one detailed step 6, i.e. synthesising the translations (France et al., 2014). Advocates argue how procedural and reporting flaws hinder the use of meta-ethnographies in health research and policy as the lack of rigour and transparency challenge the trustworthiness of findings (Campbell et al., 2011, Tong et al, 2012., France et al., 2015). In my meta-ethnography I drew on best practice (Rutter et al., 2010), with clear attention paid to the audit trail, reporting on decisions made against the seven-step approach for transparency purposes. Below each step of Noblit & Hare's (1998) approach is detailed and discussed.

3.6 The search (phase 1)

Getting started, the first step in the approach is concerned with undertaking the literature search. The process of gathering evidence and papers for a meta-ethnography remains an area of contention, as the original work offers no guidance as to how it

should be undertaken. Debates have therefore been concerned with poorly defined search strategies that adopt a priori or inductive approaches to gathering papers (Walsh & Downe 2005, Thomas & Harden 2008), alongside poor sampling methods that undermine the rigorous and transparent nature of undertaking a systematic review (Tong et al., 2012). Although many undertaking a meta-ethnography may rely on conceptual saturation, this approach is criticised in favour of an approach that systematically attempts to locate as many potential sources as possible (France et al., 2014). While there is no clear guidance on the level of data collection needed, I adopted a systematic approach to locating the most relevant papers through peer-reviewed journals using best practice systematic review techniques. It was thought that this was the most suitable approach to locate the all relevant sources that reported women's experiences of maternity care in high-income countries. Due to the large amount of papers identified in an initial scoping search, a decision was made to only focus on published papers, rather than grey literature.

Search terms were developed in stages. First, the research question was broken down into its components to form the basis of the pre-specified eligibility criteria for the review (Higgins & Green 2011). The '*Population and their Problems, Exposure and Outcomes or Themes'* (PEO) framework (Bettany-Saltikov, 2012) was utilized to develop a searchable and answerable research question. The next step was to further develop search terms by identifying related synonyms to ensure the comprehensiveness of the search strategy (Bettany-Saltikov, 2012). For example, in relation to the search term 'birth' all terms which refer to birth i.e. parturition, childbearing, child-bearing, childbirth, labour were used. It was also decided to develop a search string associated with qualitative methods of inquiry to help focus the search strategy. These were charted and reviewed by my supervision team to ensure the search strategy was fit for purpose. In Table 2 the search terms (and associated rationale for inclusion) are mapped against the PEO framework.

Table 2 PEO framework and search terms.

PEO	Inclusion	Rationale	Search terms
Population and their problems.	Women	Exploring women only experiences of care – with further checks made to only include research undertaken on women who are disadvantaged and vulnerable.	Wom?n* OR maternal OR mother*OR patient OR consumer OR service user OR service-user
Exposure.	Perinatal/intrapartum period	To identify literature that focuses on women's experiences of antenatal and/or intrapartum care.	prenatal OR pre-natal OR prepartum OR pre-partum OR antenatal OR ante-natal OR perinatal OR postnatal OR post-natal OR postpartum OR post-partum OR pueperium OR puerperal OR intrapartum OR intranatal, OR birth OR parturition OR childbearing OR child-bearing OR childbirth OR labour not work OR labor not work
Outcomes or themes	Women's views and experiences.	To elicit accounts of care from women's stories and recollections.	Experience* OR perspective* OR view* OR perception* OR encounter* OR account*OR description* OR opinion* OR observation* OR satisfaction
Additional criterial to identify relevant papers	Qualitative research papers	Undertaking a meta-synthesis focused on qualitative research studies.	qualitative OR ethnograph* OR phenomenol* OR "grounded theor* OR hermeneutic* OR "lived experience*" OR "symbolic interaction*" OR narrative* OR "life experience*" OR "action research" OR observation* OR "focus group*" OR interview* OR "mixed method*" OR mixed-method* or "multimethod"

3.7 Enhancing retrieval methods in databases

Boolean operators, truncation and wildcard were used where appropriate within each database searched. MeSH subheadings were also used where possible to ensure the searches were as efficient as possible. MeSH is a controlled vocabulary thesaurus, located within a database (Lowe & Barnett, 1994). The MeSH thesaurus consists of terms which identify related descriptions of the search term inputted within a hierarchical structure allows you to search at varying levels of specificity (Lowe & Barnett, 1994).

Using MeSH terms provided a comprehensive retrieval method for identifying appropriate literature within this study.

3.7.1 Berry picking

Conventional methods of literature searching have been criticised as mechanical and rigid, subsequently effecting the quality of retrieved data (Bates, 1989). As an alternative to conventional methods of literature searching, Bates (1989) recommends *'Berry Picking'* which includes a more iterative and fluid approach to searches. Bates believes that new information retrieved provides new ideas and lines of inquiry encouraging researchers to create a new perception of the original query (Bates, 1989). When utilizing berry picking approaches, rather than beginning with one single query, an iterative and flexible approach is used to re-direct the search, producing new and relevant information (Bates, 1989). The numerous methods suggested include citation searching (detecting literature which has cited articles of interest); footnote chasing (chasing up footnotes found articles/books of interest to recognise new references); area scanning (looking through material located under the same subject heading); journal run (searching through significant journals within subject areas) subject searches in bibliographies and abstracting and indexing services (such as classic information retrieval methods) and searching for authors.

A review by Greenhalgh & Peacock (2005) measured the effectiveness of different search methods and concluded that classical approaches (i.e. databases) only identified a quarter of the relevant literature and snowballing techniques (i.e. checking references and citations) were the most efficient with this method retrieving almost half of the total sources identified.

3.8 Methods used during the search

By combining the berry picking approach of Bates (1989), within the stages discussed by Greenhalgh & Peacock (2005), a systematic, yet iterative, approach was taken during the search phase. These methods are detailed in more depth as follows:

3.8.1 Database searches

The first strategy consisted of searching for literature within five key databases; MEDLINE; PschInfo; CINAHL; EMBASE; Cochrane databases. Prior to undertaking each database search, all search terms were piloted and assessed for efficacy in retrieval. A search log was kept that documented all search terms used and which combinations

were applied (see appendix 1 for an example of a database search). Times and dates of searchers and retrieved numbers were also documented.

3.8.2 Area scanning / journal run

Key journals were searched for all relevant articles. Due to time constraints high impact and relevant journals were prioritized and included - International Journal of Women's Health, Midwifery, Women and Birth, PLOS, BJOG, An International Journal of Obstetrics and Gynecology, Journal of Advanced Nursing, Social Science and Medicine, Journal of Sociology, Birth, Journal of Obstetrics, Gynecology and Neonatal Nursing.

3.8.3 Footnote / reference chasing

The reference lists of all included relevant papers were checked for any papers of relevance.

3.8.4 Citation tracking

Citation tracking was undertaken. This included using google scholar and web of science to identify/review any literature that had cited the relevant papers (Greenhalgh & Peacock 2005).

3.8.5 Author run

Searches were undertaken against known authors in the field of midwifery and birth trauma, these included Pauline Slade, Julie Jomeen, Susan Ayers, Cheryl Beck, Gill Thomson and Soo Downe.

3.8.6 Personal knowledge resources

As a midwife I was familiar with key texts and articles, with my supervisory team also providing me with appropriate texts. Email contact was made with a number of authors. I also attend and spoke at conferences, both national and international, enabling additional sources to be discovered.

3.9 Deciding what is relevant to the initial interest (Phase 2)

Inclusion and exclusion criteria were developed (see Table 3). These were developed prior to the literature search in order to ensure a robust and efficacious approach was adopted (Thomas & Harden, 2008).

The decision to include papers from 1993 onwards was due to the publication of the UK governmental policy '*Changing Childbirth*' (DOH, 1993). This marked a change in the discourse surrounding childbirth and prioritised women's right to choice, control, and continuity of carer. It also highlighted that the hospital is not always the safest place for

women to give birth (Walsh, 2007), paving the way for a new maternity rhetoric, which included alternative birth place choices.

The criteria used for this study were:

1. Participants: Women who had experienced all types of delivery were included at this stage as the study concerns itself with the experience of childbirth, not the mode of delivery. Childbirth experiences given by other members including those present i.e. fathers/midwives were to be excluded. When reviewing the paper, additional criteria to screen for disadvantaged and vulnerable characteristics were included.
2. Exposure: This related to only including studies that concerned the antenatal/intrapartum experiences. As the aim was to focus on how interpersonal interactions influenced women's experiences of childbirth, papers concerned with the postnatal period were not included.
3. Outcomes: All accounts of childbirth (positive or otherwise) remained of interest at this stage in the review. This was in order not to miss any key insights into women's experiences.
4. Type of study: In line with meta-ethnography only qualitative studies were to be included. This related to studies that had a qualitative focus (i.e. grounded theory, phenomenology, ethnography) or papers that adopted a mixed methods approach in which case the qualitative aspects of the study would be assessed for relevance.
5. Location: The fifth criteria related to location to ensure studies were undertaken in a high-income country to ensure experiences captured were of direct relevance to my study.
6. Additional criteria: Additional criteria related to the dates for searching, and that only studies in English were to be included.

Table 3 Inclusion exclusion criteria.

Considerations	Inclusion criteria	Exclusion criteria
1.Participants	• Women who have experienced childbirth<br>• Women who meet a definition of disadvantaged and vulnerable	• Accounts of childbirth given by other members of the women's family/friends or healthcare professionals

		• Women who were not disadvantaged or vulnerable
2.Exposure	• Studies that focus on the experience of childbirth during the perinatal/intrapartum period	• Papers that focus on postnatal experiences
3.Outcome	• Views and experiences of women's experiences of antenatal or intrapartum care	• Papers that don't provide rich accounts of these experiences
4.Type of study	• Qualitative and/or adopting a mixed-methods approach with a qualitative component.	• Experimental (randomised controlled trials, controlled trials, quasi-experiments)<br>• Non-experimental (surveys/questionnaires/ cohort studies)<br>• Discussion/opinion papers<br>• Grey literature<br>• Dissertations
5. Location	• Care experienced in a high-income country as defined using World Bank classification of high income in respect to economy/growth and health status (The World Bank 2019)	• Not a country classed as high income as defined by the World Bank
6. Additional criteria	• English language.<br>• Papers from 1993 to 2016 (Updated search undertaken in 2018)	• All other languages.

Following this stage, a full text screen was undertaken on papers remaining.

3.9.1 Full text screening

Following initial screening against the primary inclusion / exclusion criteria detailed in table 3. Papers identified were read in full to ensure the focus of the study was on the experience of antenatal or intrapartum care amongst disadvantaged and vulnerable women in high income settings.

3.9.2 Quality appraisal

All texts considered suitable for inclusion following full text review were then subjected to a quality appraisal process. Quality assessment aims to distinguish between poorly conducted studies and those that follow a robust methodology (Hannes & Lockwood 2011). There is however, debates surrounding the significance of quality assessments

when undertaking a qualitative synthesis (Atkins et al., 2008, France et al., 2014). These debates centre on the risk of excluding poorly reported studies that contain important and meaningful insights (Britten & Pope, 2011). Others highlight difficulties in reporting qualitative findings in journals with restricted word counts, hindering the ability of those undertaking research to condense and present meaningful representations of data (Campbell et al., 2011). In contrast, others report on the value of robust measures of quality to ensure minimum standards of evidence based recommendations are met (Walsh & Downe, 2006, Thomas & Harden, 2008, Campbell et al., 2011). After consideration it was my belief that a quality appraisal of included studies added an extra layer of rigor to the systematic process that would enable me to assess their credibility, transferability, dependability, and confirmability (Lincoln & Guba, 1985, Walsh & Downe, 2006).

The Walsh and Downe (2006) tool was used to undertake the quality assessment process. This tool was developed from a synthesis of a wide range of existing assessment tools and involves assessing each paper against the following criteria:

- Scope and purpose
- Design
- Sampling strategy
- Analysis
- Interpretation
- Ethical dimensions
- Relevance and transferability

In line with the Walsh and Downe tool, a grade from A-D was then assigned to each paper as seen in table 4.

Table 4 Walsh & Downe's (2006) scoring criterial for quality appraisal.

A: No, or few flaws. The study credibility, transferability, dependability and confirmability are high;<br>B: Some flaws, unlikely to affect the credibility, transferability, dependability and/or confirmability of the study;<br>C: Some flaws that may affect the credibility, transferability, dependability and/or confirmability of the study.<br>D: Significant flaws that are very likely to affect the credibility, transferability, dependability and/or confirmability of the study.

Quality assessment of 20% of papers was carried out by my supervisor Dr Gill Thomson. We then met to discuss our individual findings and as we were consistent in our scores I quality appraised all the remaining studies. Following this step, I met with my supervision team to discuss the findings and grades and to agree on the papers to be included in the next stage of review.

3.10 Findings

The database searches were initially undertaken between 21st June 2016 - 1st of July 2016. A further search in each database using the same search terms was also undertaken on the 8th of April 2019 to identify any further papers that had been published. Overall, 13,330 papers were retrieved following initial database searches and a further 22 papers were identified from additional sources. Following removal of duplicates 10,458 remained. All papers at this stage were examined and papers eliminated by screening title / abstract. This left 458 papers remaining. Studies were then assessed against the inclusion and exclusion criteria (Table 3), resulting in 73 potentially suitable papers. Following full text screening and application of the quality appraisal framework, 18 papers were included within the synthesis.

In summary, one paper was allocated an A grade due to its rigorous and methodologically sound reporting of the data. Twelve of the studies were of good quality and allocated a B grade, while these studies were assessed as having a clear methodological design with appropriate methods used. A lack of reflexivity was noted across all the studies alongside a lack of reported theoretical approach taken. Five studies were given a C grade as notable weaknesses were apparent when applying the quality criteria. These included a lack of justifications for the study, a lack of reflexivity, triangulation and theoretical insight. For a full description, all quality assessments can be seen in appendix 3.

The PRISMA diagram seen in figure 2 provides a visual representation of the searches and screening results.

Figure 2 Prisma Diagram

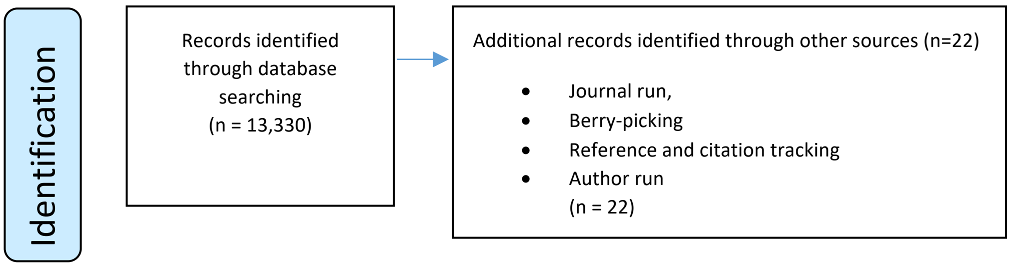

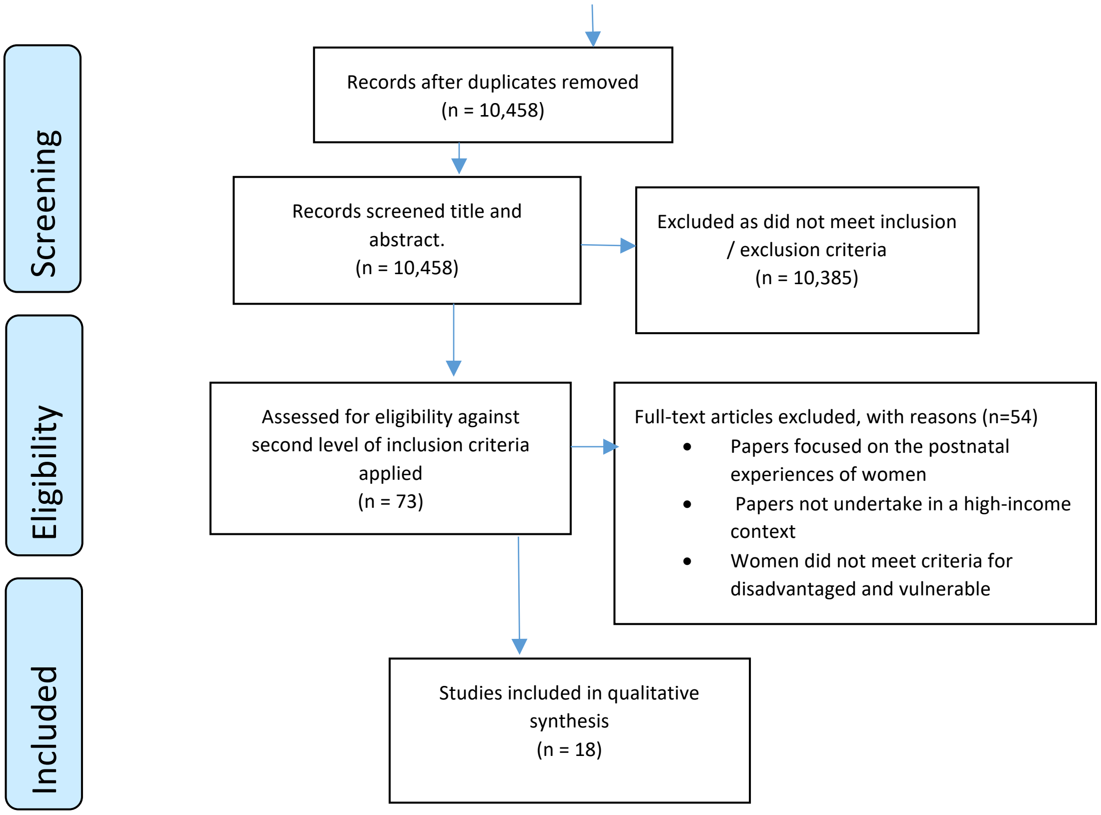

The 18 papers included within the synthesis incorporated the views of 578 women's experiences of maternity care in high income countries. These studies were undertaken in the UK (n=10), USA (n=3), Sweden (n=1), Canada (n=1) Scotland (n=1) Australia (n=1) and Norway (n=1). Table 5 presents an overview of the study's characteristics together with the quality assessment grade.

Table 5 Study codes, summary of characteristics and grades on included studies

Study code	Authors	Year of publication	Country	methods used	Number of participants	Quality grading
1	Briscoe & Lavender (2009)	2009	UK	Longitudinal exploratory study using multiple case studies	4	B
2	Ny et al (2007)	2007	Sweden	Focus groups and individual interviews	13	B
3	Reitmanova & Gustafson (2008)	2008	Canada	Qualitative narrative enquiry using semi-structured interviews	6	C
4	Davies & Bath (2001)	2001	UK	Focus group and semi-structured interviews	13	B

5	Cross-Sudworth et al (2011).	2011	UK	Semi – structured interviews and focus groups	15	B
6	Bailey et al (2004)	2004	UK	Focus groups and semi-structured interviews	38	C
7	Ebert et al (2014)	2014	UK	Focus groups recorded using three separate groups	17	B
8	Herrel et al (2004)	2004	USA	Focus groups	14	C
9	Price & Mitchel (2004)	2004	UK	In-depth interviews	10	B
10	Arthur et al (2007)	2007	UK	Semi – structured interviews	8	B
11	Robb et al (2013)	2013	Scotland	Unstructured interviews	7	B
12	Jomeen & Redshaw (2013).	2013	UK	Survey data and questionnaire with open ended responses	219	A
13	Shafiei & McLachlan (2012)	2012	Australia	Over the phone interviews using closed questions. Face-face Interviews in participants homes	50	B
14	Spidsberg (2007)	2007	Norway	Semi structured interviews	6	B
15	Ward et al (2013)	2013	USA	Focus groups	29	B
16	Wilton & Kaufman (2001)	2000	UK	Self-completion questionnaires with free text questions	50	C
17	Howard (2015)	2015	USA	Group interviews	20	C
18	McLeish & Redshaw (2018)	2018	UK	Semi structured interviews	40	B

3.11 Initial reading and familiarisation (phase 3)

During this stage papers identified to be included within the synthesis were read and re-read, building upon the familiarisation of the studies that began during the quality assessment phase. Notes were made regarding any initial impressions or interpretations relating to the studies. Each paper was then uploaded into MAXQDA, a computer software package that offers tools for the organisation and analysis of qualitative data. Using software at this stage enabled me to organise a large body of data by coding findings from each paper. The task of coding enabled me to get a deeper sense for the papers, whilst also being able to observe emergent concepts and themes.

Each individual paper was read paying attention to any metaphors that represented the study's findings (Noblit and Hare, 1988). Finfgeld-Connett (2008) describes this process as developing conceptual categories to help understand an experience, rather than just describing the experience. How I understood this is that a concept is a meaningful idea that must explain not just describe the phenomena.

I also used the meta-synthesis template devised by Downe et al (2006), as a guide to help me detail the codes assigned to each paper and then map these against the ideas, concepts, relations and themes within each original text. The codes were named *'First order constructs'* (Campbell et al., 2011). Although Noblit and Hare do not refer to *'Constructs'* within their work, this term has been used within meta-ethnographic studies (France et al., 2014) and deemed suitable when undertaking this phase.

3.12 Determining how studies are related (phase 4) translating studies (phase 5) synthesising translations (phase 6)

These phases were undertaken simultaneously whereby first order constructs were refined through an iterative process of reading and re-reading the papers/codes generated into second order constructs. Shutz (1962) describes first order constructs as participants *'common sense'*, interpretations in their own words and second order constructs as the researcher's interpretations. This involved an iterative process to ensure that any/all similarities (reciprocal) and differences (refutational) were noted.

A further stage of analysis involved the second-order constructs being grouped into third order constructs; this involved an iterative process of synthesising these constructs into more conceptual level categories. I returned to Phases 3-6 repeatedly during this process and the translations were synthesised by the process of interpretive writing. This step attempted to bring together the parts into a new interpretation (Walsh & Downe, 2005). Several repetitions of this task were carried out until the third order constructs offered meaningful and authentic representations of the whole data set.

The final translational phase is the line of argument. This seeks to move beyond the translation into a new interpretative whole (Noblit and Hare 1988, p. 64). A line of argument *'I know my place'* was developed and is discussed in section 3.18. Table 6 details the first, second and third order constructs mapped to each of the papers included in the synthesis with codes ascribe dot each included study documented in

table 6 enabling the identification of representative studies within the first order constructs.

Table 6 Synthesis interpretations.

First order constructs (code of contributing study(ies)	***Second order constructs***	***Third order constructs***
Lack of continuity (4,6,9,12,13,17,18)<br>Lack of choice (1,3,4,6,15,18)<br>Feeling the need to conform (1,4,6,7,13,10, 16)	**Disengaged from birth process**<br><br>*'Every time I saw the midwife during pregnancy and labour, I felt that I was just being processed, there was no opportunity to develop a working relationship'* (Jomeen & Redshaw, 2013, p. 286)	Depersonalization
A lonely experience (4,7,12, 15)<br>Cultural incompetence (2,4,8,13, 15)<br>Need for culturally contextual understandings (1,3,17,14,13,8, 18)<br>Language barrier (2,4,5,8,14,18)<br>Poorly communicated information (1, 3,4,10,12,13,18)<br>Communication failure (4,5,8,14,18)	**Lack of cultural contextual care**<br><br>*'They have no idea what is halal food……They offered me bacon and asked me if I can eat it. So I said 'I can't.' They told me that someone will come and ask me what food I need. And nobody came until I left the hospital.'* (Reitmanova & Gustafason, 2008, p. 106)	
Craving empathy (4,5,9,18,13)<br>Need for interactions (3,5,9,13, 18)<br>Need for compassionate care (3,7,10,13, 18)<br>Rude staff (5,18,12,13,14,15,16)	**Poor emotional connections**<br><br>*'I understand there is a staff shortage and staff are under a lot of pressure but attitudes should remain sympathetic towards mothers.... as giving birth can be very traumatic and care received has a lasting effect on their lives and views about hospital care'* (Jomeen & Redshaw, 2013, p. 286)	No care in the care
Regimented care interactions (3,7,9,1017)<br>Seeking out care entitlement (7,13, 14)<br>Driven to extremes (3,7,8,9,13,14)<br>Acceptance of substandard care (3,7,10, 17,18)<br>No other option (3,7,14,18)<br>Negotiating needs (7,10,18)	**Submissive interactions**<br><br>*'The midwife was very cold she wanted me to agree with her, and I didn't try to ask questions'* (Davis & Bath, 2001, p. 243)	
Subservient interactions (3,4,5,13)<br>Shamed (7,9,18,13,17,18)<br>Demoralized (6,8,12,18,13)<br>Being Ignored (3,4,5,6,7,9, 10,11,13,14)<br>Paternalistic care (3,4,6,10,11,13,16)	**Demoralising interactions**<br><br>*'She (midwife) treated me like a child more than older women, she treated them like a friend, because when she used to call them in they would get up and start chatting and stuff. But when it was my turn she would more or less direct me which room to go into and that was it and then make me sit down for my blood pressure'* (Price & Mitchel, 2004, p. 3)	Dehumanisation
Abandoned (8, 12,16)<br>Feeling Vulnerable (3,5, 8, 11,12,17,18)	**Absence of human rights in maternity care**	

Left to suffer (3,6,7,11,12)<br>Feeling abused (4,5, 6,7,11,12,18)<br>Scared (1,3,4,10,11,17)<br>Felt punished (2, 5, 6, 8, 16,18)<br>Feeling victimized (2, 6, 8, 10, 17,18)	*'An internal examination at nine months was so rough it made me bleed, and worse, was so painful and frightening I felt I had been assaulted'* (Wilton & Kauffman, 2001 pg 209)	
Judged (1,3,4,8, 10,18,13,16,18)<br>Feeling Stigmatized (3, 5, 8, 9, 10,18,13,18)<br>Prejudice from staff (3,4, 9, 11, 14,15,18)	**Judgemental attitudes**<br>*'I told [my midwife] I didn't like going to my appointments, and one day she just asked me, 'do you do crack?'… Just because I don't want to come to my appointments, I got to be a drug addict?'* (Ward et al., 2013, p. 1756)	Them and us
Comparing their care to others around them (2, 4,,17,18)<br>Lack of self-efficacy (2,10,11,13,16 18)<br>Made to feel guilty (2,4,14,17, 18)<br>Mistrust of professionals (2,4,11,17,18)	**Instilling inferiority**<br>*'When I saw her with the other women in the hospital and she was so respectful: `What do you want to do', and `It's your baby?' Not like with me'* (Davis & Bath 2001, p. 243)	

3.13 Expressing the synthesis (Phase 7)

In this section I present the findings of the synthesis and the resulting line of argument translation. Four third order constructs were identified relating to women's negative experiences, thoughts, feelings and views of maternity care - '*Depersonalisation*', '*No care in the care', 'Dehumanisation'* and *'Them and us'*. While a key focus of this synthesis was to identify factors that were associated with a negative/distressing birth – with this data then being used to help inform the planned educational programme - in line with Noblit and Hare's refutational translation, any contrasting data has been integrated and reported as appropriate. In the following sections, each of the third order constructs are described together with supporting quotes from the included studies.

3.14 'Depersonalization'

'*Depersonalization*' highlights the institutional marginalization of women and how interactions with health care providers rarely accounted for personal and individual preferences. Two second order constructs are detailed. First, *'Disengaged from the birth process'* reflects how women felt disengaged from the childbirth process due to negative and insensitive interactions with health care professionals. Second *'Lack of cultural contextual care'* relates to women's cultural, religious, sexual and psycho-social needs being neglected, leaving women feeling their identity was under threat.

3.14.1 Disengagement

In 11 of the studies, women described feeling disengaged from the care they received. (Wilton & Kaufman 2001, Davis & Bath 2001, Bailey et al., 2004, Price & Mitchell 2004, Arthur 2007, Reitmaonova & Gustafson 2008, Briscoe & Lavender 2009, Shafiei & McLachlan 2012, Ward et al., 2013, Jomeen & Redshaw 2013, Ebert et al., 2014). Women described objectifying encounters with their healthcare providers highlighting the impersonal process of accessing care and the barriers this created in developing relationships with their healthcare professionals:

> *'Every time I saw the midwife during pregnancy and labour, I felt that I was just being processed, there was no opportunity to develop a working relationship'.* (Jomeen & Redshaw, 2013, p. 287).

The lack of engagement between women and their healthcare professionals led some women to seek out their own care. This was reflected by a UK born black African woman in the study by Jomeen and Redshaw:

> '*During pregnancy it feels as though you are left to your own devices. Sometimes I had questions that I feel were not taken seriously, in the end I did my own reading' (*Jomeen & Redshaw, 2004, p. 284).

This sentiment was also echoed by women in a paper by Arthur (2007) that explored teenage mothers' experiences of maternity care. One woman expressed her feelings of isolation and disengagement from her midwives:

> *'The midwives don't bother with you, you're on your own.'* (Arthur 2007, p. 676).

One study that explored socioeconomically disadvantaged women's experience of maternity services in the UK provided contrasting data (McLeish & Redshaw, 2018). Within the study, women who received specialist care were more likely to feel acknowledged and valued as an individual:

> *'She [the specialist midwife] thought about me as a person, not just a pregnant mum'* (McLeish & Redshaw, 2018, p. 4).

3.14.2 Lack of culturally contextual care

A lack of culturally contextual care was evident in 12 of the studies (Davis & Bath 2001, Herrel 2004, Ny 2007, Arthur 2007, Reitmanova & Gustafson 2008, Briscoe & Lavender

2009, Cross-Sudworth et al., 2011, Shafiei & McLachlan 2012, Ward et al., 2013, Jomeen & Redshaw 2013, Ebert et al., 2014, McLeish & Redshaw 2018). On these occasions, women reported feeling that they would, or had, been mistreated due to their ethnic background:

> *'If the nursing staff see you are foreign or of a different colour, they treat you badly'* (Herrel, 2004, p. 4).

A pertinent issue across three of the studies was how requests for female doctors due to their specific cultural and religious needs was not met (Herrel 2004, Reitmanova & Gustafson 2008, Jommen & Redshaw 2013). A UK born Asian woman in Jomeen & Redshaw's study reported:

> '*Being a Muslim I requested to be seen by female doctors only during the delivery, but this request was not adhered to……. the whole situation should have been avoided, as there were female doctors available.' (*Jomeen & Redshaw 2013, p. 290).

A lack of consideration for women's cultural wishes was also echoed by a Muslim woman in the study by Reitmanova & Gustafson (2008):

> '*There was a male who entered my room, I also put a sign on the door, but they didn't respect it. This man came and saw me. I was very upset and crying.'* (Reitmanove & Gustafson, 2008, p. 106).

In extreme cases, as noted in a study by Jomeen & Redshaw (2013), some black and minority women were reported as having to rely on non-verbal cues when unable to communicate, which resulted in frightening interactions with their health care professionals*:*

> '*Some of the midwives spoke to me very arrogantly, sometimes I got scared as I don't know the English language…... It would be very nice if they gave you a smile now and then…..it would be nice if they didn't give orders'* (Jommen & Redshaw, 2013, p. 290).

Even on occasions when the midwife was from the same cultural background as the woman, this did not guarantee that the support would be positive:

> *'I had a midwife originating from my home country, but she was not nice or helpful'* (Ny, 2007, p. 8).

Refutational findings were reported in four of the studies (Reitmanova & Gustafson 2008, Cross-Sudworth et al., 2011, Jommen & Redshaw 2013, Ebert et al., 2014). Some women who experienced positive care highlighted effective communication as making a difference to their birthing experiences. One black Caribbean women accessing care in the UK but not UK born, reported how the welcoming and supportive behaviours of staff impacted upon her sense of belonging in an unfamiliar cultural context:

> *I was well cared for at (***) hospital, no one there was rude, all the staff have been great, they did not choose for me, they gave the choice. The team of midwives have been so good I did not feel worried at anytime. The midwife that took care of me during my labour was so supportive she was amazing, she did not let me feel scared at anytime, everyone was just so good I did not feel I didn't belong there, I felt like I was in good hands.* (Jomeen & Redshaw, 2013, p. 290).

3.15 'No care in the care'

Two second order constructs are reported in relation to *'Poor emotional connections'* and *'Submissive interactions*. These concepts reflect that while women wanted to feel supported and empowered, they experienced a lack of empathetic interactions with their care providers. This in turn led to women feeling anxious with increased levels of fear impacting upon their ability to advocate for themselves.

3.15.1 Poor emotional connections

Poor emotional connections were noted in 13 of the included studies (Wilton & Kaufman 2001, Davis & Bath, 2001, Price & Mitchell, 2004, Arthur et al., 2007, Spidsberg, 2007, Reitmanova 2008, Cross-Sudworth et al., 2011, Shafiei & McLachlan 2012, Jomeen & Redshaw 2013, Ward et al., 2013, Ebert et al 2014, Howard 2015, McLeish & Redshaw 2018).

In the study by Howard (2015) that focused on opioid dependant women's experiences of maternity care, a lack of provider-woman connection was evident, with women reporting feeling *'punished'* as a result of their vulnerability rather than being emotionally engaged with and supported:

> *'The providers in the unit where the babies are treated tend to punish the mothers; The mothers are making the best decisions they can, and they have a tremendous amount of guilt and sadness. I wish there were more of an emphasis on helping them parent, rather than punishing them'* (Howard, 2015, p. 2).

Ethnic minority women in a study by Jomeen & Redshaw (2013) described staff as *'insensitive'* and how they could make women *'feel an 'idiot.'*. Interestingly, in the study by Ebert et al (2014), focusing on social disadvantaged women's experiences of maternity care, one woman felt her lack of emotional connection with the midwife was directly due to external pressures on her caregiver. This woman observed tensions between lines of authority (medical v. midwife) which in turn impacted on the care she received:

> *'I could still feel it, and I'm looking at the midwife, I was crying and she's going, 'I know', and I'm thinking why you can't say anything. She [the midwife] didn't say anything she was just, I don't know. Cause he'd [the doctor] jumped in [and said], 'I will do it', and she was supposed to do it''* (Ebert et al, 2014, p. 135).

When women did not have a positive relationship with healthcare providers this could also have a lasting and negative impact on access to health care:

> *'I understand there is a staff shortage and staff are under a lot of pressure but attitudes should remain sympathetic towards mothers.... as giving birth can be very traumatic and care received has a lasting effect on their lives and views about hospital care'* (Jomeen & Redshaw, 2013, p. 286).

3.15.2 Submissive interactions

Submissive interactions were reported in nine of the studies (Herrel et al., 2004, Price & Mitchel, 2004, Arthur et al., 2007, Reitmanova & Gustafson 2008, Spidsberg 2007, Shafiei & McLachlan 2012, Ebert et al., 2014, Howard, 2015, McLeish & Redshaw, 2018) This relates to how women often felt they had no choice but to adapt to what was happening to them. Becoming submissive was a dichotomy between receiving care on

conditional terms and/or choosing not to access care due to a lack of personal care. As reflected by a mother in McLeish & Redshaw (2018), a socially disadvantaged woman who was not born in the UK felt she had to meet the demands of staff expressing how she felt pressured and threatened into accepting surgery:

> *'[The Midwife] didn't have much patience because after like six, seven hours she was like, 'they'll have to do you surgery, they'll have to do surgery' Like she is forcing me to accept that they'll have to do surgery. I was not happy but she is the doctor so…At the end she was saying, no, no, but you have to do it now, I was just saying 'Ok give me the form and I sign'* (Mcleish & Redshaw, 2018, p. 182).

One socially disadvantaged woman in a study by Ebert et al (2014) also reported feeling like a guinea pig when reliving experiences of examinations - she felt she had no choice but to conform:

> *'[I would like to] not be the guinea pig where they go, 'do you mind, once I feel how far dilated you are, if someone else has a go up there?' They need to respect if you don't [want students to do additional vaginal examinations], and you feel like you can't [say no], and you go all right, [because] if I say no then they're going to leave me alone all the time. So you sort of go, yeah, okay, even if you don't want them to [so] you're [not] going to be treated differently'* (Ebert et al., 2014, p. 136).

Conversely, women who refused to adapt to health care professionals demands and preferences reported feeling as though they had to escape care or choose not to engage with services:

> *'I had to run away from the hospital after 24 hours'* (Reitmanova & Gustafason, 2008, p. 105).

This was also highlighted by a woman in Howard's (2015) study in which an opioid dependant woman expressed how she could only sustain adaptation to the environment for a limited time before it became too upsetting for her:

> *'The way they looked at me and treated me, all of it at once was just too much for me. You know, I couldn't take it for more than like an hour at the hospital and then I'd leave. And I'd get to the point where I just wouldn't show up.'* (Howard, 2015, p. 431).

From a refutational perspective, some of the women in the study by Shafiei & McLachlan (2012) reported on how provision of adequate information allowed them to advocate for themselves:

> *I'm very happy that I was given option at 30 weeks; my baby was breech; I had my baby turning instead of caesarean section. I'm very happy to be given option to choose'* (Shafiei & McLachlan, 2012, p. 201).

3.16 'Dehumanisation'

Dehumanisation relates to how women could experience degrading interactions and inhumane treatment by healthcare professionals. Two second order constructs are presented, *'Demoralising interactions'* and *'Absence of human rights in maternity care'*.

3.16.1 Demoralising interactions

Demoralising interactions were noted in 13 studies (Wilton & Kaufmann, 2001, Davis & Bath, 2001, Bailey et al., 2004, Price & Mitchell, 2004, Arthur et al., 2007, Spidsberg, 2007, Reitmanove & Gustafason 2008, Cross-Sudworth et al., 2011, Shafiei & McLachlan 2012, Robbb et al., 2013, Jomeen & Redshaw 2013, Ebert et al., 2014, McLeish & Redshaw 2018). Demoralizing interactions were concerned with negative professional-woman exchanges that were upsetting and which undermined women's confidence. In four studies (Wilton & Kaufmann 2001, Jomeen & Redshaw 2013, Ebert et al., 2014, Howard 2015) women referred to health care professionals making inappropriate comments. An opioid dependent woman in Howard's study (2015) recalled her obstetrician saying:

> *"Get your life together." I thought to myself, "She's very unprofessional. My life is together."* (Howard, 2015, p. 430).

Women also reported paternalistic interactions with healthcare professionals in six of the studies (Davis & Bath 2001, Bailey et al., 2004, Price and Mitchel 2004, Arthur et al., 2007, Rietmanova & Gustafson, 2008, Jomeen & Redshaw, 2013). A teenage mother in the paper by Price and Mitchell (2004) expressed how she felt treated like a child during antenatal education classes and during midwife appointments:

> *'She [midwife] treated me like a child more than older women, she treated them like a friend, because when she used to call them in, they would get up and start chatting and stuff. But when it was my turn, she would direct me which room to go into and that was it and then make me sit down for my blood pressure'* (Price & Mitchell, 2004, p. 5).

Another teenage mother within the study also disclosed the negative impact of threatening phrases made during birth:

> '*She said, 'if you don't hurry up and push we'll have to take you for a caesarean' and that's not very nice.'* (Price & Mitchell, 2004, p. 683).

The socioeconomically disadvantaged women in the study by McLeish & Redshaw (2018) reported being made to feel '*low category'*, '*stupid*' and '*weird'* by professionals who made rude and thoughtless comments.

From a counter perspective, vulnerable women in the study by McLeish & Redshaw (2018) who received continuity of care expressed how this had enabled trust-based relationships to be formed:

> *'I asked my midwife [specialist] and she was really helpful so I knew what to expect'* (McLeish & Redshaw, 2018, p. 4).

3.16.2 Absence of human rights in maternity care

A lack of respectful care reported in 12 papers bordered and sometimes crossed the threshold into abusive and neglectful care (Davis & Bath, 2001, Wilton & Kaufman, 2001, Bailey et al., 2004, Herrel et al., 2004, Arthur et al., 2007, Ny et al., 2007, Cross-Sudworth et al., 2011, Robb et al., 2013, Jomeen & Redshaw, 2013, Ebert et al., 2014, Howard, 2015, McLeish & Redshaw 2018). In the paper by Arthur et al (2007) one women described how a healthcare professional refused to stop a painful procedure that resulted in her experiencing her birth as traumatic:

'I had an internal and she had a piece of skin ...I was in absolute agony. The midwife was determined she did not have a piece of skin and didn't stop, it was like it was all in my head.' (Arthur et al., 2007, p 675)**.**

A lesbian woman in a paper by Wilton & Kaufman (2001) shared a similar experience stating:

'An internal examination at nine months was so rough it made me bleed, and worse, was so painful and frightening I felt I had been assaulted' (Wilton & Kaufman, 2001, p. 209)**.**

The women in the study by Herrel et al (2004) highlighted a lack of informed consent, and how their bodies were objectified by those caring for them:

'They [The nursing staff] let the medical students practice on us' (Herrel et al., 2004, p. 347).

A woman in the study by Ebert et al (2014) noted how healthcare professionals used the threat of danger and authoritative knowledge to assure her conformity:

'You do what they say because like, you're going to be a mother and you want to do everything the special people who are the professionals tell you to do because you don't want anything to happen to your baby and if that's what they need you to do, you jump through the hoops' (Ebert et al., 2014, p. 135)**.**

A survivor of human trafficking in the study by Mcleish and Redshaw (2018) expressed a similar experience:

'She (the midwife) did not explain that to me. She just started to put – and when I shouted, she – she didn't explain nothing to me. Oh my God' (McLeish & Redshaw, 2018, p. 5).

3.17 'Them and us'

This construct concerns how women experienced a *'Them and Us'* divide with healthcare professionals. This divide had an impact upon women's psychological wellbeing during the childbearing continuum with women feeling they could not communicate with healthcare professionals as well as how the care they received differed from other women. The two second order constructs discussed are *'Judgemental attitudes'* and *'Inferiority complexes as a result of interactions.'*

3.17.1 Judgemental attitudes

In 13 studies healthcare professionals were perceived by women as making judgements about them (Davis & Bath, 2001, Wilton & Kaufmann, 2001, Herrel et al., 2004, Price & Mitchel, 2004, Spidsberg, 2007, Arthur et al., 2007, Reitmanova & Gustafson, 2008, Briscoe & Lavender, 2009, Cross-Sudworth et al., 2011, Shafiei & McLachlan 2012, Ward 2013, Robb et al., 2013, Mcleish & Redshaw, 2018). Judgements related to treatment preferences, level of family support, complex life situations, past history and sexual orientation. In the study by Ward (2013) that explored African American women with limited incomes experiences of maternity care, one woman felt labelled due to the biases of her midwife:

> *'I told [my midwife] I didn't like going to my appointments, and one day she just asked me, 'do you do crack?'… Just because I don't want to come to my appointments, I got to be a drug addict?'* (Ward, 2013, p. 1756).

A lesbian mother in Spidsberg's study (2007) reported how her GP disregarded and minimised her opinions following disclosure of her sexual orientation:

> *'He was a little, you know, 'my opinion is irrelevant' I expected more than this. I believed him to be more liberal and tolerant'* (Spidsberg, 2007, p. 483).

Wilton & Kaufmann (2001) also highlighted how lesbian women were made to feel different, reinforcing divides, which in turn made the women reluctant to share information with their health care professionals:

> *'When I first disclosed my relationship status with my GP, she was very disappointing. She stated outright that a woman should not consider childrearing unless married to a man; she was in fact quite rude.'* (Wilton & Kaufmann, 2001, p. 205).

With one of the lesbian women from this study being reported to social services due to judgemental beliefs held by her health care providers:

> *'[They] placed [my] child on [the] concern list! Because of the nature of our relationship, i.e. lesbians.'* (Wilton & Kaufmann, 2001, p. 205).

These judgemental attitudes were also highlighted in McLeish & Redshaw (2018) with one woman expressing the judgements made against her for choosing not to vaccinate her child:

> *'We didn't want to immunise the child because it is our belief, but they don't respect it…..[The health visitor] was talking about child protection, that she will contact [social services] and then it will be a problem'* (McLeish & Redshaw, 2018, p. 182)

Women also reported judgemental attitudes when extra support for language barriers was required. A Somalian woman in Davis & Bath's (2001) study stated:

> *'It's attitude, the doctors and nurses are in-experienced and they think, `Oh no, she doesn't speak English, what will we do?'* (Davis & Bath, 2001, p .242).

Two of the studies (Reitmanova & Gustafason 2008, Wilton & Kaufman 2001) highlighted issues where women felt offended by the prejudicial and presumptuous attitudes of staff. This could be in relation to women's religious choices, or their choice to wear a hijab whilst in labour. One woman reported how the midwife had said to her:

> *''Oh, why you are crying, you are beautiful. You don't need to cover yourself.'* (Reitmanova & Gustafason, 2008, p. 106).

3.17.2 Inferiority complex as a result of interactions

Women expressed a sense of inferiority due to the treatment by their healthcare professionals in 9 of the studies (Davies and Bath, 2001, Wilton & Kaufman, 2001, Ny et al., 2007, Spidsberg, 2007, Arthur et al., 2007, Shafiei & McLachlan, 2012, Robb et al., 2013, Howard 2015, Mcleish & Redshaw, 2018). Some women felt they were being watched and that their ability to mother was in question during appointments. One study identified how disrespectful interactions with her midwife resulted in one lesbian woman experiencing feelings of shame when the midwife expressed her disapproval of same sex relationships, thus denying the woman's right to freedom of sexuality without prejudice:

'The midwife said she had never heard of people like us. She wouldn't book me in; espoused her Christian beliefs' (Wilton & Kaufmann, 2001, p. 205).

Another woman from this study stated how these depersonalising interactions induced fear, and concerns for care provision:

'When she (the midwife) saw us in the waiting room, she'd roll her eyes and point at us to the receptionist. Because of my experience with her, I was frightened each time I met another midwife that she would hurt me or my baby because she didn't like lesbian' (Wilton & Kaufman, 2001, p. 209).

One woman in the study by McLeish & Redshaw (2018) expressed how poor care undermined her already fragile confidence due to previous negative life experiences and how these were seen to be exacerbated during negative interactions:

'I've had a lot of issues in the past with people telling me I'm not good enough….but that's exactly what they were doing, making you feel like you was not good enough' (McLeish & Redshaw, 2018, p. 181).

Healthcare professionals were reported to undermine women's knowledge and experience, which led to women feeling nervous and inferior in their presence. Robb et al (2012) study focused on teenage mothers' experiences of care stating how they felt labelled for being a young mother, and how they feared HCPs would remove their children from them as a result:

'Being young and they might contact social services if they are not coping and worry about getting their kids taken off them' (Robb et al., 2012, p. 1).

Some of the studies also reported how women would compare their care to others when observing how healthcare professionals interacted with other women:

'When I saw her with the other women in the hospital and she was so respectful: `What do you want to do', and `It's your baby?' Not like with me' (David & Bath, 2001, p. 243).

In contrast a lesbian woman in the study by Wilton & Kaufman (2001) expressed her feelings as positive when healthcare professionals took an interest in their reproductive journey as long as this was done sensitively and in a caring manner:

'People are curious and I don't mind being open – I hope it may broaden their experience and attitude.' (Wilton & Kaufman, 2001, p. 205).

3.18 Line of argument synthesis

The line of argument from this synthesis is *'I know my place'*. Women were, at times, aware of institutional inequality and judgements made against them when accessing maternity services and during interactions with maternity professionals. Women across the data set often felt they had no other option than to accept poor, inconsistent and dehumanising care, raising important ethical questions for maternity care providers. Women were conscious of their differences, reinforced through differential treatment, prejudiced attitudes, and lack of culturally contextual care when accessing maternity care in high income countries.

3.19 Summary of key findings

When comparing these findings to the wider literature on women's experiences of a negative traumatic birth, marked differences were apparent. In Elmir et al's (2010) meta-synthesis of women's experiences of birth trauma, she and colleagues highlighted; a lack of consent, poor information giving, lack of trusting relationships with care givers and how women experienced inhumane and degrading care as contributory factors to women experiencing birth trauma. Within this review, all these issues were apparent, however, there were key differences in regards to how disadvantaged and vulnerable women experienced varying levels of deservedness when accessing maternity services in high income countries. These findings suggest that women experienced poor care and varying levels of inequity and inequalities due to their complex life factors. Judgemental attitudes were seen to cause obvious barriers to engagement and the ability to build trustful relationships with healthcare providers. These experiences also had an impact upon women's levels of satisfaction with maternity services.

Refutational translation identified positive aspects of care where women would speak of kind, compassionate and engaging interpersonal interactions. Positive provider-woman relationships were characterised by women when they are feeling listened to, respected as an individual, and involved in care decisions.

3.20 Conclusion

This chapter has given a detailed description of the steps taken in undertaking a meta-ethnography. The chapter provides detailed insights into the experiences of

disadvantaged and vulnerable women when interacting with maternity healthcare professionals during antenatal and intrapartum care in high income countries. The third order themes to emerge were '*Depersonalisation 'No care in the care' 'Dehumanisation'* and '*Them and us'* and a line of argument synthesis '*I know my place'* captures the essence of disadvantaged women's experiences of maternity professionals in high income countries.

This review has provided the study with contextual findings on an international level when examining and exploring disadvantaged and vulnerable women's experiences of maternity care. Findings suggest that experiences of maternity care can be affected by attitudes of staff and during negative interpersonal interactions that consisted of judgemental, disrespectful and paternalistic attitudes. This interpretation fits within a critical social theory lens, in that these experiences are directly affected by our understanding of the world, shaped by social interactions and inequitable power relations when accessing maternity care. This raises many interesting questions of which will be further explored within this thesis and examined throughout the gathering of empirical data. This includes interviewing disadvantaged and vulnerable women about their negative birth experiences in a locality (The North West of England) and during the development and design phases of the educational programme.

CHAPTER 4: THEORETICAL CHAPTER

4.1 Introduction

In this chapter the theoretical approach for the research is introduced. A rationale for the selection of a critical approach to understanding phenomena within the research journey is presented in three sections. First, the epistemological and theoretical approach to research is discussed, followed by a discussion of my chosen epistemological position of social constructionism. The decision to adopt an interpretive (qualitative) approach as opposed to positivistic (quantitative) approach to research methods is explained. As a final point, different interpretive approaches are considered within the study with a justification given to why a critical theoretical approach was chosen.

The second section in this chapter presents the philosophical underpinnings of critical theory acknowledging relevant influential inter-disciplinary thinkers. This includes the work and influence of philosophers including Karl Marx, Antonio Gramsci, Theodor Adorno and the innovatory intellectual turn furnished by the likes of Horkheimer and Habermas in the influential Frankfurt School. This section is not intended to provide an in-depth summary of the lifeworks of such philosophers, but to provide a summary of theories associated with their work which have relevance to this study. A rationale for the selection and the utilisation of the chosen approaches is presented within this section.

The third section provides an overview of the works of Paulo Freire, a seminal thinker within critical theory, including the relevance of his work within the context of this study. This will include discussing and presenting critical pedagogies as the chosen approach to the design of the educational programme. Lastly, I link Freire's approach to designing and delivering critical pedagogies to the wider study and my chosen theoretical positioning. I conclude with a reflexive stance on engaging with theory and critical philosophical insights to identify an appropriate approach within this study.

4.2 SECTION 1

4.2.1 Epistemological and ontological perspectives.

Ontology, or as Aristotle termed *'The first philosophy'* is in principle concerned with revealing the essence of things (De Rijk, 2002). Ontology situates itself within the science or study of being, whereas epistemology is concerned with the nature of reality; that

which constitutes the nature, scope, possibilities and limits of human knowledge (Goldman, 2004). Ontology could be summarised as what we believe about our social reality, including the claims and assumptions about existence, how this is perceived, and what factors may influence these beliefs (Blaikie, 2009). Many theorists would argue that ontology should be the starting point of all research inquiry as methodologies and theoretical beliefs unsurprisingly follow the ontological position (Grix, 2002). Crotty argues that each theoretical perspective should encapsulate the way in which one understands *'What is'* (ontology) alongside understanding *'what it means to know'* (epistemology) (Crotty, 1998, p. 10).

A research paradigm contains three elements: ontology, epistemology and methodology (Crotty, 1998), these elements are then justified by the methods chosen by the researcher during empirical stages. It is therefore important that a researcher understands their ontological and epistemological positioning before empirical data collection begins (Crotty, 1998). It is argued that an epistemological stance needs to be identified to ensure methods of inquiry are consistent with the chosen research question to be explored (Carter et al., 2007). Denzin & Lincoln (1994) highlight the importance of the researcher remaining aware of possible influences upon their chosen paradigm, acknowledging the value of personal interpretation upon the phenomenon to be explored. As a researcher, I recognise my own experiences as a midwife, mother and researcher and how these experiences guide and influence my ontological and epistemological beliefs. As a midwife researcher aiming to explore and understand the lived experience of a traumatic birth amongst disadvantaged and vulnerable women, I was guided by the aims of this research to consider social constructionism as a theoretical perspective to direct the methodology and methods adopted within this study.

4.2.2 Social constructionism

Constructivists believe that individuals mentally construct their experiences through discursive processes, whereas social constructionism has a social rather than an individual focus (Young & Colin, 2004). Social constructionism derives from the work of Karl Mannheim (1893-1947) and Berger and Luckmanns (1972) *'Social construction of reality'* and is probably best seen as a broadly encompassing perspective, with soft and hard poles corresponding to the degree to which reality is believed to be constructed.

From a social constructionist viewpoint, the belief is that human beings construct meanings through interactions with the world and those within it (Heidegger, 1927). A social constructionist approach understands that social practices, relationships and institutional customs, principles and practices have a variable level of impact upon the construction of knowledge, dialogue and language (Bourdieu, 1984, Bauman, 2000, Freire, 2018), believing that meanings credited to objects arise from interactions and associations, as a result, meanings are both objective and subjective in nature. Knowledge is seen as formed through the medium of dialogue, which is interpreted to create meanings, which are then also open to interpretation. These interpretations are context sensitive, meaning they are dependent upon environments, conditions and time (Lincoln & Guba, 1985). Framed within this study, exploring disadvantaged and vulnerable women's accounts of a traumatic birth requires research to address not only the lived experience of a phenomena (birth trauma), but also the context and dialogue within which it occurred (birth environment / health care professionals).

4.2.3 Addressing issues of power through a social constructionist lens

Although no explicit concept of power is established in Berger & Luckmans (1957) '*The Social Construction of Reality*' several theorists consider the phenomenon of power as theoretically relevant, outlining how a theory of power can be developed from this vantage point of social theory (Dreher, 2016). For example, Burr believes that the phenomenon of enculturation[10] in a society is ultimately linked to power, in that it tends to be the powerful who are more successful at having their version of events predominate (Burr, 1995). To deepen this understanding, I had to first grasp what macro level influences social constructionists place at the centre of an analysis, with a specific focus on maternity care. I draw upon the work of Foucault when attempting to understand power in the context of healthcare. Foucault (1980) believes that knowledge is indivisible from power, and the (meta) narrative of medicine (a powerful way of knowing) predominates and squeezes out alternative ways of making sense of the world (or at least health and health services). For positivists, it is possible to conceive of illness and its treatment independently of considering the person afflicted by the illness, and

[10] Enculturation is the process by which people learn the dynamics of their surrounding culture and acquire values, norms and worldviews appropriate or necessary in that culture. If successful, enculturation results in competence in the language, values, and rituals of a given culture (Grusec & Hastings, 2014).

all their subjectivities (Wilson, 2000). In contrast, Conrad & Barker (2010) oppose this stating that there is a case to view illness as a social construction:

> *'Some illnesses are particularly embedded with cultural meaning—which is not directly derived from the nature of the condition—that shapes how society responds to those afflicted and influences the experience of that illness. Second, all illnesses are socially constructed at the experiential level, based on how individuals come to understand and live with their illness. Third, medical knowledge about illness and disease is not necessarily given by nature but is constructed and developed by claims-makers and interested parties'* (Conrad & Barker, 2010, p. 67).

Berger and Luckmann (1957) highlight that although reality is socially defined, it is individuals and groups of individuals who define it, live it, and ultimately, are able to change it. Newnham (2014) places this theory in the context of midwifery stating that medical fraternity has successfully laid claim to birthing power/ knowledge resulting in a *'dominant discourse'* in relation to maternity care, resulting in oppressive experiences of care. This approach ties in with Foucault's beliefs regarding the social control/governance function of healthcare enforced through numerous and diverse techniques, aiming to subjugate bodies and control populations (Foucault, 1976). These theories of power in relation to healthcare / maternity spoke to my inquisitiveness regarding the impact power has on the experiences and accounts of disadvantaged and vulnerable women accessing maternity care. As situated in chapter two, a social focus on the experiences of these women accessing maternity care requires a consideration of issues of power to illuminate and address not only the social control and governance function of an institution (the NHS), but also the particular social control and governance of disadvantaged and vulnerable women.

Burr (1995) illuminates the difficulties in challenging and addressing power, stating that the powerful will always attempt to present themselves and their version of events in such a way that it will prevail over other versions, highlighting the limits to potential emancipation within hierarchical structures built on extant power relations. Conversely, as reality is subjectively perceived, it is important to examine the cultural and social context within which inequitable power relations are at play; in this case within the social space of birth.

It is felt that this approach to understanding phenomena within this study will facilitate a deeper theoretical understanding of power relations within the birth environment and the impact upon disadvantaged and vulnerable women's experiences of birth. As this study adopts a social focus, aimed at exploring the lived reality of birth trauma in the social space of birth, an interpretivist approach to research inquiry was deemed most suitable.

4.2.4 Interpretivist approaches to research inquiry

A turn to the social aspect of research inquiry within sociological theories was inspired by key philosophers in the early 19th century including Emile Durkheim, Max Weber and Karl Marx who to a greater or lesser degree, were responsible for shifting study of the social world away from natural scientific methods of research inquiry (Hughes & Sharrock, 2016). They believed that the social world cannot be investigated and understood using the same methods of investigation as those used in the natural sciences (May & Williams, 2002).

The two key approaches to research inquiry are positivism and interpretivism. Within a paradigm of positivism the role of the researcher is limited to data collection and interpretation through an objective approach, with research findings usually being observable and quantifiable (Wilson, 2000). Positivist researchers favour quantitative data and experimental methods to allow for careful measurement of a phenomenon. These methods of data collection are deductive and usually begin with a hypothesis to allow for replication in methods (Prasad, 2017). A positivist approach to understanding human behaviours is criticised by interpretivists who believe that we do not react to external factors and social forces in a predictable way (Creswell & Poth, 2017). Interpretivists consider that to understand human behaviour, the researcher needs to view the world or experience through the individual's eyes (Schwartz-Shea & Yanow, 2013), therefore investigation of the social realm requires a different epistemology. Interpretivists also recognise the impact of their own background and experiences when aiming to understand '*the world of human experience*' (Cohen & Manion, 1994, p. 36).

To adopt an interpretivist approach within this study requires me to gain a deeper understating of the social processes involved in how people experience phenomena

(Creswell & Creswell, 2017), in this instance disadvantaged and vulnerable women during birth.

4.2.5 Otherness

Social constructionist theories also open up the consideration of othering as discussed in chapter two. Othering offers an explanation of the ways in which disadvantaged and vulnerable women's social realities are constructed through dialogue and narrative. The idea of '*otherness*' is fundamental to contemporary sociological analyses of how majority and minority identities are constructed in a society and how associated experiences can be understood (Henriques et al., 1984, Sampson, 1993). Zygmunt Bauman, an influential critical thinker, believes otherness is central to the way in which societies establish identity categories and offers insight into how a sociological process of labelling individuals with direct regard to difference, is ultimately related to power:

> *'In dichotomies crucial for the practice and the vision of social order, the differentiating power hides as a rule behind one of the members of the opposition. The second member is but the other of the first, the opposite (degraded, suppressed, exiled) side of the first and its creation. Thus, abnormality is the other of the norm…. woman the other of man, stranger the other of the native, enemy the other of friend, 'them' the other of 'us'* (Bauman, 1991, p. 8).

Bauman recognises gender as an aspect of difference that is highlighted in ascribing otherness, in that the power of man over women relates back to the power over another but need not directly be related to patriarchy. Okolie (2003) recognises how social identities and power are interlinked:

> *'Social identities are relational; groups typically define themselves in relation to others. This is because identity has little meaning without the 'Other'. These definitions of self and others have purposes and consequences. They are tied to rewards and punishment. Power is implicated here, and because groups do not have equal powers to define both self and the other, the consequences reflect these power differentials.'* (Okolie, 2003, p. 2).

Feminists and other scholars have also contributed to this body of knowledge, with reference to the social representations of women (under patriarchy) with a contemplation of how simple matters of identity may be complicated by notions of

intersectionality. Patricia Hill Collins' theory of intersectionality gained prominence in the 1990s proposing that cultural patterns of oppression are not only interrelated but are bound together and influenced by the intersection of factors such as gender, class, and ethnicity (Collins, 2016). This added a new dimension to critiquing power and how influential contributory attributes become associated with differential social outcomes, including discrimination amidst varying levels of meaning and experience.

Although it is vital to acknowledge intersectional factors that impact upon women's experiences in maternity care, less attention is paid within these theories with deconstructing systems of power to understand how women arrive at a point of experiencing a traumatic birth. Within this study an exploration of power using a critical theoretical lens would allow me to explore the birthing space and the interactions that take place within it, aiming to identify key triggers for a traumatic birth experience amongst those who may be othered in maternity care.

4.2.6 Social abjection

Flowing from considering the notion of otherness within this study, the concept of abjection allows us to explore the means by which self-other constructions operate to demonise marginal groups and defend the esteem of, often similarly disadvantaged, in-groups. Such theories are arguably highly relevant to my exploration of the subjective experiences of birth in today's NHS. In her 2013 book *'Social Abjection and Resistance in Neoliberal Britain 'Revolting Subjects'* (Tyler, 2013), Imogen Tyler describes how power is constituted through forms of '*Inclusive exclusion'* fuelled by aesthetic political strategies aimed at stigmatizing, marginalizing and promoting social exclusion and injustice within a society. Tyler describes social abjection in terms of a theoretical resource which discusses states of exclusion from multiple perspectives, particularly from the perspective of those groups and populations who are subject to its violent and stigmatising effects. This revised notion of abjection is political, social and historical in orientation, theorising abjection as a social force (a cultural, political economy of disgust) which operates on numerous scales as a method and practice of governmentality, binding together societies and states through inclusive forms of exclusion. (Tyler, 2013). This approach offers a lens through which to examine disadvantaged and vulnerable womens' experiences of maternity care through questioning how states of being (human life) and states of belonging (political life) are

made and unmade and how we may critically participate in this practice of making and unmaking within healthcare settings (Butler and Spivak, 2007). This includes an acknowledgment and understanding of neoliberal ideologies and the influence this political ideology has on society (Macrine, 2016).

When adopting a critical lens to understand the lived experience of birth trauma for disadvantaged and vulnerable women, acknowledging the influence of neo-liberal ideologies upon collective social understandings is important to contextualise the impact of this ideology upon publicly run institutions, namely the NHS. Tyler's work highlights existing labels such as *'Revolting Subjects'* and *'Chav Mum Chav Scum'* to illustrate how neo-liberal ideologies, expressed through the media, have rendered abject the socially disadvantaged and vulnerable within society. Furthermore, these abject groups are viewed as draining the economic resources of the country, creating class disgust towards those marginalised on political, social and economic levels, and hence dehumanised (Tyler, 2008, Tyler, 2013).

Lewis et al (2018) believe that these messages, delivered through popular and unregulated means, disproportionately influence public opinion regarding the operation of the NHS and welfare system, replete with notions of perceived deservedness. A critical approach to understanding such phenomena could illuminate the impact this ideology has upon NHS staff attempting to deliver equitable care. Speed & Mannion (2017) note that political influences and public opinion are key factors that impact upon care delivery in the NHS, acknowledging the impact austerity measures and current right-wing rhetoric has had on the public's perception of healthcare. Rather than adopting an observational stance to understand disadvantaged and vulnerable women's experiences of maternity care, this approach provides me with a starting point from which to identify forces within maternity care that strip women of their right to a positive birth experience.

I now draw of the theories of Miranda Fricker to further explore the issues of abjection in a maternity context, focused on epistemic and hermeneutical injustices, offering a lens through with to explore the phenomena of traumatic experiences of birth.

4.2.7 Testimonial & hermeneutic injustice and power

Miranda Fricker's work on *'Epistemic injustice'* offers a lens through which to examine how social agents aim to make sense of their social experiences, providing an interesting standpoint from which to examine birth trauma amongst disadvantaged and vulnerable women. (Fricker, 2009). Fricker discusses *'Hermeneutical injustice'* and *'Testimonial injustice'* as two forms of understanding epistemological dilemmas. '*Hermeneutical injustice'*, wherein someone has a significant area of their social experience obscured from understanding, owing to prejudicial flaws in shared resources for social interpretation (Fricker, 2009). With '*testimonial injustice'* occurring when prejudice causes a hearer to give a deflated level of credibility to a speaker's word (Fricker, 2009). Fricker's work strengthens the theoretical underpinnings of this research in the context of healthcare as her beliefs on epistemic injustice can be adapted and applied to critical arguments that place power at the epicentre of their deconstruction. Fricker's argument enables me to contextualize the study's focus on a macrosociological[11] level examining power injustices that flow from the dominant knowledges of the healthcare system, the episteme, that may then impact upon microsociological[12] level relations between women and their midwife.

Fricker's work illuminates' considerations of the interpretation of a lived experience resulting from prejudicial flaws in the understanding of *'what makes a birth traumatic'.* As a concept, testimonial injustice can be used to examine the biases held by those in power that may influence and impact upon interpersonal interactions during birth. This can be through paternalistic interactions, ineffective communication strategies, language barriers, biases and iniquitous power relations. These practices can present an unbalanced power interplay between women and their midwives in which women's voices are silenced through the exertion of power. Fricker's notion of hermeneutical injustice could illuminate flaws in the interpretive resources available to women, that operate in:

[11] Macrosociology; allows observation of large-scale patterns and trends but runs the risk of seeing these trends as abstract entities that exist outside of the individuals who enact them on the ground.
[12] Microsociology; allows for this on-the-ground analysis but can fail to consider the larger forces that influence individual behaviour.

> *'Preventing the subject from making sense of an experience which it is strongly in her interests to render intelligible'* (Fricker, 2009, p. 7).

Adopting Fricker's stance on hermeneutical injustice allows for an examination of possible collective social understandings held within maternity care that could be argued to result from prejudicial biases held by those in power, i.e. midwives as subsidiary to medical practitioners. What Fricker's theory also provides the study with is an acknowledgment of emancipatory strategies concerned with consciousness raising about where power lies, whose interests are served, and the promotion of alternative knowledge.

Joining both hermeneutical injustice and social abjection within a critical theoretical positioning provides a lens through which to examine disadvantaged and vulnerable women's experiences within a publicly run institution such as the NHS, created and delivered within a political state and society under a prevailing neoliberal ideology. This allows for an analysis of power and domination, conscious and unconscious, that may provide the study with an explanation for how these factors impact upon women's experiences of traumatic birth.

4.3 SECTION 2

4.3.1 Introduction

Building upon my chosen social constructionist foundations, I move now to justify a critical social theoretical standpoint that supports progressive critique of the aforementioned iniquitous power relations and, importantly, points to potentially emancipatory action. Karl Marx's contribution to understanding society contributed to me vindicating this critical social theory standpoint, informed by neo-Marxist writings, typified by the Frankfurt School, who synthesise classical Marxism with newer and broader theoretical perspectives. Marx's understanding of the human endeavour in a society laid claim to the idea that social inquiry (research) ought to be concerned with the value of knowledge for changing the world, not just understanding it (Marx, 1968). In his works '*Eleven Theses on Feuerbach'* Marx stated that philosophers have only interpreted the world, yet the point of research should be to change it (Marx, 1968). Despite often being associated with a more structuralist standpoint, Marxist affinities for constructionist ideas were arguably forged in his seminal works, that examined

human consciousness in social relations. In his work '*A Contribution to the Critique of Political Economy*' Marx states:

> *'It is not the consciousness of men that determines their existence but their social existence that determines their consciousness'* (Marx, 1904, p .41).

Subsequently, Erich Fromm of the Frankfurt School built upon the early works of Marx, bringing in newer psychosocial theorising. Fromm sought to re-emphasise the ideal of freedom. He became one of the founders of socialist humanism, promoting the early writings of Marx and his humanist messages to the US and Western European public regarding the power of a collective consciousness to break down the dehumanising aspects of society (Fromm, 1967). The work of the inter-disciplinary Frankfurt School also included notable contributions from others such as Max Horkheimer and Jurgen Habermas, forms the theoretical basis for a critical social theory in which emancipation, alongside an examination of human relations, is placed at the centre of research endeavours and analysis (Horkheimer, 1982, Habbermas, 1987a).

4.3.2 Critical social theory

As a researcher working within an area concerned with power, agency and equity (healthcare, namely the NHS), a critical lens to underpin my study provides me with the foundations upon which to analyse, present and deliver my findings (Horkheimer, 1937, Fromm, 1967, Gramsci, 1971, Horkheimer & Adorno 1972, Habermas, 1987b, Adorno, 2005). Critical theory stresses the importance of critiquing society and culture through reflection and applying knowledge from the humanities and the social sciences. Along with the aforementioned Fromm, key thinkers include Herbert Marcuse, Theodor Adorno, Max Horkheimer, Walter Benjamin, Axel Honneth and Claus Off who made up various waves of Frankfurt School theoreticians. Critical theory has also been influenced by other key thinkers not directly linked to the Frankfurt School, including György Lukács and Antonio Gramsci, as well as the second generation Frankfurt School scholars, notably Jürgen Habermas. The theorist Max Horkheimer described a theory as critical insofar as it seeks:

> '*To liberate human beings from the circumstances that enslave them*'. (Horkheimer 1937, p. 244).

Critical social theory was born from neo-Marxist scholars of the Frankfurt school in the 1930's, inspired both by the optimism of classical Marxism[13] and pessimism regarding failings of grand-scale Marxist praxis, notably the collapse of the Soviet revolution into totalitarian dominance. According to Frankfurt theorists a critical theory differs from a traditional theory according to its explicit concrete purpose. Critical theorists examined the influence consumerism and popular culture had upon society, seeking new models for political change and emancipation from capitalist oppression (Marcuse, 1969, Horkheimer & Adorno 1972, Fromm, 1985). Whilst not located in the Frankfurt School, nor necessarily inspired within a Marxist tradition, other contemporary theories arguably merit a *'critical'* appellation offering a critique of society, popular culture, educational systems and healthcare (Bourdieu, 1984, Foucault, 1995[14], Benhabib, 2002). These theorists accept that whilst social influences impact upon nature and human theories, it is humans that can ultimately alter society through their actions. Critical theory emphasises the need for people to understand their social, cultural and historical situations to be able to engage in emancipatory practices.

In his seminal work '*Prison Notebooks'*, Gramsci (1971) states that people need to be consciously aware of the limits of freedom before they can set themselves free. Critiques of critical theory believe this to be a purely rhetorical theory that supports action, but places little emphasis on practical action, or more specific '*praxis*'[15] (McNiff, 2013, p.50). Carr and Kemmis describe praxis as more complex than purely action based upon reflection, but that it symbolises a pledge to human emancipation, respect for others and the pursuit of truth (Carr & Kemmmis, 2003). From an educational perspective Freire (1984) believed that critical theories of emancipation should be adopted as a process of constant reflection and action that ultimately lead to praxis. Freire believes that action without reflection is *'activism'* and that reflection without action is *'subjectivism'* stating:

[13] Marxism - the economic and political theories of Karl Marx and Friedrich Engels that hold that human actions and institutions are economically determined, and that class struggle is needed to create historical change.
[14] Foucault notably engaged in critical debate with Habermas a key Frankfurt School scholar, yet arguably they shared a substantial amount of common theoretical ground. Foucauldian accounts of power and governmentality are undeniably critical, if not immediately concerned with demonstrating paths to emancipation (Kelly 1994, McCarthy, 1994, Outhwaite, 1996).
[15] Praxis is the process in which a theory, lesson, or skill is enacted, embodied, or realized.

> *"It is only when the oppressed find the oppressor out and become involved in the organized struggle for liberation that they begin to believe in themselves. This discovery cannot be purely intellectual but must involve action; nor can it be limited to mere activism but must include serious reflection: only then will it be a praxis"* (Freire, 1972, p. 65).

This first requires a look at cultural understandings of a society from a critical perspective. As discussed, the practical aim of recognising and overcoming the conditions that limit human freedom within a critical theory must engage and acknowledge the cultural, psychological, social and institutional forms of domination. Recognising the cultural conditions and social norms of disadvantaged and vulnerable women and how these combined factors impact upon their psycho-social wellbeing will ensure these premises are implemented when adopting a critical theoretical lens to the design of an educational programme.

I now present a discussion on the cultural understandings of a society, using the theory of hegemony to frame the study within a critical lens.

4.3.3 Hegemony

Critical traditionalists are suspicious of the constructed meaning of '*culture*' when using this approach to examine social experiences. They believe that constructed meanings have been formed over time through *'sedimentation'*[16] which, within a critical lens, aims only to impose *'hegemony'*[17] within society (Giroux, 2018). Gramsci believed that this hegemonic ideology is linked to the economic *'base'* of society, such that those that own the means of production have the power to affect consciousness within that society to control the masses (Gramsci, 1971), explaining how consent is artificially sustained through social and cultural practices including media, religion and education (Giroux, 2018). Gramsci produced new theories and interpretations of power and cultural

[16] Berger & Luckmann (1972) believe Institutions are a product of the historicity and need to control human habitualization (repeated behaviours or patterns). The shared nature of these experiences and their commonplace occurrence results in sedimentation, meaning they lose their memorability.

[17] Hegemony derives from a Greek term that translates simply as 'dominance over' and that was used to describe relations between city-states. Its use in political analysis was somewhat limited until its intensive discussion by the Italian politician and philosopher Antonio Gramsci.

hegemony[18] in relation to the role education could play in the struggle for emancipatory change.

Tyler's theorising of social representations and, specifically, abjection offers insights to understanding the impact hegemonic ideals have upon disadvantaged and vulnerable groups in society. She argues that hegemonic political discourses promote the idea of a denigrated *'other'* making them handy scapegoats for social ills that they are not actually responsible for (Tyler, 2013). This can be seen to create a misunderstanding within society regarding the cultural variations and hardship people face in their lives, impacting upon how they are perceived and engaged with in society. Giroux (1981) argues that in order for critical theorists to understand the idea of a politicized notion of culture and its impact upon the disadvantaged and vulnerable in a society, the idea must first be defined in relation to where power resides, offering a more nuanced analysis than a mere consideration of class and power alone:

> *"Culture would be defined in terms of its functional relationship to the dominant social formations and power relations in society…… Issues regarding gender and ethnicity, as well as the dynamics of nature, cannot be framed exclusively within class definitions and the link between power and culture cannot be reduced to a simple reflex of the logic of capital,"* (Giroux, 1981, p. 26).

This interesting standpoint can be used to examine the notions of power within a maternity context for disadvantaged and vulnerable women, ensuring that not only are power relations between women and their care providers examined, but also the beliefs and attitudes held by maternity care professionals and how these may contribute to professionally deficient interactions, causing situations of birth trauma for these women.

Gramsci emphasises the role theories of domination and power can play in constructive action in a society (Gramsci, 1971, 2009). Harden (1996) approaches Gramsci's ideas from a practical stance, stating there is a need for professionals to seek emancipation from oppressive power relations within the healthcare system to deliver humanistic

[18] Cultural hegemony is a domination over society by the ruling class who are seen to manipulate the culture of that society. This includes a manipulation of peoples beliefs, perceptions, values, and morals so that the ruling-class worldview becomes the accepted cultural norm (Adamson, 1983).

care. He calls on healthcare professionals to engage in '*The pursuit of radical change'* through a continuous questioning and critiquing of unacceptable conditions in which certain people and groups in our society are forced to live (Harden, 1996, p. 36). Similarly, Clare (1993) urges healthcare professionals to question dominance of specific types of information and values to fully understand that education is a political act and that knowledge is inextricably related to power.

Thus, through engaging with key authors in critical theory my understanding of society, healthcare systems and educational processes relevant to this study has been influenced by the works of Gramsci (1971), Freire (1972) and Giroux (1988) in particular. Their guides for human emancipation and the application of critical theory within educational concepts were deemed most applicable to my study allowing me to question established social orders, institutions, ideologies and dominating practices in maternity care.

4.3.4 Counter Hegemony

In attempting to address these issues within the study, I turn now to Gramsci's notion of '*counter hegemony'.* Counter-hegemony refers to the attempt by humans to critique or dismantle hegemonic power (Carroll, 2009), encouraging confrontation and/or opposition to an existing status quo and its legitimacy, offering in the context of this study an approach to questioning current practices that may result in traumatic experiences for women. This theory proposes that liberation through education requires a critical consciousness to be raised by intellectuals who are involved in educational forms of emancipation. Gramsci believes that for this vison to be realised mass participation is required, as is a facilitative approach in which intellectuals work with people to emancipate them rather than working in their name (Gramsci, 1971).

This proposes a difficult dilemma for academics who by virtue of their professional situation may be seen to occupy an expert level of social positioning when embarking upon emancipatory research endeavours. Critical approaches to education require an active exploration of social realities that dominate and lead to constraints in human decision making, placing a subject (e.g. disadvantaged and vulnerable women) in the wider cultural, economic and political context (healthcare institutions such as hospitals) to facilitate liberation (Harrington, 2005). Drawing on these concepts within this study, the application of these theories during the design phase of a tailored educational programme for maternity professionals would allow for an exploration of the social

realties amongst disadvantaged and vulnerable women during birth. Thus, this would explore how these realities may impact upon perceptions of birth and interpersonal interactions that cause situations of trauma.

A critical social theory-based movement illuminates the relationship between social systems and people within it and how they produce and reproduce each other. A critical social theory provides the descriptive and normative basis for this research inquiry by focusing on decreasing dominance and increasing freedom for women in maternity care. Critical social theories are also pragmatic, in the sense they aim to combine rather than separate the poles of philosophy and the social sciences i.e. explanation and understanding, structure and agency, regularity and normativity (Bohman, 2005). Adopting a pragmatic approach to the application of critical social theory within this study will allow me to hold to emancipatory ideals at the same time as being aware of constraining forces likely to impede progress.

4.3.5 From Gramsci to Freire: A critical pedagogical focus for midwifery education

Gramsci was interested in the role of intellectuals in supporting processes of emancipation through the acknowledgment and delivery of a counter hegemony. He defined two distinct categories, traditional and organic (Gramsci, 2005). Traditional intellectuals are people who believe themselves to be autonomous from the governing social groups. He believed that these beliefs were often unfounded and, rather, they generally served the needs of the ruling class (Gramsci, 2005). In contrast, organic intellectuals originate from the community and are not primarily professionals working in exclusive structures such as universities, healthcare systems or religious organisations (Gramsci, 2009). Gramsci argues that organic intellectuals can foster critical consciousness throughout the community producing counter hegemonic ideas amongst the oppressed within society (Gramsci, 2005). The ideal of the organic intellectual as a facilitator for change represents the ideal form of the community worker, working alongside those whom they wish to emancipate. Difficulties arise for academics who identify with those that they strive to emancipate yet hold positions of power through their work.

Cresswell & Spandler (2013) highlight the contradictions faced by researchers when assuming the identity of a critically engaged academic allied to the oppressed within a

society, yet possessing the privilege of a university role. Within this study, believing I am aligned with disadvantaged and vulnerable women through my own experiences, I prefer to adopt the identity of an organic intellectual. However, this is largely unsustainable given the fact that as a midwife and a PhD researcher I could be viewed as a figure of authority by women, hence the notion of being a critically engaged academic is appealing, acknowledging that my reflexive subjectivity and personal experiences can guard against some of the more obvious contradictions and pitfalls when undertaking research. Conversely, Levinson believes that those who strive to emancipate need not fall neatly into either one or the other category as their purpose is not individual advancement but human emancipation (Levinson et al., 2015).

Within section three below, my thoughts and understandings of Gramsci's theories of education and the role of the engaged intellectual in delivering emancipatory education are further developed using the works of Paulo Freire.

4.4 SECTION 3

4.4.1 Introduction

The previous sections have laid the foundations for a critical theoretical stance to be adopted within this study. Drawing upon the theories of Gramsci detailed in the previous section, I now present a detailed discussion on the application of critical theory in education focused on the philosophy of education known as critical pedagogy. I draw upon the works of Paulo Freire as the leading scholar in critical pedagogy citing his seminal work *'Pedagogy of the oppressed'* as an underpinning approach to the design of an immersive educational programme

4.4.2 Paulo Freire

Critical theorists have widely credited Paulo Freire (1921-1997) for the first applications of critical theory towards education and wider social emancipation stating with the medium of learning. A Brazilian educator, philosopher, public intellectual and activist, Freire was a leading advocate for critical pedagogy, best known for his influential work '*Pedagogy of the oppressed'* which was first translated into English in 1972 following its release in 1968 (Freire, 1972). This seminal work captivates by relating critical theory to democratised, participatory educational approaches, in the spirit of the Frankfurt School, without necessarily acknowledging direct influence. Freire's approach explicitly aimed to facilitate and liberate oppressed groups in a society through emancipatory,

dialogic education. Mayo (1999) captures the essence of Freire's beliefs on the role education plays in the emancipation of vulnerable groups stating that:

> *'There is no such thing as a neutral education process. Education either functions as an instrument which is used to facilitate the integration of generations into the logic of the present system and bring about conformity to it, or it becomes the 'practice of freedom', the means by which men and women deal critically with reality and discover how to participate in the transformation of their world.'* (Mayo, 1999, p. 5).

4.4.3 Critical pedagogies

Critical pedagogy is a philosophy of education and social movement that has established and practically applied concepts from critical theory and related traditions into the realm of education (Kincheole et al., 2011). Critical pedagogy is a dialectical process that examines conventions and statements for accuracy as part of an ongoing questioning of political and historical claims, a process that can lead to social justice action by teachers (McLaren, 2015). Freire acknowledged that education cannot be separated from politics believing that teaching and learning are political acts in themselves, describing this connection as the main principle of critical pedagogy (Freire, 1993). He believed that within a critical pedagogy facilitators and participants must be made aware of the political influences that impact upon social structures and power relations that impact the way education is enacted (Freire, 1972). Critical education in seen as having a political focus, believing an awareness and reflection of the political beliefs and ideologies held by teachers is required to ensure these notions can be challenged and discussed to encourage critical consciousness. Indeed, adopting such a stance sees the roles of the teacher and the taught blur in a process of mutual learning that is as much led by the learners as any facilitator of the process (Ledwith, 2007).

Darder & Baltodano (2003) believe that the development and delivery of critical pedagogies must include an awareness of and where possible an inclusion of the following aspects:

- Political economy of schooling and the concept of class
- Historical context of knowledge construction
- Critiques of ideologies

• Dialogue and development of critical consciousness

• Hegemony as *'A process of social control that is carried out through the moral and intellectual leadership of a dominant sociocultural class over subordinate groups'* (Darder & Torres, 2003, p. 13).

• Counter-hegemony as *'Moments of resistance, through establishing alternative structures and practices that democratize relations of power, in the interest of liberatory possibilities'* (Darder & Torres, 2003, p. 14).

Critical pedagogies that follow these principles acknowledge Horkheimer's three distinct points required in delivering a critical pedagogy; it must be explanatory, practical, and normative, all at the same time. That is, it must explain what is wrong with the current social reality to be addressed, identify the actors to change it, and provide both clear norms for criticism and achievable practical goals for social transformation:

> *'A critical pedagogy has as its object human beings as producers of their own historical form of life'* (Horkeimer, 1993, p. 21).

Giroux states that Freire's concept of knowledge as a liberating tool, provides educators with a framework upon which such ideas could be employed to enrich educational theory and practice for emancipation (Giroux, 2018). This study aims to adopt such a premise in the design of an educational programme for maternity professionals, aiming to explore the birthing space, examining potential power issues and oppressive interpersonal interactions that may cause situation of trauma amongst disadvantaged and vulnerable women during birth. Freire believes that knowledge is fundamentally linked to the questioning of social relationships and hints at the forms of transformed relationships of care that this study aims to achieve:

> *'The critical pursuit of knowledge has to be paralleled by a quest for mutual humanization among those engaged in such a pursuit. A radical pedagogy requires non-authoritarian social relationships that support dialogue and communication as indispensable for questioning the meaning and nature of knowledge and peeling away the hidden structures of reality'* (Friere, 1993, p. 7).

A critical pedagogy within this study would aim to peel away the hidden structures of the maternity care system that create the nature of reality for disadvantaged and

vulnerable women who experience a traumatic birth, questioning how these structures impact upon their experience and treatment. The aim here would be to use a critical pedagogical approach to engage participants (i.e. maternity professionals) in the pursuit of liberating both themselves and disadvantaged and vulnerable women during birth, through the development of critical consciousness; a Freirean notion not dissimilar from Gramsci's concept of counter-hegemony.

On a cautionary note, Freire discusses the requirement of consciousness amongst oppressors and oppressed and its relation to liberation. Freire (1972) argues that if a person's consciousness is sleeping, their world is sleeping at the same time. In other words, if the oppressors are deeply rooted in the consciousness of the oppressed, it is impossible to remove oppression. Hence, the practice of freedom necessitates people to engage in an historical, cultural and political psychoanalysis of society and its structures to decode freedom limiting situations (Glass, 2001). As Giroux remarks, this is an important point in Freire's work and indicates the ways in which domination is subjectively experienced through its internalization and sedimentation in the very needs of one's personality (Giroux, 1988). What is at work here in Freire's thought is an important attempt to examine the subconscious repressive aspects of domination and the possible internal barriers to self-knowledge and self-emancipation. Appreciation of this important psychosocial dimension and wider democratic, participatory pedagogical practices will inform the design and implementation of the educational programme within this study. Such internalised aspects of dominance for the purpose of this study will include the intertwining of both neoliberalism and bio-medicine with tropes of otherness and abjection.

As positioned in section one, adopting a constructionist view point, requires me to ensure that the programme illuminates core constructs of experiences, in this case, birth trauma for disadvantaged and vulnerable women. This study aims to address components involved in these experiences by illuminating salient critical factors within the birthing environment during the design and delivery of the finalised educational programme. This section concludes that at the core of critical interventions there must be a practice of liberation, which begins with the educational process of cultivating people's critical consciousness aiming to change the world (Freire, 1972). For Freire, this practice of liberation is known as '*Conscientization'.*

4.4.4 Critical consciousness 'conscientization'

It has been identified within the previous section that a critical pedagogy aims to question everyday life, identify contradictions within it and make critical connections within the structures of society that oppress. In Freire's seminal work *'Pedagogy of The Oppressed'*, he aimed to illuminate unjust structures of society that discriminate against the least powerful in a society, to create a more just and equal world through the analysis of power. The idea of *'conscientization'* sets the context for questioning the lived reality of birth trauma for disadvantaged and vulnerable women by critiquing the acceptance or taken for granted oppressive aspects of everyday life (Freire, 1972). Freire believes that looking upon situations from a distance allows for the adoption of a more reflective analysis to why situations are the way they are (Freire, 1972). Habermas agrees, believing we must pave the way for a communicative, rather than instrumental, rationality in which those seeking an understanding of a phenomenon:

> '*Find a place for the subject within acts of communication that construct subjects as well as discourse*' (Habermas, 1987b, p. 5),

believing this approach enables the identification of possible contradictions that we all live by, but sometimes fail to notice due to their familiarity (Habermas 1987b). In essence, striving for highly respectful and humanising relationships towards women (subjects) within pedagogies where communication is enabled (critical discourse) is the context within which driving progressive change is facilitated by the realisation of a critical consciousness (conscientization), that has the potential to achieve social transformation.

4.4.5 Critical pedagogies in healthcare education

From its development and throughout its expansion, critical pedagogy has been situated as a broad and applied theory of education (Giroux & McLaren, 1986, Shor & Freire, 1987, Solorano, 2000, Giroux, 2001, McLaren, 2015). Ledwith (2015) notes how critical pedagogy is in a continuous process of evolution and development and promotes delivering critical pedagogical teaching with innovation and creativity, keeping conscientization as the end goal. Others agree, stating education that aims to enhance critical consciousness, ultimately fosters compassionate, humanistic, socially conscious health professionals (Racine et al., 2012). To date, despite knowledge of Freire's work, critical approaches to education design have been sparsely applied in the healthcare

sector. Halman et al (2017) suggests that when attempting to apply critical pedagogy within current healthcare structures, researchers are required to pay nuanced and ongoing attention to how a critical pedagogy fits and conflicts with prevailing approaches. This highlights the issues faced when aiming to illuminate power structures and how this may be faced with hierarchical resistance. Jarvis et al (2012) acknowledge this sentiment yet reiterates the importance of delivering humanistic care within healthcare professions as a duty that moves beyond competency-based care and hierarchies of expertise within healthcare institutions. Approaching the design of a critical pedagogy within this study requires an acknowledgment of the on-going resistance to critical approaches and the issues faced when questioning the status quo in the delivery of care. That said, efforts to enact critical pedagogies can be influential, even if radical transformation fails to immediately result, as new democratic imaginaries or small scale situated exemplars are rendered visible, if not necessarily realisable at scale (McKeown et al., 2018).

Critical pedagogies move beyond procedural educational techniques and methods to facilitate reflection and promote equality and social justice through illuminating power structures (Halman et al., 2017). Critical pedagogies also allow participants to recognise connections between individual problems and experiences and the social contexts in which they are embedded (Diemer et al., 2016). Realizing one's consciousness through '*conscientization*', is the first step in achieving '*Praxis*', defined as the ability and knowledge to take action against oppression through liberating education (Taylor et al., 2017). Applying these concepts in midwifery education focused on reducing birth trauma aims to encourage reflective practices, creating discussion of oppressive elements of care and placing those in a wider context, including an examination of potential contributory factors to experiencing birth as traumatic amongst the disadvantaged and vulnerable.

Research suggests that competency-based education falls short of this imperative with a gap identified in how critical approaches can be practically applied within a dominant competency-based curriculum (Ledwith, 2015). Other researchers agree, believing education that focuses on measurable skills and behaviours risks stripping healthcare professionals of personhood and values, as opposed to critical pedagogies that explore assumptions at individual, institutional and cultural levels (Whitehead et al., 2014).

When deciding what framework to use to help inform the programme I recognised that critical pedagogies encourage participants to reflect upon factors associated with psychosocial and cultural influences that cause situations of dehumanisation, opposed to a focus on competence-based assessments. Within an educational programme for maternity professionals, critical pedagogies offer a practical approach that could facilitate reflection upon care delivery to promote emancipation and the humanisation of care during birth, replacing conventional methods of education delivery amongst healthcare staff. Culture circles offer one critical pedagogical approach on how to facilitate reflection and are discussed below.

4.4.6 Culture circles

Freire talks about the creation of *'Culture Circles'* as a way of questioning contradictory, paradoxical or dehumanising viewpoints (Freire, 1972). Culture circles consist of the gathering of people in an educational capacity to facilitate alternative thoughts and critical consciousness when analysing a situation or scenario. This can be facilitated in many ways but begins with presenting information in a different context to encourage and offer a different perspective. By facilitating a dialogue to critique the information presented, it is thought that connections and insights begin to form regarding disempowerment and structural discrimination, providing a new way of seeing the world (Freire, 1972). As discussed in chapter two, midwifery education currently lacks a reflective element focused on aspects of care that create situations of disempowerment, namely factors relating to interpersonal interactions during birth. The notion of culture circles is supported by Ledwith (2015) discussing how reflection upon our own beliefs encourages a deeper level of self-reflection and reflexivity in care, ultimately helping foster greater understandings of health inequalities and issues faced by disadvantaged and vulnerable women. Novel digital technologies as presented and discussed in chapter two arguable also offer means for potentiating the required professional critical reflection necessary for conscientization.

4.4.7 Theoretical synergies: Using critical consciousness to inform midwifery education

Technological advancements in healthcare risk an over reliance on algorithms opposed to physiological processes and human interaction. With technology and science interwoven in the nature of healthcare advances, a turn towards the humanities in technological design provides a key underrepresented aspect of midwifery edcuation,

often missed in the design of educational interventions that aim to teach subjective aspects of care.

Raw content delivered within the learning environment serves as a mean for bringing experience directly up against consciousness (Ledwith, 2015). The use of technology within this study aims to enhance the learning experience within a participatory session, providing participants with evidence-based content for reflection. A critical stance would allow the exploration of the social and political nature of healthcare, the influences power and privilege play in the delivery of care, and how maternity professionals as individuals working within a healthcare culture can deal with unexamined norms that foster oppression. Placed in context of the aims of this study a critical pedagogy could foster autonomous personhood alongside critical consciousness development in conjunction with practical based competencies. This could ensure that midwifery educational systems do not unintentionally create a workforce of midwives skilled in reproducing desired competencies but are unable to advance the increasing social responsibility agenda of healthcare (Halman et al., 2017).

The emergence of technology in education, if utilized within a critical pedagogy, could serve to enhance critical consciousness within a technocratic society. Although the potential dehumanising effect of technology in maternity care is discussed and presented within the research (Tripp et al., 2014, Craswell et al., 2015). Adopting a Freirean approach in the design of educational resources that utilize technology could present a virtue of its use, aimed at the emancipation of women during birth (see chapter nine for a more detailed discussion of the complexities of technological contributions to critical pedagogy).

4.5 Reflections

My critical reading of social constructionist theories chimed with my personal view of the world and how people interact within it, shape it, and imagine future alternatives. A social constructionist perspective allowed me to frame the phenomena of traumatic birth experiences within an understanding that a subjective reality exists, one that is linked to power. I concluded that social relations within maternity care would be viewed through a critical theoretical lens, acknowledging that both maternity professionals and disadvantaged and vulnerable women bring into the birthing room their own interpretations of the world, based on their life experiences. Using a critical pedagogical

approach to the design of an educational programme, underpinned by critical theory, will allow me to keep issues of power and the dehumanization of women at its centre, aiming to facilitate conscientization amongst maternity professionals.

From engaging in the philosophical underpinnings of research and the nature of knowledge I position myself within this research as a social constructionist, believing that knowledge is formed through social processes and shared assumptions about reality. A critical basis for social inquiry ensures that critiques of power and oppression form the normative basis for exploring phenomena, aimed at decreasing domination and increasing freedom in all forms. In line with the aims of this research I have adopted a practical and applied approach to research inquiry and emancipatory action as opposed to a purely theoretical and descriptive account of data. At the crux of exploring disadvantaged and vulnerable women's accounts of birth trauma to inform educational resources during research processes must aim to be explanatory, practical, and normative with emancipation and the critique of power at the centre of analysis within this thesis.

4.6 Conclusion

The critical stance I have chosen to adopt has encouraged me to reflect on my own preconceptions as a maternity professional and academic researcher and my own birth experience as a disadvantaged teenage mother. Critical social theory has furnished a set of perspectives relevant to critique the neoliberal circumstances that frame the work of contemporary midwives and the serial oppressions of vulnerable mothers under their care who face intersectional disadvantages, epistemic injustices, prejudicial social abjection and othering. The writings of Gramsci and Freire have provided me with a framework in which I can engage as a *'change agent'*[19] and a self-identified critically engaged intellectual, with the intention of harnessing technology to enhance midwives' learning to facilitate conscientization. After presenting the argument for my chosen theoretical lens, choosing compatible methods that would enable me to empirically investigate birth experiences amongst disadvantaged and vulnerable women and design a critically informed educational programme will be discussed. The next chapter will

[19] Change agent; essentially, a change agent can be defined as an event, organisation, material thing, or, more usually, a person that acts as a catalyst for change.

present a justification of chosen methods within the study and how these were conducted.

CHAPTER 5: METHODS

5.1 Introduction

The previous chapter provided the description and justification of the epistemological and theoretical approach for this study. This chapter provides the methodological details for my study. As identified in the previous chapters the aim of my study was to develop an education programme to raise maternity professionals' awareness of birth trauma amongst disadvantaged and vulnerable women. In this chapter, I provide methodological details for my empirical phase - interviews with disadvantaged and vulnerable women who have experienced birth trauma. I then describe and discuss the design and development of an immersive education programme that uses virtual reality technology, informed, designed and developed using empirical evidence, underpinned by critical theory. Figure 3 presented in this section gives an overview of the three-step model used to design the programme. Information on the ethical principles that underpin healthcare research and how these were adopted throughout the study are also presented. I conclude this chapter with a summary of my chosen methods.

5.2 Critical methods

As discussed in chapter four, critical social research aims to challenge established social order, disrupt and address cultural conventions and encourage productive opposition rather than accepting surface consensus as a starting point of inquiry when exploring a phenomenon (Giroux, 2001). The aim of adopting critical research methods within this study is to contribute to the emancipation of disadvantaged and vulnerable women and maternity professionals through the delivery of an educational programme. Critical research methods in healthcare create a balance between being informed by theory and adopting an empirically sensitive approach to discovering and uncovering themes of repression for action (Mill et al., 2016). In the case of this study, undertaking a meta-ethnography and empirical interviews with disadvantaged and vulnerable women allowed for an exploration of repressive interactions during birth between women and their health care providers and how these may have contributed to traumatic birth experiences. This data was then used to inform an educational programme using critical approaches to encourage a reflexive praxis on how these situations of oppression could be challenged and addressed.

Research methods are concerned with how a researcher develops their research question, how they attend to the social reality of a given phenomenon, and how a researcher logically and systematically addresses the ambiguity and contradictions within empirical material (Creswell & Creswell, 2017). Classical qualitative methods aim to gain a deeper understanding of the underlying reasons, opinions, and motivations of a given phenomenon (Bryman, 2016). The aim of qualitative research varies dependant on the disciplinary background of the researcher and the topic to be explored, yet the aims remain the same; to research the many *why* and *how* questions of human experience (Crotty, 1998). Critical research places these questions at the forefront of research aims, alongside a critical exploration of power relations and hierarchies that impact upon these experiences. Critical methods are not just a case of managing the data, or how to obtain the data, but a reflexive activity in which theoretical, political and ethical issues are central to chosen methods of research activity (Ledwith, 2015).

5.3 Interviews with disadvantaged and vulnerable women

5.3.1 Engaging participants

The following section outlines all the methods employed to recruit, interview and analyse the interviews with disadvantaged and vulnerable women. The rationale for undertaking empirical interviews was twofold – a) to identify key triggers for birth trauma related to interpersonal interactions between women and maternity care providers and b) to ascertain if women's recent experiences of a distressing/traumatic birth resonated with insights collected via the meta-ethnography ensuring the validity and transferability of findings.

5.3.2 Sampling methods

A purposive sampling method was used to identify participants to be interviewed. This sampling method allowed participants to be identified based on their previous experiences and to ensure they met the criteria of disadvantaged or vulnerable using the characteristics listed in table 1. Sampling methods for qualitative inquiry are generally assumed to enable researchers to purposefully yield cases that are '*information rich'* (Patton, 2002). This approach involves identifying and selecting individuals that are knowledgeable about or have experienced with a phenomenon of interest (Cresswell & Plano Clark, 2011), in the case of this study, disadvantaged and vulnerable women who have experienced a distressing birth. Purposive sampling

would allow for these women to be identified during the recruitment phases as to maximize the efficiency, validity and transferability of findings (Morse & Niehaus 2009).

5.3.3 Sample size / recruitment process

When undertaking qualitative research, good practice includes the conduct of around 20-30 interviews, with the goal of data collection to achieve theoretical saturation (Mason, 2010). The number of participants in qualitative inquiries is usually small due to the large volume of data generated and the amount of time needed to analyse and give justice to participants voices (Sandelowski, 2004). This said, sample size is dependent upon the research question where smaller or larger samples may be required (Vasilios et al., 2018). When considering the sample size for my study I had to address practical considerations, such as the feasibility of data collection (Creswell & Creswell, 2017). Given I aimed to recruit from hard to reach groups, and I was exploring an emotive topic, I expected difficulties in the recruitment phase. My original aim was to conduct 10-20 interviews with disadvantaged and vulnerable women who had experienced a traumatic birth.

5.3.4 Inclusion criteria

The inclusion criteria for women was that they were over the age of 18, they met the definition of disadvantaged and vulnerable and had experienced a difficult or distressing birth within the last three years. Within this study restrictions on time passed since birth were to ensure that findings were reflective of care practices in a locality where the educational programme aimed to be delivered.

5.3.5 Recruitment

Women were recruited from the East Lancashire area in Sure Start / Childrens Centre locations (i.e. attending new mothers/baby groups) and using snowballing methods (Sadler et al., 2010). Prior to recruiting women, I attended the community centres to brief staff about eligibility criteria, study aims and what participation would involve. A supportive relationship with these staff members enabled me to attend groups and meetings that the staff believed would include representative women. Relationships with staff members was built up over time including me meeting with each staff member prior to recruitment phases to discuss my study's aims and objectives and provide them with opportunities to ask questions.

Initially it was decided that staff in the Sure Start centres would play an active part in recruitment as they would have prior relationships with attendant women. However, this raised power related issues and how being asked to take part in the study by those known to them, and potentially perceived to be in a position of authority could be seen as breeching their autonomy and risked being coercive. As a result, it was decided that I would undertake all the recruitment to ensure that the relationships between staff and women were not compromised in any way.

The centres and groups were accessed over a six weeks period (November 1st 2016 – December 20th 2016). Groups attended included playgroups for women and children up to the age of three, mother and baby groups, the sensory room groups and breastfeeding peer support groups. Snowballing techniques were also adopted in which women who agreed to take part were asked if they knew of any other women who may be willing to take part in an interview. When I attended a group session, I provided an overview of my study and explained what participation would include. I informed women that if they wished to discuss the study further, I would be present for the remainder of the session to give them the chance to ask any questions or request a participant information sheet

Women who showed an interest in participating were provided with a participant information sheet. To ensure that women met inclusion criteria, those who expressed an interest were asked to complete a socio-demographic form and prior to collecting contact details (see appendix 5). The form collected information on women's age, marital status, ethnicity, parity, level of education and complex life situations such as difficulties or issues with their mental health, any conflict in their personal relationships or any other difficulties that made them feel vulnerable. While it was intended that women would be advised at this stage if they were not eligible to take part, all the women who were approached and expressed an interest were eligible. Following the completion of a sociodemographic form contact details of women were taken and recorded (see appendix 6).

A 24-hour cooling off period was then given. Women were called at least 24 hours later to establish whether they still wished to take part. If women expressed an interest in taking part in an interview, a date, time and location was agreed. While I had originally intended to recruit between 10-20 women, overall ten representative women

participated. A decision to stop after 10 interviews was also made after reaching data saturation[20] (Saunders et al., 2018).

5.3.6 Data collection

At the start of the interview I provided women with verbal information about the study and offered them a chance to ask any questions. All ethical considerations were discussed to ensure women were fully informed as to the aims and purpose of the study, voluntary nature of participation, right to withdraw and potential confidentiality issues. These considerations were clearly addressed in the participant information sheet. Once all queries had been addressed the woman was then asked to sign the consent form (appendix 7). Women were reminded at this point that they did not have to answer all the questions, and could stop and end the interview at any point. Women were also advised that the interview data could be withdrawn up until one-month post interview.

Semi structured interviews were chosen to collect data and an interview guide was used to ensure these was focused on exploring issues relating to interpersonal interactions, as seen in appendix 8. This semi structured interview method of data collection enables participant's experiences of a phenomena to be explored by providing rich detailed accounts of their experience (Sandelowski, 2004). It is important to note that interviews are not usually recommended when undertaking critical qualitative inquiry due to the inherent nature of power imbedded in the relation between interviewer and interviewed (Green & Thorogood, 2018). Interviews may cause barriers due to an imbalance of power, for example between two people in dialogue or the relationship between interviewer and interviewee (DiCicco-Bloom & Crabtree, 2006). Cotterill (1992) advises researchers to steer away from using interviews as a method for gaining insight to power, criticising the lack of active participation as passive recipients of questions, yet Morse (1994) argues that if researchers remain aware of their interview purpose, the theoretical model that underpins the research and the relevant methodological issues that may occur then the lived experiences of power can be explored ethically.

[20] Data saturation – Data saturation has attained widespread acceptance as a methodological principle in qualitative research. It is commonly taken to indicate that the data have been collected or analysed hitherto, further data collection and/or analysis are unnecessary

When undertaking interviews, I used several strategies to address the potential imbalance of power, these strategies are discussed further in section (link to ethics section below). While other data collection methods were considered, i.e. focus groups, alternative methods were deemed inappropriate due to the personal and emotive nature of a distressing, traumatic birth, and the fact that women may be less likely to share rich insights in a group setting. Once women had voiced their birth story and paused for prompting, a more conversational/dialogical style of engagement was adopted. This involved me as a midwife reflecting upon the issues voiced by the women, using prompts to explore issues of power during their interactions with health care professionals. In summary, interviews allowed for in-depth emic accounts to be captured therefore deemed a suitable approach to explore factors associated with interpersonal interactions with health care professionals during the birth experience.

5.3.7 Style of engagement

During the interviews it was important to ensure I nurtured a supportive and relaxed environment while being aware of my position as a midwife when exploring negative and distressing interactions with women. The researcher disclosing personal information during these interactions is valued as an alternative approach to traditional hierarchical interview encounters, and strongly recommended within feminist literature (Oakley, 1981). As interviews often provoke strong emotions, and particularly in regard to my subject area, it felt important, as advocated by Corbin et al (2003) that I established a rapport prior to commencing the interview. Luckily my strong working-class Lancashire accent and modest dress sense provided me with a less intimidating presence when speaking to women, although I sensed unease from some of the women in relation to my job. Some women asked direct questions seeking reassurance regarding confidentiality. For example, one woman asked a question concerned information sharing:

> *'So even though you work at (name of hospital) you're not going to mention my name at work, are you?* (Participant 4).

Women were reassured that no identifiable information would be used in the presentation of the findings. I explained to women that I was bound by the Nursing & Midwifery Council NMC Professional standards of practice and behaviour for nurses, midwives and nursing associate to uphold confidentiality (NMC, 2018), alongside the

ethical principles that underpin research activities, discussed in more depth below. Two of the women also made statements about their poor interactions with midwives and then apologised to me about their feelings towards my profession and the hospital Trust where I worked:

> *'In all honesty it's made me feel like I'd never go back there to have my baby, no offense'* (Participant 2).

This statement highlights the importance of building trust when interviewing women, acknowledging possible pre-existing thoughts and feelings towards the midwifery profession when exploring birth experiences. To build up a trusting relationship in a short space of time, I first began by instigating unrelated conversations. I asked women about their children, family, home and hobbies until I sensed that the woman felt relaxed and comfortable with me. Alongside these conversations I entwined my own story and spoke about my own life including children, work and upbringing. This was an important part of the interview process as although I saw myself as equal and aligned with the women I was speaking to, I often forgot that by being a midwife and a researcher those who do not know me may make presumptions about my personal circumstances and social status.

Once a rapport had been built up with women I asked if they were happy to continue with the interview. I reminded women that I would be recording the interviews, placed the audio-recorder on the table and reassured women to not feel nervous and encouraged them to speak freely. I started the interview with an open-ended question: *'Can you tell me about your birth experience'.* This question allowed women to begin their story where they felt most comfortable and provided me with insights as to what information was important to them. Once women had finished narrating their story, prompt questions were used dependant on the level of information disclosed. These questions included *'How did that make you feel', 'How do you feel you were treated during the birth?' 'How would you describe your relationship with the healthcare staff'?* These questions allowed for a deeper exploration of given descriptions or clarification of the issues raised during the interview. At the end of the interview, women were asked if they knew of anyone else in their personal networks who may be willing to take part. On these occasions, a further information sheet was given (for subsequent transfer to

the potential participant), and women asked to contact me direct. As discussed above in section 5.3.3 data collection continued until saturation had occurred.

5.3.8 Transcribing and storing the data

All interviews were audio-recorded following consent and transcribed in full to ensure accuracy and quality of data collection. Non-verbal information was included in the transcripts including emotions, pauses and disruptions. All interviews were transcribed by me. Transcription allowed me to revisit each interview on a deeper level during the process, including annotating the transcriptions, noting pauses and emotions. All data was stored in line with the Data Protection Act (1998). Data was stored securely on University premises in locked facilities (i.e. hard copy data – consent forms) or via password protected/encrypted University IT systems and files (e.g. interview recordings and transcripts). All information was linked by a code (i.e. consent form, interview transcript and socio-demographic information) and any personal/sensitive data (consent/socio-demographics) stored separately.

5.4 Data analysis

Data analysis was conducted using MAXqDA software for data analysis. This form of software was chosen for its ease of use and its ability to store and manage high volume of text data. Debates exist on the use of computer software to manage qualitative data arguing that this form is inflexible and mechanical (Holloway & Wheeler 1996). That said, it is important to note that the software does not analyse the data, rather it is a useful and practical tool to help organise and manage large data sets (Carter, 2004). Methods of data analysis need to be based on how best to answer the research question. For this research, specific questions needed to be answered in terms of what interpersonal factors influenced disadvantaged and vulnerable women having a traumatic birth.

5.5 The framework method

When considering which analytical framework to analyse the transcripts, it was important to consider that: a) I wanted to focus on the triggers for trauma within the interpersonal interactions between women and their maternity care providers during childbirth; and b) in order to create a more robust, evidence-based content for the video scenario, I wanted to map these triggers against the accounts of women in the included studies in the meta-ethnography; thereby validating these findings within the wider

literature. I therefore needed an approach that allowed for inductive and deductive methods of analysis.

The framework approach was developed in the 1980's by qualitative researchers working in an independent school for social research and social community planning (Ritchie & Lewis, 2003). Several authors have since provided guidance on how to conduct the framework approach within the context of healthcare research (Gale et al., 2013, Ward et al., 2013). A framework analysis was considered an appropriate method to analyse the data within this study as it involves a twostep process which includes both an inductive approach to data analysis (i.e. to identify the key triggers for birth trauma within the empirical accounts) and a deductive approach (i.e. to map findings across existing data, which could identify new/unreported areas as appropriate) (Spencer et al., 2003).

5.6 The benefits of using a framework approach

A framework approach to analysing data makes certain a priori assumptions about the kinds of themes that might be in the data it is used for (Spencer et al., 2003). It therefore differs from a purely data driven thematic analysis, in which themes emerge iteratively as the analytic process progresses (Gale et al., 2013). It is described as a straightforward approach to analyse qualitative data that provides transparent results that are relatable to the original data (Ward et al., 2013) and can be performed both during and after data collection (Ritchie et al., 2013). A framework analysis also provides researchers with the option to examine studies separately and then combined in the final analysis (Gale et al., 2013). This approach is used to recognise and report cross-cutting themes, enabling others to identify how decisions were made when deriving themes from the data (Ritchie et al., 2013).

A framework analysis was utilized to address specific questions related to the study's aims and objectives. The framework approach allowed me to examine traumatic birth experiences within a context (the social space of birth), by focusing on the interpersonal interactions that caused situations of trauma from women. The purpose was to identify key mechanisms and social processes that contribute to a traumatic/distressing birth, to provide the educational programme with evidence-based content.

A modified version of the framework method developed by Gale et al (2013) was used within this study. Gale's worked example was chosen as it offered a structured and comprehensive application of the framework method for health-related research. In the following sections I describe each of the steps within the Gale et al (2013) approach, outlining how it was applied and/or modified as appropriate when analysing the interview data.

5.7 Stage 1 Transcription

This phase of Gale et al's (2013) approach relates to providing verbatim transcripts. I transcribed all the interviews myself to help familiarisation with the data, with each interview given a number to aid in coding in the subsequent stages. Although within a framework method it is not necessary to include the dialogue conventions (Gale et al., 2013) (i.e. emotions displayed) I felt this additional contextual related information was important as I was exploring a highly emotive topic with the women. Before beginning the process of sifting and analysing the data, it was important I became familiar with the range and diversity of insights captured (Easton et al., 2000).

5.8 Stage 2 Familiarization with the interviews

In line with Gale et al (2013) approach, this stage involved gaining a sense of the data set as a whole. During this stage I re-listened to the audio recordings and immersed myself in each interview without interruptions. Listening to the interviews allowed me to gain a sense of what was important to the women and to map key emergent issues:

'*Making analytical notes, thoughts or impressions*' (Gale et al, 2013, p. 4).

5.9 Stage 3 Coding

This stage relates to coding of the data set (Gale et al., 2013). This phase involved inductive coding with a specific focus on interpersonal interactions due to their association with birth trauma. I underlined key parts of the text and used the left-hand margin to describe the content of each passage with a label or code. This could range from only a few words, to parts of sentences or whole paragraphs. I then used the right-hand margin to record more detailed notes and ideas, for example questions to bear in mind as the analysis proceeded and ideas for explanations or patterns in the data. As women interviewed were discussing their traumatic experiences of birth, narratives were predominately of a negative nature when discussing interpersonal interactions with their health care providers. That said, during coding all aspects of interpersonal

care were captured and coded, this included positive aspects of care. In Table 7 below, an excerpt of open coding is presented.

Table 7 Example of Interview transcription coding undertaken on interview transcripts.

Coding labels	**Participant 9 Interview**	Notes & Ideas
Self blame	123 Yeah maybe that's what is was, I don't know, but<br>124 anyway apparently, I wasn't pushing enough, he was<br>125 tired and I was tired, so they had to give me an<br>126 episiotomy. Erm, the midwife was saying before that,<br>127 push if you have a contraction, don't push if you don't<br>128 have one yet the other woman was like push push,	Self-blame, felt she wasn't pushing hard enough therefore pain her fault
Poor information<br><br>Confusion about the process	129 Samantha push push, I was thinking oh my god what<br>130 am I doing am I pushing when I have one or when im<br>131 not having I was really confused to what I was<br>132 supposed to be doing. it was my first baby so, I didn't<br>133 know what to do, both like saying different things so I	Feeling unsure about the process causing her to feel scared and fearful
Loss of control	134 was like what the hell am I doing? In the end I thought<br>135 I'm just going to push when I'm not having one because<br>136 I can't cope with this anymore I just want him out, I<br>137 kept looking at the clock and thinking what time is it<br>138 going to be when he comes out? Erm, so they give me	Emotive language used to convey the message of feeling out of control of the situation
Abusive care practices	139 the episiotomy that consultant woman who ever she<br>140 was literally arms in trying to stretch me to get him out,<br>141 I remember her saying to me 'Samantha, your too tight'<br>142 I was like what do you want me to do, she said it about	Describing procedures as in inhumane to emphasise the distress it caused
Painful procedures<br><br>Submissive interactions<br><br>Unconsented interactions<br><br>Fear	143 three times she had her leg up, doing this, that hurt<br>144 more than anything, and my bum kept shooting off the<br>145 bed and she kept shouting at me 'Samantha, keep your<br>146 bottom down' she was horrible, honestly she was<br>147 horrible. I was like I can't your hurting me, and that's<br>148 the only time I made a noise like a man because she<br>149 was killing me, anyway, I just kept pushing and pushing	Consultant shouting at her illuminating power issues and submissive interactions during birth

5.10 Stage 4 Developing a working analytical framework

This stage involves developing a framework to organise the data in a manageable and meaningful way (Gale et al., 2013). Identifying a framework involves creating a new structure for the data (rather than the full original accounts given by participants) which is helpful when summarizing and reducing the data to answer the research questions (Gale et al., 2013). This stage requires the researcher to develop a framework that is characterised by both a priori assumptions, alongside emergent aspects of the data identified from the initial familiarization stage. Bryman and Burgess (2002) state that at this stage the researcher is not only gaining an overview of the richness, depth, and diversity of the data, but also beginning the process of abstraction and conceptualization.

Devising and refining a framework is not an automatic or mechanical process but involves both logical and intuitive thinking (Bryman & Burgess, 2002). Within this stage it involved me making judgements about meaning, about the relevance and importance of issues, and about implicit connections between the issues discussed. This process allowed me and my supervision team to agree on a set of themes to form the initial analytical framework. These were identified by discussing codes assigned from each transcript as noted in table 8 and coming to an agreed consensus on what the data was highlighting.

The process of applying and refining the framework was repeated multiple times until no new codes emerged from the data. The final framework consisted of 32 codes.

5.11 Stage 5 Applying the analytical framework

I applied the final analytical framework to each transcript using MAXqDA software. Each transcript was revisited whereby each relevant passage of text was assigned one of the 32 codes from the final analytical framework. Below in table 8 an excerpt from the transcript for 'Participant 7' provides an example.

Table 8 The application of codes to interview transcripts.

Participant 7	Codes
She has a horrible Scottish accent and it sounded like she was always shouting at me. She kept saying you can't get up, stay on the bed when I needed a wee and just kept telling me baby wasn't happy and the heartbeat keeps dropping. I kept saying is baby going to be ok and she kept saying she cant guarantee anything, but I was in the right place, I kept thinking well that doesn't really help me does it. I was led there thinking my baby is going to die any minute and it was really upsetting me. I was hooked up to a hundred different things honestly, my blood pressure was up too so I was on this drip to help me, something about protecting baby's brain she (midwife) said, so then I'm thinking she's got brain damage already. Maybe that's why she is behind with stuff now because her heartbeat kept dropping then, can that happen?	Negative interactions<br><br>Feeling restricted<br><br>Not sure what was happening<br><br>Women didn't understand what was being said

5.12 Stage 6 Charting data into the framework matrix

Qualitative data can be quite voluminous generating pages of text. Each interview lasted an average of one hour generating over 50 pages of text to be charted into the framework matrix. Being able to manage and reduce data into a manageable format is vital in the analysis stage. Once all the data had been mapped using codes identified in step 4, all data was summarised in a matrix format using Microsoft Excel.

This stage involved a more in-depth analysis in considering how the codes were conceptually related. For example, as can be seen in table 9 four codes related to the same underlying principal and were grouped together to contextualise the theme *'poor information giving',* relating to the many ways in which women felt the information they received was inadequate.

Table 9 Refining and reconceptualising the codes.

Code	Description
**POOR INFORMATION GIVING**	
**Knowledge of midwife**	*Midwife unable to provide adequate information when asked (women aware the midwife lacked confidence – possible impacting upon the woman's confidence?)*
**Rushed information**	*Rushed procedures, not enough time given to ask questions (Lack of informed consent)*
**Not sure what was happening**	*Women feeling frightened when being taken to theatre unsure of what was going to happen (fear of the unknown)*
**Women didn't understand what was being said**	Inappropriate use of medical terminology *(women felt demoralised / exempt from conversation)*

Six key codes (triggers for birth trauma) emerged from this stage and are detailed in chapter 6. In line with Gale et al's (2013) approach these codes were used on a deductive basis to revisit the transcripts, while still allowing for an inductive process whereby data that did not 'fit' could be charted.

5.13 Step 6.1 Additional step to cross reference triggers for birth trauma across studies within the meta-ethnography

This step was undertaken as an additional aspect of the data analysis. At this stage the key codes/triggers identified through stages 1-6 above were deductively applied to the studies included in the meta-ethnography to validate the findings within the wider literature. This step included re-reading each study and using the six key codes as a lens to assess whether the same (or different) issues were evident. To undertake this work, I created a matrix into which I could input findings, specifically allowing me to map the codes within the individual studies to ascertain which were present and which were most prominent. Findings from this step are detailed in chapter 6.

5.14 Step 7 Interpreting the data

Within the Gale et al (2013) framework they describe step 7 as a process of creating a typology and/or theoretical concepts. As the purpose of undertaking the analysis was to identify, chart and validate the key interpersonal triggers for birth trauma, this phase was not explicitly undertaken. Although a critical theoretical lens was used to design the educational programme (see below). The aim here was not to theorise how women experience birth trauma, rather to elicit descriptive factors to be utilised within a critical pedagogy.

5.15 Limitations of framework approach

As with all research methodologies used in qualitative analysis, the risk of adopting the framework approach is that others may argue it leads researchers to engage with data in a linear mechanical fashion. The risk relates to researchers attempting to quantify qualitative data i.e.; 'X number of participants believed Y' due to the matrix format adopted and the use of spreadsheets to organise the data. While this critique is valid as the steps do provide a systematic way of managing, indexing and charting data, within this study these steps served as a practical way of making the data more visual and enhanced my ability to map and interpret the findings appropriately. Polit & Beck (2010) point out that qualitative analysis is not designed to be representative of a population but to purposively capture the diversity surrounding a particular phenomenon. Gale et al (2013) emphasises the involvement of multiple stakeholders and disciplines in the analysis and interpretation of data within a framework method - highlighting the labour intensity of including many different perspectives resulting in decision-making being very time consuming and resource-intensive. In my study this was managed well within my supervisory team by sharing accounts and holding rich discussions.

The inclusion of an additional layer enabled me to assess the validity of the findings from my empirical work to the findings from a wide body of literature. This stage enabled me to generate a more robust and accurate representation of the key triggers for trauma within the interpersonal interactions between women and their health care providers during childbirth. The themes emerging from the application of the framework method, were subsequently used and mapped within a real-life filmed scenario that formed part of the educational programme.

5.16 The design and delivery of an immersive education programme for midwives using VR technology

This section discusses the methods chosen and steps taken during the design and delivery of the immersive educational programme.

5.16.1 Choosing an approach to design a critical pedagogy

At this stage, I began engaging in the literature concerned with delivering critical pedagogies, searching for use cases, examples and potential design methodologies to guide this phase. From engaging in the literature, it could be argued that the core understandings of critical theory risks being misunderstood as radical social protest by educational institutions, thereby hindering its successful application in practice (How, 2017). Carr & Kemmis (2003) believe that because critical pedagogies question systems of power and oppression they can often be mistaken as anarchistic and rebellious by those in positions of authority. Giroux agrees, yet acknowledges that a level of resistance is a fundamental step in changing practices and cultures and is a necessary step in the application of emancipatory interventions (Giroux, 1979).

Mill et al (2016) suggests that to enhance chances of successful application, researchers who use critical theory should aim to follow a mainly conventional approach to qualitative methods and then add specific elements from a critical approach to apply the findings into practice. Following this advice, rather than adopting a purist and distinct critical methodology for my research, I felt it important to adopt a framework where I was guided by the principles, guidelines and reflections for qualitative research underpinned by critical theories. I therefore aimed to address issues of power as a reflective tool for emancipatory action.

It became apparent when engaging in the literature, that although critical pedagogical approaches have been utilized in many areas, there was a lack of specific use cases in the design of educational resources for healthcare professionals. A three-phase model based on the writings of Freire was identified within a paper by Matthews (2014). Within this paper, Matthews uses specific cases that have utilised a critical pedagogical approach, such as the study by Sharma & Romas (2012) who use the SHOWED approach to facilitate critical thinking (discussed in more depth below). Matthews model provided me with a framework of how to design the educational programme and cases and ideas on how a critical pedagogical approach could be facilitated. In figure 3, I provide a visual

representation followed by a brief description of the three stages, followed by a detailed description of how the immersive educational programme was developed, drawing on the specific cases and steps detailed by Matthews (2014).

Figure 3 Overview of the immersive educational programme.

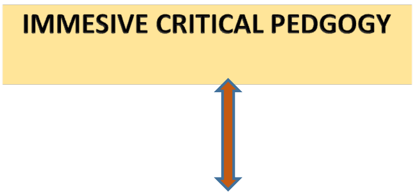

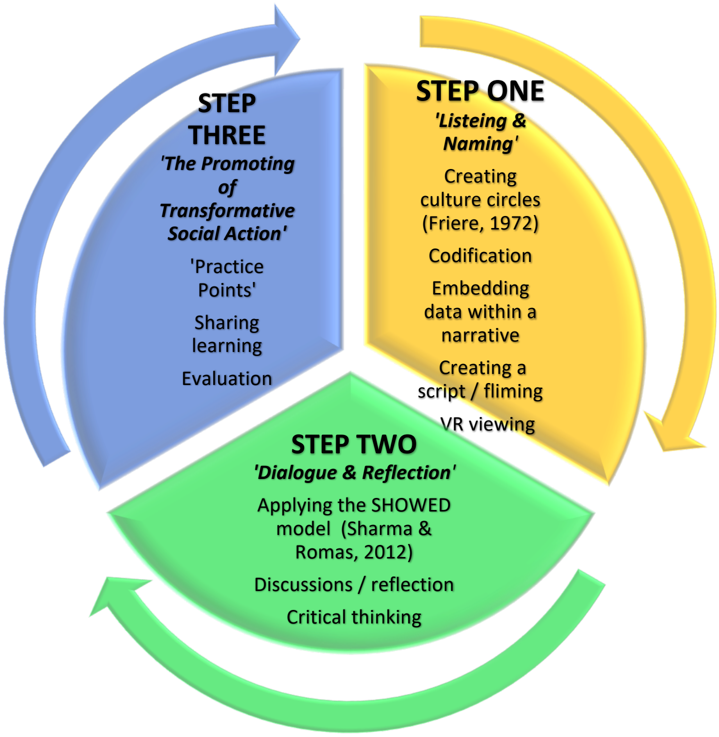

5.11 Overview of the three-phase critical pedagogy model

Identified above, the three-phase model designed by Matthews has three main stages: Listening and Naming; Dialogue and Reflection; The Promoting of Transformative Social Action (Matthews, 2014). The first step *'Listening and Naming'* relates to learning the problems, issues and real-world experience of the learners. In the case of this study - the contributory factors to birth trauma within the social space of birth. Second – *'Dialogue and Reflection'* - allows for a problem-posing approach, encouraging critical thinking amongst participants (Matthews, 2014). When adopting problem-posing approaches, learners are encouraged to think critically, enabling them to focus on learning as opposed to outcomes. An example of this approach is utilising the SHOWED model as seen in figure 5 and discussed in more detail in section 5.13. Finally – *'The Promoting of Transformative Social Action'* - participants are facilitated in challenging

the ideas and practices that support inequality and come up with their own ideas about what action to take rather than having other people's ideas imposed on them (Matthews, 2014).

In the following sections each stage of the model is discussed in more depth, together with details as to how it has been applied in my study.

5.12 Step 1 listening and naming

The listening and naming step within the three-step model provides the opportunity for participants to draw upon knowledge of their own environment and culture (Sharma & Romas 2012, Halman et al., 2017). This approach presents issues from participants' daily lives, involving a process of listening and discussing to uncover themes and issues which are of interest and concern (Wallerstein & Bernstien, 1988, Halman et al., 2017). This approach is popular with educators working with marginalised groups who believe they are powerless to change their environment and lives (Aliakbari & Faraji, 2011). Drawing on the findings from the recent large international programme - Work, Health and Emotional Lives of Midwives in the United Kingdom study (Hunter et al., 2018) – this study identified that 83% of midwives reported burnout in their job, 66% stated they had thought of leaving the profession, 52% dissatisfied with the quality of care they were able to provide and over one third were experiencing moderate/severe/extreme range for stress (36.7%) anxiety (38%) and depression (33%). These insights thereby highlighted how midwives themselves are also marginalised in an oppressive system, in which they feel powerless to change their environment and working lives.

When drawing on the works of Freire, examples of listening and naming are present in his literacy programmes. He spent time within the environment of his learners to develop a vocabulary so he could adapt his pedagogy to the cultural needs of those in attendance. This enabled him to adapt his teaching to create culturally contextual and appropriate content to aid reflection, learning and dialogue (Ledwith, 2015). As a practising midwife I felt I held a depth of understanding of the cultures, environment and language used in practice. As I had also spent time researching and interviewing disadvantaged and vulnerable women, I also had an understanding of their experiences and lived reality, providing me with a holistic and informed approach to the listening and naming stage. This helped me in creating content that was context specific and authentic.

5.12.1 Codification

The listening and naming step provides an opportunity for learners to use knowledge from their own environment and culture to create points for critical reflection (Wallerstien, 1992, Sharma & Romas, 2012). This step included using women's experiences of care identified during the meta-ethnography and through empirical interviews to create a 'codification'. Codification is a pedagogical technique to draw issues from learners' daily lives (Matthews, 2014). A code is a physical representation of an identified community issue (Wallerstien & Bernstien, 1988) or in the case of this study a practice issue. According to Matthews (2014) a code can be a case study, role play, story, slide show, photograph, song, picture, video, YouTube clip or poetry. An effective code portrays a problem or social issue that has many sides (Fernandez-Balboa & Marshall, 1994). For example, in a study by Wallerstein & Bernstein (1988), patients' life stories were used as a dynamic code in which learners were shown videos of real patients' life stories to enhance dialogue and reflection. They explore codification further, stating:

> '*No matter what the form, code is a projective device that is emotionally laden and identifiable to student.'* (Wallerstein & Bernstien, 1988, p. 19).

Matthews (2014) believes that within a critical pedagogy film and photographs serve as a code that:

> '*Reflects the community back upon itself, mirroring the everyday social and political realities that influence people's lives'* (Matthews, 2014, p. 5).

By listening (*meta-ethnography & Interviews*) and naming (*Identification of triggers for birth trauma*), it was important to decide how this information would be presented to participants to aid the next stage within the three-phase model, dialogue and reflection. The use of codification in this study was done via film, creating a script that used identified triggers as codes, creating an emotionally powerful real-life scenario for reflection; a woman experiencing a traumatic birth. Steps included in the design of the filmed scenario are discussed below.

5.12.2 Using narratives with a critical pedagogy

Methods of delivering education using narrative style approaches have been supported in many texts as a means to deliver innovative training to health care professionals

(Greenhalgh, 1999, Carson, 2001, Laukner & Doucet, 2012, Jurecic, 2013, Batt-Rawden et al., 2013). Narrative pedagogies have been identified as an effective tool to restructure education in health care to reconnect staff with why they chose to work in a caring profession (Ironside, 2006). Research has been undertaken with promising results using narrative action learning sets to educate staff (Gerdtz et al., 2013). Within a critical pedagogy, presenting real world situations forms the basis of raising critical consciousness amongst participants in which they can illuminate situations of oppression and dehumanisation, although there remains little guidance of how to structure and embed narratives within a critical pedagogy. Within this study, I felt that narrative pedagogies could allow for an engaged understanding and discussion about both the context of care delivery and women's experiences, aiming to facilitate critical consciousness amongst participants.

5.12.3 Writing a script

In order to deliver a narrative as part of a critical pedagogy, a script needed to be written. This process was a new endeavour for me and required an engagement with film and scriptwriting literature concerned with collating and developing reality-based films. This was a difficult task as it required me to read research in a different field. I accessed the University's library to identify any key books related to screenplay and script writing. By doing this I was able to identify key authors in the field. I used these authors names to run an author search in the library's database to bring up relevant literature and references. Through this work I discovered a paper titled *'Research based script development'* (Batty et al., 2016). The paper drew on creative methodologies to find ways of conceiving and executing screenwriting differently within academia, using research to underpin creative practice and creating an '*Academic Screenplay'* (Batty et al., 2016). This paper identified the benefits of using empirical research to create screenplays, encouraging academics to engage in the creative arts to develop and design educational material. Although the paper offered little practical guidance on the development of such a screenplay, the premise resonated with my own thoughts and feelings on how empirical accounts gathered from research could be used to create meaningful content for the film.

The final script used in the final film was co-written by myself and my supervisory team and shared with maternity clinicians to ensure the script was representative of a birthing

scenario and that it was representative of the empirical data (i.e. the interviews and meta-ethnography). Figure 4 shows how each stage in the process informed the development of the script.

Figure 4 Stages in the development of the script.

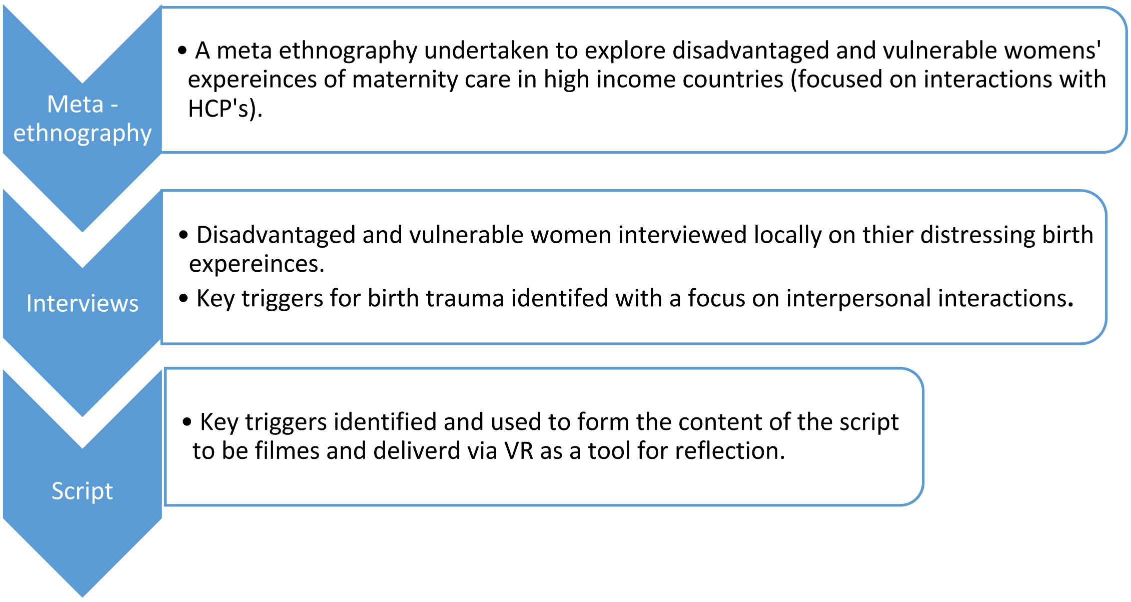

5.12.4 Considering perspectives

I had known when I embarked upon the study that I wanted to utilise interactive methods of delivering education. The idea to use VR came about during attendance at a conference where I used VR for the very first time[21]. This made me question the possibility of using VR in the planned midwifery education programme and the impact this could have on participant's perceptions of care when experiencing it from a first-party woman perspective. As discussed in chapter two, the emergence of VR technology in healthcare is unexplored in terms of its effectiveness and acceptability. Delivering reality-based stories via a first-person perspective in midwifery education has not been used previously, therefore there was no previous literature to draw upon regarding the design. A recent meta-analysis, amongst other literature, identify the positive benefits of utilizing VR in medical education (Nicholson et al., 2006, De Faria et al., 2015, Cochrane 2016, Gunn et al., 2018, Kyaw et al., 2019). Benefits include enhanced learning

[21] During attendance at the ICM Toronto the company Johnson & Johnson invited delegated to experience VR to immerse them into the body of a baby at bath time. For more info please visit a blog written about how I came to explore the idea of VR in midwifery education please read the blog written by myself and published in March 2019. (https://www.all4maternity.com/reducing-traumatic-birth-experiences-immersive-education-for-midwives-bringing-research-to-life/)

capacity, noting how its use immerses the viewer into a space that enhances the senses, thereby providing an innovative medium compared to classic and current educational methods (Daniela & Lytras, 2019).

VR has also been reported to enhance emotional responses (Schoeller et al., 2018, Marín-Morales et al., 2018) and an increased retention of knowledge compared to conventional methods (Huang et al., 2019). A recent RCT evaluating the effect of virtual simulated learning in nursing education also reported significant improvements in knowledge (P=.001; d=1.13), and learning satisfaction (P<.001; d=1.33) after using VR (Padilha et al., 2019). Given the promising results from existing studies and a lack of use cases within midwifery that I could draw upon, I decided that an experiential approach would be adopted. I concluded that if the scenario could not be facilitated using VR to enhance the learning experience, the final film could still be shown via conventional methods, such as on screen without compromising the original aim; reflection on care from a first-person perspective to facilitate reflective discussions and conscientization.

5.12.5 Filming - Technical aspects

The intention to use VR was fully supported by my supervision team. I was encouraged to undertake some exploratory work to assess the feasibility of this approach. During the design phase I collaborated with the university's media and innovation team to discuss my idea and explore the practicalities of filming from a first-person perspective. As a starting point I bought a Kodak 360 camera to test the practicality of the idea and to ensure it was possible to design and film the scenario within the given time frame using the finances and time I had available for my study. During this stage I attended the supporting hospital Trust to test the camera and filming angle and to produce some prototype material to edit and produce with the innovation team. Image 1 is myself in a hospital bed with the camera strapped to my head, testing out different angles for filming and gathering some content to edit.

Image 1 Prototype testing

Appendix 11 provides an in-depth explanation to the technical aspects involved with the filming and editing. This information was created during the test runs stages to guide us in the final shooting of the scenario. It was also important at this stage to consider the room in which we would film as all rooms in the hospital ward were slightly different.

The location, the equipment in the room and the lighting were all considered prior to filming, settling on one of the bigger rooms in the unit for practicality reasons. Elsasser & Hagener (2015) highlight the importance of creating a narrative field that tells a story with the props, content and set alongside the narrative and actors. In this situation that was not hard to achieve as the set was a birthing room on site in an NHS maternity hospital. As identified in section 5.12.1 using knowledge from familiar environments and culture helps to facilitate critical reflection (Wallerstien, 1992, Sharma & Romas 2012), therefore it was important that the scenario that was to be shown was real not a simulated birthing room, which could have been replicated within a filming set.

5.12.6 Choosing actors

Once all technical aspects were in place the next step was to choose the actors. Initially the use of midwifery students was discussed, however, it felt important that the final product was professional and realistic to enhance its acceptability when being delivered in practice. A film producer (who was known to my Director of Studies, has been involved in other education-based activities) was contacted and actors were recruited following a brief on characteristics required (i.e. a doctor, woman and midwife). It was decided that the doctor providing the care would be male. This decision was made as most women during the interviews had received care from male doctors, rather than female doctors. The midwife was to be a woman, as again it was felt this would be more representative of the reality of women receiving midwifery care in the UK. The ethnic background of the woman was deliberated as there was a conflict of agreement between myself and my lead supervisor. I had decided that casting a woman from an ethnic minority background would allow the scenario to be representative of diversity and to present issues related to race and culture when discussing the scenario with midwives. My supervisor did not feel this was necessary due to the risk that the discussion would focus on ethnicity-related issues, rather than interpersonal interactions. In contrast I felt that a woman's ethnic background could highlight unconscious biases, illuminating important and pressing issues regarding inequalities and potential institutional biases, thereby facilitating critical consciousness amongst maternity professionals.

These steps conclude step one of the three step model of designing a critical pedagogy, leading onto step 2.

5.13 Step 2 Dialogue and reflection

Dialogue and reflection are key components required during step 2 of the critical pedagogy design method. This step gives learners the chance to contest assumptions, uncover inequalities and discover and establish practices that are equitable and empowering (Culpan & Bruce, 2007). In the study by Sharma & Romas (2012), a model named 'SHOWED' was used to structure delivery and engagement during critical discussions, drawing upon critical inquiry to address situations of oppression. Participants within the study were asked five key questions following participating in the Alcohol and Substance Abuse Prevention programme. SHOWED emphasised the importance of addressing the reflection of what participants '**S**ee', the reality of what is '**H**appening', encouraging participants to ask 'how does this relate to **O**ur lives', '**W**hy' did what is to be discussed occur, how can they encourage '**E**mpowerment' and what can they '**D**o' about it (Sharma & Romas, 2012). The SHOWED model was adapted from Freirean techniques and is presented in figure 5.

Figure 5 The Showed Model as seen in Sharma & Romas (2012)

SHOWED model (adapted from Sharma and Romas 2012)
• What do we **S**ee here?<br>• What is really **H**appening?<br>• How does the story relate to **O**ur lives?<br>• **W**hy did the person acquire the problem?<br>• How is it possible for this person to become **E**mpowered?<br>• What can we **D**o about it?

The SHOWED model of questioning provided me with a practical guide for the inclusion of critical questioning techniques when designing the structure of the educational programme. Through this process of reflection, learners are challenged to define what they see and feel; to critically present the many levels of the problem; encouraged to share comparable experiences; raise questions on why the issue exists and to collectively develop action plans to address the problem (Sharma & Romas, 2012). Discussion based sessions as part of the educational programme were therefore felt to be crucial to facilitate participant's thoughts and experiences of traumatic birth experiences for vulnerable and disadvantaged women and how interpersonal interaction may impact upon experiences. This SHOWED model has been found to encourage participants to challenge assumptions, expose possible inequalities and seek

out and establish working practices that were more socially equitable (Sparkes, 1992, Culpan & Bruce, 2007).

5.13.1 Theory based session. Adding context

A further element within the dialogue and reflection stage was to present empirical insights into birth trauma and PTSD. This information included findings from the meta-ethnography and interviews, findings on prevalence and incidence of birth trauma/PTSD and risk factors associated with PTSD onset. It was felt that this information was required to provide evidence-based insights to raise knowledge and awareness, alongside the discursive and reflection-based activities.

An additional part of the education programme involved participants being given additional context to the scenario. The information provided was as follows:

> *'Emma [fictional name given to the woman who is labouring within the scenario] is new to the area, she is originally from London fleeing from an abusive relationship. She is 17 years old and had a history of sexual abuse and drug use. She spent some time prior to arriving in the area in a women's refuge, this is her first baby'*

Freire states that to achieve praxis, education must include a cycle of theory, application, evaluation, reflection, and then back to theory (Freire, 2018). Adding context during this step facilitated participants engaging in critical thinking, encouraging them to revisit the scenario and to specifically consider how care practices (and how it is experienced) may differ for women who are vulnerable and marginalised. The SHOWED questions seen in figure 5 were then presented to the group again to explore how these complex factors may impact upon their practices and the experience of women during birth.

5.14 Step 3 The promotion of transformative social action

The final phase in the Freirean model is the promotion of transformative social action, or the critical action phase (Matthews, 2014). This process is where those taking part in the programme take part in the continuous process of action and reflection to facilitate praxis (Freire, 1972). This step is often identified as difficult to implement (Matthews, 2014) and is discussed further in relation to this study in chapter 8 where I discuss some of the difficulties participants faced. Critical action has been identified as having three different forms; campaigning, awareness raising and education (Andreotti & Souza,

2008). Campaigning or convincing an organisation or government to change policy could be at one end of the spectrum of possible change (Matthews, 2014). On the other end, this can relate to equipping learners with the tools they need to identify inequality and injustice so that they can seek change if that is what they desire (Andreotti & Souza, 2008). While this was less explored in the Matthews (2014) study, I, similar to Sharma and Romas (2012) drew upon this phase.

This phase concerns promoting transformative action by influencing how people think and react (Sharma & Romas, 2012). In my study this related to encouraging participants to reflect on practice from the woman's perspective, the midwife's perspective and the doctors. Within a study by Cheetham & Shen (2003) the three-phase model was used to address the problem of sexual harassment and its possible associations with reproductive and mental health problems. Through a process of critical reflection, participants gained a better understanding of these issues and were asked to identify actionable points to implement in their own lives (Cheetham & Shen, 2003). This study provided me with the idea of using a similar technique within the educational programme – for participants to identify *'Practice Points'* that could help inform and influence practice.

5.14.1 Practice points

This activity was aimed at consolidating learning into a shareable format for knowledge translation and to facilitate conscientization. Participants as a final activity in the educational programme were asked to work as a group to identify and name five key points for practice that could have a personal impact upon a woman's childbirth experience and document these on a template poster to be displayed in their clinical areas. This was described to participants as actions that could be shared with colleagues (i.e. by being detailed on a poster located in a shared area) and individually implemented when caring for women during birth.

5.15 Recruitment of participants to attend the immersive educational programme

Introduction

This section provides an overview of the recruitment process undertaken when identifying suitable participants to attend the immersive educational programme. Study

site and sampling strategy are discussed followed by an introduction to the approach adopted when designing the immersive programme.

5.15.1 Study site

Originally, I had intended to deliver the educational programme in the Trust in which I worked as a midwife. However, the Trust refused due to issues surrounding time for participants to attend stating that the Trust has no capacity to support the delivery of the programme. Thankfully, another local maternity Trust agreed, and I was able to use their facilities for filming and provided with free reign to advertise and recruit midwifery staff (following NHS ethics approval).

5.15.2 Sample strategy

Deciding on a sample in qualitative research is multifaceted and can be unclear, not least because of the intrinsic flexibility and lack of guidance on the process (Coyne, 1997, Englander, 2012). Researchers should be both adaptive and imaginative when designing their sampling strategies, including an open mind to real world conditions that may enable or hinder them. For this stage in the study, the participants needed to be able to relate to what it is like caring for a woman in labour. As a result, participants were identified via convenience sampling through visiting clinical areas and discussing the study with midwives on shift. Convenience sampling is a type of nonprobability or non-random sampling whereby the target population meet certain practical criteria (Etikan et al., 2016). In relation to this stage of the study, criteria consisted of midwives who worked in one particular Trust who delivered intrapartum care to women in labour and was detailed on the participant information sheet as seen in appendix 12.

After gaining ethical approval to recruit on site, I attended the wards personally to engage with staff and to discuss the nature of the programme and what participation would include. If they expressed an interest in attending the programme, I provided them with a participant information sheet. Sampling was undertaken in clinical areas within the supporting Trust where midwives provided intrapartum care both on the high-risk unit and on the birthing centre. It was provisionally agreed between myself and my supervision team that we could only facilitate the session with a maximum of 12 participants due to the number of VR headsets available. Given the nature of the programme it was also felt that a smaller group would help facilitate discussions and dialogue ensuring minimal distractions when engaging in critical debate (Ledwith, 2015).

During this phase, interested parties were also informed on the six week focus group follow up and what this would include. Information relating to the follow up focus group was detailed on the PIS sheet, with potential participants able to ask me any specific questions relating to the programme of the focus group. Those who expressed an interest to take part provided me with their contact information (appendix 13) and were contacted 24 hours following to confirm if they still wished to take part. Once agreed, the date and time of the programme was provided informing them that I would contact them via their preferred method one week before delivery as a reminder.

The nature of the programme gained considerable interest reflected in ease of recruitment. After attending the unit on two separate occasions, I had recruited 12 participants.

5.16 Addressing rigour

Rigour relates to the evaluation of research standards against which all research is measured (Sandelowski, 1986). The key concept to assess rigour in qualitative research relate to *'Credibility' 'Confirmability' 'Transferability'* and '*Dependability*', the four pillars of qualitative research (Lincoln & Guba 1984). Below I describe and discuss how each pillar was addressed in my study.

5.16.1 Credibility

Credibility refers to the degree which the data presented reflects the reality of the participants and is achieved when researchers present descriptions of the human experience that others with similar experiences would instantly recognise (Lincoln & Guba, 1984). This is also enhanced through consistent and regular reflexivity during the research journey (Nadin & Cassell, 2006). In my study a reflexive diary was kept to document thoughts, feelings, ideas and interpretations. Reflexivity is an important aspect of research endeavours encouraging those undertaking research to interrogate their own cultural and conceptual frameworks (Berger, 2015). Reflexivity as discussed and explored by Berger (2015) is concerned with the positions the researcher can occupy and how this can influence the research. In line with the work of Etherington (2004) the reflexive journaling encouraged me to have a constant level of consciousness as to what interpretations and decisions were being made, thereby helping to ensure credibility in my research endeavours (Lincoln & Guba, 1984).

Credibility was also achieved through collaborative data analysis via regular consultations with my supervisory team, both during the collection and analysis of data exploring women's experiences and during the analysis of data collected from the evaluation phase of the immersive educational programme. Van Manen (2016) believes that these collaborative analyses are helpful in gaining a deeper understanding and meaning generated from the data. The findings of my study have also presented at a range of academic conferences enabling me to gain critical feedback, and to assess the extent to which my findings rang 'true' with others.

5.16.2 Confirmability

Similar to the positivist standard for rigour, objectivity, confirmability is otherwise known as the assurance that interpretations and descriptions of a given phenomenon are undeniably grounded in a specific social context. Confirmability relates to research findings being the product of a robust approach and the adoption of suitable methods during the research process, and not the biases held by the researcher (Lincoln & Guba 1984). Ensuring confirmability in this study required me to demonstrate that the data and interpretations were rooted in real life circumstances and contexts (Denzin & Lincoln, 1994). These considerations aim to ensure the participants (women during interviews and maternity professionals attending the educational programme) reported accounts are not, *'figments of the inquirer's imagination'* (Schwandt, Lincoln & Guba, 2007, p. 299). The discussion and presentation of findings during supervision meetings and academic audiences helped achieve confirmability. Note taking during the delivery of the programme also aided confirmability. I have used a wide range of quotes within the presentation of my findings so that all interpretations are substantiated. A clear audit trail also enhances the confirmability of research (Houghton et al., 2013) and was adopted during each stage to ensure rigor in the research process.

5.16.3 Transferability

Equivalent to the positivist standard for rigour, generalisability, transferability in qualitative endeavours refers to the applicability of this study's findings to wider, similar populations and contexts (Polit and Beck, 2012). In essence, transferability is the extent to which the findings are transferable to the wider context outside of the research and to what degree the findings are meaningful and applicable to the wider literature (Sandelowski 1986). Transferability also allows researchers to question the relevance

their study has and how their findings may influence future research (Houghton et al., 2013). For constructivist enquiry, the situational uniqueness of a particular phenomenon offers an inherently challenging task in transferring study findings to wider populations / contexts (Krefting, 1991). The researcher is consequently only able to make claims regarding the local meaning of the findings, assumptions regarding transferability of study findings is made by the reader of the research, basing their opinions on sufficiently thick descriptions of the data (Lincoln and Guba, 1985).

Transferability in the context of this study was enhanced by exploring and presenting findings that disconfirmed themes and interpretations and ongoing discussions with my supervision team to ensure all meaningful data was represented (Lincoln & Guba, 1989). As a product of methodological rigor, the aims and objectives of this study reproduced the development of an educational programme, underpinned by the thick descriptions gathered from empirical and secondary data stages. Within this study, thick descriptions provided sufficient information to assure the reader of the 'fittingness' of the study, ensuring that the data was represented authentically within the educational programme. As a result, the study's findings are transferable to other contexts beyond those integrated within the boundaries of the study (Sandelowski, 1986, p.32).

5.16.4 Dependability

Parallel with the positivist standard for rigour, reliability, dependability provides the research endeavours with the assurance that the processes adopted a logical, predictable development, proportionate with specified epistemological, theoretical and methodological approaches and methods (Lincoln & Guba, 1984). Dependability stresses the need for researchers to account for the ever-changing context of the research (Polit & Beck 2004). In my study I aimed to ensure that methods and related decisions were documented and externally verifiable, thereby enabling the reproduction of the study by others (Jensen, 2008, Given & Saumure, 2008). Due to the nature of qualitative inquiry, precise replication of findings was not possible within this study. This is due to the ever-changing context and circumstances determining the lived reality of participants at the time of the study (Hammersley, 2003, Francis & Hester, 2004). However, to provide a rigorous account that may yield similar findings, an audit trail of all research practices and steps taken during each phase of the study has been provided, as suggested by Koch (1994). This approach ensures the processes of data

collection, data analysis, and theory generation are of high quality and transparent with clear descriptions and rationale provided for all methodological decisions (Polit & Beck, 2004).

5.17 Methods for evaluation of the educational programme

When researchers are engaged in systematically and empirically examining the effectiveness of interventions, through careful data collection and thoughtful analysis, one is said to be engaged in evaluation research (Creswell & Clark, 2017). Evaluation uses many of the same methodological approaches used in traditional social research, yet evaluation usually takes place within an organisational context. During searches to identify literature concerned with evaluation methods, many studies were identified focused on evaluating interventions within a healthcare context, providing me with a plethora of studies from which to draw from. One study recommended that when evaluating education resources in healthcare, facilitators should not rely on a single assessment tool, rather the method of assessment should include a measure of skill, knowledge, behaviour and attitude (Wilkes & Bligh, 1999). Within the literature, questionnaires were highly favoured as a way of evaluating the effectiveness of educational programmes (Rattray & Jones, 2007). It was decided that pre and post questionnaires that included the approaches recommended in Wilkes & Bligh (1999) would allow for assessments to be made on skill, knowledge, behaviour and attitudes.

5.17.1 Pre and post questionnaires

Drawing upon Patten's (2016) guidance on questionnaire design, pre and post questionnaires were devised (appendix 15 and 16). Questions included in both questionnaires involved exploring midwife's knowledge of birth trauma and PTSD to allow for evaluation of the educational programme on their learning. The current level of training in relation to birth trauma and PTSD was explored in the pre questionnaire only. Free text sections were also included in the post questionnaire to explore participant's thoughts and feelings on the use of VR during the programme, and recommendations for development. It felt important to capture open responses to allow exploration of the acceptability and experiences of VR, as well as exploring the possible potential for further development in wider areas. Notes were also taken on the day of the training/evaluation to capture key responses and issues that could help develop the programme further. Questionnaires were anonymised ensuring that responses could

not be identifiable yet each pre and post questionnaire completed by each participant was assigned a separate number to allow comparisons to be made for each individual's responses. Findings from the evaluation phase are presented in chapter 8.

5.17.2 Focus group follow up

It was originally intended that all participants who took part in the evaluation of the educational programme would participate in a focus group. A focus group is a flexible, dynamic interactive method of data collection where discussion is stimulated amongst participants (Kitzinger, 1995).

It was planned that the focus group would take place approximately 6 weeks following the educational event. A focus group question guide (appendix 18) was devised with questions designed to explore the potential impact of the programme on participants own experiences of caring for women, as well as sharing the identified practice points in practice. Unfortunately, it became impossible to coordinate an event for everyone, or even a small group to participate; largely due to work commitments. Following a discussion with the supervisory team, it was therefore decided that we would ask participants if they would be willing to share their experiences via email. The focus group questions were shared electronically, with five of the participants providing feedback. Comments and responses from the feedback are presented in chapter 8 section 8.15.

5.17.3 Data analysis

The scores from the likert scale responses on the pre and post evaluation questionnaires were analysed descriptively. A basic thematic approach (Braun & Clark, 2006) was used to analyse the qualitative comments (i.e. open text comments on questionnaires, and 6-week feedback data). This involved reading and re-reading and grouping the data into meaningful headings/sections. All analytical findings were discussed and shared within the supervisory team.

5.18 Ethics

Ethics clearance for phase 1 to undertake empirical interviews was gained from the Science, Technology, Engineering, Medicine and Health (STEMH) ethics committee on the 26th of September 2016 seen in appendix 19. NHS ethics was not required as women were recruited away from NHS premises. Ethical clearance for phase 2 to enable me to deliver the educational programme within an NHS Trust with maternity staff included both clearance from the STEMH ethics committee on the 1st of February 2018 (see

appendix 21) and ethical clearance from the Health Research Authority, on the 28th February 2018 (appendix 20) following a proportionate IRAS (IRAS number 238226) application.

5.18.1 Ethical principles

When undertaking any research, ethical issues need to be considered. In healthcare research there are four guiding ethical principles that need to be addressed; beneficence, non-maleficence, justice and autonomy (Beauchamp & Childress, 2001). *'Justice'* obligates researchers to ensure costs and benefits are fairly distributed amongst those affected by the research and/or findings. *'Respect for autonomy'* relates to informed consent and acknowledging the capacity of participants to make meaningful choices. *'Beneficence'* and *'non-maleficence'* are the two overriding principles that define moral conduct in research. Beneficence is action that is done for the benefit of others. In healthcare this principle often clashes with the principle of respect for autonomy when the patient makes a decision that the healthcare professional does not think will benefit the patient. Non-maleficence means to *'do no harm',* so to avoid unnecessary harm to research participants. This principle can sometimes be problematic in healthcare research as e.g. interventions may cause harm, be it with side effects to drugs, or invoking emotional issues via asking participants to recount an emotive experience.

Considerations of the four ethical principles detailed above, urges researchers to think deeply about their methods, patient benefit, potential harm and ensuring that beneficence and non-maleficence are considered throughout (Beauchamp & Childress 2001). Below I demonstrate how the four-core underpinning ethical principles were adhered to in my study.

5.18.2 Ethical considerations

All participants in each phase were provided with a detailed information sheet which provided them with full details about the aims and purpose of the study and asked to sign a consent form prior to collecting any data ('*respect for autonomy*'). Plain language was used within the information sheets to ensure barriers related to reading ability was considered. I also offered to read the information sheet to eligible women should this be required. This promoted 'justice' within the study by ensuring that all women who showed an interest were provided with equitable levels of information regarding

participation. The information sheet addressed ethical issues regarding the voluntary nature of participation and that they were able to withdraw their data from the study up until one month after the interview had taken place ensuring I was respecting participants *'respect for autonomy'.*

In regard to midwifery participants, the midwives were informed of the sensitive nature of the programme (non-maleficence) and each participant was given equal opportunity to attend (justice) by running the programme over a lunchtime period, and providing food. This meant that I was able to minimise burdens on the healthcare professionals and ensuring that patient care was not compromised (beneficence).

Attention to beneficence and non-maleficence was addressed by making participants aware (i.e. within the participant information sheet) that if concerns of poor practice were raised, that these issues would need to be addressed. This could involve encouraging midwives to utilise established processes within their workplace, or directly women into the appropriate Patient Advice and Liaison Service. If any issues regarding unsafe practice were disclosed, as a registered practitioner I had a duty of care to report such issues in line with my NMC code of conduct (NMC, 2018). It was also stipulated prior to taking part that participants should refrain from mentioning names when discussing care/experiences and to adhere to data protection and confidentiality. Midwives were made aware that as part of the educational programme an exercise would be required that encouraged them to take their points of learning to share with others in their areas of practice therefore confidentiality regarding their involvement was not possible. However, it was stipulated that all reported data (i.e. from questionnaire and focus group responses) will be anonymised, and no-one with be able to identify them from the responses provided (non-maleficence).

Confidentiality and anonymity (non-maleficence) was upheld by using codes in place of names on all key documentation (e.g. consent forms, personal demographic sheet, contact information sheet and interview data). With participant numbers rather than names used in the reporting of the findings to protect anonymity. All electronic data was stored on password protected University computers and hard data was kept on university premises in a lockable filing cabinet.

Considerations were also given to the principle of justice and the balance between beneficence and non-maleficence when addressing the possibility of emotional upset when women recounted their distressing/traumatic birth experience. The first consideration related to the sensitive nature of the topic and how this may cause distress to women and the researcher. To ensure this was dealt with appropriately, steps were taken to ensure harm was minimised. This was achieved by referring women to appropriate services/staff if they wished to access further support for their birth, such as the Supervisors of Midwives team for debriefing services. However, it should be noted that all women declined the need for help following interview, and rather they expressed the therapeutic nature of talking over their experiences in a safe and supportive space.

5.19 Conclusion

This chapter has outlined the methodological frameworks adopted in this study contextualised by the critical theoretical approach adopted. A detailed description of the use of a three-phase critical pedagogical framework to design the educational programme is provided. Ethical considerations have been presented and discussed, and finally I consider how a trustworthy and rigorous approach has been achieved by drawing upon existing literature concerned with methodological rigor. The next chapter presents findings from the interviews identifying the key triggers for birth trauma amongst vulnerable and disadvantaged women. Reflections on the process recruitment process are also offered.

CHAPTER 6. FINDINGS

6.1 Introduction

This chapter presents the findings from the empirical interviews. First, I provide the participant characteristics followed by personal reflections on recruitment and data collection stages. I then present the findings from the inductive (key triggers for a traumatic birth related to interpersonal interactions between women and health care professionals) and deductive (mapping the key triggers against the studies within the meta-ethnography) phases of the framework method approach adopted.

6.2 Participants

Mothers who were over the age of 18, lived in the North West of England, met criteria for being classified as disadvantaged and vulnerable and had experienced a previous distressing and/or negative birth in the last three years were eligible to participate. The earliest accounts of birth during interviews was six months postnatal, with the latest three years postpartum. Overall, ten women took part, and the sociodemographic characteristics of all women interviewed are presented in Table 10. The women were aged between 18- 30 years with a mean age of average of 25 years.

Table 10 Characteristics of women interviewed

Interview participant	Age at birth of first child	Nationality	Marital status	In receipt of social security benefits	Highest level of Education	Teenage mother	Existing mental health issues
Participant 1	20	Enlish	Single	Yes	Some high school	No	Yes
Participant 2	21	English	Married	Yes	College	No	Yes
Participant 3	20	English	Single	Yes	College	No	Yes
Participant 4	19	English	Single	Yes	Some high school	Yes	Yes
Participant 5	28	American	Married	Yes	University	No	Yes
Participant 6	21	English	Single	Yes	Some high school	No	Yes
Participant 7	18	English	Single	Yes	Some high school	Yes	Yes
Participant 8	26	Pakistani	Married	No	University	No	Yes
Participant 9	22	English	Single	Yes	Some high school	No	Yes
Participant 10	19	English	Single	Yes	High school	Yes	Yes

In regard to women meeting the criteria for being disadvantaged and vulnerable, 90% were in receipt of social security benefits, three had been teenage parents, seven were single parents and all the women had an existing mental health issue.

6.3 Reflections on recruitment and data collection

Although access to groups within Children's Centres and Sure Start Centres where recruitment was planned ran smoothly, I found that women were quite reluctant to speak to me once I informed them of my role as a practising clinical midwife still working in the area. I sensed women felt they had to act a certain way in my presence. I tried to eliminate this tension as much as possible by being relatable, dressing casual and not wearing any official badges or identification. I spoke to staff to see if they could introduce me to the women at the beginning of the group so that women were aware of who I was and were able to approach me individually if they wished. However, this was not successful. As an alternative I began addressing the whole group by introducing myself and the study. This method was equally ineffective, and on reflection may have been an overly formal way to engage women in conversations about sensitive topics such as traumatic birth experiences. Further reflections on recruitment and data collection are made in chapter 11 section 11.2.

Finally, I began approaching women on an individual informal basis to introduce myself and the study and ask if they would like to talk about their birth experience. Over a period of four weeks I attended four sessions a day which included mother and baby groups, sensory play groups, baby massage and postnatal feeding groups at two different locations. Each session lasted approximately an hour which meant that once I had engaged a woman in conversation, I usually only managed to speak to one or two women per session. The individual approach was the most effective way of engaging women in taking part although this came with its own challenges such as the length of time involved in conversations. This was partly due to my midwife role in that once engaged in discussions with women who had recently had a baby, I ended up in in-depth dialogue on topics that were not relevant to the study, i.e. feeding or sleeping issues. This caused a moral dilemma as while I felt obligated to give women advice or support, I was also mindful that I wished to recruit women who met the eligibility criteria for my study. The tensions I faced as a health care professional undertaking research in my own setting are raised within the wider literature (Asselin, 2003, Dwyer et al., 2009). I found

myself having to challenge my negativity and worries about time constraints knowing that I helped a woman with whatever query or challenge she was facing as a new mother.

After three weeks of attending sessions, I discussed time constraints and recruitment issues with my lead supervisor who suggested attending at the end of each session and providing a brief introduction to the group. It was felt that this would provide women with a choice to either approach me about the study or not after the session had ended. It was also considered that this felt that this approach would be more likely to capture women who wanted to speak to me in direct relation to the study, rather than seeking generic advice while they accessed the group. Once I had adopted this approach, I was able to recruit 10 women for interviews over a four-week period.

One key issue emerged during recruitment related to a lack of participant diversity, particularly in relation to BAME women. This was also particularly apparent after one of the participants from Asian heritage raised some interesting and important insights into her birth experience. Waheed et al (2015) highlights issues faced in research when representing minority groups, stating that researchers should plan to deploy strategies such as involving religious leaders in recruitment, cultural competence training and using incentives for participation. As a midwife used to engaging with women from diverse backgrounds, I had not envisaged this problem. Through undertaking the research, I became aware that women from BAME backgrounds would rarely engage with community services and groups. While this limitation highlights wider inequalities in access and service provision, it also meant that the voices of BAME women are underrepresented in this study. This issue is considered further in chapter 9 section 9.14.5.

6.4 Identifying triggers for traumatic experiences across the data set

Key triggers for trauma within the social space of birth were identified following the application of the framework method detailed in chapter 5. Overall six key triggers were identified. A brief description of each trigger is detailed in table 11, together with how many women made reference to this issue. Mapping the frequency of reporting allowed for a conclusion that *'A lack of emotional support'*, *'Poor use of language'* and *'Judgemental attitudes'* were the most prevalent themes to be reported by women who had experienced a difficult/traumatic birth. In the following sections, I describe each

trigger together with exemplar quotes from the data set and contextualised by wider literature.

Table 11 Themes emerging from the data using the framework method

Themes that emerged (triggers for trauma)	Description	Number of participants who highlighted this issue (n=10)
**A lack of emotional support**	A lack of emotional support refers to how women felt they were not supported emotionally by healthcare professionals.	N=10
**Poor information giving**	Poor information giving highlights how women felt they were not provided with sufficient information and were unsure of what was happening and why.	N=8
**Poor use of language**	Poor use of language related to inappropriate language used.	N=8
**Unconsented interventions**	Unconsented interventions were explicitly and implicitly reported by the women highlighting issues around consent and choice during birth.	N=7
**Submissive interactions**	Submissive interactions related to women being shouted at or not feeling that they were able to voice their concerns.	N=6
**Judgemental attitudes**	Judgemental attitudes related to professionals' negative preconceptions based on women's social, cultural and ethnic backgrounds.	N=9

While the focus of analysis was to identify key triggers for birth trauma, it felt important to capture positive aspects of the data during the analysis stage. These aspects of care were captured during coding and presented in section 6.5.

6.4.1. Trigger 1: A lack of emotional support

A lack of emotional support during birth was a key issue for all women that were interviewed. Not building up positive connections with their midwife made women feel reluctant to express their own feelings and women feeling unsupported in dealing with their emotions during birth. One woman discussed how she supressed her own emotions for the benefit of the midwife after being told to stop making so much noise during labour:

'She [midwife] said I was being overdramatic, I just thought I'm going to have to reign it in and not show her [midwife] that I'm upset, not show my emotions and pretend it's alright, but I shouldn't have had to do that' (Participant 9).

Feeling disconnected and not having meaningful emotional connections with their health care professionals could lead to negative impacts on decision making. Williams (2002) states that not being believed or listened to can prevent an individual from being a participant in trustful conversations that *'steady the mind'*[22]; with untrusting interactions subsequently creating a cascade of negative emotions and beliefs about the self. One woman in my study who had been in labour for 34 hours had informed the maternity team that she did not wish to have a caesarean section. However, due to *'threatening'* interactions with her healthcare professionals, she discussed how her wishes and emotions were disregarded and felt coerced into agreeing to a caesarean section:

'It got really nasty and threatening [during labour] it was all together one of the worst experiences of my life. Eventually we [the woman and the HCP'S] finally agreed to a section, it was a decision that was made out of desperation, I was emotional, I felt like I couldn't take the situation any more' (Participant 4).

Another woman described how her midwife's lack of emotion led her to feeling uncared for:

'There were no emotions with her [midwife], she was just going through the motions, I didn't feel like she cared that I was suffering' (Participant 3).

Feelings of interdependency and personal growth have been identified as positive outcomes of supportive and empathetic interpersonal interactions during birth (Dalberg & Aune 2013). In contrast defensive interactions, dissatisfaction with care and disengagement with services are all noted as possible outcomes of poor interpersonal interactions when accessing healthcare (Lavender et al., 1999, Bohren et al., 2015, Rayment-Jones et al., 2017). Fostering positive interpersonal interactions with women

[22] Steadies the mind – a term coined by Bernard Williams (Truth and Truthfulness: An Essay in Genealogy (Princeton: Princeton University Press, 2002), Williams's proposal is that engagement in mutually reliant, and so mutually trustful, dialogue with others is the chief impetus for this process by which the mind becomes settled.

during childbirth is highlighted as a key aspect to facilitate a positive birth experience (WHO, 2018), yet women within the data set felt these basic needs were not being met.

6.4.2. Trigger 2: Poor information giving

Poor and inconsistent information giving was expressed by eight participants. These women frequently described how a lack of effective communication resulted in women feeling uninformed, uninvolved and unable to make informed choices on care. One woman reported that she been inadequately informed about what an instrumental birth was and how this would be performed. When reflecting upon her experience she stated that had she known what to expect, she would have refused the intervention:

> *'If I had known more about instrumental births I could have made more of an informed choice, but it wasn't a choice, it was more ''we* [the HCP's] *are using forceps, that what's happening'' if I would have known more I would have refused'* (Participant 5).

Women's ability to understand what was happening to them due to poor information giving was also expressed. One teenage mother noted how she did know she was having a forceps delivery until she was in theatre and was asked to push:

> *'I said to her [midwife] why do I need to push if I'm having a caesarean section, she said 'oh no you're going to try to push him out first and we are going to help you', which confused me because I was in theatre so I thought I was having a section, I was just so confused'* (Participant 8).

Another woman expressed her upset during labour in which a lack of information giving about her baby's condition made her worry and ruminate about possible outcomes:

> '*I just remember him [doctor] saying to her [midwife] that its likely she [baby] had been starved of oxygen for some time, I just kept thinking oh god what have I done wrong, all I could think of was that my baby was going to be brain damaged and when I asked they just kept saying don't worry, but I heard them say it, I thought they was hiding something from me'* (Participant 1).

Poor information giving also related to how health care professionals would use jargon and language that women did not understand, creating feelings of fear and anxiety for women. Similar to the quote above, one mother reported how a lack of appropriate

information giving when her baby was showing distress resulted in her fearing for her baby's life:

> '*She kept saying to the doctor that the CTG was suspicious and when I asked her what was wrong, she said that she [baby] was compromised, then she ran out to get the doctor, I thought my baby was going to die'* (Participant 10).

6.4.3. Trigger 3 Poor use of language

The use of negative and judgemental language by midwives was highlighted by eight of the women interviewed. Negative and disempowering statements can have a detrimental effect on a woman's ability to make rational decisions during a vulnerable state (Wilmore et al., 2015). Negative language is reported to also have a detrimental effect on women's sense of self highlighting issues relating to poor language and inverse care in midwifery practice (Kirkham et al., 2002, Hunter, 2006, Burns et al., 2016). One woman expressed how she was referred to as *'being soft'* (Participant 4) by a midwife when she sought pain relief during labour. Another woman believed her prolonged birth and subsequent instrumental delivery was a result of her inability to '*push hard enough'* after being told by a midwife that she needed to '*pull it together for the baby's sake'* (Participant 9). These negative and disempowering statements impacted upon her women's sense of self- efficacy during childbirth.

One woman expressed how during birth the language used by the midwife was undermining and made her feel like a failure when she required an episiotomy that caused considerable issues postnatally:

> ''*Come on you need three good pushes, harder, harder, no you're not pushing into your bottom, baby is getting tired one more push and if it's not good enough I'm going to have to make that cut', horrible, I don't like thinking about it to be honest, I just think if I'd have pushed harder, I wouldn't have needed to be cut'* (Participant 7).

6.4.4. Trigger 4: Unconsented interventions

Seven women's negative accounts of care included unconsented interventions and feeling of coercion during birth. One woman discussed how she felt the staff had performed vaginal examinations unnecessarily:

'She [midwife] had just done one and it was agony, I had to tell her to stop. Then the doctor came in and told me I had to have another one [vaginal examination] so she could check or else I would need to have a caesarean section, I didn't see the point as the midwife had just told her I was 5cm' (Participant 10).

Another participant expressed her distress of having an unconsented episiotomy and requests for the procedure to stop:

'They give me the episiotomy I didn't want then that consultant woman was literally arms in trying to stretch me to get him out, I was begging her to stop but she didn't' (Participant 9).

Unconsented interventions ranged from vaginal examinations (as highlighted above) to a cannula being inserted without consent:

'I was scared anyway, there was so many people in the room rushing about getting me ready to go to theatre, people asking me questions, the next thing I felt a sharp stab in my arm, I looked down and there was blood everywhere, I didn't even know she was putting one in (a cannula), I panicked because it was all over the floor' (Participant 4).

One woman (who had had a negative forceps delivery in her previous birth) expressed her upset at feeling coerced into having a forceps delivery despite informing staff throughout her pregnancy that she did not wish this to happen:

'*I had told them all the way through that I rather get put to sleep than have forceps, anything but that. They told me I had no choice, its forceps or risk my baby being dead, apparently it was too late for a caesarean, I had no choice in the matter'* (Participant 2).

6.4.5. Trigger 5 Submissive interactions

Submissive interactions related to both paternalistic and controlling experiences of care for six of the women interviewed. For example, one teenage mother discussed how she felt she was treated like a child during labour:

'My bum kept shooting off the bed and she kept shouting at me, 'keep your bum on the bed [name]', I didn't like the way she [midwife] spoke to me. I'm not a child, yeah it's my first baby but I'm not a kid' (Participant 9).

Two participants reported feeling like health care professionals were monitoring their fitness to mother based on being single parents who had previously required specialist input from social services. One discussed how she felt obligated to agree with care decisions, irrespective of her wishes, due to concerns of her midwife contacting social services:

> *'The real worry was that they were going to call me a bad mum and start saying that they [HCP's] will get social services involved again because I'm saying I don't want the medication [syntocinon] so I just went with it'* (Participant 2).

When speaking about their experiences these women would express how care felt more like surveillance than support. This was particularly evident for one woman who described how the midwife's interrogations during latent labour following a report of domestic abuse from the police created feelings of psychological submissiveness:

> *'When I arrived she [midwife] didn't even ask how I was, she looked at me and asked me if I had anything to tell her, it was like she was playing mind games with me, it took her about ten minutes to get to the point, then that made me look stupid. Then she started telling me how it's her place to protect my baby, I thought no, it's my place to protect my baby and your supposed to help me figure out how' But I didn't say that* (Participant 7).

Such interactions highlight the detrimental effects that the exertion of power during birth can have on women's experiences.

6.4.6. Trigger 6: Judgemental attitudes

Nine women within the data set spoke of judgemental interactions with their healthcare professionals. These experiences included women feeling that staff held judgemental preconceptions of them based on factors such as their ethnicity, social status and age. Women felt that, in turn, this influenced how they were spoken to and treated:

> '*She [midwife] said to me 'Not many Asian women want to get into the pool', I thought well maybe they don't but it would have been nice to be asked no?'* (Participant 8).

Another woman discussed how she felt being a young mother served as a barrier to receiving pain relief during labour:

'I wanted the morphine but she [midwife] kept saying you're young and healthy and it's your first. She [midwife] suggested a walk up and down the corridors to help the pain and then asked my mum if I'd had paracetamol before' (Participant 9).

A further participant highlighted feeling stigmatized by staff due to her socioeconomic status and the disclosure of drug use prior to pregnancy:

'She said that I should be made of tough stuff coming off the stoops [council estate] then she asked me the last time I took drugs. I did not get it. I was in labour, in agony, being asked random questions, it really pissed me off' (Participant 10).

Such judgemental comments could result in women blaming themselves for adverse outcomes, which in turn exacerbated negative beliefs about themselves. For example, one woman stated that her midwife had told her that her smoking was a contributory factor for her baby's inability to cope with labour, thereby inferring it was her fault that she needed forceps to deliver her baby:

'It's made me think that it's all my fault, it's like she said [midwife] because I smoked, she's small so she wasn't growing properly inside me, so I guess I couldn't even get that right either' (Participant 7).

Ascribing traits based on social status, age and ethnicity risks exposed women to a multitude of misinformed judgements. These barriers had a ripple effect in which women were then unable to build respect-based relationship with their midwives. Complex life factors were seen to be reinforced during interactions making women feel *'othered'.*

6.5 Positive aspects of the data

While the focus of the interviews was on women's experiences of a traumatic birth, it is important to highlight the positive aspects of care that emerged from the interviews as insights into what made care good were also reported. Women described positive care as informative, caring and genuine, and used positive terms when describing midwives. These terms included '*calming*' (Participant 8), '*clued up*' (Participant 1) '*genuinely*

interested' (Participant 9) and '*friendly*' (Participant 4). Positive experiences also related to health care professionals listening to women without judgement, which in turn made women feel more in control and empowered:

> '*When she came back not the room, I felt calmer, I trusted her, she knew what I wanted and I could see she was fighting for me, it gave me the strength to speak up'* (Participant 2).

For four participants their positive experiences related to birth that predated their traumatic childbirth experience. These insights emphasise the benefits of a responsive and emotionally intelligent approach to caring for women throughout their childbearing experience, discussing this further in chapter 9 where I offer recommendations for practice.

6.6 Mapping key triggers within papers identified in the meta-ethnography

As discussed in Chapter 5 section 6.6, the next stage in the framework approach was to use these triggers as a deductive lens. This stage involved mapping the triggers against the studies included within the meta-ethnography (see Table 12). The purpose was to assess the importance of these issues across other women's accounts, and to provide validation and justification to use these triggers to inform the critical pedagogy.

Table 12 Triggers mapped against papers included in the meta-ethnography

	**Themes**					
**Papers**	**A lack of emotional support**	**Poor information giving**	**Poor use of language**	**Unconsented interventions**	**Submissive interactions**	**Judgemental attitudes**
1 – Briscoe & Lavender (2009)	Yes	No	Yes	No	No	Yes
2 -Ny et al (2007)	Yes	No	No	Yes	Yes	Yes

3 - Reitmanova & Gustafson (2008)	Yes	Yes	Yes	No	No	Yes
4 – Davies & Bath (2001)	Yes	No	Yes	No	Yes	Yes
5 - Cross-Sudworth et al (2011)	No	Yes	Yes	No	No	Yes
6 – Bailey et al (2004)	Yes	Yes	No	Yes	No	Yes
7 – Ebert et al (2014)	Yes	No	No	No	Yes	Yes
8 – Herrel et al (2004)	Yes	Yes	No	Yes	No	No
9 - Price &Mitchell (2004)	Yes	No	Yes	No	Yes	Yes
10 – Arthur et al (2007)	Yes	No	Yes	Yes	No	Yes
11 - Robb et al (2013)	Yes	No	No	Yes	Yes	Yes
12 – Jomeen & Redshaw (2013)	Yes	Yes	Yes	No	No	Yes
13 – Shafiei et al (2012)	Yes	No	No	Yes	Yes	No
14 – Spidsberg (2007)	Yes	Yes	No	Yes	Yes	Yes
15 – Ward et al (2013)	Yes	No	Yes	No	No	Yes
16 - Wilton & Kaufman (2001)	Yes	Yes	Yes	No	No	Yes
17 Howard (2015)	Yes	No	Yes	No	Yes	Yes
18 – McLeish & Redshaw (2018)	Yes	Yes	No	Yes	No	Yes
Papers identifying trigger	**17**	**8**	**10**	**8**	**8**	**16**

Overall this deductive based assessment revealed that all triggers were evident in this wider body of literature, and similar to what I found within the interview data set, the most recurring issues related to *'A lack of emotional support'*, *'Judgemental attitudes'* of health care professionals and *'Poor use of language'*. Although these issues were most prevalent, as all the issues frequently occurred in women's negative accounts of birth, it was considered important that all triggers were embedded within the filmed scenario.

6.7 Conclusion

The interview data provided invaluable insights into disadvantaged and vulnerable women's experiences of childbirth on a local level, with these findings then assessed against those from the wider literature. Similar to the findings of the meta-ethnography, women-provider interactions directly affected the cognitive and emotional stability of women through the childbearing continuum. The data highlights how social, cultural and attitudinal factors play an important role part in how health care professionals interact with more marginalised women, and to what extent care during labour is tailored to meet the needs of these vulnerable populations. This illustrates how social structures and class divides can add an extra layer of pressure and complexity to women who start off from a position of disadvantage; which is then exacerbated throughout pregnancy, birth and in the postnatal period as a result of poor interpersonal interactions.

How these triggers were used to inform development of the filmed scenario script are presented in the following chapter.

CHAPTER 7: DEVELOPING AN IMMERSIVE EDUCATION PROGRAMME

7.1 Introduction

The previous chapter presented the findings from empirical interviews exploring traumatic birth experiences amongst disadvantaged and vulnerable women within a locality. This chapter leads on from empirical stages, detailing the development of the evidence-based script delivered via VR as part of the immersive educational programme, and the wider content to be used within the educational programme. This chapter begins by discussing the script development followed by a detailed description of the filming process. A table is then presented mapping key triggers to each scene within the film. Image stills from the finalised film are then presented alongside a synopsis for each scene. I conclude by providing an overview of the educational programme, mapped to the three-step model of a critical pedagogy as detailed in chapter five section 5.16.

The findings of the evaluation of the delivery of the immersive educational programme are presented in the following chapter (chapter 8).

7.2 Script & filming. Mapping triggers for trauma within the narrative

When creating a narrative for the film it was important to develop a familiar and relatable scenario to enable viewers to engage with the content, both emotionally and professionally. Impactful visual and/or audio narratives are recommended in place of written text when attempting to enhance emotional responses during data presentation (Flemming et al., 2018). As discussed in chapter five, actors were hired to ensure the scenario was professionally executed and able to convey emotional cues as intended. Key triggers identified and discussed in chapter six were used, ensuring the script was representative of women's experiences. The aim was to illustrate power relations between the woman (named 'Emma') and her health care providers during birth. No direct quotes from the interviews were used in the script, rather the reported issues were used to create the evidence-based content. For example, women experiencing '*Poor use of language*' was depicted by the healthcare professionals making negative comments about the women's lack of progress. *'Submissive interactions*' was contextualised by a woman having her legs in stirrups with a light shining between her legs during an examination. In these occasions the key trigger is identifiable, although

it was recognised that other text, or situations could represent these same issues. Within the script, I and the colleagues I had working with me (i.e. supervisors, actors, casting director), had to ensure that what was presented was likely to stimulate an emotional reaction to aid learning from a first-person perspective.

7.3 The script

Swain and Swain (1988) state that every film's pattern of organisation is built on the same two underpinnings: a topic and a point of view. In this context the topic is a traumatic birth (underpinned by empirical findings), and the point of view is that of a disadvantaged or vulnerable woman (enhanced by filming from a first-person perspective). The script followed the principles of script writing discussed in chapter 5 section 5.12.3 with the finalised script presented in appendix 10.

7.4 Filming

Filming took place in a delivery room at the local hospital. As this was a large size room there was enough space to capture the whole birthing space, which was needed for the 360 view. The birthing room was a typical labour room usually used for women and babies with complications. This meant it had all the fixtures and fittings needed to convey a simulated scenario for a woman in labour. It was felt that a hospital birthing room provided the scenario with an impersonal feel, as opposed to using a low risk birth setting such as a birthing unit or a home environment. Filming in a low risk birth centre would also not have been representative of the stories of the women I collected, nor the experiences of most women who birth in hospital in the UK[23]. Image 2 titled *'The birthing room'* shows the room used. The room was spacious, with a typical birthing bed used on a delivery suite with stirrups attached for instrumental deliveries and for suturing if needed. The CTG machine as seen in image 2 are routinely used for women on the delivery suite and enable a continuous monitoring of baby's heartbeat if needed. While CTG can be helpful to detect risk, it can restrict free movement and all of women who were interviewed referred to the use of this technology during their labour.

[23] Most births (87 per cent in 2012) take place in obstetric units, with 11 per cent in midwife-led units and 2.4 per cent at home (National Audit Office, 2013).

Image 2 'The birthing room'

7.5 Directing the day

Directing the film had to be undertaken via an iPad in the ensuite bathroom of the hospital room (see image 3). To enable the cameras to capture the whole 360 view of the room, the directing team (myself, my main supervisor, the director) needed to be out of the field of view. Between takes, the director made suggestions to the actors regarding pose, speech and emotional cues to emphasize specific triggers embedded within the script.

Image 3 Observing and directing

7.6 The film

The six key triggers for birth trauma identified in chapter six were used to develop the script. In table 13 an overview of each key scene of the film is presented together with the specific trigger(s) and an extract from the script to provide examples of how the triggers were being conveyed.

Table 13 Triggers for birth trauma mapped to each scene of the film

Scene	Trigger	Exemplar extracts from the script
**Scene 1**. Introduction to the film	No specific triggers are expressed. The intro scene acknowledges the funders and the title of the film '***Emma's story'.***	No script within this scene. 30 second white screen with acknowledgments allowing viewers to settle into using the headset and prepare for viewing.
**Scene 2**. The midwife holding a discussion with the woman following a vaginal examination	Triggers - Poor information giving / Poor use of language / A lack of emotional support	**Midwife** (when providing feedback to Emma about her progress) *'Hmm, (removing gloves) you're still 5cms [disappointed face] so the same as the last time I did the examination, you've got a stubborn little one in there haven't you! I'll have to go and inform the doctors but I'll be back in a minute'*
**Scene 3**. Midwife and doctor conversation	Trigger – Poor information giving / a lack of emotional support	**Midwife** (in discussion with the doctor – and not including Emma in the conversation) *'There has been no progress since the last VE she's still 5cm, no decent of the fetal head. Not really coping well with gas and air but refused an epidural and diamorphine as she has a fear of needles. She also had a temperature of 37.8 on her last observations'*

**Scene 4**. Doctor discussing the woman's progress with the woman	Trigger – Submissive interactions / poor information giving / Poor use of language / unconsented interventions	**Doctor** (standing over the woman who is lying prone in the bed) '*Now I know you wanted to aim for a natural birth without intervention, but I feel now is the time that we need to intervene and look at maybe putting up a drip to speed things along. All it is is a little needle in your hand and you will be attached to a drip, the midwife will be in control of the rate and we ideally want to strengthen those contractions and hopefully see some changes at the next vaginal examination'*
**Scene 5**. Cannulation and drop of fetal heart	Trigger – Unconsented interventions/ A lack of emotional support / Submissive interactions	**Midwife** (in response to Emma stating that she wants to 'push') *'what's that? You want to push? It will just be baby pressing against your back passage, your baby is back to back so you feel it a lot more, just keep going on that gas and air, deep breaths through that pain, that's it, that's it, well done see. Now you can use that gas and air whilst I put this cannula in your hand it will take your mind off it'*
**Scene 6**. Reviewing the CTG	Trigger – Poor information giving / Poor use of language / Submissive interactions	**Doctor** (conveying differences in information provided to Emma and the midwife) *'Hello, me again I've just come in to look at this trace seems your baby is being a little naughty [looking at the woman] – [turns to the midwife], 'Hmm, yeah that's a couple now, atypical, prolonged and the variability is not great how have the movements been?'*
**Scene 7**. Vaginal examination	Trigger – Unconsented intervention / Poor information giving / A lack of emotional support / Submissive interactions	**Doctor** (in conversation with midwife, and not including Emma in decision making) *[Turns to the midwife whilst performing a vaginal examination] 'Yep, still 5 and the head is really high, so I think we need to look at our next move as baby is getting a little tired – I'll just have to go and speak with my consultant about a plan'*
**Scene 8.** Decision for a caesarean section, preparing for theatre	Trigger – A lack of emotional support / Poor information giving / Poor use of language	**Midwife** (conversation made at a highly vulnerable point – when Emma is being prepped for a caesarean) *'if you would like someone to be with you then you're probably best calling them now, we will be in theatre in the next half hour'*
**Scene 9**. Alone in the room – end of scene.	Trigger – A lack of emotional support	**Emma** repeating to self *'Its ok its ok, (crying) its ok'*

In the following section a visual still for each of the key scenes is presented, together with a summary of the content. This film can be viewed following the YouTube link in the footnote[24].

7.6.1 Scene 1: Introduction to the film

At the start of the film the viewer is presented with the screen seen in image 4 for 30 seconds. This was to ensure the viewer had enough time to get used to the headset and adjust any straps or headsets ready for viewing the scenario. This was useful and allowed me time to assist each of the midwife once they the film began. The introduction screen acknowledged the funding body and my host academic institution.

Image 4 scene 1 synopsis

7.6.2 Scene 2. Summary of content. Discussion with the midwife following a vaginal examination.

Following the introduction, the viewer is then immersed into the film from the woman's (Emma's) perspective, where they are wearing a hospital gown and lying on the hospital bed. The overhead clinical light is shining in the viewer's point of vision. The midwife then moves the light so that the person viewing (i.e. Emma) can visualise the room around them. This was purposefully scripted to give a visual representation of the medicalisation of the birthing process. The midwife speaks with the woman discussing findings from the vaginal examination that she has just performed. The midwife then informs the women she is going to get the doctor to come and review her care. The woman askes the midwife for more information, clearly expressing that she does not understand the information that the midwife has given her.

Image 5 scene 2 synopsis

7.6.3 Scene 3. Summary of content midwife and doctor conversation.

On return from getting the doctor, the midwife and doctor do not make eye contact with Emma and make their way to the bottom of Emma's bed and stand with her notes next to the computer. This visual representation highlights the impersonal care that

[24] Please follow the link for viewing on the screen or via google cardboard headsets if you wish to view the film in 2D format. For full 360 immersion a VR headset is required: https://www.youtube.com/watch?v=XRbxYK0i2oA&feature=youtu.be

women report in which they felt disengaged from discussions and conversations about their care. Audibly the viewer can hear the conversation in which the midwife and doctor talk Emma's progress and care decisions, but without involving her in the discussion.

Image 6 Scene 3 synopsis

7.6.4 Scene 4. Summary of content. Discussion with doctor.

In this scene, the doctor is positioned over Emma, and using a patronising tone while informing her about the need for labour to be induced to speed up the process. The doctor is leaning over and speaking down to Emma which represents the felt submissive interactions that woman frequently report when describing their traumatic birth. This still was designed to depict power differentials within the birthing environment in which women may feel a loss of control and vulnerability during birth. During this scene the midwife can be seen at the other end of the room on the computer, thereby representing a lack of emotional support and advocacy when the doctor is giving information to Emma whilst in a vulnerable state.

Image 7 Scene 4 synopsis

7.6.5 Scene 5. Summary of content. Cannulation and reduction in fetal heart rate.

This scenario highlights the issues of unconsented interventions and submissive interactions. While Emma informs the midwife that she feels as though she wants to push, the midwife dismisses these feelings thereby highlighting submissive interactions in which Emma's voice is ignored. The midwife continues to insert a cannula despite acknowledging that Emma has a fear of needles. The midwife uses paternalistic language and continues to site the cannula regardless of Emma's evident pain during the procedure.

The still below shows the midwife looking up at the CTG monitor. The midwife is interrupted by the sound of the fetal heart dropping whilst attempting to cannulate Emma. Her body language and facial expressions signify that something may be wrong, conveying a sense of panic. While Emma asks what is wrong, the midwife hurriedly leaves the room with no explanation thereby reflecting a lack of emotional support and leaving Emma evidently worried about the safety of her baby.

Image 8 Scene 5 synopsis

7.6.6. Scene 6. Summary of content. Reviewing the CTG.

During this scene, and depicted in the still below, the midwife and the doctor examine the CTG trace while standing beside Emma. Poor information giving is depicted in this scene in which the midwife and doctor fail to provide Emma with clear information regarding the significance of the CTG findings. The language used is clinical and complicated by the use of medical jargon, thereby serving to isolate Emma from her care decisions. The spatial dynamics designed to depict the professionals power and authority and Emma's consequent feelings of submissiveness and passivity.

Image 9 Scene 6 synopsis

7.6.7. Scene 7. Summary of content. Vaginal examination.

Following the doctor and midwife discussing the trace, a decision is made to perform a vaginal examination (VE). The VE is unconsented as the doctor does not provide Emma with adequate information to make an informed choice. Emma can be seen with her legs in the stirrups providing the viewer with a simulated feeling of being in a submissive position. Using stirrups for performing VEs was reported in the interviews as making women feel exposed, vulnerable and uncomfortable. During the VE the doctor tells the midwife and not Emma what the findings are, thereby emphasising the objectifying experience of a VE. The midwife in the scene is stood at the bottom of the bed shining the light to help the doctor perform the procedure, as opposed to providing emotional support to Emma.

Image 10 Scene 7 synopsis

7.6.8. Scene 8. Summary of content decision for a caesarean section, preparing for theatre.

Following the VE the doctor makes the decision to perform a caesarean section with no consultation with Emma (i.e. trigger of unconsented interventions). The midwife then starts to prepare for the operation but does not engage Emma in any meaningful conversation, such as whether she has any concerns, how she is feeling, etc. This highlights how a lack of information can impact on woman feeling disengaged from care. The midwife asks Emma mandatory 'tick-box' questions that are required prior to a caesarean delivery. The midwife asks Emma if she has anyone to go with her to theatre,

and while Emma says ‘no’ the midwife offers no reassurance, reinforcing the poor midwife-Emma emotional connection.

Image 11 Scene 8 synopsis

7.6.9. Scene 9. Summary of content alone in the room. End of film.

This still forms the last part of the scenario. Following the midwife leaving the room to get changed for theatre, Emma is left alone. Emma expresses her fear at being left with no-one in the room supporting her. This scene allows the viewer to digest the scenario whilst looking around the room. Emma is audibly crying and stroking her stomach telling her baby that it was ‘going to be ok’. This scene was conceived to provoke an emotional response in viewers through the experiencing of being left alone in a hospital room, and with no reassurance for a forthcoming major procedure.

Image 12 Scene 9 synopsis

End of Film

Following filming the footage was edited and converted into a 360-video using specialist software as discussed in appendix 11.

7.7 Overview of Educational Programme

In Table 14, I provide an overview of all the elements that make up the 90-minute immersive educational programme, mapped to the three-step model of developing a critical pedagogy as discussed in chapter five section 5.16. The programme was split into five key stages: VR viewing; discussion of VR content; presentation on birth trauma and PTSD; return to scenario adding additional context to Emma’s story and discussion; ‘practice points’ group work. In Table 14 these five segments are described and linked to the stages with the three-step model (Matthews, 2014). Additional evaluation elements to the programme are discussed in chapter eight.

Table 14 Overview of the immersive educational programme mapped to the three-step model of designing a critical pedagogy

Segments of the educational programme	Description of the activity	Links to three step model (Matthews, 2014)

VR scenario viewing	Participants were supported in the use of the VR google cardboards and how to view the scenario. The VR scenario lasted 7 minute and can be viewed following the link within the footnote on p. 140.	**Listening and naming**<br>This segment links back to section 4.4.6 focused on the development of culture circles and how revisiting a familiar environment with a critical eye allows participants to open up dialogue on dehumanising aspects of a scenario. In the case of this study; a birthing scenario.
Discussion	This activity included reflecting upon the VR scenario. Participants were encouraged to reflect on the scenario from the woman, midwife and doctor's perspective using the SHOWED model as presented and discussed in section 5.13.	**Dialogue & Reflection**<br>Through dialogue in education, people can name, interrogate, and re-imagine their reality (Nyirenda, 1996). Dialogue in this section encouraged a deeper look at the social space of birth and factors that impact upon women's experience and the potential for birth trauma. This included discussing care practices from different viewpoints to facilitate critical consciousness.
Presentation on birth trauma and PTSD	The delivery of a presentation aimed to address issues associated with the development of birth trauma and PTSD, including information on contributory factors that can place some women at a higher risk of experiencing birth trauma and PTSD.	**Listening & Naming**<br>This involved providing evidence from recent reviews into birth trauma and PTSD onset, including information on incidence, prevalence risk factors and symptoms (Alcorn et al., 2010, Grekin & O'Hara, 2014, Thomson & Downe, 2016, Ayers et al., 2016, Yildiz et al., 2017). Empirical quotes from both the meta-ethnography and the empirical interviews were presented to participants at this stage.
Return to scenario adding additional context to Emma's story and discussion	This section included adding context to the scenario. Participants were given additional information about Emma in the scenario as listed in chapter 5 section 5.13.1. Participants were then encouraged to discuss interactions within the scenario that may have impacted upon Emma's experience.	**Dialogue and reflection**<br>Dialogue and reflection at this stage in the programme aimed to critically reflect upon the situation once participants held more information about the personal circumstances of 'Emma' within the scenario. This encouraged a reflection on practices that may cause situations of trauma for women who may have complex life situations.
'Practice points', group work	During this activity participants were given an A1 sheet as seen in Appendix 17. This was a template used to document agreed practice points so participants could display in their clinical areas, encouraging discussion anda change in working practices. Practice points consisted of 5 actionable points they felt would impact positively on a woman's birth experience.	**The promoting of transformative social action**<br>Wallerstein and Bernstein (1988) describe this step of a critical pedagogy as a group effort to identify problems, through the process of critically assessing the social and historical roots of the problem to be addressed and then developing strategies to improve current practices. The aim of the practice points was to facilitate and encourage activated and informed, critical practitioners. This section of the programme facilitated this by working together to develop actionable points that could be undertaken on a personal level and be shared with others.

7.7 Conclusion

The script has been linked to key triggers identified in secondary and empirical accounts to demonstrate its evidence-based content. I have provided a detailed description of the script and the filming of the scenario. As discussed in chapter five this finalised film was embedded within a critical pedagogy as a tool for knowledge translation and reflection. The next chapter will present findings from the delivery of the immersive educational programme, discussing how the filmed scenario was used as a tool for reflection within a critical pedagogical framework, detaining each stage of the programme and how data was gathered when evaluating its acceptability and feasibility.

CHAPTER 8: FINDINGS FROM EVALUATION PHASE. THE DELIVERY OF AN IMMERSIVE CRITICAL PEDAGOGY

8.1 Introduction

This chapter presents findings from the evaluation of the educational programme. I begin this chapter by detailing the running of the day, including information on location, layout and environment. Secondly, I present a table that provides a summary of the evaluation and educational programme components as well as timings to each activity. This chapter then details findings from the pre/post questionnaires together with quotes from participants during the discussions. The practice points devised by participants are also presented. Data presented in this chapter includes participant's reflections on practice, discussions on the VR scenario, critically addressing interpersonal interactions with women and how participants felt using the VR as a method for learning. The final section reports on follow up data collected in relation to how the programme influenced their clinical practice.

8.2 Running the event

The immersive educational programme was delivered in an NHS Trust in the North West of England on the 10th of April 2018. The session was booked six weeks in advance through liaising with the practice education midwife and in close collaboration with the delivery suite coordinator. Fostering relationships within clinicians was important to ensure that delivery did not impact upon clinical practice, as some participants attended during work hours. The programme was delivered in the education centre at the host Trust, booked for 12 mid-day to coincide with lunch time (with food and refreshments provided to the participants). A reminder email was sent to each participant the week before, providing details of the location and time. While twelve had signed up to attend the session, on the day two midwives cancelled due to personal reasons.

There were various elements to the day that included introductions, pre and post questionnaires, as well as all elements of the educational programme (see Table 14). Table 15 builds on Table 14 (chapter 7 section 7.7) by combining the evaluation and educational components to detail all the activities and timings, mapped to Matthews (2014) three-step critical pedagogical model.

Table 15 Evaluation stages mapped to stages within the immersive educational programme

**Segments of the educational programme**	**Time spent on activity**	**Description of the activity**	**Links to three step model of a critical pedagogy (Table 14)**
Introductions	5 MINS	Introductions aimed to ensure participants were clear about the content of the programme and provided them with an opportunity to ask any questions before the programme began. This stage was also an opportunity for people to introduce themselves to one another.	
Consent	5 MINS	Gaining consent was to ensure participation was voluntary and informed, respecting the autonomy of participants informing them they had the right to withdraw at any time.	
Pre-questionnaire	5 MINS	The pre-questionnaire aimed to gather a baseline of participants knowledge of birth trauma and PTSD. The questionnaire also explored their knowledge of risk factors associated with birth trauma and PTSD, alongside exploring their own identified learning needs.	
VR scenario viewing	15 MINS	Participants were supported in the use of the VR google cardboards and how to view the scenario. The VR scenario lasted 7 minutes.	**Listening & Naming**
Discussion	15 MINS	This activity included reflecting upon the VR scenario. Participants were encouraged to reflect on the scenario from the woman, midwife and doctor's perspective using the SHOWED model seen in section 5.13	**Dialogue & Reflection**
Theoretical presentation on birth trauma and PTSD	15 MINS	The delivery of a theoretical presentation aimed to address issues associated with the development of birth trauma and PTSD, including information on contributory factors that can place some women at a higher risk of experiencing birth trauma and PTSD.	**Listening & Naming**
Return to Emma's story adding additional factors	15 MINS	This section included adding context to the scenario. Participants were given additional information about Emma in the scenario as listed in chapter 5 section 5.13.1. Participants were then encouraged to discuss interactions within the scenario that may have impacted upon Emma's experience.	**Dialogue & Reflection**
Practice points group work	10 MINS	Five practice point identified during a group discussion and listed in section 8.12. All points agreed upon through reaching a consensus. Four separate templates were created allowing participants from different areas to have a poster they could display in their own practice areas.	**The promoting of transformative social action**
Post questionnaire	5 MINS	To collect data on participants knowledge, understanding and experiences following participation in the programme. An additional question explored participants views on using VR:<br><br>*'Do you have any thoughts on the use of VR?'* (Appendix 16).	
Closing and any final questions	5 MINS	Participants given the opportunity to ask questions.	

Follow up assessment	Text responses via email	Participants were contacted as part of a follow up assessment as discussed in chapter 5 section 5.14.4. These questions are listed in appendix 18 to explore how the programme may / or may not have influenced practice. This evaluation method also allowed me to explore how participants found sharing practice points with their clinical teams and peers in practice.	

8.4 Participants

In total 10 participants attended the immersive educational training programme. Participants included two student midwives (n=2) (who were also qualified nurses). The remaining participant's clinical backgrounds included both working on the local midwife-led birth centres (n=3), joint research and clinical roles (n=2) and centralised hospital labour ward (n=3).

8.5 Venue

The room that was assigned was large and spacious as seen in image 13. Tables were gathered into a circular formation to ensure the session facilitated a group dynamic and promoted inclusion (Wolff et al., 2015). As a key focus of the session was to encourage reflective discussions, a layout that facilitated this was important. All questionnaires, consent forms and headset instructions were laid out on the table ready for those attending. As time was limited this facilitated the smooth running of the session. The room also included PowerPoint facilities needed to deliver the theory-based PowerPoint on birth trauma and PTSD.

Image 13 Evaluation of programme location and room layout

8.6 Delivering a critical pedagogy, *'Building trust'*

At the beginning of the session I encouraged participated to introduce themselves. I then introduced myself as both a midwife and a researcher and a brief recap of the study as discussed on the patient information sheet. My supervisor Gill Thomson then introduced herself as a researcher with an interest in birth trauma. It was also made clear at this stage that myself and Gill would be taking notes on the day and that information collected would be used in the thesis and at conference attendances and publications (and also detailed within the participant information sheet – appendix 12). Following initial introductions at the beginning of the session, it was important to ensure that any possible power relations were identified and minimised as to ensure participants felt safe and supported in discussing sensitive content. Hall (2005) identifies this dynamic as

complex, especially when teaching healthcare professionals who may hold senior positions in their clinical roles over other participants. As the event involved students and more senior staff this did create a dilemma as to whether those in attendance would speak openly about their experiences adding a complex dynamic to the emancipation and engagement of those learning within a hierarchical healthcare setting (Olenick et al., 2019). To try and address this issue, at the beginning I made it clear about the sensitive nature of the content and that a supportive and non-judgemental approach to discussions would make for an authentic and collaborative approach to learning. I also made it clear how I would participate in discussions by sharing my own experiences of practice, further building trust among the group.

Following introductions, the midwives were asked to complete the consent forms and the pre-questionnaire. Once these had been completed, the midwives were asked to follow the instructions to individually view the filmed scenario through the VR headset.

8.7 Reflective thoughts '*Becoming the other'*

Once midwives had viewed the VR scenario, a reflective discussion was held. This discussion lasted for approximately 30 minutes in total. Discussions were facilitated by asking the group *'How did that make you feel?'.* This question aimed to facilitate a critically reflective discussion in which the participants were guided to explore their thoughts and feelings within the social space of birth, exploring interpersonal interactions between the midwife, doctor and woman. Participants shared their thoughts on the sensory experience of using VR, noting it made for a powerful and realistic experience of being the woman:

> *'Wow that was powerful, you really become the other, I felt like I was in the room and it was happening to me'* (Participant 3).

Experiences of '*becoming the other'* via a first- person perspective were explored both during discussions and in the post-questionnaire following the session. Participants expressed how a first-person perspective enhanced their situational awareness:

> *'It was interesting to be looking up all the time because I was on the bed, that must be really uncomfortable and intimidating, you just forget that dynamic of the situation in practice'* (Participant 6).

Participants also began sharing the most poignant moments for them during viewing. One particular scene that provoked reflection was when the midwife left the woman alone in the room:

> *'The worst bit for me was when everyone left the room, I was looking around to see if I had a birthing partner, it was powerful, just waiting around worried about what was going to happen next'* (Participant 4).

Another midwife focused on the lack of communication between her and the midwife/doctor during the scenario. The VR perspective prompted her to question why the doctor and midwife were not sharing information with her, expressing how this made her feel unnerved and anxious:

> *'The silences in the room were unnerving for me being the woman, I wanted her to ask me if I had understood what the doctor had said, I thought why is she [the midwife] not talking to me,'* (Participant 10).

This sentiment was echoed by another participant who was receptive to the midwives' body language during viewing:

> *'She didn't need to say anything, I could tell something was wrong, her body language and the way she wasn't making eye contact with me'* (Participate 9).

During these discussions, participants also made references to witnessed and personal experiences of supporting women during childbirth. A couple of the participants reported empathy towards the midwife, believing that the stressful nature of the situation was likely contributing to her task-orientated working practices:

> *'I feel sorry for her, you can clearly tell she is stressed and trying to get everything ready before theatre, you know what it's like, you're worried you will miss something or that something will go wrong'* (Participant 3).

Another midwife felt similar, noting that the current culture of working practices could have contributed to her actions:

> *'It's that worry, something's going to go wrong and it's going to be your fault, so you do panic, we are only human, I really don't think she's being that way on purpose'* (Participant 1).

These comments promoted discussions on how midwives may support each other in practice to prevent traumatic birth experiences. One midwife made a suggestion on how best to support colleagues who may find themselves in similar situations:

> '*She needed some help, even just for another midwife to go in and chat to the woman whilst the other midwife got her jobs done, I mean it doesn't even need to be a midwife does it, a healthcare assistant or a student midwife, just someone to help support the woman'* (Participant 5).

Becoming the woman encouraged a critical reflection of practices that could impact upon women's birth experiences. A mixture of operational and emotional aspects of care were discussed and simulated via the use of VR. This included participants stating more practical support in caring for women was needed and identifying how focusing on operational tasks can result in maternity professionals being less mindful of the impact poor interpersonal interactions have upon women.

8.8 Professional reflections and sharing experiences '*Nurturing professional empathy and understanding*'

As discussed above, participants were encouraged to share personal experiences of care delivery. The VR scenario aided this process by immersing them into a social context they were familiar with. No one was asked directly to contribute to the discussion, rather those in attendance were given the chance to voluntarily contribute. Following viewing, one of the midwives reflected on how paternalistic interactions are at times practiced in midwifery, although acknowledged how this may not be empowering for women:

> *'We* [midwives] *can be very paternal at times, but that's not empowering'* (Participant 5).

Critical pedagogies aim to illuminate dehumanisation practices, placing emphasis on the importance of being critical of how things are (Rosenberg, 2014). This process was organic during reflective discussions with participants commenting and identifying dehumanising aspects of care within the VR scenario. For example, a further midwife reported:

> *'It was like watching a united front from us [midwife and doctor], we have decided, two people together, two people against one, that this is what we are*

going to do to you and as the woman I was thinking hang on, what about what I think' (Participant 2).

With others acknowledging that those within the scenario were likely unaware of the impact of their actions upon 'Emma':

> *'You could see they were trying to do their best but failing miserably.'* (Participant 9).

A task-oriented approach to care was also highlighted during discussions, with one participant highlighting the need to prioritise aspects of care in practice:

> *'I don't think she was being purposefully uncaring she was just very task orientated as she had a lot to do and probably wanted to make sure everything was safe before they went to theatre, she did ask if she had anyone to go with her, so she was thinking about the woman needing support, but she couldn't be that person because she had a job to do'* (Participant 6).

This comment opened up a critical debate around the use of language in maternity care and exploring effective communication approaches and information giving when caring for women. For instance, one of the participants reported:

> *'Yes, but then she [Midwife] never followed it up when the women said no, she could have said don't worry I will be with you in theatre and I will support you.'* (Participant 7).

Reflections were also made on the impact prescriptive care can have on a woman's ability to advocate for herself, highlighting issues of how inappropriate languge and communication impacts upon choice and consent:

> *'It was all 'we are going to do this [midwife & doctor]', there was not much chance for the woman to disagree or ask questions and although they were doing what needed to be done, I think they could have put it to the woman a bit better'* (Participant 8).

Participants reflected on the difficulties of keeping women at the centre of care in their everyday practice. This encouraged participants to consider what impacts on the midwife's ability to provide good care. One participant recognised that whilst all

midwives could be accused of providing similar treatment, this in part could be attributed to a lack of support for midwives providing one to one care in labour:

> *'It's horrible to see but we have all been there, I feel that the midwife could have done with some support herself to better support the woman'* (Participant 1).

Another participant stated:

> *'It's easy to forget that there may be a midwife in another room who needs supporting and feeling like that when your busy with your own work, makes you think twice'* (Participant 6).

These comments highlight how the scenario triggered professional empathy, and a recognition of what support midwifery staff should receive to enable them to provide quality care to women.

8.9 Providing the theory '*What, why, when'*

As presented in Table 16, a theory-based presentation was delivered at this stage providing information on incidence and risk factors for birth trauma and PTSD, and insights into the secondary and empirical data collected as part of my study (i.e. findings from the meta-ethnography and interview data). Some of the participants expressed their upset when reading women's quotes:

> *'It really saddens me to read those, I'd hate to think that I've ever made a woman feel that way'* (Participant 7).

Participants lack of awareness as to what birth trauma and PTSD is and the prevalence of these conditions were also highlighted:

> *'I'd never have thought it would have been that prevalent, because you don't really hear about PTSD as a midwife, its more postnatal depression, I've never had any training on PTSD so I would not have known what to look out for'* (Participant 1).

With another participant highlighting the need for training due to the high number of women they care for clinically who present with symptoms:

Me neither, I've never had any sort of training on birth trauma or PTSD, I think it's so worthwhile, we see so many women with symptoms, training would really help us to help women' (Participant 3).

During discussions, participants identified a need for improved training for prevention rather than identification purposes, with one participant stating:

'I think it's would be much better that the focus is on preventing PTSD in the first place rather than receiving training after its happened, that's something we can actively do' (Participant 4).

These comments highlighted an identified need for training of this nature and how receptive participants were to receiving information on birth trauma and PTSD.

8.10 Addressing the context '*Critical thinking'*

Participants were then provided with additional information regarding the scenario as discussed in chapter five section 5.13.1. Participants were then asked:

'Thinking back to the scenario, how do you think these additional factors would impact upon Emma's birth experience?' (Facilitator).

Prior to adding this context, discussions focused on interpersonal interactions. However, following its presentation, participants began making links about providing care for women with complex needs, and placing the women within a psychosocial context of birth.

Initial responses focused on physical interactions such as the vaginal examination (VE) during the scenario. For instance, one midwife began the discussion by highlighting how the VE was a potential trigger for trauma:

'The VE, her legs being held open and her legs being man handled then putting them in the stirrups, with her history I imagine that would have been a trigger' (Participant 5).

Another midwife felt the VE could have impacted upon Emma's ability to retain control, discussing how her complex life situation could be exacerbated during situations that were disempowering or restrictive, creating a mistrust of those caring for her:

‘Lack of consent, she needs control more than anything, she needs to be in control so that she can trust the people looking after her, they didn’t give her any control over what was going on. Things were being done to her’ (Participant 4).

One participant used the VE performed within the scenario as an example of a potential trigger for trauma for women with a complex history:

‘She could have a history of sexual abuse and the VE could have traumatised her, it was a man too and the midwife never asked if the woman was ok with that, she [midwife] probably didn’t have any notes she could check either as she was out of area’ (Participant 3).

One midwife questioned the use of the overhead light during the VE as a potential trauma trigger and identified this as common practice:

‘The light, why does he [doctor] need to see doing a VE, its very objectifying, but they do use a light you see it all the time, crazy’ (Participant 1).

One participant also stated how she felt the midwife and doctor’s attitudes could have been a trigger for Emma, noting the dismissive body language and lack of reassuring facial expressions during the scenario could have made her feel as though the healthcare professionals did not want to engage with her:

‘*Lack of eye contact, no smiling or reassuring looks, I felt that, I could tell they didn’t want to engage with me and that was upsetting’* (Participant 7).

Another participant noted how the negative language made her feel, explaining how this could be a trigger for a woman with complex life factors:

‘‘I’ll be gentle, don’t worry it will only take a minute’, that language is very paternalistic and could massively impact upon a woman with a history of sexual abuse, or any kind of abuse’ (Participant 9).

During the discussion it was noted how a history of drug abuse would likely suggest complex life factors in which the woman may have experienced previous trauma. Reflecting upon the scenario, participants began making critical links to potential triggers for trauma:

'She has a history of drug abuse, so I imagine she had probably suffered trauma before putting her at greater risk of being re-traumatised, that's probably why she had a fear of needles' (Participant 8).

Another participant recalled witnessed practices during her previous employment, in which she exposed a culture of judgemental attitudes and stigma attached to disadvantaged and vulnerable woman accessing services:

'Where I trained, I would say the way staff treated disadvantaged women was absolutely appalling, but it was acceptable, for whatever reason, it became the norm' (Participant 5).

Another midwife agreed stating that cultures exist in labelling women who access care with complex life situations:

'You see it all the time, not here, but where I worked before, a woman comes in with a social Kardex [information provided in women's notes relating to social service involvement / complex life factors] and people automatically make judgements' (Participant 2).

To explore these issues further, I asked participants what kind of judgements would be made and how did they feel this could affect women's care during labour. Some recounted comments made in practice relating to women's choices to have a baby. For example, one reported:

'Women who come in who may have had other children removed, you hear others label those women as selfish and irresponsible for having a baby if they can't afford to look after it' (Participant 8).

With a consensus agreed in the room that a culture of stigma and judgements are witnessed across the board in healthcare towards those with complex life factors:

'That happens in healthcare full stop, even in nursing people will say that those accessing A+E with drug problems, alcohol issues etc, mental health problems, people, even staff, will stand there and say that they are draining the system' (Participant 3).

Addressing the wider psychosocial issues that impact upon women's experience of care encouraged a discussion on the importance of sharing potential risk factors for trauma that may have been identified or information that women may have disclosed to their healthcare professionals. However, this was challenged by another midwife who considered that many women may not disclose prior abuse due to a lack of relationship with their midwife:

> '*Yeah but not all women disclose abuse do they, we shouldn't be focusing on what if she has had previous trauma because not many women are going to disclose sensitive information like that in the 15 minutes they get to see a midwife in the community, or here [hospital]'* (Participant 7).

It was clear from the discussions that adding critical context to the scenario appeared to enhance participants ability to make connections between interpersonal interactions and the possibility of contributing to a traumatic experience.

8.11 Critiquing the system *'Fit for purpose?'*

During discussions, participants expressed their frustrations of working in a system that did not facilitate individualised care. They expressed how the midwifery care system left them feeling overworked and under resourced when it came to caring for women with complex needs:

> *'It's a one size fits all approach, when women come into the unit with additional needs or demanding certain things it can sometimes be quite stressful as there is not always the resources or the specialist care needs to support them'* (Participant 3).

One midwife expressed her frustrations at feeling under scrutiny in situations such as the one presented in the scenario:

> *'I mean she's trying her best, she's in task mode, she needs to get everything sorted before she goes to theatre or they will probably shout at her for not having the right paperwork, you know what it's like'* (Participant 8).

Another participant offered similar sentiments, stating that she often felt frustrated when caring for women who did not speak English in labour:

'It's so hard when you have a woman that doesn't speak English, you can't use language line on women in labour, it's not practical at all, you just got to do what you can'' (Participant 4).

One participant stated how they did not have the resources to care for women at risk of trauma or those who may have complex needs:

If women don't come in with a partner, we can sometimes feel helpless because we don't have the time or resources to fulfil the role of partner and midwife' (Participant 2).

Freire's (1972) theory of conscientization provides a valued reference point for considering the views people express and challenge through a process of problematisation and conscientization. This was apparent during discussions in which participants empathised with each other, identifying the pressures and difficulties faced by all when working in an oppressive environment. Foster & Mckenzie (2018) recognise this approach in education as enhancing learners' emotional intelligence by fostering empathetic connections with their peers. This is challenged by Methot et al, (2016) who identified that although these relationships enhanced job performance through trust, these can also impact upon the psychological wellbeing of staff due to emotional exhaustion when working in environments that required a high level of human interaction. These points highlight the need for critical pedagogies, such as the one used within this study, that promote action-reflection as a tool for dismantling such oppressive environments that impact upon the quality of care women receive during birth (Patterson et al., 2019).

Shared learning during delivery raised interesting questions regarding perceived levels of self-efficacy and autonomy held by participants. For example, some agreed that the demands upon time and the failures of the wider hospital system impacted upon care, but felt this was not something they were able to personally impact upon. However, as discussions evolved, participants focused on the individual impact they could have on women:

'I do think the midwife could have reassured her more, maybe explain things to her a little better about where she [the midwife] was going, how long she would

be, or give her the buzzer so if she was worried she could buzz her [midwife] to come back then she didn't feel abandoned' (Participant 10).

The group dynamic focused on issues that participants felt they were able to change, whilst acknowledging that the system caused situation of oppression, both for themselves as practitioners and women as patients:

'*Something needs to change, the system is set up to fail, women and midwives need to speak up, let the government know that more needs to be done to prevent birth trauma, these women need the support they deserve'* (Participant 3).

Adding context and facilitating dialogue and reflection, promoted thought provoking discussions amongst the group and set the scene for the next stage of the programme, *'Practice Points'.*

8.12 Creating critical knowledge *'Practice Points'*

It was important within the delivery of the programme to facilitate a way of sharing learning into clinical areas. '*Practice Points'* as discussed in chapter five section 5.14.1, were to provide those in attendance with learning points that could be displayed and shared in clinical areas, aimed at stimulating discussions around reducing birth trauma and promoting respectful care. *'Practice Points'* were devised during a group session. Participants were asked to think of key points for practice, which were then documented on a template (appendix 17), and taken away by those in attendance to display in their clinical areas.

It was important at this stage to facilitate the discussion, as opposed to being actively involved. This was to ensure that the points produced were organic, context specific and developed by participants. Critical knowledge devised through the development of *'Practice Points'* aimed to facilitate praxis when delivering care to women. For example, instead of midwives receiving, filing, and storing deposits of learning material delivered, praxis was encouraged through the facilitation of reflection, theory and action embedded with a critical pedagogy (Freire, 1970). The five practice points that were devised and agreed amongst all participants were:

1. **Inclusive handover with woman.**
2. **Be considerate of disclosed and undisclosed history of trauma.**

3. **One to one care in labour**.
4. **Use positive language**.
5. **Put yourself in the woman's shoes.**

Interestingly, participants challenged each other in the language used when devising practice points. It was considered important to use language that would not offend, induce blame or cause distress to colleagues ensuring the language used could not be interpreted in any other than which it was intended. The first practice point ***'Inclusive handover'*** related to how participants felt it was important to ensure women were included in discussions and decisions. Participants identified that although handovers took place in the labour room, this could be a disempowering process if the woman is not actively involved in the discussions.

The second practice point '***Be considerate of disclosed and undisclosed history of trauma'*** formed part of an interesting discussion regarding previous trauma and risk factors for PTSD. Initially the group felt that is was important to identify previous trauma as a risk factor for birth trauma. However, as it was felt that many women may not disclose their personal history it was deemed important to treat all women as potentially having risk factors for birth trauma and PTSD.

For practice point three ***'One to one care in labour'*** the group identified the positive impact of continuity of carer. One participant stated how multiple caregivers can increase anxiety and stress for women, especially those who found it hard to form trusting relationships:

> *'I see it all the time, women get anxious and really stressed when people are in and out of the room, especially if they are in the zone. It's even worse for women who are alone and those with complex histories, a lot come in with their guard up'* (Participant 5).

This created a dialogue in how one to one care may be an important factor to reduce the risk of birth trauma:

> *'Like we said before, if there was just someone there reassuring the woman and providing that emotional support that could have prevented her from experiencing her birth as traumatic'* (Participant 8).

For practice point four ***'Use positive language'*** participants identified the need to change the terms that are regularly used that may impact upon women's sense of achievement and self-efficacy during birth. This opened up a discussion about terminology used in practice and how it can be disempowering for women:

> '*Failure to progress, baby is tired, ineffective pushing, only 5cm or still 5cm, there are some pretty negative statements made to women in labour, looking objectively those comments would come across as negative'* (Participant 3).

Discussions on positive language centred on the dialogue between women and her care providers, discussing the impact of language on experiences:

> *'Positive language is so important, we can all do better in that respect, you go into autopilot sometimes, maybe just to say be mindful of your language and how you say things'* (Participant 6).

Lastly, participants felt it was important to encourage ongoing reflexivity by encouraging health care practitioners to ***'Put yourself in the woman's shoes'.*** Participants felt that this area of practice is often neglected due to factors such as time constraints and the lack of a structured debriefing system for staff:

> *'It's important to take the time out to reconnect with the experiences of women, to put yourself in their shoes and think about how things may feel on the other side of your care'* (Participant 7).
>
> *'Definitely, I also think we could do things better in practice, how we speak to women, what we say and how that could make them feel'* (Participant 8).

The discussion highlighted how encouraging reflective practice could serve as a means to reconnect midwives with the essence of being in a caring role:

> *'Sometimes it takes something like this [the programme] to make you take a step back and think about your own practice and how you may have become desensitised, it's a good way of remembering the reason you became a midwife in the first place, it is for me anyway'* (Participant 4).

8.13 Pre/post questionnaire findings

This section presents findings from the pre/post questionnaires (see appendix 15 & 16).

From responses to the question on the pre-evaluation questionnaire, all participants reported that they had received no training in relation to birth trauma / PTSD throughout the course of their midwifery career.

Responses on questions to assess the participants learning and knowledge at both time points are presented in table 16.

Table 16 Pre and post questionnaire result

Question	Strongly agree		Agree		Neither agree not disagree		Disagree		Strongly disagree	
	Pre	Post	Pre	Post	Pre	Post	Pre	Post	Pre	Post
Do you feel you have a good understanding of how birth trauma /PTSD onset following childbirth is caused?		50%	30%	50%	30%		40%			
Do you feel able to recognise women at risk of birth trauma / PTSD onset?		50%	30%	40%	50%	10%	20%			
Do you feel able to positively impact upon a woman's birth experience?	50%	60%	50%	40%						

Pre / post questionnaire findings suggest that attendance enhanced participants knowledge and understanding of birth trauma and PTSD onset following childbirth and risk factors that may place women at an increased risk. Findings also suggest participants felt more able to recognise women at risk of birth trauma and PTSD following attendance. Only a small increase was noted in regarding to participants feeling able to positively impact upon a woman's birth experience after attendance, although pre-responses suggest participants already held positive beliefs about their ability to positively impact upon women's experiences. When placed in context with qualitative responses collated during discussions, frustrations with oppressive environments, a lack of support in caring form women with complex needs and an acceptance of varying levels of care and judgemental and stigmatising practices towards women who needed additional care were noted as barriers to being able to positively impact upon a woman birth experience.

8.14 Exploring the experience of VR

Participants were also asked to provide qualitative feedback on the use of VR to enhance the learning experience during the post evaluation questionnaire. The feedback was extremely positive with midwives expressing how using VR enhanced their understanding of birth trauma from a first-person perspective. Midwives felt that learning this way enabled them to identify points in practice that they may not have otherwise noticed. One participant stated:

> *'It wouldn't have been the same if you had just filmed it, but this was great because I was in the room'* (Participant 6).

Attendees were also asked what they felt was good about the training. References were made to the use of VR and how it enhanced their learning experience, one midwife said:

> *'It's a different kind of learning isn't it; I could feel the vulnerability just with simple things like having my legs in the stirrups and being alone in the room when the midwife rushed out'* (Participant 3).

They expressed how they felt the VR had been useful in enhancing reflection:

> *'I thought it was great because it's good for looking at your own behaviours and reflecting upon them, usually in training everyone feels they have to put on their best performance because everyone is watching your every move, with this [the VR training] it's more of a private thing because you're watching thinking 'oh I do that', 'maybe I should change the way I think about that' (*Participant 3).

Another participant stated how viewing through the VR had made her more sensitive to the environment and how vulnerable this can feel on the receiving end of care:

> *'It's strange as I've never been in that position myself, but I felt vulnerable and I didn't like how it felt looking at my legs in stirrups, I felt exposed'* (Participant 5).

Feedback suggested the use of VR enhanced perceptions and enabled participants to identify key issues within the scenario that could cause situations of trauma. Viewing the scenario via VR engaged participant's senses and emotions unlike traditional teaching methods:

'It's one thing being told how to be and what not to do by someone stood at the front of a classroom, it's another thing having those things happen to you [via VR] and you feeling helpless to do anything about it' (Participant 10).

8.15 Follow up questions

At the end of the session a time and date were agreed with all participants for the focus group. Despite a reminder email sent to all participants the day before the focus group session, no participants attended. The transformation of social action through praxis was difficult to measure through classical Freirean approaches (focus group / dialogue) due to the impracticalities associated with facilitating a group session, within the hospital, in the staff's own time. After contemplating how best to address this issue as discussed in chapter five section 5.15.4, participants were contacted individually by providing the follow-up questions via email. The questions included:

- Do you feel attending the educational programme has influenced how you interact with women? If so how, if not, why not?
- What was your experience of sharing the practice point with colleagues?
- How were the practice point received? Did they help influence practice?
- How do you feel the educational programme could be developed i.e.; what more is needed? How else could it be delivered? What other 'films' could be used?

Five participants responded in total. Overall, the respondents provided positive feedback in that they felt the programme had made them more mindful of possible risk factors for trauma providing them with points for reflection when delivering intrapartum care:

'Now I'm more aware of how vulnerable a woman feels when I'm speaking over her or how she may feel lost when I leave the room. I'm more conscious now of being precise about why I am leaving the room and when I will return' (Respondent 4).

All respondents stated that sharing practice points was difficult and were unable to comment as to whether sharing the practice points in their clinical areas had been received positively or negatively. However, some did refer to how others in the team were curious about the VR training programme.

All respondents stated that attending the training has enhanced their understanding of birth trauma and PTSD:

> *'I feel I am more knowledgeable about risk factors for birth trauma and PTSD following the training'* (Respondent 2),

although others highlighted the difficulty in explaining to others the impacts this had upon them:

> *'It was hard to explain the programme to someone else other than it was really useful and powerful. I also don't think you can get across to people the emotion attached to the session or viewing the scenario, they need to attend themselves, I think that would be better'* (Respondent 5).

Suggestions for development of the programme was to use the VR concept to train for emergency situations such as neonatal resuscitation and obstetric emergencies. One midwife also suggested filming a positive birth experience could help demonstrate how care should be delivered. One midwife also highlighted the barrier to engagement with training that is not embedded within their mandatory requirements:

> '*it's so difficult working full time and keeping up to date with CPD [continual professional development] stuff, I think it would be great to having something like this in mandatory training, it's definitely needed'* (Respondent 5).

Overall feedback was positive and provided future points for knowledge translation into practice.

8.16 Conclusion

This chapter provides insights into details, practicalities and evaluation of the educational programme. Findings suggest that the use of VR to deliver evidence-based content may enhance maternity professionals' perceptions, reflections and sense of engagement with women experiencing oppressive forms of maternity care. The use of a critical pedagogy reflected in how it enhanced critical reflections to address oppression and dehumanising situations that may cause birth trauma. A critical approach to delivering maternity education in practice offers a space in which situations of power and institutional norms and practices may lead to oppressive and impersonal care experiences, both for the woman, and health care practitioners. Becoming the other

allowed participants to experience a fully immersive birthing scenario to appreciate the spatial, interpersonal and emotional experience that some women may face.

Findings from the pre/post questionnaires suggest that the immersive educational programme enhanced participants understandings of birth trauma and PTSD, creating a space in which they could share experiences. Findings also highlight gaps in current training and education for midwives on birth trauma and PTSD. Chapter nine positions the finding of my thesis and the immersive critical pedagogy within the wider literature, discussing its original contribution to knowledge and recommendations for policy, practice and research in terms of comprehensibility, feasibility and acceptability, Chapter nine also discussed the strengths and weaknesses of the study with a conclusion of the thesis presented.

CHAPTER 9: DISCUSSION

9.1 Introduction

In the previous chapters I discuss the design and delivery of the immersive educational programme, together with findings from the evaluation study. Within this chapter I summarize my study. The unique contribution to knowledge within this study is then discussed, followed by central interpretive findings. Through a critical lens I offer insights into the lived experiences of disadvantaged and vulnerable women during birth within my discussion, drawing on literature concerned with power and othering. I then discuss findings from the evaluation phase of the study, offering insight into critical issues illuminated during delivery, alongside the acceptability of using an immersive critical pedagogy in maternity education for health care professionals. I also offer reflections on the experimental use of VR within a critical pedagogy, aimed at facilitating conscientization. This chapter concludes by detailing the study's potential global impact, implications for practice policy and research, strengths and limitations and recommendations for further development in practice.

9.2 Summary of study

The main aim of this study was to develop and deliver a feasible and acceptable educational programme for maternity professionals to reduce the number of disadvantaged and vulnerable women experiencing a traumatic birth and the development of post-traumatic stress disorder (PTSD). A meta-ethnography allowed for the identification of key themes relating to disadvantaged and vulnerable women's experiences of maternity care in high income countries. Findings from the meta-ethnography suggest that disadvantaged and vulnerable women experienced poor care and varying levels of inequity and inequalities due to their complex life factors, exacerbated during interpersonal interactions with health care professionals. Judgemental attitudes were seen to cause obvious barriers to engagement and the ability to build trustful relationships with healthcare providers. These experiences also had an impact upon women's levels of satisfaction with maternity services.

Empirical interviews within a locality provided rich narratives from disadvantaged and vulnerable women who had suffered a traumatic birth, allowing for the identification of key triggers for trauma, in direct relation to interpersonal care interactions. Using findings, an immersive 90-minute educational programme was developed using an

identified framework for designing critical pedagogies, in line with the theoretical lens applied within this study. Adopting an innovative approach to translating findings, a 360 VR video was filmed using women’s narratives to develop an evidence-based script, highlighting key triggers for a traumatic birth experience. An evaluation of the programme was undertaken in an NHS hospital with key findings suggesting the programme enhanced participants knowledge of birth trauma and PTSD. VR as a tool for reflection was also highlighted as positive aspect of the programme, offering a sensory experience and unique approach to addressing and reflecting upon care practices.

9.3 Unique contribution to knowledge

My research offers an original contribution to the important field of research concerned with birth trauma and within the field of maternity education, both in terms of specific findings and the novel approach to knowledge translation applied in this context. Firstly, a meta-ethnography had not been undertaken exploring the experiences of maternity care for disadvantaged and vulnerable women in high income countries prior to this study. Themes generated from this review provide a comprehensive basis from which to compare disadvantaged and vulnerable women’s traumatic experiences of maternity care within the global literature. The accounts of traumatic birth experiences amongst disadvantaged and vulnerable women in high income countries indicate that these women experience varying levels of disrespectful, judgemental, dehumanising and inverse care practices, with experiences resonating with those of women in developing nations.

Secondly, evaluating the acceptability and feasibility of immersive critical pedagogies has provided an original contribution to knowledge, not only regarding the use of critical pedagogies in midwifery education, but also the use of VR as a tool for presenting evidence-based narratives to facilitate reflection amongst healthcare professionals. The educational programme was received well and is reflected in findings gathered from pre /post questionnaires and a six week follow up. Findings suggest the immersive educational programme enhanced participants knowledge and confidence in identifying and supporting women at risk of developing birth trauma and contributory factors associated with its development. Key barriers to sharing knowledge in practice were explored during the six weeks follow up, collecting data via email. Responses from participants suggested that time constraints and a lack of opportunity to sit and discuss

the programme and the practice points with colleagues served as barriers to sharing learning in clinical areas. Findings suggest further work is needed to explore effective ways of sharing knowledge in practice, as is exploring new ways on how to best to facilitate this practice.

Lastly, findings highlighted the benefits of using VR as a reflective tool and how this enhanced participants emotional responses to the situation from a first-person perspective. Furthermore, the use of VR from a first-person perspective to explore interpersonal care interactions in healthcare settings has not been implemented elsewhere, opening up unprecedented opportunities for its use in many disciplines to raise conscientization. To date this is the first study of its kind that uses immersive critical pedagogies to address interpersonal care, and the first midwifery programme that focuses on birth trauma and PTSD using this approach.

9.4 Central interpretive findings

Five central and interrelated findings from my study are summarised below:

1. Disadvantaged and vulnerable women in high income countries experience similar issues to women in developing nations that contribute to negative and traumatic subjective perceptions of childbirth. These include judgemental attitudes, inverse care practices, lack of emotional support, loss of control, unconsented interventions and disrespectful care.
2. Women were not counted as *'knowers'* during a traumatic birth, reinforcing negative self-perceptions of self-amongst women with complex life factors.
3. Women's socio-economic positions and complex life factors served as a point of stigmatization and judgement when interacting with maternity professionals during birth.
4. Simulating a first-person perspective via the use of immersive technology was seen to facilitate a critical approach to exploring interpersonal factors that contribute to women experiencing a traumatic birth and PTSD.
5. The use of a critical pedagogy in maternity education was seen to facilitate conscientization amongst participants.

These central interpretive findings are situated in the wider literature below.

9.5 '*Them & us*' a neoliberal crisis in maternity care?

Key findings from empirical stages within this study suggest that poor interpersonal interactions directly impacted upon women's sense of control, agency, sense of self and ability to form meaningful relationships with their healthcare professionals during birth, contributing to the potential for birth trauma (Ayers et al, 2016, McLeish & Redshaw, 2018, Patterson et al., 2019). Findings highlighted that women felt their care differed from those around them, raising questions about inequalities in care provisions for women from disadvantaged and vulnerable backgrounds. Sturgeon (2014) notes how such practices in healthcare are seen as a degraded morality of practice, in which the consumer approach to care is seen to outcast those who are seen as unworthy of public resources, based on their economic worth in a society. Others offer similar commentaries, believing provision of healthcare are directly related to assumptions about the economic contribution and worth of human endeavour (Janes et al., 2006, Benoit et al., 2010, Giroux 2014, Benoit et al., 2015, Macintyre et al., 2018), including within maternity care (Sandal et al., 2009).

Within this body of work, I propose that the aforementioned could be further understood by applying the theoretical stance of lifeworld and system world, a critical theory proposed by Jürgen Habermas. In his seminal work '*The Theory of Communicative Action*', Habermas describes the lifeworld as based on mutual communication; face to face interactions and the development of shared norms and values (Habermas, 1984). In contrast, the systems world is based on inputs and outputs, supply and demand, revolving around changes concerned with money and power (Habermas, 1984). Habermas highlights a worrying tendency in society in which the system world is moving into the lifeworld, colonising and eroding mutual understandings, creating a capitalist rationality (Habermas, 1984). Health services are seen to sit at the intersection between system and lifeworld resulting in potential contradictions when driving the dogma of an equitable service in a system working under a capitalist ideology.

To offer a potential explanation to how these issues manifest, Roberts and Ion (2014) draw on the writings of Arendt, to discuss how moral catastrophes of care result from healthcare professional's inability to question systems, actively partaking in and accepting challenging work practices. Thesen (2005) disagrees, noting an individual's ability to rationalise and question their reality should not be accepted or seen as an

inability but explored, asking why individuals choose not to engage or question situations of oppression. Drawing on findings from the delivery of the immersive educational programme, at times, participants seemed complicit in a consumer approach to care delivery, in which one participant noted how women would *'demand'* (chapter 8 section 8.11) levels of care. This was further noted when participants offered reflections on practices, including commentaries on the attitudes of some professionals towards women accessing care with complex life situations. These reflections included direct references to a consumer rationality, including women who could not *'afford'* to look after children and those accessing care, or in direct relation to those who were seen to be *'draining'* the system (chapter 8 section 8.10).

Critical commentaries on healthcare systems have noted similar tensions and strains on the midwifery workforce, in a context of increasing uncertainties about resources and the knock-on effects of neoliberalism for professionalism and professional practice (Sandall et al, 2009, Benoit et al., 2010, Nkansah-Amankra et al., 2018). These commentaries were also noted by participants during delivery when discussing perceived barriers to delivering quality care (Chapter 8 section 8.11). Bauman (2000) coined the term '*liquid modernity'* to illuminate how a context of fluidity and uncertainty amongst public service workers due to the precariousness of their working practices, facilitating a crisis of '*moral blindness*[25]*'* within current healthcare systems. Unsurprisingly, this has been associated with a detriment in quality and safety of care and patient experience (Randall & McKeown 2014). In the extreme, it could be argued that the maternity workforce habituate to this erosion of standards in practice, becoming increasingly complicit in a dilution of concern for others and colleagues (Roberts & Ion, 2014).

Following on from this proposition, Bal & Dóci (2018) note how neoliberal economic policies emphasize the importance of individualism against claims of collective responsibility, resulting in the co-modification of social relationships as market values (Harvey, 2005, Van Apeldoorn & Overbeek, 2012, Morgan, 2015, Curran & Hill, 2017).

[25] Moral blindness is noted as the state of being unaware or insensible to moral issues pertaining both to oneself and to one's relations to others.

My findings seem to suggest that neoliberal ideologies may not only be infiltrating working conditions in maternity care, but also the beliefs and attitudes of staff, leaving maternity professionals both complicit in their own oppressive actions and potentially unaware of the ideologies that lay these foundations. Such limitations of consciousness serve as a potential barrier to progressive actions on improving maternity care experiences for women, making the case for the conscientization work within this study.

9.6 Deconstructing the *'other'* in maternity care

Drawing upon disadvantaged and vulnerable women's traumatic birth narratives within this study has allowed me to explore the political and social influences that may impact upon their experiences. This led me onto exploring theories that may offer an explanation on the phenomena of birth trauma for these women. Through engaging with the critical theories of Imogen Tyler, Miranda Fricker and Paulo Freire, I have explored the impact of othering, epistemic injustices and oppressive environments upon the interpersonal interactions between healthcare professionals and disadvantaged and vulnerable women. *'Dehumanisation'*, a theme generated in the meta-ethnographic review highlights the impact of poor interpersonal interactions and *'othering'* in maternity services, also reflected in the global literature concerned with disrespectful and abusive care practices in maternity care (Browser & Hill 2010, Bohren et al., 2016).

Giroux draws on the idea of othering believing that a social responsibility toward those who are vulnerable, or in need of care, is now a thing of the past, propositioning that societies' most vulnerable people are now viewed as a scourge or pathology in healthcare (Giroux, 2014), resulting in a discourse of the *'other'* dominating healthcare spaces. Similar views were expressed by women within this study, both reported in the meta-ethnography and during interviews, in relation to judgemental attitudes that made them feel the care they received differed to those around them (chapter 3 section 3.17, chapter 6 section 6.4.6). Situated within the wider critical literature concerned with othering, it is important to note that other intersectional aspects of identity are also open to judgemental constructions and representations, be it through discursive processes of attributing difference, or when individuals are *'othered'* in relation to race, nationality, class or disability (Johnson et al., 2004, Alpers et al., 2018). As such, the healthcare system is replete with opportunity to construct people as the *'other'*, readily

collapsing social interactions into a *'them and us'* dynamic during work encounters (Mercer & Flynn, 2017).

From this perspective, the dynamic of othering can be readily associated with the demonization of the other, locating them as a repository for blame and the projection of acknowledged, and unacknowledged, faults and flaws within the self and society (Fanon, 1986, Miles, 1989, Timmi, 1996, Jacob et al., 2016); creating a discontent towards those accessing healthcare with complex needs. Findings within this thesis provide examples of the *'appalling'* treatment of disadvantaged and vulnerable women in environments where participants had worked in previous employment (chapter 8: section 8.10). These reflections included stigmatizing practices and attitudes towards those accessing care with complex needs. Harkins et al (2016) offers an explanation for such practices, suggesting that the media plays an important role in casting the disadvantaged and vulnerable as undeserving of public and state support, thereby feeding the wheel of inequality within a society. This rhetoric is exacerbated by the continuous reporting of the difficulties faced by the NHS, now seen as a disjointed and underperforming business that is unable to deliver quality care; highlighted by participants during discussions (chapter 8, section 8.11). Findings within this thesis suggest that a critical pedagogical approach could help to illuminate potential social and political contradictions within maternity care that may impact upon the attitudes and beliefs of those providing care. Exploring to what extent this rhetoric has upon the beliefs and attitudes of HCP's towards those accessing care with complex needs, raises the potential for a more complete conscientization of staff through an exploration of the *'other'* within a maternity context.

Offering a lens through which to explore examples of othering within a maternity context, Tyler coined the phrase '*Chav Mum, Chav Scum*', in which she discusses class disgust and the vilification of young white working-class mothers who choose to have a baby (Tyler, 2008). Tyler believes that media representations of the other (for example; disadvantaged and vulnerable women) are mobilised in ways to assign superior forms of social capital to the social group they are implicitly or explicitly differentiated from (for example; maternity professional). Within this thesis women reported feeling they were treated differently, either due to their class, ethnicity or age, raising questions about the constructs of the '*other*' and the identification of a possible class disgust,

displayed in a maternity context (chapter 6 section 6.4.6). Participants demonstrated this position during delivery of the programme, in which examples of witnessed practices towards those accessing care with complex life factors were given (chapter 8 p. 162). I argue within this thesis that the practice of social abjection during birth is exacerbated by the pressures placed upon the underfunded NHS system, where abjection collides with extremes of resource depletion and professional uncertainties, contributing to the potential for disjointed and broken relationships during birth.

9.7 Epistemic injustices in maternity care practices

Epistemic injustices were also highlighted within this thesis. Women expressed concerns that their healthcare professionals did not listen or engage with preference for their care during birth, contributing to situations of trauma. These findings resonate with the wider literature on poor care experiences in the general population of women who report issues of not feeling listened to during birth and having no ownership over their birth experience (Bohren et al., 2015, Thomson & Schmied, 2017, Greenfield, Jomeen & Glover, 2019). Drawing on '*Epistemic injustice*' (chapter 3 section 4.2.7), examining power relations and virtues of justice within a maternity context serves as a way of reflecting upon potential contributory factors to the oppression of women during birth. Additionally, not feeling informed of decisions or feeling pressured to conform undermined women's abilities to make responsible choices and their ability to form trusting relationships with their care providers, further contributing to situation of trauma (chapter 6 section 6.4.4).

Fricker (2009) describes this as *'Testimonial injustice'* - not being counted as a *'knower'*, in that being counted as a source of knowledge matters just as much as whether your beliefs are true and justified (Fricker, 2009). In the context of birth, this phenomenon could be seen as a source of objectification, in that when women were not treated as a knower, informant, speaker, they were not treated as fully human. Epistemic and testimonial injustices during birth could exacerbate vulnerable women's negative beliefs about themselves when they are dismissed, stigmatised, and judged contributing to a lack of agency. Reflecting upon practices that threatened women's autonomy and agency during birth during discussions facilitated critical thinking about routine practices that could be experienced as traumatic for women (chapter 8 section 8.10), in particular when reflecting upon becoming the woman during the simulation, in which participants

became passive recipients of care (chapter 8 section 8.7). Raising the conscientization of participants during the delivery of the programme was seen as a step forward in naming epistemic and testimonial injustices played out in the social space of birth that impact upon experiences of care, enhanced by a first-person perspective.

These issues highlight that whilst it is useful to focus on interpersonal interactions during birth as a preventative approach to birth trauma, a whole systems approach to addressing epistemic injustices and social abjection, facilitated by those working within maternity care, is also needed.

9.8 Collective efficacy – a critical approach to change

In 2016, the Royal College of Midwives (RCM) published a report, *'Why midwives leave – revisited'*, with three recurrent issues highlighted: staffing levels at work; the quality of care they were able to give; and their unmanageable workloads (RCM, 2016). Patterson et al (2019) note that the failure to recognise and meet the human and relationship needs of both women and midwives by maternity care organisations, contributes to poor care provider interactions and negative experiences of care by women. These issues were raised during delivery of the programme in which participants discussed their frustrations at the NHS system, stating they felt it was not conducive with quality care (chapter 8 section 8.11). Chomsky offers a lens through which to explore how healthcare workers may reach this point, believing that the system world manufactures collective consent parameters, so that any radical ideas or possible solutions to improve working conditions are silenced (Chomsky, 2017); creating defeatist attitudes towards the potential for progressive change. Habermas believes that this lends a dependability to the identity of such beliefs, by facilitating situations of acceptance and submission (Habermas, 1987a), creating feelings of apathy and frustrations amongst staff who feel unable to impact upon their situation.

These attitudes were noted during delivery of the programme, in which participants expressed their frustrations and empathy towards the midwife in the scenario. Participants stating that although they could identify that her care was poor, they understood how she may be finding it difficult to deliver quality care due to her working conditions (chapter 8 section 8.11). A critical pedagogical approach was seen to facilitate thinking about the system level issues (working conditions) under which they practice, alongside lifeworld issues (interpersonal interactions) that may impact upon birth

experiences for women; making key links and distinctions between the two (chapter 8 section 8.11).

For those that attended the programme, the creation of *'practice points'* aimed to provide participant's with self-initiated small-scale change actions that would give meaning and direction to their participation in a social movement (Ledwith 2015, Halman et al 2017). In the case of this study, the emancipation of both the women in their care and the working conditions under which they practice. This approach is encouraged by Moskovitz & Garcia-Lorenzo (2016) who propose that a meaning which is created through personal agency and collective efficacy can contribute to a sense of vocational and organisational identity. Such approaches to maternity education provide an example of how critical pedagogies may help foster a collective sense of social responsibility amongst staff, who may feel like their working practices impact upon their ability to deliver respectful and personalised care to women.

9.9 Tensions between policy and practice

Visions set out in the Better Births recommendations (NHS England, 2016) are supported by Denny (2018) who believes the ability to make informed choices, receive safe, personal and empowering care begins and ends with respectful relationships between maternity professionals and the women accessing care. Positive accounts of women's care during empirical stages support this, with women listing trust, kindness and respectful interactions as key attributes associated with good interpersonal care. Unfortunately, recommendations for inclusive, respectful and individualized care for disadvantaged and vulnerable groups are failing women, reflected in the level of inequalities faced by these women on a global scale (Koblinsky et al., 2016). To begin tackling the issue of birth trauma and poor experiences of care, this thesis suggest that healthcare systems must recognise trustful and respectful relationships as a key factor in improving these women's birth experiences, breaking down potential power structures that infiltrate the social space of birth. Historically, operations of power in the field of maternity care reflect a power struggle within healthcare between the expert (doctors, midwives, nurses) and the patient (women) (Peterson & Bunton, 1997, Gupta & Richters, 2008, Solnes Miltenburg et al., 2018). Mkandawire-Valhmu (2018) believes a critical lens must first be applied to deconstruct poor experiences of healthcare that illuminate such power structures. To begin realising this vision in maternity care, a

critical deconstruction of external influences and political discourses that drive abjection and unjust power relations within the social space of birth is encouraged (Robinson, 2001, Smith et al., 2009). Critiquing the social space of birth through listening and naming, dialogue and reflection and the promotion of transformative social action offers an approach where this could be facilitated, demonstrated within this body of work.

9.10 The value of narratives in conscientization

When narratives are presented in meaningful ways, everyday examples of practice can be used as an opportunity to learn. Drawing on Kohlberg's learning taxonomy (Kholberg 1958), McKeown, Malihi-Shoja and Downe (2011) state how using real life narratives in education promotes reflections and provokes emotions linked to the lived reality of the narrative, encouraging a deeper understanding of a given phenomenon. The aim of using narrative in education, demonstrated in this thesis, centres around the idea of creating empathetic responses that mimic real life situations to aid learning and reflection (Alismail, 2015, Scamell & Handley, 2017, Siu, 2018). Hoffman defines empathy as:

> *'A psychological process that make an individual have feelings that are more congruent with another's situation than with his or her own'* (Hoffman, 2000, p. 30).

According to Hoffman a key feature of empathy is '*empathic distress'*, which is the psychological discomfort felt when one encounters someone who is suffering (Hoffman, 2000). Facilitating such an approach within this work was seen to be a powerful tool in connecting participants to the experiences of women through the use of VR to deliver an evidence-based narrative (Chapter 8 section 8.7). Within this study using empirical accounts of women, embedded within a VR scenario, not only facilitated an empathetic response, but also encouraged a personal emotional response as participants felt as though they were experiencing the situation first-hand. Narratives are also an essential aspect of maternity care redesign, with the Maternity Service Review encouraging services to use women's feedback to help identify areas for improvement and highlight good practices that enrich the experiences of women's care (NHS England, 2016). Conversely, midwifery education focused on reducing birth trauma and PTSD must also acknowledge the historical and complex interconnected factors that place women at risk of birth trauma, through an acknowledgment of contributory factors associated with

its development (Simpson & Caitling, 2016, Thomson Beck & Ayers 2017), facilitated by the three step model adopted within this study.

Providing information of the aetiology of trauma and PTSD as part of the immersive educational programme was seen to empowering participants in understanding the physiology and manifestation of trauma, as well as the factors that contribute to its development (chapter 8 section 8.10). Providing participants with this information enhanced their ability to identify women who may be at risk and an awareness of interactions and conditions that can cause situations of trauma during birth (chapter 8 section 8.13). Despite results suggesting attendance provided participants with knowledge, self-reflection and changes to their working practices in subsequent care delivery (chapter 8 section 8.15), responses from the six weeks follow suggest participants found it difficult to share learning with others in practice. Respondents reported that attendance to the programme is required to fully appreciate the sensory experience of VR and to engage and partake in reflective stages to truly impact upon the practices of others (chapter 8 section 8.15). Despite issues faced in sharing learning, this study highlights the potential of using narratives to impact upon individual practices, potentially reducing the number of disadvantaged and vulnerable women experiencing a traumatic birth.

9.11 Complexities of technological contributions to critical pedagogy

Effective and responsible pedagogy in the 21st century requires the incorporation of technology into teaching and learning strategies (Fullerton & Ingle, 2003). This said, critical theorists identify the difficulties faced in liberating practices in an ever-evolving techno scientific society (Feenberg 1991, Brock, 2018). Hardy and Negri (2000) challenge this view believing that user intentionality is key in utilizing technology as a tool to turn against capital or oppressive states, if wielded strategically. Contradictory to Horkheimer and Adorno's belief that technological advances favour capitalism and consumerism (Horkheimer, 1982, Adorno, 2005), global economic advances urge us to revisit this belief, with 62.9% of the global population owning a mobile phone that allows them to browse the internet (Statista, 2019). These statistics open up unprecedented opportunities for critical educators to use technology and global communication platforms as a tool for change.

In a healthcare context, the adoption of technology can face resistance from professionals. Identified issues include perceived ease of use; less time to interact with patients; issues with navigating complex interconnected systems and a concern that technology will replace the human interaction in caring professions (Gagnon et al., 2015, Piwek et al., 2016). In contrast, Benjamin and Jennings (2010) proposed the idea that technological advances may sweep away oppressive aspects of a technocratic culture, pointing out the progressive possibilities in new technologies of cultural production. The use of technology to enhance the user experience has been supported in the arts, especially within film, radio, and photography (Jameson, 1991, McRobbie, 2003, Thompson, 2015). Benjamin & Jennings (2010) concur, stating how new and contemporary forms of art that utilize technology maintain their cultural power through the aura of the authentic and original. Within this study, evaluation data suggested that a key value of the education programme was its use of technology. The use of VR as a tool for knowledge translation, provided participants with a sensory experience to facilitate emancipatory praxis. My study demonstrates how technology can contribute to progressive forms of education in midwifery aimed at improving care experiences. Although whilst an immersive critical pedagogy offers potential for reflection and emancipation, a social and political revolution is needed to unleash greater progress in the techno sciences to harness its implementation for the cause of human liberation (Horkheimer 2013).

Innovative approaches to cultural production, although largely disfavouring the use of technology, acknowledge its use in the creation of a new lens through which to critique forms of power, including epistemic power and its critique (Sandlim & Milam, 2008, Tatarchevskiy, 2011, Almer, 2015). Habermas (1987) urges people to search for truth in both discourse and an understanding of the individual human subject, demonstrated during delivery of the immersive educational programme (chapter 8 section 8.10). Discussions following VR viewing were seen to align with Kemmis' thoughts on critical reflection, in which it is noted that:

'Truth can only emerge in settings where all assertions are equally open to critical scrutiny, without fear or favour' (Kemmis, 1993 p, 49).

Participants reflected on how the immersive educational content provided them with unknown realities (first person perspective). This included a recognition of the dehumanising and oppressive nature of poor interpersonal interactions such as facing a computer, lack of eye contact, poor use of language etc (chapter 8 section 8.7). Other research has used similar methods to encourage first-person reflections to influence practice including simulated scenarios with actors to facilitate difficult situations in medical education (Teal et al., 2009, Little, 2015, Vincent & Amalberti 2016, Arakelian et al., 2017). Regardless of the breadth of literature highlighting the importance of using narratives and simulation as a means for quality improvement in services (NHS England, 2014), gaps exist in the consistency of how this in facilitated and used to drive change in maternity services (Wenzel & Jabbal, 2016).

Immersive scenarios offer an innovative and engaging way of presenting evidence-based feedback as part of a co-collaborative approach to knowledge production and translation. It is proposed within this thesis that first-person reflections and feedback from women in maternity care should not just serve as a vehicle to capture comfort measures, but become fully integrated into the system to drive change, learning and quality improvement strategies. This novel approach to presenting narratives to participants opens up unprecedented opportunities to use immersive narratives in maternity education to improve women's birth experiences, through the technological embodiment of the other.

9.12 VR - a tool for 'Conscientization'?

Although new in its application within healthcare settings, the use of VR spans the globe. Use cases of VR include the teaching of surgical techniques (Pelargos et al, 2017), alternative approaches to medical education (Huang et al, 2016, Gunn et al, 2018), used to improve the understanding and empathy towards people living with dementia (Wijma et al, 2018) and used more recently as a distraction technique to reduce pain in a multitude of settings (Atzori et al, 2019). Despite its many use cases, VR remains an unexplored method to instigate behavioural change amongst healthcare professionals where other approaches have been unsuccessful (Fertleman et al., 2018). To date, no study has explored the use of VR as part of an educational programme focused on

enhancing reflective practices and raising awareness of interpersonal interactions. Proposed within this thesis, implementing innovative critical pedagogical approaches in midwifery seeks to train critical and reflexive professionals, capable of problem solving and identifying oppressive aspects of care (Halman et al, 2017). This educational programme is arguably a critical pedagogy for the 21st century.

Moving at an unprecedented pace, it is important that innovators, researchers and educators approach the use of technology as an emancipatory endeavour with care and thought, ensuring that the correct ethical and moral frameworks are considered (Huda, 2019). In line with the critical stance taken within this study, it is also wise to acknowledge the disconnection between humans within the digital age, and how its application could be argued as a step towards the commercialisation and control over human autonomy. Current digital platforms of communication are equally changing the way humans interact on an interpersonal as well as a communicative level (Baym, 2015, Okdie et al., 2018). Bauman (2013) highlights how technology presents moral and ethical dilemmas regarding the essence of what it means to be human in the digital age. Despite these concerns' technology is at the forefront of strategies deployed by the DOH and NHS England aimed at improving outcomes and enhancing access to care, suggesting that technology has the power to transform services and save the NHS billions in cost and reduce health inequalities (DOH 2018, NHS England, 2019). That said, caution must be applied ensuring that technology is utilized within a humanistic framework to ensure its use is emancipatory opposed to exclusionary (Yaldren & Van Loo, 2018).

9.13 Conclusion

This discussions chapter has highlighted how interpersonal and spatial features of intrapartum care can influence disadvantaged and vulnerable women's experiences of birth, exacerbated by predefined judgements and beliefs held by maternity care professionals and compounded by complex life factors women may be facing. Key interpersonal factors that contribute to a traumatic birth experience transcend continents and economic structures, highlighting the need to raise awareness of birth trauma whist enhancing interpersonal midwifery skills to prevent its manifestation. Introducing resources focused on enhancing relational, respectful and humanistic care during birth, framed within a critical pedagogy, could encourage the acknowledgment

of political, social and inequality structures at play that impact upon maternity care experiences for women.

Through an exploration of the literature concerned with othering in healthcare and society, women's traumatic birth experiences have been situated within the current neoliberal rhetoric that arguably invades the moral foundations of some in society, impacting upon communicative discourses and care practices in healthcare settings. Operating under conditions of neoliberal modernity, negative care experiences were seen to exacerbate feelings of worthlessness and failure amongst disadvantaged and vulnerable women. This worrying trend in maternity care, in which maternity professionals (purposefully or inadvertently) contribute to and compound women's disadvantage and vulnerability, is seen to fuel a cyclical process of dehumanisation and inequality, as when women are not counted as a *'knower'* during a transformational process, such as childbirth, their belief in their ability and sense of achievement can be diminished beyond repair.

The value of critical pedagogies in midwifery education has also been discussed in context with the wider literature. Delivery of the immersive educational programme within this study was seen to empower and improve the knowledge of staff in relation to birth trauma, PTSD and associated risk factors. Attendance was also seen to facilitate critical thinking about processes that may cause situation of trauma and disrespectful care, whilst focusing on ways they could personally impact upon a women's birth experience. This chapter has discussed how immersive critical pedagogies could help illuminate how socially constructed power structures filter into relational care practices, both in terms of how this process affects organisational cultures and how it affects communication and interpersonal interaction between maternity professionals and women during birth.

9.14 Implications of study

Within this section, I discuss the impact of the thesis and the work within it. I begin by discussing implications for policy, practice and research, situating the study within a global context. I then discuss the study's strengths and limitations.

9.14.1 Implications for policy and practice

The *'Better Birth'* maternity review (NHS England, 2016) recognises the need for individualised, respectful and dignified care to optimise the birthing experience, with a

focus on continuity of carer and individualised care throughout the childbearing continuum. The intrapartum guidelines from the World Health Organization (2018) recently called upon maternity services to promote a positive pregnancy and birth to enable a successful transition into parenthood (WHO, 2016). Whilst it is vital that all women are given the opportunity for continuity and individualized support throughout pregnancy, the primacy of the social space of birth provides maternity professionals with an opportunity to positively impact upon the birth experience for women in the absence of relational based care models. The importance of education as a means of raising awareness of oppressive aspects of maternity care that may cause situations of trauma is recommended. Such interventions also provide an opportunity for staff to discuss/ reflect on practices on a regular basis facilitating the action – reflection cycle.

The need for specialist care pathways for women with complex needs has also been highlighted within this work. The needs for maternity services to consider the levels of support in place within their organizations to reduce the risk of women experiencing birth trauma and the support given throughout their childbearing continuum is timely. Interventions focused on these issues, such as specialised care pathways and dedicated specialist roles to provide additional support could bring about change by ensuring staff have more knowledge and awareness of factors that cause situations of trauma and that women at risk receive the most appropriate care for their needs. Ensuring such pathways are in place to support women at risk of birth trauma promotes a preventative approach to complications and chronic conditions associated with traumatic experiences.

It is also vital that services ensure staff feel supported in delivering care to those with complex needs, avoiding potential variations in service provisions across the UK. To facilitate such recommendations, additional funding to support NHS Trusts in delivering and invest in specialised care programmes is needed.

9.14.2 Implications for future research

A larger evaluation study exploring the impact of the educational programme across multiple NHS sites would also allow for a more comprehensive measurement of impact on women's experiences. The design and delivery of more evidence-based scenarios in relation to other areas of maternity could also provide insights into how immersive pedagogies influence relational care practices.

An area of research arising from this study is the exploration of maternity professionals' attitudes towards women with complex needs accessing services during labour and birth. That said, it is acknowledged that exploring personal opinions within a professional capacity is complex due to conflicting issues surrounding personal beliefs and professionals conduct. The use of ethnographic methods to observe midwives in practice may help to overcome this challenge and to assess potential variations in care provided to different groups of women.

A synthesis of quantitative data focused on disadvantaged and vulnerable women's maternity care experiences in high-income countries would be beneficial, as to validate findings from the meta-ethnography undertaken within this study. Potential future research could also explore the experiences of disadvantaged women using qualitative surveys. This would allow for a larger sample to data to be collected and potentially address the issue of capturing hard to-reach groups as completing surveys can be doing from home or online.

9.14.3 An emerging framework for creating immersive educational resources in healthcare

As highlighted in chapter three section 2.15 a gap has been identified regarding the creation of immersive content amongst researches and developers who may want to explore this approach. This study has developed an immersive evidence based educational resource, informed and creatively constructed using women's narratives, framed within a critical pedagogy. Further work is now needed to create an appropriate and robust framework for creating immersive content to promote critical thinking. In figure six key components to include within an immersive pedagogy are presented and serve as a guide for researchers who may wish to create similar immersive educational resources within a critical pedagogical framework.

Figure 6 list of key components included within immersive critical pedagogies

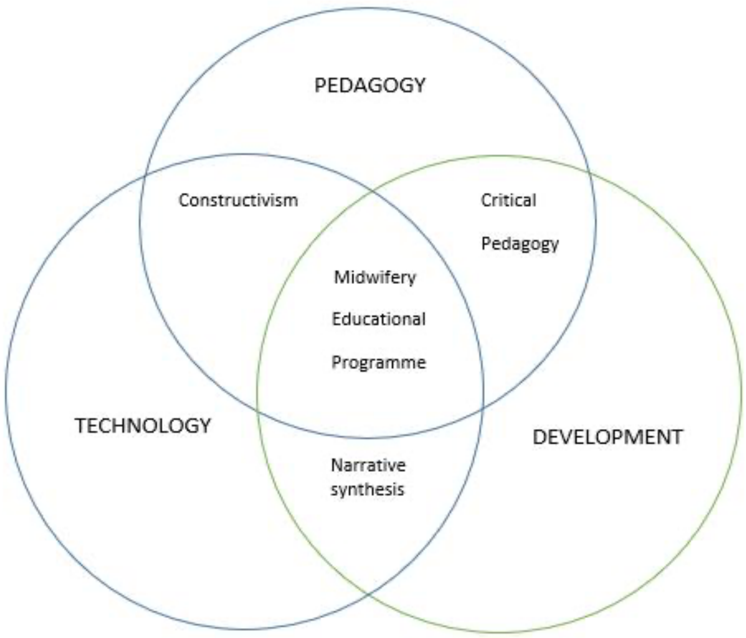

9.14.4 Strengths of the study

Informed by a robust systematic collection of empirical data, triggers for trauma have been identified and verified against the wider/international literature, highlighting the strength of this study's robust evidence base, ensuring content was representative of disadvantaged and vulnerable women who experience a traumatic birth. The use of a director and professional actors during filming stages also provided the programme with a professional approach to enhance the user experience, authentically representing women's narratives. This approach ensured the scenario was as representative as is possible, with a director possessing the knowledge and skills on how best to present non-verbal cues when attempting to present particular emotive triggers within scenes.

The use of a three-stage critical pedagogical framework was used to inform all stages of the education programme, providing a structured and systematic approach to developing and designing the programme. This approach has facilitated transparency of the research process whilst maintaining the essence of critical theory within the design. Following this approach ensures others are able to follow steps detailed, enabling them to replicate an immersive educational programme, should they wish to do so. Opportunities taken to gather pre and post data to assess potential impact on knowledge and capturing the discussions during delivery is also a strength of this study, providing me with points for reflection on how to develop and expand upon the work in the future.

9.14.5 Limitations of the study

Several limitations are apparent within this work and may therefore affect the transferability of its findings. A lack of representation for mothers born outside of the UK and those from black and ethnic minorities BAME communities is a limitation to the study's findings. Within this study, only one woman who was of Asian origin took part in the interviews. Issues relating to stigma and judgements were highlighted within the data. That said, this could have been an isolated incident, further strengthening the case for future research aimed at exploring care experiences for women from BAME backgrounds and hard to reach groups.

Women's lack of involvement in script development presents another limitation to this thesis. Although women's accounts were used to inform the script, the final narrative for the VR film was created by myself and my supervisory team. In line with critical approaches to research, the co-creation of knowledge through inclusive and emancipatory research endeavours would have presented this study with a purist approach to facilitating praxis (Jull, Giles & Graham 2017). Ledwith (2017) refers to this approach as a *'critical living praxis'* in that the unity of theory and action, based on lived experiences, provides us with the glue needed to integrate thinking and doing during emancipatory action research. To add, not only does critical living praxis provide a logical approach to co-creation, but it also encourages collaborative approaches to evaluation and feedback when developing or testing new interventions (Mauser et al., 2013). Adopting such a premise would have provided this research with a constant action reflection cycle, including both women and maternity staff throughout the design, development and evaluation stages.

Adopting evaluation methods that adopt a cyclic approach to action and evaluation would also have been useful, yet few existing methods for evaluating critical pedagogies have been designed. Murray et al (2016) believe that researchers should broaden their portfolio of evaluation methods during the research design phase, helping them develop the necessary knowledge base to inform decisions on policy, practice, and research in healthcare. To assess the impact the immersive educational programme may have had in practice, a larger study incorporating broader and more robust methods of evaluation into the research design should be undertaken.

The critical nature of questioning systems and behaviours makes evaluating service improvement interventions complex (Nielsen & Randall., 2013, Hall & Hall 2017), highlighting another limitation to the research. A further limitation relates to the pre/post questionnaires used to evaluate the training programme. These were designed with a pragmatic focus of eliciting insights into whether the training impacted on the midwives knowledge of risk factors for birth trauma and PTSD, and participants views on the acceptability of VR to facilitate reflection and the sensory experiences of its use. While a key method to assess fidelity included having critically reflective discussions during the training event and gathering qualitative insights from participants following the programme, it is recognised the more formal methods to assess the impact of the training (i.e. attitudes, beliefs, knowledge) could have been undertaken via use of validated questionnaires. Such an approach has been demonstrated in a recent study that evaluated a person-centred care evaluation instrument to assess the delivery of patient-centred care (PCC) to people with intellectual disabilities, as perceived by professionals (Cramm & Nieboer., 2017). The study rendered such a tool as a valid and reliable instrument for assessing the eight dimensions of PCC among professionals providing care, highlighting the possibility of adapting and using such an approach to measure the impact of the immersive educational programme.

Limitations also relate to staff not attending or being unable to attend the planned follow-up focus group due to work commitments. This highlights a need for organizational support and protected time for staff to fully engage with interventions aimed at service improvement, such as via integrating feasibility studies and research evaluations of educational interventions into mandatory training sessions. This said, systematic and directed approaches to evaluating interventions can be somewhat challenging when using a critical approach due to the fluid nature of knowledge acquisition, questions of power and oppression and the very nature of participant led approaches within this theoretical paradigm.

Limitations of this phase in the study also relates to the small sample size during empirical interviews with disadvantaged and vulnerable women (n=10). Restrictions also apply to the small geographical location in which women were recruited. Another possible limitation to this thesis is that the meta-ethnography focused on qualitative

studies only when gather data on women's experiences of midwifery care. Responses gathered from quantitative studies may have included statistical supportive data, further strengthening the narratives and themes that emerged. During the evaluation phase, limitations relate to the participants that attended the session. (n=2) of those that attended were working in joint office-based / clinical roles and (n=2) student midwives. This may have affected their response in relation to not being immersed in a birthing environment and labour ward culture, possibly influencing upon the authenticity and reliability of their accounts.

CHAPTER 10: TRANSLATING ACADEMIC OUTPUTS INTO TOOLS FOR IMPACT

10.1 Introduction

This chapter provides an overview of developments following delivery of the immersive educational programme, discussing activities undertaken aimed at maximising the programmes chances of implementation. I begin by presenting an overview of the collaboration between myself and the University of Central Lancashire's Business School. This section includes a discussion on the design and development of a marketing and campaign strategy, a working task, embedded within the BA Business course module *'Campaign Planning and Strategy'* for undergraduate students. I then present and detail the business analysis tool adopted to map the study within a marketing and business framework, producing a guide for the students undertaking the module. The final presentations developed and delivered by the students to an expert panel are then detailed, with the winning presentation and report provided within the appendices. I conclude this chapter by discussing the chosen campaign strategy and how this will be taken forward as a postdoctoral study.

10.2 Dissemination: developing a campaign and marketing strategy

The Improvement & Efficiency Directorate (2011) aimed at scaling up innovation in the NHS define innovation as:

> '*An idea, service or product, new to the NHS or applied in a way that is new to the NHS, which significantly improves the quality of health and care wherever it is applied'* (Improvement & Efficiency Directorate, 2011, p. 9).

Recently, Innovate UK invested over £15 million in innovative strategies and implementation methodologies aimed at addressing health care challenges in the NHS (Gov.Uk, 2016). However, despite these investments, barriers to implementing innovations present certain challenges, especially in the healthcare sector (Collins, 2018). A recent systematic review identified widespread failings in the adoption and spread of evidence-based interventions in healthcare (Cinar et al., 2019), with the Kings Fund stating the NHS spends less than 0.1 per cent of available resources on the

adoption and spread of innovation, compared to the 25 percent private sector multinational companies invest in this regard (Collins, 2018). Levine & Prietula (2013) encourage the identification of key barriers across disciplines and specialties in healthcare that may impact upon the development and delivery of an innovation, rather than adopting a linear approach to addressing context specific barriers. Key factors associated with unsuccessful implementation include self-interest, rivalry of goods and resources, complex systems and possible competition associated with open collaboration (Jacobs et al., 2015). Lau et al (2015) recommend that healthcare professionals and innovators embed evidence-based implementation strategies into their quality improvement approaches, but acknowledge the difficulties faced in this endeavour, with many clinicians not possessing the managerial and leadership skills to execute such an approach.

Although many support networks and strategic partners are commissioned by the government to collaborate with the NHS to help drive innovation, poor adoption statistics highlight potential system level failings and poor collaborative efforts (Murry et al., 2014, Fascia & Brodie, 2017, Collins, 2018). Key barriers include a lack of consideration for stakeholder engagement, cost saving targets favouring quick fixes over long term measurable outcomes (Lau et al., 2015), alongside poor strategic planning when attempting to implement change (Collins, 2018). With impending changes expected within the UK healthcare service, due to uncertainties such as Brexit, NHS Trusts are already being encouraged to foster partnerships with industry to enhance efficiency and procurement strategies (Li et al., 2018). Despite these changes opening up potential for investment in innovative approaches to healthcare challenges, corporate and industry investments also have the potential to widen the inequality gap (Moberley, 2019). Perusal of the literature concerned with adoption and spread of innovation revealed a gap in methodologies aimed at clinical partners (such as nurses, midwives, doctors and AHCP's) as opposed to commissioned partners (AHSN's Innovation agencies, CCG's), forcing me to think of innovative ways on how I could best maximise the chances of the immersive educational programme being adopted by NHS maternity Trusts in the UK.

I wanted to develop a working partnership that acknowledged the structural and institutional practices of the NHS, whilst appreciating the need for strategic planning

with key stakeholders. To do this, I needed business expertise, without losing sight of the humanistic and emancipatory ethos of my work. I was aware that this would involve a reliance upon my own moral compass and a pragmatic acceptance that exploring the commercial potential of the immersive educational programme, within an increasingly marketized public service, would present certain dilemmas. That said, to not consider available support to ensure uptake of the programme into practice, to me, represented something of an abrogation of commitment towards progressive change. Not seeking potential investment could also be regarded as a let-down concerning the emancipatory ideals and empowered contributions of the women who shared their birth stories with me. In this sense, my efforts to explore the business potential of the programme represent a reflexive approach to working within the interstices of a contemporary public service systems (Newman & Clarke, 2009).

Next steps included discussions between myself, the academic lead in the university's Business School and my lead supervisor Dr Gill Thomson. Following talks, it was decided that my study would act as a live project for the '*Campaign & Marketing Strategy*' module. This partnership enabled me to utilise my expertise as an academic and clinician and merge the expertise of the expert business team to write a module descriptor for the business students.

10.3 Working across-disciplines in healthcare innovation: developing a module descriptor

Once the decision had been made to use the study as a live project for the business students, the next step was to develop a module descriptor. The aim here was to provide students with a lay description of the study, background context to the aims of reducing traumatic birth experiences and details regarding the content and aims of the immersive educational programme. The module descriptor and outcomes can be seen in appendix 20. To ensure the descriptor met the learning outcome needs of the students I used the PESTEL tool (Collins, 2018). This tool enabled me to focus on action and business success using a structured analysis of the current factors that may influence success (Collins, 2018). The tool aimed to give a structured insight into the factors that may influence the educational programmes capability and potential uptake (P for Political, E for Economic, S for Social, T for Technological, L for Legal and E for Environmental) and is presented below in table 17.

Table 17 PESTEL tool used to map the study within a marketing and business framework.

Political	The current political climate has placed unprecedented pressures on the NHS. Current recommendations from the Department of Health (2010) and NHS England (2016) include addressing compassionate care and human factors in care delivery to foster improved outcomes. Within midwifery the recent National Maternity Review (2016) highlighted the need for reformative change within maternity services, emphasising a focus on women's choice, experiences and satisfaction with care. Unfortunately, women's experiences are not always positive which can lead to issues such as birth trauma, PTSD, poor mental health and difficulties in bonding with baby (Fenech & Thomson 2014, Ayers 2017, Beck 2018). Women with existing complex life issues, such as those from disadvantaged backgrounds, ethnic minorities and teenage mothers are more likely to have poor experiences of care (The World Health Organization 2007, Gamble & Creedy 2009).
Economic	A recent costing analysis highlighted that poor maternal health costs an estimated £8.1 billion every year (Bauer et al., 2014). Economically the impact of poor maternal mental health is undeniable and subsequently impacting on the economic stability and ability to deliver high quality maternal care to women.
Social	Poor birth experience, birth trauma and PTSD all impact upon a women's social wellbeing. PTSD can affect a woman's sense of self, isolate her from friends and family, have wider societal impacts such as lack of engagement with services, lack of self-care, lack of bonding with baby and family, physical symptoms of trauma such as flashbacks, etc (Ayers et al., 2016). The educational programme specifically focuses on woman-midwife social and interpersonal interactions; with a view to raise awareness of how these factors contribute to birth trauma and thereby operate to improve care practices.
Technological	This educational programme uses digital technology. The use of Virtual Reality technology enables the midwives to view poor experiences of care from a first-person perspective; the woman. The use of technology touches upon many different recommendations from the Department of Health (DOH) (2016) and The Kings Fund (2016). Simulated education has also been highlighted to lead to better outcomes in midwifery care enhance learning and developing skills related to real life situations (Black, 2018). The digital transformation of the NHS is currently pushing for technological solutions. Current innovations include online consultations with doctors, Apps to manage your own health records, amalgamating systems across services and VR technologies in surgery and medicine. This programme harnesses technology in education providing a technical solution to addressing human factors and interpersonal interactions in healthcare. Currently this structured programme using VR to educate midwives is the only one of its kind proving it with a unique selling point.
Legal	From a legal perspective this programme concentrates its focus on issues surrounding respectful care in healthcare. It addresses human rights issues by focussing on the importance of delivering respectful, safe and compassionate

	care. Mandatory training for midwives does not currently include any training on birth trauma or interpersonal interactions and human factors in care delivery. In line with the Nursing & Midwifery Council (NMC) all midwives and services delivering care to women have a *'Duty of care'* clause where they are bound to deliver respectful care. This educational programme addresses this clause to ensure staff are meeting legal requirements set by their professional body.
Environmental	Environmental issues relate to nurturing strong, competent and confident mothers by ensuring care is empowering rather than repressive. The focus of the educational programme is on what happens in the birth environment via communications and interactions with healthcare providers. This programme aligns with the millennium development goal number five set out by the United Nations (UN) through its focus on improving maternal health as a key area for global improvement (WHO, 2015). This provides the educational programme with scope to be place in a wider global context – we currently know care during childbirth can be very poor in developing nations. One of the main barriers for women to access maternity care is the fear of how they will be treated by healthcare staff (Bohren et al., 2015).

This framework provided students with key factors that would be influential in the likelihood of the programme's success as a business endeavour. It is important at this stage to acknowledge contradictions between adopting a critical approach to the design and delivery of the programme, to work on the scaling up of commercial interest in the educational programme. I had a '*crisis of conscience*[26]' during these stages and it felt important to discuss my reservations with my supervision team. My supervisors highlighted how the impact of the programme was the most important focus, and that any potential commercial returns would a by-product to help develop the programme further. When pitching and presenting to the business school students, I did brief them on my dilemma, ensuring they were aware of the emancipatory endeavour of the collaboration. This generated some interesting discussions in which we debated how commercial returns could be viewed as contradicting in an attempt to reduce health inequalities. I posed a hypothetical situation to the students in which some NHS Trusts may not be able to budget for the programme, prompting them to consider such barriers when designing their strategy. By placing the study in a wider context this encouraged

[26] A crisis of conscience is when someone feels worried or uncomfortable because they have done something which they think is wrong or immoral. In the case of this study I had prior beliefs about the encroachment of neoliberalism into healthcare through an engagement in critical literature, leading to me believing that business involvement represented a potential moral challenge.

students to think about the issues faced in attempting to drive innovation in a publicly funded institution such as the NHS and encouraged creativity as to how to address/moderate these concerns when designing their campaigns.

10. 4 Business school presentations

Eighty business students formed groups of 2-5 and had twelve weeks to work on developing a campaign and marketing strategy for disseminating and implementing the immersive educational programme into maternity education in the UK. At the end of this period, over the course of two days, the students presented their ideas to a panel in 20-minute slots (with and additional ten minutes for questions). The panel consisted of midwifery experts and a communication specialist from the NIHR (Myself, Peter Ashfield, Professor Soo Downe, Dr Gill Thomson, Dr Debby Kenny, Dr Anna Byrom, and Darren Charles[27]). The highest scoring campaign can be viewed in both appendix 21, which is the presentation the team delivered and in appendix 22 which is the report submitted as part of their assignment. It is important at this point to acknowledge and thank the students whose work is presented within this thesis: Harriet Hartley, Meghan Bone, Christian Hopes, Navin Persaud. The quality of work produced by these students is a perfect example of the dedication and passion instilled in students by their inspirational and dynamic course leader Peter Ashfield of the university's Business school.

10. 5 Summary

This part of the PhD was a harsh reality check regarding the complex institutional structures of the NHS and the irony that came with needing to engage in strategic marketing campaigns to enhance the uptake of emancipatory innovations. Issues relating to implementing interventions into practice were explored at a developmental, strategic and implementation level within the presentations. This was a useful endeavour during discussions with students on their chosen approaches and marketing campaigns to addressing such difficulties. These discussions allowed for a participatory approach to sharing expertise amongst both the panel and the students. I really enjoyed engaging and listening to their wonderfully dynamic and innovative strategies and felt I

[27] Please follow link for NIHR blog and photos from the delivery of the day. https://www.clahrc-nwc.nihr.ac.uk/news/article.php?title=Students+immerse+themselves+in+virtual+reality+pitch++

learnt a lot about how businesses such as the NHS operate. Their creative flair and input were outstanding. Working in collaboration with the business school most definitely enhanced my own understandings of influential factors within a business led model (the NHS) that can impede frontline innovations. I also hope this collaboration encouraged students to think about emancipatory approaches to business endeavours and how they may adopt these strategies in their future careers.

10. 6 Conclusion

Within this chapter I have detailed the collaborative endeavours between myself and the business school at the University of Central Lancashire. Following initial misgivings, I capitalised upon business orientated expertise without losing sight of the foundational ideals that underpin this thesis, with a view to maximising potential for uptake/implementation within the reality of the modern NHS business model. The next chapter presents my personal reflections of my PhD journey.

CHAPTER 11: PERSONAL REFLECTIONS

11.1 Introduction

This chapter represents the end of my journey. Here I reflect on the problems, difficulties and issues I have encountered whilst undertaking a PhD. In this chapter, I discuss my experiences as a researcher including reflections on engaging with participants, both with women during interviews and maternity professionals during the delivery of the programme. I also reflect upon my own personal and professional story. Through a reflexive process, I offer insights into how my initial standpoint has been questioned and transformed. Lastly, my final thoughts are offered, concluding the thesis and my PhD study.

11.2 Personal journey: Becoming an academic

Drawing upon a collection compiled by Mahony & Zmroczek '*Class Matters: 'Working Class' Women's Perspectives on Social Class'* (Mahoney & Zmroczek, 2005), I focus on the contributions of Diane Reay. Within her contribution to the collection; '*The Double-Bind of the 'Working-Class' Feminist Academic: The Success of Failure or the Failure of Success?'* she explores the difficulty faced in reconciling socialization into an academic culture, whilst holding a working-class identity. Reay discusses the struggles with identity and class consciousness amongst working class individuals within the academy, stating how this involves conflicting experiences, freighted with feelings of fictitious and fraudulent recognition. Throughout this journey I resonate with such sentiments. My journey to date has meant I have had to face my own battle into adulthood and academia in which I have metaphorically climbed the social ladder. A motherless teen born into a dysfunctional family, a single teenage mother and a social security claimant, it is fair to say that my identity was shaped in less than favourable circumstances. Reay identifies the contradictory position of the working-class female academic as inherently paradox to the radical potentialities of revisionist policies (Reay, 2002).

As an academic from a working-class background, I may be thought of as representing a justification of the right-wing rhetoric of meritocracy, an uncomfortable reality given the critical theoretical stance taken within this study. When reading back upon my reflexive diary, it became clear that my identity (a working-class woman), at times shadowed the reality of my position (an academic midwife researcher), highlighting the struggles I faced in accepting that my role as a researcher automatically created a power imbalance

when interviewing women. This realisation required me to consider my own issues with identity, requiring me to adopt a constant action reflection approach throughout my PhD journey. Secondly, I had to accept that my life experiences and self-identity could potentially impact upon the data, was I not complicit in the process of reflexivity throughout the journey of undertaking a PhD. Reflexivity acknowledges that:

> *'A researcher's background and position will affect what they choose to investigate, the angle of investigation, the methods judged most adequate for this purpose, the findings considered most appropriate, and the framing and communication of conclusions'* (Malterud, 2001, p. 483).

My drive to begin this journey was a quest to help others and to contribute to the field of midwifery research, focused on inequalities faced by disadvantaged and vulnerable women. Addressing potential biases and preconceptions of the data was a cathartic experience. By using a reflexive diary, having discussions with my supervisory team and accessing therapy during my PhD journey, I have grown in confidence and learnt a lot about myself and how my own experiences have helped shape and contribute to my own understandings of the world, whilst appreciating the lived experiences of others.

Although my birth experience was not traumatic, my experiences of navigating motherhood under complex life circumstances has provided me with a lived experience of disadvantage and vulnerability, something I believe was a strength when undertaking this research. While some researchers see these different ways of knowing as a reliability problem (Koch & Harrington 1998), others feel that these different ways of seeing provide a richer and more developed understanding of complex phenomena (Lincoln & Guba 1985, Barry et al., 1999, Berger, 2015). My role as a mother, midwife, nurse, researcher and woman have been an asset to me throughout this research journey, through which the reflexive process has enabled me to engage, understand, question, reflect and immerse myself in the processes, methods and data within this study.

11.3 Climbing the ladder

My upward social mobility has been one of the most challenging aspect of this journey. Very different from the competitive individualism rife in academia, my personal journey has been shaped by poverty, grief and community. Now holding a middle-class social

status, I have had to face many challenges in understanding and comprehending the culture of academic institutions. While I still accept there are established equalities programmes implemented within academic institutions, such as Athena Swan, none of these are explicitly focused on class. In defence of the academy, post 1992 universities, less hidebound to class elitism than Russel groups and Oxbridge, can be a place for critical ideas to flourish, yet my loss of identity seems to be an ongoing personal conflict that supersedes potential opportunities to feel accepted, encouraging me to think a little deeper about the psychosocial aspects of my journey to date.

These reflexive thoughts and ideas have raised interesting questions for me, namely the impact complex life experiences and social class may have on the professional performance and career choices of those pursuing academic pathways and to what extent the academy acknowledges the difficulties faced by those from disadvantaged backgrounds with socialisation into an unfamiliar environment. Trying to straddle the complex and interwoven hierarchies embedded within an academic institution, whilst attempting to dispel and address my own insecurities of not fitting in has and is an ongoing challenge.

11.4 Professional identity and conflicting feelings

Taking myself away from a full time clinical position as a midwife to undertake a full time PhD forced me to unearth and explore the resistance and difficulties I faced from a professional point of view. Perhaps one of the most challenging aspects of this transition was the prevailing anti-intellectualism I faced from colleagues, rife in the *'real'* world of clinical practice. This was the beginning of a crisis for me, in which I was attempting to maintain my clinical skill set and friendships with colleagues in practice, who in turn became distant when I was seen to represent an alien world from which they felt excluded. This felt exclusionary as my mission and drive for pursuing a PhD was/is to help improve care for women and their families, yet I began feeling mistrusted and under supported, seen as not *'one of us'* anymore when working clinically during bank shifts. My academic expertise was scrutinised in practice when working clinical shifts, making me feel under-valued as a midwife outside of the academy, further exacerbating feelings of not belonging.

Discussing these realties with my peers has been cathartic in enabling me to share my own experiences and to know I am not alone, yet questions remain. I can only relate

back to the arguments made within this thesis to offer an explanation, in that power resides in all spaces and to tackle such experiences of oppression, testimonial and hermeneutic injustices, individuals must first seek to emancipate themselves through inquiry, action reflection and a collective sense of ethical and social responsibility. I aim to continue striving for excellence in practice through an exploration of critical methods and lines of inquiry that seek to illuminate and explore these oppressive elements within midwifery that impact upon staff, women, patients, attitudes, behaviours and beliefs.

11.5 Final thoughts

This PhD journey has been enduring, exciting, but for the majority extremely anxiety provoking. Little did I know embarking upon a PhD would be such a transformative process. By utilizing a critical theoretical approach to analyse findings and to inform a critical pedagogy, I myself went through the process of *'conscientization'* through a constant reflection upon my own:

> '*Habits of thought, reading, writing, and speaking which go beneath surface meaning, first impressions, dominant myths, official pronouncements, traditional clichés, received wisdom, and mere opinions, to understand the deep meaning, root causes, social context, ideology, and personal consequences of any action, event, object, process, organisation, experience, text, subject matter, policy, mass media, or discourse.'* (Shor, 1992 p. 129).

This transformation was realised whist engaging and working in the world of academia in which my own preconceptions, social positioning, ideology, beliefs and biases were a constant reminder that situations of power and oppression are constant and require constant action-reflection to facilitate praxis. Becoming critically conscious through my own education resonates closely with the study and its aims. Embarking upon the journey to emancipation through immersion in the literature and the academy has provided me with points of reflection on my own struggles with *'Imposter syndrome'*[28].

Being reflexive has allowed me to take a critical stance on swapping newer forms of elitists oppressions and micro-discriminations, for older/previous disadvantages I have

[28] Imposter syndrome - A concept describing high-achieving individuals who are marked by an inability to internalize their accomplishments and a persistent fear of being exposed as a *'Fraud'*.

experienced within the working-class community, and when practicing as a midwife in the NHS. Many similarities exist within the structures of academia and that of healthcare providing me with a lived reality of the difficulties faced when working and communicating within a system built on power structures. Although imposter syndrome seems to be a constant state for me, engaging with the philosophical literature concerned with power discourses and oppressive social structures has only served to emancipate me both personally and professionally, through my own understanding of what it is to be human.

Questioning the ostensibly unquestionable premises of our way of life is arguably the most urgent of services we owe our fellow humans and ourselves.'
(Bauman, 1998, p. 41).

REFERENCES

Abbasi, K. (2018). The complexities and successes of the NHS. BMJ https://doi.org/10.1136/bmj.k2163 last accessed 24/06/2019

Abedian, Z., Narges, S., Mokhber, N., and Esmaily, H. (2013). Comparing post-traumatic stress disorder in primiparous and multiparous women with preeclampsia. *Journal of Midwifery and Reproductive Health* 1, 13–18. doi: 10.22038/jmrh.2013.755

Adamson, W. L. (1983). Hegemony and revolution: *A study of Antonio Gramsci's political and cultural theory*. University of California Press.

Adorno, T. W. (2005). *The culture industry: Selected essays on mass culture*. Routledge.

Agarwal, R., Gao, G., DesRoches, C., & Jha, A. K. (2010). Research commentary—The digital transformation of healthcare: Current status and the road ahead. *Information Systems Research*. 21(4), 796-809.

Alcorn, K L., O'Donovan, A., Patrick, J.C., Creedy, D., & Devilly, G.J. (2010). A prospective longitudinal study of the prevalence of post-traumatic stress disorder resulting from childbirth events. *Psychological Medicine*. 40, 1849–1859

Aliakbari, M., & Faraji, E. (2011). Basic principles of critical pedagogy. *In 2nd International Conference on Humanities, Historical and Social Sciences.* IPEDR (Vol. 17, pp. 78-85).

Alismail, H. A. (2015). Integrate Digital Storytelling in Education. *Journal of Education and Practice*. 6(9), 126-129.

Allen, S. (1998) A qualitative analysis of the process, mediating variables and impact of traumatic childbirth. *Journal of Reproductive Infant Psychology*. Special Issue: Postnatal Depress: Context Exp. 16, 107–131

Allmer, T. (2015). *Critical theory and social media: between emancipation and commodification*. Routledge.

Alpers, L. (2018). 3.10-P14 Ethnic minority patients experience othering and distrust. *The European Journal of Public Health*, *28*(suppl_1), cky048-110.

Alverson, D., Caudell, T., Goldsmith, T., & Riley, R. (2015). Creating virtual reality medical simulations: A knowledge-based design and assessment approach. *Manual of Simulation in Healthcare*. p. 411.

American Psychiatric Association, (1994) *Diagnostic and statistical manual of mental disorders* (4th ed.), Author, Washington, DC (1994).

American Psychiatric Association. (1980). *Diagnostic and statistical manual of mental disorders*; DSM-III. Washington DC.

Andersson, N. W., Hansen, M. V., Larsen, A. D., Hougaard, K. S., Kolstad, H. A., & Schlünssen, V. (2016). Prenatal maternal stress and atopic diseases in the child: a systematic review of observational human studies. *Allergy*. 71(1), 15-26.

Andreotti V and de Souza L. (2008) Global learning in the 'knowledge society' Four tools for discussion. Journal of International Educational Research and Development Education. 31(1): 7–12.

Arakelian, E., Swenne, C. L., Lindberg, S., Rudolfsson, G., & von Vogelsang, A. C. (2017). The meaning of person-centred care in the perioperative nursing context from the patient's perspective–an integrative review. Journal of clinical nursing, 26(17-18), 2527-2544.

Arthur, A., Unwin, S., & Mitchell, T. (2007). Teenage mothers' experiences of maternity services: a qualitative study. British Journal of Midwifery, 15(11), 672-677.

Asselin, M. E. (2003). Insider research: Issues to consider when doing qualitative research in your own setting. Journal for Nurses in Professional Development, 19(2), 99-103.

Atkins, S., Lewin, S., Smith, H., Engel, M., Fretheim, A., & Volmink, J. (2008). Conducting a meta-ethnography of qualitative literature: lessons learnt. BMC medical research methodology, 8(1), 21.

Atzori, B., Hoffman, H. G., Vagnoli, L., Messeri, A., & Grotto, R. L. (2019). Virtual reality as distraction technique for pain management in children and adolescents. In Advanced Methodologies and Technologies in Medicine and Healthcare (pp. 483-494). IGI Global.

Ayers, S., & Pickering, A. D. (2001). Do women get posttraumatic stress disorder as a result of childbirth? A prospective study of incidence. Birth, 28(2), 111-118.

Ayers, S., Bond, R., Bertullies, S., & Wijma, K. (2016). The aetiology of post-traumatic stress following childbirth: a meta-analysis and theoretical framework. Psychological Medicine, 46(6), 1121-1134.204.

Ayers, S., Claypool, J., & Eagle, A. (2006). What happens after a difficult birth? Postnatal debriefing services. British Journal of Midwifery, 14(3), 157-161.

Ayers, S., Eagle, A., Waring, H., (2006). The effects of childbirth-related posttraumatic stress disorder on women and their relationships: a qualitative study. Psychol. Health Med. 11, 389–398.

Ayers, S., Harris, R., Sawyer, A., Parfitt, Y., & Ford, E. (2009). Posttraumatic stress disorder after childbirth: Analysis of symptom presentation and sampling. Journal of Affective Disorders, 119, 200

Bailey, N., Brown, G., Letherby, G., Wilson, C., & Di Marco, H. (2004). Teenage pregnancy: medical encounters. British journal of midwifery, 12(11), 680-685.

Baker, P., Friel, S., Kay, A., Baum, F., Strazdins, L., & Mackean, T. (2018). What enables and constrains the inclusion of the social determinants of health inequities in

government policy agendas? A narrative review. International journal of health policy and management, 7(2), 101.

Bal, P. M., & Dóci, E. (2018). Neoliberal ideology in work and organizational psychology. *European Journal of Work and Organizational Psychology*. 27(5), 536-548.

Balaam, M. C., Cooper, M., Korfke, D., & Savona-Ventura, C. (2017). *Psychosocial Resilience and Risk in the Perinatal Period*: Implications and Guidance for Professionals Asylum seekers & refugees: A cross European perspective. P. 28-42.

Baldwin, A., Sobolewska, A., & Capper, T. (2018). Pregnant in prison: An integrative literature review. *Women and Birth*. https://doi.org/10.1016/j.wombi.2018.12.004 Last accessed 24/6/2019

Barley, E. (2017). Improving mental healthcare. *New Vistas.* 3(1), 4-10.

Barnett-Page, E., & Thomas, J. (2009). Methods for the synthesis of qualitative research: a critical review. *BMC medical research methodology*, 9(1), 59.

Baron, E. C., Hanlon, C., Mall, S., Honikman, S., Breuer, E., Kathree, T., ... & Patel, V. (2016). Maternal mental health in primary care in five low-and middle-income countries: a situational analysis. *BMC health services research*, *16*(1), 53.

Barry, CA., Britten, N., Barbar, N., Bradley, C. & Stevenson, F. (1999). *"Using reflexivity to optimize teamwork in qualitative research."* Qualitative Health Research. 9(1), 26-44.

Bastos MH, Furuta M, Small R, McKenzie-McHarg K, Bick D (2015) *Debriefing interventions for the prevention of psychological trauma in women following childbirth*. Cochrane Database Syst Rev.;4:CD007194

Bates, M. J. (1989). The design of browsing and berrypicking techniques for the online search interface. Online review, 13(5), 407-424.

Batt-Rawden SA, Chisolm MS, Anton B, Flickinger TE (2013) Teaching empathy to medical students: an updated, systematic review. Academic Medical. Vol 88. Pg 1171-7

Batty, C., Sawtell, L., & Taylor, S. (2016). Thinking through the screenplay: The academy as a site for research-based script development. *Journal of Writing in Creative Practice*, 9(1-2), 149-162.

Bauer, A. Parsonage, M. Knapp, M. Iemmi, V. Adelaja, B (2014) *Cost of Perinatal Mental Health Problems.* London School of Economics & Centre for Mental Health. http://www.centreformentalhealth.org.uk/costs-of-perinatal-mh-problems. Accessed 5/7/2017

Bauman, Z. (1991). The social manipulation of morality: Moralizing actors, adiaphorizing action. *Theory, Culture & Society*, 8(1), 137-151.

Bauman, Z. (1998). *Globalization: The human consequences.* Columbia University Press.

Bauman, Z. (2000) *Liquid Modernity.* Cambridge: Polity Press.

Bauman, Z. (2013). *Liquid modernity*. John Wiley & Sons.

Baym, N. K. (2015). *Personal connections in the digital age.* John Wiley & Sons.

Beauchamp, T. L., & Childress, J. F. (2001). Principles of biomedical ethics. Oxford University Press, USA.

Beck, C. T., & Watson, S. (2010). Subsequent childbirth after a previous traumatic birth. *Nursing Research*, 59(4), 241-249.

Beck, C.T., (2004a). Birth trauma: in the eye of the beholder. Nurs. Res. 53, 28–35 p. 32

Beck, C.T., (2004b) Post-traumatic stress disorder due to childbirth: the aftermath. Nurs. Res. 53, 216–224.

Beck, C.T., Watson, S. (2008) Impact of birth trauma on breastfeeding. Nurs. Res. 57, 228–236.

Beckett, P., Holmes, D., Phipps, M., Patton, D., & Molloy, L. (2017). Trauma-Informed Care and Practice: Practice Improvement Strategies in an Inpatient Mental Health Ward. Journal of psychosocial nursing and mental health services, 55(10), 34-38.

Benhabib, S. (2002). Political geographies in a global world: Arendtian reflections. Social Research: An International Quarterly, 69(2), 539-566.

Benjamin, W., & Jennings, M. W. (2010). The work of art in the age of its technological reproducibility [first version]. Grey Room, 11-37.

Benoit, C., Declercq, E., Murray, S. F., Sandall, J., Van Teijlingen, E., & Wrede, S. (2015). Maternity care as a global health policy issue. In The Palgrave international handbook of healthcare policy and governance (pp. 85-100). Palgrave Macmillan, London.

Benoit, C., Zadoroznyj, M., Hallgrimsdottir, H., Treloar, A., & Taylor, K. (2010). Medical dominance and neoliberalisation in maternal care provision: The evidence from Canada and Australia. Social science & medicine, 71(3), 475-481.

Berger, P. L., & Luckman, T. (1957). The Social Construction of Realitu. Garden Citu, NY: Anchor Books.—362–References.

Berger, P.L. and Luckmann, T. (1967). The Social Construction of Reality. New York: Anchor Books.

Berger, R. (2015). Now I see it, now I don’t: Researcher’s position and reflexivity in qualitative research. Qualitative research, 15(2), 219-234.

Bettany-Saltikov, J. (2012). How to do a systematic literature review in nursing: a step-by-step guide. McGraw-Hill Education (UK).

Bhui, K., Halvorsrud, K., & Nazroo, J. (2018). Making a difference: ethnic inequality and severe mental illness. The British Journal of Psychiatry, 213(4), 574-578.

Burch, R. L., & Gallup, G. G. (2004). Pregnancy as a stimulus for domestic violence. *Journal of family violence*, *19*(4), 243-247.

Birt, L., Scott, S., Cavers, D., Campbell, C., & Walter, F. (2016). Member checking: a tool to enhance trustworthiness or merely a nod to validation?. Qualitative health research, 26(13), 1802-1811.

Blaikie, N. (2009). Designing social research. Cambridge: Polity Press.

Bohman, J. (2005). Critical theory.

Bohren, M. A., Vogel, J. P., Hunter, E. C., Lutsiv, O., Makh, S. K., Souza, J. P., ... & Javadi, D. (2015). The mistreatment of women during childbirth in health facilities globally: a mixed-methods systematic review. PLoS medicine, 12(6), e1001847.

Boland, A., Cherry, G., & Dickson, R. (Eds.). (2017). Doing a systematic review: A student's guide. Sage.

Boorman, R. J., Devilly, G. J., Gamble, J., Creedy, D. K., and Fenwick, J. (2014). Childbirth and criteria for traumatic events. Midwifery 30, 255–261. doi: 10.1016/j.midw.2013.03.001

Booth, A., Sutton, A., & Papaioannou, D. (2016). Systematic approaches to a successful literature review. Sage.

Bourdieu, P. (1984). Distinction. Translated by Richard Nice. Cambridge, MA: Harvard Univer.

Bowen, E. A., & Murshid, N. S. (2016). Trauma-informed social policy: A conceptual framework for policy analysis and advocacy. American journal of public health, 106(2), 223-229.

Braun, V., & Clarke, V. (2006). Using thematic analysis in psychology. Qualitative research in psychology, 3(2), 77-101.

Bray, L., O'Brien, M. R., Kirton, J., Zubairu, K., & Christiansen, A. (2014). The role of professional education in developing compassionate practitioners: A mixed methods study exploring the perceptions xof health professionals and pre-registration students. Nurse education today, 34(3), 480-486.

Briscoe, L., & Lavender, T. (2009). Exploring maternity care for asylum seekers and refugees. British Journal of Midwifery, 17(1), 17-23.

Briscoe, L., Lavender, T., & McGowan, L. (2016). A concept analysis of women's vulnerability during pregnancy, birth and the postnatal period. *Journal of advanced nursing*, *72*(10), 2330-2345.

Britten, N., & Pope, C. (2011). Medicine taking for asthma: A worked example of meta-ethnography. Synthesizing qualitative research: Choosing the right approach, 41-57.

Brock, A. (2018). Critical technocultural discourse analysis. New Media & Society, 20(3), 1012-1030.

Bromley, P., Martin, C. J. H., & Patterson, J. (2017). Post traumatic stress disorder post childbirth versus postnatal depression: a guide for midwives. British Journal of Midwifery, 25(8), 484-490.

Broussard A, Weber-Breaux J (1994) Application of the childbirth belief-efficacy model in childbirth education classes. Journal of Perinatal Education. 1994;3(1):7–15.
Bryman, A. (2016). Social research methods. Oxford university press.

Bryman, A., & Burgess, B. (Eds.). (2002). Analyzing qualitative data. Routledge.

Burns, E., Fenwick, J., Sheehan, A., & Schmied, V. (2016). 'This little piranha': a qualitative analysis of the language used by health professionals and mothers to describe infant behaviour during breastfeeding. Maternal & child nutrition, 12(1), 111-124.

Burr, V. (1995). An introduction to social constructionism. London: Routledge.

Burr, V. (2003) Social Constructionism (2nd edition). Hove: Routledge.

Butler, J., & Spivak, G. C. (2007). Who sings the nation-state. Language, politics, belonging, 58-61.

Buultjens, M., Murphy, G., Robinson, P., & Milgrom, J. (2013). The perinatal period: A literature review from the biopsychosocial perspective. Clinical Nursing Studies, 1(3), 19-31..

Byrom, S., & Downe, S. (2015). The roar behind the silence: why kindness, compassion and respect matter in maternity care. London: Pinter & Martin. Pg 235-240

Campbell, R., Pound, P., Morgan, M., Daker-White, G., Britten, N., Pill, R., ... & Donovan, J. (2012). Evaluating meta ethnography: systematic analysis and synthesis of qualitative research.

Campbell, J. M., Klugar, M., Ding, S., Carmody, D. P., Hakonsen, S. J., Jadotte, Y. T., ... & Munn, Z. (2015). Diagnostic test accuracy: methods for systematic review and meta-analysis. *International journal of evidence-based healthcare*, *13*(3), 154-162.

Canales, M. K. (2000). Othering: Toward an understanding of difference. Advances in Nursing Science, 22(4), 16-31.

Carr, W., & Kemmis, S. (2003). Becoming critical: education knowledge and action research. Routledge.

Carroll, W. K. (2009). Hegemony, counter-hegemony, anti-hegemony. Socialist Studies/Études Socialistes, 2(2).

Carson A. (2001) That's another story: narrative methods and ethical practice. Journal of Medical Ethics. Vol 27 Pg 198-202

Carter, B (2004) How do you analyse qualitative data. In T Lavender, G Edwards & Z Alfirevic eds. Demystifying qualitative research in pregnancy and childbirth. Quay Books, 87107.

Carter, S. M., & Little, M. (2007). Justifying knowledge, justifying method, taking action: Epistemologies, methodologies, and methods in qualitative research. Qualitative health research, 17(10), 1316-1328.

Cheatham A and Shen E. (2003) Community-based participatory reseach with Cambodian girls in Long Beach, California. In: Minkler M and Wallerstein N (eds) Community-based participatory research for health. San Francisco: Jossey-Bass, 2003, pp.316–331.

Chomsky, N. (2017). Noam Chomsky: Neoliberalism is destroying our democracy (C. Lydon, Interviewer). The Nation.

Cinar, E., Trott, P., & Simms, C. (2019). A systematic review of barriers to public sector innovation process. Public Management Review, 21(2), 264-290.

Clare J (1993) Change the curriculum – or transform the conditions of practice? Nurse Educ Today. 1993;13:282–6.

Clare, J. (1993). A challenge to the rhetoric of emancipation: Recreating a professional culture. Journal of Advanced Nursing, 18, 1033-1038.

Cochrane, T. (2016). Mobile VR in education: From the fringe to the mainstream. International Journal of Mobile and Blended Learning (IJMBL), 8(4), 44-60.

Cohen, L., &Manion, L. (Eds.). (1994). Research methods in education (4th ed. ed.). London: Longman Pg 36.

Collins, B. (2018). Adoption and spread of innovation in the NHS. The King's Fund website. Available at: www. kingsfund. org. uk/publications/innovation-nhs.

Collins, P. H. (2016). Shifting the center: Race, class, and feminist theorizing about motherhood. In Mothering (pp. 45-65). Routledge.

Collins, P. H., & Bilge, S. (2016). Intersectionality. John Wiley & Sons.

Confidential Enquiry into Maternal and Child health (CEMACH) (2008). Why Children Die: A Pilot Study 2006. Children and Young People's Report. National Children's Bureau, London

Conrad, P. & Barker, K. K. (2010) The Social Construction of Illness: Key Insights and Policy Implications. Journal of Health and Social Behaviour. Vol. 51, No. 1, pp.6779.

Cookson, R., & Walden, A. (2017). Inequality and the NHS hospital crisis: why social disadvantage needs attention. British Journal of Hospital Medicine, 64-65.

Corbin J, Morse JM (2003) The unstructured interactive interview: Issues of reciprocity and risks when dealing with sensitive topics. Qualitative Inquiry ;9(3):335-54.

Cotterill, P. (1992). Interviewing women: Issues of friendship, vulnerability, and power. In Women's Studies International Forum (Vol. 15, No. 5-6, pp. 593-606). Pergamon

Coyne, I.T. (1997) Sampling in qualitative research. Purposeful and theoretical sampling; merging or clear boundaries. Journal of Advanced Nursing. 26: 623-630.

Cramm, J. M., & Nieboer, A. P. (2017). Validation of an instrument to assess the delivery of patient-centred care to people with intellectual disabilities as perceived by professionals. *BMC health services research*, *17*(1), 472.

Craswell, A., Moxham, L., & Broadbent, M. (2015). Midwives' concerns about a shift of focus to computers in maternity settings: technology invading birth. MIDIRS Midwifery Digest, 25(2), 147-153.

Creedy, D., Shochet, I., & Horsfall, J. (2000). Childbirth and the development of acute trauma symptoms. Birth, 27, 104–111.

Cresswell, M., & Spandler, H. (2013). The engaged academic: academic intellectuals and the psychiatric survivor movement. Social Movement Studies, 12(2), 138-154.
Creswell, J. W., & Clark, V. L. P. (2017). Designing and conducting mixed methods research. Sage publications

Creswell, J. W., & Creswell, J. D. (2017). Research design: Qualitative, quantitative, and mixed methods approaches. Sage publications

Creswell, J. W., & Plano Clark, V. L. (2011). *Designing and conducting mixed method research* (2nd ed.). Thousand Oaks, CA: Sage.

Creswell, J. W., & Poth, C. N. (2017). Qualitative inquiry and research design: Choosing among five approaches. Sage publications

Crispi, F., Naci, H., Barkauskaite, E., Osipenko, L., & Mossialos, E. (2018). Assessment of devices, diagnostics and digital technologies: a review of NICE medical technologies guidance. Applied health economics and health policy, 1-23.

Cross-Sudworth, F., Williams, A., & Herron-Marx, S. (2011). Maternity services in multi-cultural Britain: using Q methodology to explore the views of first-and second-generation women of Pakistani origin. Midwifery, 27(4), 458-468.

Crotty, M. (1998). The foundations of social research: Meaning and perspective in the research process. Sage.

Crouch C. (2011) The Strange Non-Death of Neoliberalism. Polity, Cambridge.

Culpan I and Bruce J. (2007) New Zealand physical education and critical pedagogy: Refocusing the curriculum. Int J Sport Health Sci 2007; 5: 1–11.

Curran, T., & Hill, A. P. (2017). Perfectionism is increasing over time: A meta-analysis of birth cohort differences from 1989 to 2016. Psychological Bulletin. in press. doi:10.1037/bul0000138

Currie, G., Lockett, A. and Enany, N.E. (2013). From what we know to what we do: Lessons learned from the translational CLAHRC initiative in England. Journal of Health Services Research & Policy, 18(3), 27-39.

Czarnocka, J., & Slade, P. (2000). Prevalence and predictors of post-traumatic stress symptoms following childbirth. British Journal of Clinical Psychology, 39(1), 35-51.

Dahlberg, U., & Aune, I. (2013). The woman's birth experience—The effect of interpersonal relationships and continuity of care. Midwifery, 29(4), 407-415.

Dahlen, H. G., Kennedy, H. P., Anderson, C. M., Bell, A. F., Clark, A., Foureur, M., ... & Downe, S. (2013). The EPIIC hypothesis: intrapartum effects on the neonatal

Daniela, L., & Lytras, M. D. (2019). Themed issue on enhanced educational experience in virtual and augmented reality. Virtual Reality, 1-3.

Darder, A., & Baltodano, M. (2003). The critical pedagogy reader. Psychology Press.

Data Protection Act (1998). Data protection act. London Station Off.

Davies, M. M., & Bath, P. A. (2001). The maternity information concerns of Somali women in the United Kingdom. Journal of Advanced Nursing, 36(2), 237-245.

de Faria, J. W. V., Teixeira, M. J., Júnior, L. D. M. S., Otoch, J. P., & Figueiredo, E. G. (2016). Virtual and stereoscopic anatomy: when virtual reality meets medical education. Journal of neurosurgery, 125(5), 1105-1111.

De Rijk, L. M. (2002). Aristotle: Semantics and Ontology: Volume I: General Introduction. The Works on Logic. Brill.

De Schepper, S., Vercauteren, T., Tersago, J., Jacquemyn, Y., Raes, F., & Franck, E. (2016). Post-Traumatic Stress Disorder after childbirth and the influence of maternity team care during labour and birth: A cohort study. Midwifery, 32, 87-92.

De Schepper, S., Vercauteren, T., Tersago, J., Jacquemyn, Y., Raes, F., & Franck, E. (2016). Post-Traumatic Stress Disorder after childbirth and the influence of maternity team care during labour and birth: A cohort study. Midwifery, 32, 87-92.

Dekel, S., Stuebe, C., & Dishy, G. (2017). Childbirth induced posttraumatic stress syndrome: a systematic review of prevalence and risk factors. Frontiers in psychology, 8, 560.

Denny, E. (2018). Respectful maternity care needs to be the standard for all women worldwide. BJOG: An International Journal of Obstetrics & Gynaecology.

Denzin, N. K./Lincoln, Y. S. (eds.), (1994) Handbook of Qualitative Research, Thousand Oaks: Sage Publications 1994.

Denzin, N. K./Lincoln, Y. S. (eds.), (1994) Handbook of Qualitative Research, Thousand Oaks: Sage Publications.

Department of Health (2018) The future of healthcare: our vision for digital, data and technology in health and care. The Department of Health. London https://www.gov.uk/government/publications/the-future-of-healthcare-our-vision-for-digital-data-and-technology-in-health-and-care

Department of Health, & Great Britain. Dept. of Health. (2006). Our health, our care, our say: a new direction for community services (Vol. 6737). The Stationery Office.

Department of Health. (1993). Changing childbirth: Report of the expert maternity group.

Department of Health. (2010). The NHS quality, innovation, productivity and prevention challenge: an introduction for clinicians.

DiCicco-Bloom B, Crabtree BF. (2006) The qualitative research interview. Med Education;40(4):314-21

Diemer, M. A., McWhirter, E. H., Ozer, E. J., & Rapa, L. J. (2015). Advances in the conceptualization and measurement of critical consciousness. The Urban Review, 47(5), 809-823.

Dixon-Woods, M. D., Kirk, M. D., Agarwal, M. S., Annandale, E., Arthur, T., Harvey, J., & Riley, R. (2005). Vulnerable groups and access to health care: a critical interpretive review. National Coordinating Centre NHS Service Delivery Organ RD (NCCSDO) Retrieved May, 27, 2012.

Donkin, A., & Marmot, M. (2017). HEALTH INEQUALITIES AND THE IMPORTANCE OF ACTION ON PERINATAL RISK FACTORS. Transforming Infant Wellbeing: Research, Policy and Practice for the First 1001 Critical Days, 8.

Downe, S., Finlayson, K., & Fleming, A. (2010). Creating a collaborative culture in maternity care. Journal of Midwifery & Women’s Health, 55(3), 250-254
.

Draper, E., Gallimore, I., Kurinczuk, J., Smith, P., Boby, T., Smith, L., & Manktelow, B. (2018). MBRRACE-UK perinatal mortality surveillance report, UK perinatal deaths for births from January to December 2016. Leicester: The Infant Mortality and Morbidity Studies, Department of Health Sciences, University of Leicester.

Dreher, J. (2016). The social construction of power: Reflections beyond Berger/Luckmann and Bourdieu. Cultural Sociology, 10(1), 53-68.

Dwyer, S. C., & Buckle, J. L. (2009). The space between: On being an insider-outsider in qualitative research. International journal of qualitative methods, 8(1), 54-63.

Easton, K.L., McComish, J.F., Greenberg, R., (2000) Avoiding common pitfalls in qualitative data collection and transcription. Qualitative Health Research, 10, 703–707

Ebert, L., Bellchambers, H., Ferguson, A., & Browne, J. (2014). Socially disadvantaged women's views of barriers to feeling safe to engage in decision-making in maternity care. Women and Birth, 27(2), 132-137.

Ehlers, A., & Clark, D. M. (2000). A cognitive model of posttraumatic stress disorder. Behaviour research and therapy, 38(4), 319-345.

Ekstrand, C., Jamal, A., Nguyen, R., Kudryk, A., Mann, J., & Mendez, I. (2018). Immersive and interactive virtual reality to improve learning and retention of neuroanatomy in medical students: a randomized controlled study. CMAJ open, 6(1), E103.

Elmir R, Schmied V, Wilkes L, Jackson D. 2010. Women's perceptions and experiences of a traumatic birth: a meta-ethnography. Journal of Advanced Nursing 66(10): 2142-53

Elsaesser, T., & Hagener, M. (2015). Film theory: An introduction through the senses. Routledge.

Engelhard, I. M., M. A. van den Hout, et al. (2001). "Posttraumatic stress disorder after pregnancy loss." General Hospital Psychiatry 23(2): 62-66

Englander, M. (2012) The Interview: Data collection in descriptive phenomenological human scientific research. Journal of Phenomenological Psychology. 43: 13-35.

Eriksson, A. (Ed.). (2015). Punishing the Other: The social production of immorality revisited. Routledge.

Etherington, K. (2004). Becoming a reflexive researcher: Using our selves in research. Jessica Kingsley Publishers.

Etikan, I., Musa, S. A., & Alkassim, R. S. (2016). Comparison of convenience sampling and purposive sampling. American journal of theoretical and applied statistics, 5(1), 1-4.

Fairburn, C. G., & Patel, V. (2017). The impact of digital technology on psychological treatments and their dissemination. Behaviour research and therapy, 88, 19-25.

Fanon, F. (1952/1986). Black skin, white masks. London: Pluto Press.

Fascia, M., & Brodie, J. (2017). Structural barriers to implementing open innovation in healthcare. British Journal of Healthcare Management, 23(7), 338-343.

Feder, G. S., Hutson, M., Ramsay, J., & Taket, A. R. (2006). Women exposed to intimate partner violence: expectations and experiences when they encounter health care professionals: a meta-analysis of qualitative studies. Archives of internal medicine, 166(1), 22-37.

Feenberg, A. (1991). Critical theory of technology (Vol. 5). New York: Oxford University Press.

Fenech, G., & Thomson, G. (2014). Tormented by ghosts from their past': a meta-synthesis to explore the psychosocial implications of a traumatic birth on maternal well-being. Midwifery, 30(2), 185-193.

Fernandez-Balboa J and Marshall J. (1994) Dialogical pedagogy in teacher education: Toward an education for democracy. Journal of Teaching Education 45: 172–182

Fertleman, C., Aubugeau-Williams, P., Sher, C., Lim, A. N., Lumley, S., Delacroix, S., & Pan, X. (2018). A discussion of virtual reality as a new tool for training healthcare professionals. Frontiers in public health, 6, 44.

Finfgeld-Connett, D. (2008). Meta-synthesis of caring in nursing. Journal of clinical nursing, 17(2), 196-204.

Firth, A. D., & Haith-Cooper, M. (2018). Vulnerable migrant women and postnatal depression: A case of invisibility in maternity services?. British Journal of Midwifery, 26(2), 78-84.

Flemming, D., Cress, U., Kimmig, S., Brandt, M., & Kimmerle, J. (2018). emotionalization in science communication: The impact of narratives and Visual representations on Knowledge gain and risk Perception. Frontiers in Communication, 3, 3.

Forssén AS (2012) Lifelong significance of disempowering experiences in prenatal and maternity care: interviews with elderly Swedish women. Qual Health Res 22(11): 1535–46. Doi

Foster, K., & Mckenzie, H. (2018). Educational approaches and activities to enhance emotional intelligence. In Emotional intelligence in health and social care (pp. 127-145). Routledge.

Foucault, M. (1980) Power/Knowledge: selected interviews and other writings, 197277. Brighton: Harvester.

Foucault, M. (1995) Discipline and Punish: The Birth of Prison. New York: Random House.

Foucault, M. (1976) History of Sexuality (The Will to Knowledge: History of Sexuality).

France, E. F., Ring, N., Thomas, R., Noyes, J., Maxwell, M., & Jepson, R. (2014). A methodological systematic review of what's wrong with meta-ethnography reporting. BMC medical research methodology, 14(1), 119.

Francis, D. & Hester, S. (2004) An Invitation to Ethnomethodology; Language, Society and Interaction, Sage, London.

Francis, R. (2013). Report of the Mid Staffordshire NHS Foundation Trust public inquiry: executive summary (Vol. 947). The Stationery Office.

Freedman, L. P., & Kruk, M. E. (2014). Disrespect and abuse of women in childbirth: challenging the global quality and accountability agendas. The Lancet, 384(9948), e42-e44.

Freire, P. (1972). Pedagogy of the Oppressed. 1968. Trans. Myra Bergman Ramos. New York: Herder. (p. 143)

Freire, P. (1993). Pedagogy of the city. Burns & Oates.

Freire, P. (2018). Teachers as cultural workers: Letters to those who dare teach. Routledge.

Fricker, M. (2009). Epistemic injustice: Power and the ethics of knowing. Oxford University Press.

Fromm, E. (1967). Socialist humanism: An international symposium.

Fromm, E., & Drabsch, B. (1985). Psychoanalysis and religion. Queensland Tape Service for the Handicapped.

Fuchs, C. (2016). Neoliberalism in Britain: from thatcherism to cameronism. tripleC: Communication, Capitalism & Critique. Open Access Journal for a Global Sustainable Information Society, 14(1), 163-188.

Fullerton, J. T., & Ingle, H. T. (2003). Evaluation strategies for midwifery education linked to digital media and distance delivery technology. Journal of midwifery & women's health, 48(6), 426-436.

Furber, C. M., & McGowan, L. (2011). A qualitative study of the experiences of women who are obese and pregnant in the UK. Midwifery, 27(4), 437-444

Gagnon, M. P., Ngangue, P., Payne-Gagnon, J., & Desmartis, M. (2015). m-Health adoption by healthcare professionals: a systematic review. Journal of the American Medical Informatics Association, 23(1), 212-220.

Gale, K. N., Heath, G., Cameron, E., Rashid, S., & Redwood, S. (2013). Using the framework method for the analysis of qualitative data in multi-disciplinary health researcH. *BMC Medical Research Methodology, 13*(117), 1-8.

Gamble, A. (2014). Austerity as statecraft.

Gamble, J. A., Creedy, D. K., Webster, J., & Moyle, W. (2002). A review of the literature on debriefing or non-directive counselling to prevent postpartum emotional distress. Midwifery, 18(1), 72-79.

Garthus-Niegel, S., von Soest, T., Vollrath, M. E., & Eberhard-Gran, M. (2013). The impact of subjective birth experiences on post-traumatic stress symptoms: a longitudinal study. Archives of women's mental health, 16(1), 1-10.

Gerdtz M, F. Daniel, C. Dearie, V. Prematunga, R. Bamert, M. Duxbury, J. (2013) The outcome of a rapid training program on nurses' attitudes regarding the prevention of aggression in emergency departments: a multi-site evaluation. International Journal of Nursing Studies. Vol 50 Pg 1434-45

Giroux, H. (2001). Theory and Resistance in Education: Towards a Pedagogy for the Opposition (revised and expanded). Westport, CT: Bergin & Garvey.

Giroux, H. (2018). Pedagogy and the politics of hope: Theory, culture, and schooling: A critical reader. Routledge.

Giroux, H. A. (1979). Paulo Freire's approach to radical educational reform.

Giroux, H. A. (1988). Teachers as intellectuals: Toward a critical pedagogy of learning. Greenwood Publishing Group.

Giroux, H., & McLaren, P. (1986). Teacher Education and the Politics of Engagement: The Case for Democratic Schooling. Harvard Educational Review, 56, 213-238.

Giroux, H, (1981) Ideology, Culture and the Process of Schooling (Temple University Press 1981) p. 26

Given LM, Saumure K (2008) Trustworthiness. In Given LM (Ed) The Sage Encyclopedia of Qualitative Research Methods. Sage Publications, Thousand Oaks CA.

Glass, R. D. (2001). On Paulo Freire's philosophy of praxis and the foundations of liberation education. Educational Researcher, 30(2), 15-25.

Goldman, A. (2004). Epistemology. In Fundamentals of Philosophy (pp. 23-47). Routledge.

Good, L., & Officer, C. (2012). Developing the culture of compassionate care: Creating a new vision for nurses, midwives and care-givers.

Goodman, K. W. (2015). Ethics, medicine, and information technology: Intelligent machines and the transformation of health care. Cambridge University Press.

Gramsci, A (1971) The Intellectuals", in *Selections from the Prison Notebooks*. Translated and Edited by Q. Hoare and G. N. Smith. New York: International Publishers, page 3-23;

Gramsci, A. (2005). The intellectuals. Contemporary Sociological Thought, 49, 60-69.

Gramsci, A. (2009). Hegemony, intellectuals and the state. Cultural theory and popular culture: A reader, 2, 210-216.

Gramsci, A (1971) *Selections from the Prison Notebooks of Antonio Gramsci*, New York, International Publishers.

Green, J., & Thorogood, N. (2018). Qualitative methods for health research. Sage.

Greenfield, M., Jomeen, J., & Glover, L. (2016). What is traumatic birth? A concept analysis and literature review. British Journal of Midwifery, 24(4), 254-267.

Greenfield, M., Jomeen, J., & Glover, L. (2019). 'It can't be like last time'-Choices made in early pregnancy by women who have previously experienced a traumatic birth. Frontiers in Psychology, 10, 56.

Greenhalgh T. (1999) Narrative based medicine in an evidence-based world. British Medical Journal. Pg 318:323

Greenhalgh, T., & Peacock, R. (2005). Effectiveness and efficiency of search methods in systematic reviews of complex evidence: audit of primary sources. Bmj, 331(7524), 1064-1065.

Grekin, R., & O'Hara, M. W. (2014). Prevalence and risk factors of postpartum posttraumatic stress disorder: a meta-analysis. Clinical Psychology Review, 34(5), 389-401.

Grix, J. (2002). Introducing students to the generic terminology of social research. Politics, 22: 175-186

Grote, N. K., Katon, W. J., Russo, J. E., Lohr, M. J., Curran, M., Galvin, E., & Carson, K. (2015). Collaborative care for perinatal depression in socioeconomically disadvantaged women: a randomized trial. *Depression and anxiety*, *32*(11), 821-834.

Grusec, J. E., & Hastings, P. D. (Eds.). (2014). Handbook of socialization: Theory and research. Guilford Publications.

Guarino, K., & Decandia, C. (2015). Trauma-informed care: An ecological response. *Journal of Child and Youth Care Work*, *24*, 7-32.

Gunn, T., Jones, L., Bridge, P., Rowntree, P., & Nissen, L. (2018). The use of virtual reality simulation to improve technical skill in the undergraduate medical imaging student. Interactive Learning Environments, 26(5), 613-620.

Gupta, J. A., & Richters, A. (2008). Embodied subjects and fragmented objects: Women's bodies, assisted reproduction technologies and the right to self-determination. Journal of Bioethical Inquiry, 5(4), 239-249

Habermas, J. (1984). The theory of communicative action (Vol. 2). Beacon press.

Habermas, J. (1987a). The Philosophical Discourse of Modernity (F. Lawrence, Trans. Original text in German 1985, 1st ed.). Cambridge: Polity Press.

Habermas, J. (1987b). The Theory of Communicative Action, Volume 2. Lifeworld and System: A Critique of Functionailst Reason (T. McCarthy, Trans. Original text in German 1981, 1st ed.). Cambridge: Polity Press.

Hall, P. (2005). Interprofessional teamwork: Professional cultures as barriers. *Journal of Interprofessional care*, 19(sup1), 188-196.

Hall, I., & Hall, D. (2017). *Evaluation and social research*. Macmillan International Higher Education.

Halman, M., Baker, L., & Ng, S. (2017). Using critical consciousness to inform health professions education. *Perspectives on Medical Education*, 6(1), 12-20.

Hammersley, M. (2003). Social research today: Some dilemmas and distinctions. *Qualitative Social Work*, 2(1), 25–44.

Hannes, K., & Lockwood, C. (2011). Pragmatism as the philosophical foundation for the Joanna Briggs meta-aggregative approach to qualitative evidence synthesis. *Journal of Advanced Nursing,* 67(7), 1632-1642.

Harden, J. (1996). Enlightenment, empowerment and emancipation: the case for critical pedagogy in nurse education. Nurse Education Today, 16(1), 32-37.

Hardt, M., & Negri, A. (2001). Empire. Harvard University Press.

Harkins, S., & Lugo-Ocando, J. (2016). How Malthusian ideology crept into the newsroom: British tabloids and the coverage of the 'underclass'. Critical Discourse Studies, 13(1), 78-93.

Harrington, A. (2005). Modern social theory. Oxford: Oxford University Press

Harris, R., & Ayers, S. (2012). What makes labour and birth traumatic? A survey of intrapartum 'hotspots'. *Psychology & health*, *27*(10), 1166-1177.

Harvey, D. (2005). *Neoliberalism: A brief history*. Oxford: Oxford University Press

Harvey, G., Fitzgerald, L., Fielden, S., McBride, A., Waterman, H., Bamford, D. and Boaden, R. (2011). The NIHR collaboration for leadership in applied health research and care (CLAHRC) for greater Manchester: Combining empirical, theoretical and experiential evidence to design and evaluate a large-scale implementation strategy. *Implementation Science*, 6 (1), 1-9.

Harvey, L. (2015). Beyond member-checking: A dialogic approach to the research interview. *International Journal of Research & Method in Education*, 38(1), 23-38.

Health and Social Care Act (2012) *Health and Social Care Act* 2012.

Health Education England HEE (2017). Stepping forward to 2020/21: The mental health workforce plan for England.

Heidigger, M. (1927). 1978 *Being and Time*.

Henriques, J., Hollway, W., Urwin, C., Venn, C.& Walkerdine, V. (1984). Changing the subject: Psychology, social regulation and subjectivity. London: Methuen.

Herrel, N., Olevitch, L., DuBois, D. K., Terry, P., Thorp, D., Kind, E., & Said, A. (2004). Somali refugee women speak out about their needs for care during pregnancy and delivery. The Journal of Midwifery & Women's Health, 49(4), 345-349.

Herringa, R. J. (2017). Trauma, PTSD, and the developing brain. Current psychiatry reports, 19 (10), 69.

Higgins J.P.T. & Green S.E. (2011) Cochrane Handbook for Systematic Reviews of Interventions Version 5.1.0 (updated March 2011). Available at: www.cochrane-handbook.org (11 August 2015).

Higgins, A., Tuohy, T., Murphy, R., & Begley, C. (2016). Mothers with mental health problems: Contrasting experiences of support within maternity services in the Republic of Ireland. *Midwifery*, *36*, 28-34.

Hill, E., & Firth, A. (2018). Positive birth experiences: a systematic review of the lived experience from a birthing person's perspective. https://core.ac.uk/download/pdf/153515855.pdf last accessed 5 6 2019

Hodnett, E.D. (2002) Pain and women's satisfaction with the experience of childbirth: A systematic review. American Journal of Obstetrics & Gynecology, 186, 160-172.

Hoffman ML (2000) Empathy and Moral Development: Implications for Caring and Justice. Cambridge, UK; New York, NY: Cambridge University Press.

Hollis, C., Morriss, R., Martin, J., Amani, S., Cotton, R., Denis, M., & Lewis, S. (2015). Technological innovations in mental healthcare: harnessing the digital revolution. The British Journal of Psychiatry, 206(4), 263-265.

Holloway I., Wheeler, S (1996) Qualitative Research for Nurses. Blackwell Science, Oxford.

Honeyman, M., Dunn, P., & McKenna, H. (2016). A Digital NHS. An introduction to the Digital agenda and plans for implementation.

Hopper, E. K., Bassuk, E. L., & Olivet, J. (2010). Shelter from the storm: Traumainformed care in homeless service settings. The Open Health Services and Policy Journal, 3, 80–100.Pg 56

Horkheimer, M. (1993). The present situation of social philosophy and the tasks of an institute for social research. Between philosophy and social science: Selected early writings, 11. Pg 21

Horkheimer, M. (2013). Critique of instrumental reason. Verso Trade.

Horkheimer, M., & Adorno, T. W. (1972). Dialectic of Enlightenment: Max Horkheimer and Theodor W. Adorono. Seabury Press.

Horkheimer, M., (1937) Critical Theory: Selected Essays. Trans. Matthew J. O'Connell et al. New York: Continuum.

Horkheimer, M (1982). Critical Theory Selected Essays.p 244 New York: Continuum Pub.

Horkheimer, M (1982). Critical Theory Selected Essays.p 244 New York: Continuum Pub.

Horton, R., & Astudillo, O. (2014). The power of midwifery. Lancet, 384(9948), 1075-6.

Houghton, C., Casey, D., Shaw, D., & Murphy, K. (2013). Rigour in qualitative case-study research. Nurse researcher, 20(4).

Houweling, Tanja AJ, Carine Ronsmans, Oona MR Campbell, and Anton E. Kunst. (2007) "Huge poor-rich inequalities in maternity care: an international comparative study of maternity and child care in developing countries." Bulletin of the World Health Organization 85 (2007): 745-754.

How, A. (2017). Critical theory. Macmillan International Higher Education.

Howard, H. (2015). Reducing stigma: Lessons from opioid-dependent women. Journal of Social Work Practice in the Addictions, 15(4), 418-438.

Huang, H. M., Liaw, S. S., & Lai, C. M. (2016). Exploring learner acceptance of the use of virtual reality in medical education: a case study of desktop and projection-based display systems. Interactive Learning Environments, 24(1), 3-19.

Huang, K. T., Ball, C., Francis, J., Ratan, R., Boumis, J., & Fordham, J. (2019). Augmented Versus Virtual Reality in Education: An Exploratory Study Examining Science Knowledge Retention When Using Augmented Reality/Virtual Reality Mobile Applications. Cyberpsychology, Behavior, and Social Networking, 22(2), 105-110.

Huda, M. (2019). Empowering application strategy in the technology adoption: insights from professional and ethical engagement. Journal of Science and Technology Policy Management, 10(1), 172-192.

Hughes, J. A., & Sharrock, W. W. (2016). The philosophy of social research. Routledge.

Hunter, B, Henley, J, Fenwick, J et al (2018) Work, Health and Emotional lives of Midwives in the United Kingdom: The UK WHELM Study. School for Health Sciences, Cardiff University.

Hunter, L. P. (2006). Women give birth and pizzas are delivered: language and western childbirth paradigms. Journal of Midwifery & Women's health, 51(2), 119-124.

Hyde, A., & Roche-Reid, B. (2004). Midwifery practice and the crisis of modernity: implications for the role of the midwife. Social science & medicine, 58(12), 2613-2623.

Ii, S. S., Fitzgerald, L., Morys-Carter, M. M., Davie, N. L., & Barker, R. (2018). Knowledge translation in tri-sectoral collaborations: An exploration of perceptions of academia, industry and healthcare collaborations in innovation adoption. Health Policy, 122(2), 175-183.

Improvement & Efficiency Directorate (2011) 'Innovation and Service Improvement' Pg 9

Iornside, P (2006) Using narrative pedagogy: learning and practising interpretive thinking. Journal of Advanced Nursing. Vol 55 Pg 478–486.

Jacob, J. D., Perron, A., & Corneau, P. (2016). Structural othering: Towards an understanding of place in the construction of disruptive subjectivities. *Power and the Psychiatric Apparatus: Repression, Transformation and Assistance*, 2014-149.

Jacobs, S. R., Weiner, B. J., Reeve, B. B., Hofmann, D. A., Christian, M., & Weinberger, M. (2015). Determining the predictors of innovation implementation in healthcare: a quantitative analysis of implementation effectiveness. BMC health services research, 15(1), 6.

Jakobsen, S. P., & Overgaard, C. (2018). 'They'll be judging us'a qualitative study of pregnant women's experience of being offered participation in a supportive intervention. Midwifery, 61, 81-87.

Jameson, F (1991) Postmodernism, or, the cultural logic of late capitalism. Durham, NC: Duke University Press

Janes, C. R., Chuluundorj, O., Hilliard, C. E., Rak, K., & Janchiv, K. (2006). Poor medicine for poor people? Assessing the impact of neoliberal reform on health care equity in a post-socialist context. Global Public Health, 1(1), 5-30.

Janoff-Bulman, R. (2010). Shattered assumptions. Simon and Schuster.

Jarosova, D., Gurkova, E., Palese, A., Godeas, G., Ziakova, K., Song, M. S., ... & Fras, M. (2016). Job satisfaction and leaving intentions of midwives: analysis of a multinational cross-sectional survey. Journal of nursing management, 24(1), 70-79.

Jarvis-Selinger S, Pratt DD, Regehr G (2012) Competency is not enough: integrating identity formation into the medical education discourse. Acad Med. 2012;87:1185–90.

Jayaweera, H., & Quigley, M. A. (2010). Health status, health behaviour and healthcare use among migrants in the UK: evidence from mothers in the Millennium Cohort Study. Social science & medicine, 71(5), 1002-1010.

Jensen, D. (2008). Dependability. The Sage encyclopedia of qualitative research methods, 1, 208-209.

Johnson, J. L., Bottorff, J. L., Browne, A. J., Grewal, S., Hilton, B. A., & Clarke, H. (2004). Othering and being othered in the context of health care services. Health communication, 16(2), 255-271.

Jomeen, J. (2010). Choice, control and contemporary childbirth: Understanding through women's stories. Radcliffe Publishing.

Jomeen, J., & Redshaw, M. (2013). Ethnic minority women's experience of maternity services in England. Ethnicity & health, 18(3), 280-296.

Jomeen, J., Glover, L. F., & Davies, S. A. (2009). Midwives' illness perceptions of antenatal depression. British Journal of Midwifery, 17(5).

Jull, J., Giles, A., & Graham, I. D. (2017). Community-based participatory research and integrated knowledge translation: advancing the co-creation of knowledge. *Implementation Science*, *12*(1), 150.

Jurecic A. (2013) Illness as narrative. University of Pittsburgh Press, Columbia University. Program in narrative medicine. Mission statement.www.narrativemedicine.org/mission.html.

Kemmis, S. (1993). Foucault, Habermas and Evaluation. Curriculum Studies, 1(1), 35-54.

Kincheloe, J. L., McLaren, P., & Steinberg, S. R. (2011). Critical pedagogy and qualitative research. The SAGE handbook of qualitative research, 163-177.

Kirkham, M., Stapleton, H., Curtis, P., & Thomas, G. (2002). The inverse care law in antenatal midwifery care. British Journal of Midwifery, 10(8), 509-513.

Kirkup, B. (2015). The report of the Morecambe Bay investigation. London: The Stationery Office.

Kitzinger, J. (1995). Qualitative research: introducing focus groups. Bmj, 311(7000), 299-302.

Klein, N. (2015). The Shock Doctrine: The Rise of Disaster Capitalism [translat.]. Dobraja kniga. Moscow (rus).

Knight, M. (2019). The findings of the MBRRACE-UK confidential enquiry into Maternal Deaths and Morbidity. Obstetrics, Gynaecology & Reproductive Medicine, 29(1), 21-23.

Koblinsky, M., Moyer, C. A., Calvert, C., Campbell, J., Campbell, O. M., Feigl, A. B., ... & McDougall, L. (2016). Quality maternity care for every woman, everywhere: a call to action. The Lancet, 388(10057), 2307-2320.

Koch, T. & Harrington, A. (1998). "Reconceptualizing rigour: The case for reflexivity." Journal of Advanced Nursing. 28(4), 882-890.

Kohlberg, L. (1958). The Development of Modes of Thinking and Choices in Years 10 to 16. *Ph. D. Dissertation*, University of Chicago.

Kotz, D. M. (2009). The financial and economic crisis of 2008: A systemic crisis of neoliberal capitalism. Review of radical political economics, 41(3), 305-317.

Kramer M. S., Seguin L., Lydon J., & Goulet L. (2000) Socio-economic disparities in pregnancy outcome: why do the poor fare so poorly? *Paediatr Perinat Epidemiol*. 2000; 14: 194–210. pmid:10949211

Krefting, L. (1991). Rigor in qualitative research: The assessment of trustworthiness. American Journal of Occupational Therapy, 45(3), 214-222.

Kyaw, B. M., Saxena, N., Posadzki, P., Vseteckova, J., Nikolaou, C. K., George, P. P., ... & Car, L. T. (2019). Virtual reality for health professions education: Systematic review and meta-analysis by the Digital Health Education collaboration. Journal of medical Internet research, 21(1), e12959.

Labonté, R., & Stuckler, D. (2016). The rise of neoliberalism: how bad economics imperils health and what to do about it. J Epidemiol Community Health, 70(3), 312-318.

Lange, M. M., Rogers, W., & Dodds, S. (2013). Vulnerability in research ethics: a way forward. *Bioethics*, *27*(6), 333-340.

Lau, Rosa, Fiona Stevenson, Bie Nio Ong, Krysia Dziedzic, Shaun Treweek, Sandra Eldridge, Hazel Everitt et al. (2015) "Effectiveness of strategies to facilitate uptake or implementation of complex interventions: A systematic review of reviews." In Implementation Science, vol. 10, no. S1, p. A67. BioMed Central, 2015.

Lauckner H, Doucet S (2012) Patients as educators: the challenges and benefits of sharing experiences with students. Medical Education Vol 46 Pg 992-1000.

Lavender, T., Walkinshaw, S. A., & Walton, I. (1999). A prospective study of women's views of factors contributing to a positive birth experience. Midwifery, 15(1), 40-46

Ledwith, M. (2007). On being critical: Uniting theory and practice through emancipatory action research. Educational action research, 15(4), 597-611.

Ledwith, M. (2011). Community development: A critical approach. Policy Press

Ledwith, M. (2015). Community development In Action: Putting Freire into practice. Policy Press.

Ledwith, M. (2017). Emancipatory action research as a critical living praxis: From dominant narratives to counternarrative. In *The Palgrave international handbook of action research* (pp. 49-62). Palgrave Macmillan, New York

Levine, S. S., & Prietula, M. J. (2013). Open collaboration for innovation: Principles and performance. Organization Science, 25(5), 1414-1433.

Levinson, B. A., Gross, J. P., Hanks, C., Dadds, J. H., Kumasi, K., & Link, J. (2015). Beyond critique: Exploring critical social theories and education. Routledge.

Lewis, C. (2019). Realising the digital ambitions of the NHS. Practice Management, 29(4), 20-22.

Lewis, L., Hauck, Y. L., Ronchi, F., Crichton, C., & Waller, L. (2016). Gaining insight into how women conceptualize satisfaction: Western Australian women's perception of their maternity care experiences. BMC pregnancy and childbirth, 16(1), 29.

Lewis, S., Collyer, F., Willis, K., Harley, K., Marcus, K., Calnan, M., & Gabe, J. (2018). Healthcare in the news media: The privileging of private over public. Journal of Sociology, 54(4), 574-590.

Lincoln, Y. S., & Guba, E. G. (1985). Establishing trustworthiness. Naturalistic inquiry, 289, 331.

Lincoln, Y. S./Guba, E. G. (1984) Naturalistic Inquiry, Beverly Hills: Sage Publications 1984.

Little, M. O. (2015). The use of critical reflection in clinical practice and health profession education. *Journal of the American Medical Directors Association*, 16(3), 177-178.

Littler, J. (2017). Against meritocracy: Culture, power and myths of mobility. Routledge.

Lowe, H. J., & Barnett, G. O. (1994). Understanding and using the medical subject headings (MeSH) vocabulary to perform literature searches. Jama, 271(14), 1103-1108.

Luna, F. (2009). Elucidating the concept of vulnerability: Layers not labels. *IJFAB: International Journal of Feminist Approaches to Bioethics*, *2*(1), 121-139.

Lupton, D. (2013). The digitally engaged patient: Self-monitoring and self-care in the digital health era. Social Theory & Health, 11(3), 256-270.

Macias-Konstantopoulos, W. L. (2017). Caring for the trafficked patient: ethical challenges and recommendations for health care professionals. *AMA journal of ethics*, 19(1), 80-90.

Macintyre, A., Ferris, D., Gonçalves, B., & Quinn, N. (2018). What has economics got to do with it? The impact of socioeconomic factors on mental health and the case for collective action. Palgrave Communications, 4(1), 10.

Mackenbach J. P., Stirbu I., Roskam A. J. R., Schaap. MM., Menvielle G., Leinsalu M. et al., (2008) Socioeconomic inequalities in health in 22 European countries. *New Eng J Med*. 2008; 359: E14–E14.

Mackintosh, N., Sandall, J., Collison, C., Carter, W., & Harris, J. (2018). Employing the arts for knowledge production and translation: Visualizing new possibilities for women speaking up about safety concerns in maternity. Health Expectations, 21(3), 647-658.

Macrine, S. (2016). Pedagogies of Neoliberalism, in *Springer*, *S*., *Birch*, *K*., and *MacLeavy*., J. (*eds*), The *Routledge Handbook of Neoliberalism (*ch.26) in (Abingdon: *Routledge*, *2016*). Pg 2

Maggioni, C., Margola, D., & Filippi, F. (2006). PTSD, risk factors, and expectations among women having a baby: A two-wave longitudinal study. Journal of Psychosomatic Obstetrics and Gynecology, 27, 81–90.

Mahony, P., & Zmroczek, C. (2005). Class Matters:" Working Class" Women's Perspectives On Social Class. Taylor & Francis.

Malterud, K. (2001). "Qualitative research: Standards, challenges and guidelines." The Lancet. 358: pp. 483-488.

Marcuse, H. (1969). An essay on liberation (Vol. 319). Beacon Press.

Marín-Morales, Javier, et al. (2018) "Affective computing in virtual reality: emotion recognition from brain and heartbeat dynamics using wearable sensors." Scientific reports 8.1 (2018): 13657.

Marmot, M. (2005). Social determinants of health inequalities. The lancet, 365(9464), 1099-1104.

Marmot, M., & Allen, J. J. (2014). Social determinants of health equity.

Marmot, M., (2010) Fair Society, Healthy Lives: A Strategic Review of Health Inequalities in England Post-2010. The Marmot Review, London. /http:// www.marmotreview.org/AssetLibrary/pdfs/Reports/FairSocietyHealthyLives.

Marryat, L., & Martin, C. (2010). Growing Up in Scotland: Maternal mental health and its impact on child behaviour and development.

Martin, G.P., Ward, V., Hendy, J., Rowley, E., Nancarrow, S., Heaton, J., Brittene, N., … Ariss, S. (2011). The challenges of evaluating large-scale, multipartner programmes: The case of NIHR CLAHRCs. Evidence & Policy: A Journal of Research, Debate and Practice, 7(4), 489-509.

Marx, K. (1904). A contribution to the critique of political economy (No. 1). International Library Publishing Company; London, Kegan Paul, Trench Trubner, Limited.

Marx, K. (1968). Eleven theses on Feuerbach, 1845 found in Karl Marx and Frederick Engels; selected works in one volume.

Mason, M. (2010). Sample size and saturation in PhD studies using qualitative interviews. Forum Qualitative Sozialforschung, 11(3)

Maternal Mental Health Alliance (2016) NHS England Launches Perinatal Mental Health Community Service Development Fund. http://maternalmentalhealthalliance.org/tag/everyones-business-campaign/page/4 (Last accessed: 1 April 2019)

Matthews, C. (2014). Critical pedagogy in health education. Health Education Journal, 73(5), 600–609. doi:10.1177/0017896913510511

Mattheys, K., Warren, J., & Bambra, C. (2018). “Treading in sand”: A qualitative study of the impact of austerity on inequalities in mental health. Social Policy & Administration, 52(7), 1275-1289.

Mauser, W., Klepper, G., Rice, M., Schmalzbauer, B. S., Hackmann, H., Leemans, R., & Moore, H. (2013). Transdisciplinary global change research: the co-creation of knowledge for sustainability. *Current Opinion in Environmental Sustainability*, *5*(3-4), 420-431.

May, T., & Williams, M. (2002). An introduction to the philosophy of social research. Routledge.

Maynard, A., & Williams, A. (2018). Privatisation and the National Health Service. In Privatisation and the welfare state(pp. 95-110). Routledge.

Mayo, P (1990) Gramsci, Freire, and Adult Education: Possibilities for Transformative Action, by Peter Mayo, Macmillan, 1999, ISBN 1-85649-614-7, pg 5

McKeown, M., Jones, F. & Spandler, H. (2018) Conscientization and transformation in the workplace: new forms of democracy for mental health services. In A. Melling & R. Pilkington (eds) Paulo Freire and Transformative Education: Changing Lives and Transforming Communities. Palgrave Macmillan, Basingstoke.

McKeown, M., Malihi-Shoja, L., & Downe, S. (2011). Service user and carer involvement in education for health and social care: Promoting partnership for health (Vol. 9). John Wiley & Sons.

McLaren, P. (2015). Life in schools: An introduction to critical pedagogy in the foundations of education. Routledge.

McLeish, J., & Redshaw, M. (2019). Maternity experiences of mothers with multiple disadvantages in England: A qualitative study. Women and Birth, 32(2), 178-184.

McNiff, J. (2013). Action research: Principles and practice. Routledge. Pg 50
McRobbie, A., 2003. Postmodernism and popular culture. London: Routledge.

Mead, G. H. (1934). Mind, self and society (Vol. 111). University of Chicago Press.: Chicago.

Meier, K. J. (2016). The Politics of Sin: Drugs, Alcohol and Public Policy: Drugs, Alcohol and Public Policy. Routledge.

Ménage, D., Bailey, E., Lees, S., & Coad, J. (2017). A concept analysis of compassionate midwifery. Journal of advanced nursing, 73(3), 558-573.

Mental Health Taskforce (2016) The Five Year Forward View for Mental Health. http://www.england.nhs.uk/wp-content/uploads/2016/02/Mental-Health-Taskforce-FYFV-final.pdf (Last accessed: 1 April 2019)

Mercer, D., & Flynn, M. (2017). Neoliberal demolition of the NHS: challenges of caring in a corporate culture. Critical approaches in nursing theory and nursing research: Implications for nursing practice, 14-33.

Methot, J. R., Lepine, J. A., Podsakoff, N. P., & Christian, J. S. (2016). Are workplace friendships a mixed blessing? Exploring tradeoffs of multiplex relationships and their associations with job performance. Personnel psychology, 69(2), 311-355.

Miles, R. (1989). Racism. London: Routledge.

Mill, J. E., Allen, M. N., & Morrow, R. A. (2016). Critical theory: Critical methodology to disciplinary foundations in nursing. Canadian Journal of Nursing Research Archive, 33(2).

Miller, S., Abalos, E., Chamillard, M., Ciapponi, A., Colaci, D., Comandé, D., ... & Manuelli, V. (2016). Beyond too little, too late and too much, too soon: a pathway towards evidence-based, respectful maternity care worldwide. The Lancet, 388(10056), 2176-2192.

Mind (2014) http://www.mind.org.uk/news-campaigns/news/mind-reveals-unacceptably-low-spending-on-public-mental-health/#.VrC6z7KLTIU Last accessed 20/01/2016

Mkandawire-Valhmu, L. (2018). Cultural Safety, Healthcare and Vulnerable Populations: A Critical Theoretical Perspective. Routledge.

Moberly, T. (2019). NHS joint working with industry is out of public sight. BMJ, 364, l1353.

Mold, A. (2015). Making British patients into consumers. The Lancet, 385(9975), 1286-1287.

Moreton, R. Adamson, J. Robinson, S. Richards, N. and Howe, P. (2016) Fulfilling lives: supporting people with multiple needs. Annual report of the national evaluation.

http://mcnevaluation.co.uk/wpfb-file/fulfilling-lives-multiple-needs-evaluation-annualreport-2016-pdf/

Morgan, G. (2015). Elites, varieties of capitalism and the crisis of neoliberalism. Research in the Sociology of Organizations, 43, 55–80.

Morse, J. M. (Ed.). (1994). Critical issues in qualitative research methods. Sage.

Morse, J. M., & Niehaus, L. (2009). *Mixed method design: Principles and procedures*. Walnut Creek, CA: Left Coast Press.

Moskovitz, L., & Garcia-Lorenzo, L. (2016). Changing the NHS a Day at a Time: The Role of Enactment in the Mobilisation and Prefiguration of Change.

Murray, R., Imison, C., & Jabbal, J. (2014). Financial failure in the NHS. What causes it and how best to manage it [Internet]. London: The Kings Fund.

Murray, E., Hekler, E. B., Andersson, G., Collins, L. M., Doherty, A., Hollis, C., ... & Wyatt, J. C. (2016). Evaluating digital health interventions: key questions and approaches

Muskett, C. (2014). Trauma-informed care in inpatient mental health settings: A review of the literature. International journal of mental health nursing, 23(1), 51-59.

Nadin, S., & Cassell, C. (2006). The use of a research diary as a tool for reflexive practice: Some reflections from management research. Qualitative Research in Accounting & Management, 3(3), 208-217.

National Audit Office. (2013). Maternity services in England.

National Institute for Health and Clinical Excellence. (2010). *Pregnancy and complex social factors: a model for service provision for pregnant women with complex social factors*. NICE.

Nielsen, K., & Randall, R. (2013). Opening the black box: Presenting a model for evaluating organizational-level interventions. *European Journal of Work and Organizational Psychology*, *22*(5), 601-617.

Newburn M and Fletcher G (2015). Running your maternity services liaison committee: a practical guide from good practice to troubleshooting, London: NCT.

Newman, J., & Clarke, J. (2009). Publics, politics and power: Remaking the public in public services. Sage.

Newnham, E. C. (2014). Birth control: Power/knowledge in the politics of birth. Health Sociology Review, 23(3), 254-268.

NHS England (2014). Five year forward view.

NHS England (2016) National Maternity Review. Better Births. Improving outcomes of Maternity Services in England. A five year forward view for maternity care. NHS England. https://www.england.nhs.uk/wp-content/uploads/2016/02/national-maternity-review-report.pdf

England, N. H. S. (2017). Five Year Forward View for Mental Health: One year on. *Publications Gateway Reference*, *6480*.

NHS England (2018). NHS Improvement, National Collaborating Centre for Mental Health (2018) The Perinatal Mental Health Care Pathways.

NHS England (2019). The NHS long term plan.

Nicholls, K., Ayers, S., (2007) Childbirth-related post-traumatic stress disorder in couples: a qualitative study. Br. J. Health Psychol. 12, 491–509

Nichols F. H, Gennaro S. (2000). The childbirth experience. In F. H. Nichols & S. S. Humenick (Eds.), Childbirth education: Practice, research and theory (2nd ed., pp. 66–83). Philadelphia: W. B. Saunders.

Nicholson, D. T., Chalk, C., Funnell, W. R. J., & Daniel, S. J. (2006). Can virtual reality improve anatomy education? A randomised controlled study of a computer-generated three-dimensional anatomical ear model. Medical education, 40(11), 1081-1087.

Nkansah-Amankra, S. S., Diedhiou, A., & Mendoza, J. (2018). Elaborating population health inequalities in the United States: maternity care in the era of free market system of neoliberalization. Int J Pregn & Chi Birth, 4(3), 143-153.

Noblit, G. W., & Hare, R. D. (1988). Meta-ethnography: Synthesizing qualitative studies (Vol. 11). sage.

Nursing & Midwifery Council. (2018). *The code: Professional standards of practice and behaviour for nurses, midwives and nursing associates*. London: Nursing & Midwifery Council.

Ny, P., Plantin, L., Karlsson, E. D., & Dykes, A. K. (2007). Middle Eastern mothers in Sweden, their experiences of the maternal health service and their partner's involvement. Reproductive Health, 4(1), 9.

Nyirenda, J. E. (1996). The relevance of Paulo Freire's contributions to education and development in present day Africa. Africa Media Review. Retrieved from http://sanweb.lib.msu.edu/DMC/African%20Journals/ pdfs/africa%20media%20review/vol10no1/jamr010001002.pdf Last accessed 22/02/2018

Oakley A. (1981) Interviewing women: a contradiction in terms. In: Roberts H, editor. Doing feminist research. London: Routledge, 1981: 30-61.

Office For National Statistics ONS (2011) http://www.ons.gov.uk/ons/rel/census/2011-census-analysis/health-gaps-by-socio-economic-position-of-occupations-in-england--wales--english-regions-and-local-authorities--2011/rpt-health-gaps-2011.html Last accessed 22/02/2016

O'Hara, M. W., and McCabe, J. E. (2013). Postpartum depression: current status and future directions. Annu. Rev. Clin. Psychol. 9, 379–407. doi: 10.1146/annurev-clinpsy-050212-185612

Okdie, B. M., & Ewoldsen, D. R. (2018). To boldly go where no relationship has gone before: Commentary on interpersonal relationships in the digital age. The Journal of social psychology, (just-accepted).

Okolie, A. C. (2003). Introduction to the Special Issue--Identity: Now You Don't See It; Now You Do. Identity: An International Journal of Theory and Research, 3(1), p. 2

Olde, E., van der Hart, O., Kleber, R. J., van Son, M. J., Wijnen, H. A., & Pop, V. J. (2005). Peritraumatic dissociation and emotions as predictors of PTSD symptoms following childbirth. Journal of Trauma & Dissociation, 6(3), 125-142.

Olde, E., van der Hart, O., Kleber, R., and van Son, M. (2006). Posttraumatic stress following childbirth: a review. Clin. Psychol. Rev.26, 1–16. doi: 10.1016/j.cpr.2005.07.002

Olenick, M., Flowers, M., Muñecas, T., & Maltseva, T. (2019). Positive and Negative Factors That Influence Health Care Faculty Intent to Engage in Interprofessional Education (IPE). In Healthcare (Vol. 7, No. 1, p. 29). Multidisciplinary Digital Publishing Institute.

Olmos-Raya, E., Ferreira-Cavalcanti, J., Contero, M., Castellanos-Baena, M. C., Chicci-Giglioli, I. A., & Alcañiz, M. (2018). Mobile Virtual Reality as an Educational Platform: A Pilot Study on the Impact of Immersion and Positive Emotion Induction in the Learning Process. Eurasia Journal of Mathematics Science and Technology Education, 14(6), 2045-2057.

Orsolini, L., Valchera, A., Vecchiotti, R., Tomasetti, C., Iasevoli, F., Fornaro, M., ... & Bellantuono, C. (2016). Suicide during perinatal period: epidemiology, risk factors, and clinical correlates. *Frontiers in psychiatry*, *7*, 138.

Ozer, E., Best, S., Lipsey T., & Weiss, D. (2008). Predictors of posttraumatic stress disorder and symptoms in adults: A meta-analysis. Psychological Bulletin, 129, 52-73. doi: 10.1037/1942-9681.S.1.3

Padilha, J. M., Machado, P. P., Ribeiro, A., Ramos, J., & Costa, P. (2019). Clinical Virtual Simulation in Nursing Education: Randomized Controlled Trial. Journal of medical Internet research, 21(3), e11529.

Pairman, S. (2006). Midwifery partnership: working ‘with’ women. The new midwifery: Science and sensitivity in practice, 73-97.

Parfitt, Y. M. and S. Ayers (2008). "The effect of post-natal symptoms of post-traumatic stress and depression on the couple's relationship and parental baby bond." Journal of Reproductive and Infant Psychology 99999(1): 1 - 16.

Patten, M. L. (2016). Questionnaire research: A practical guide. Routledge

Patterson, J., Hollins Martin, C., & Karatzias, T. (2018). PTSD post-childbirth: a systematic review of women's and midwives' subjective experiences of care provider interaction. Journal of reproductive and infant psychology, 1-28.

Patterson, J., Martin, C. J. H., & Karatzias, T. (2019). Disempowered midwives and traumatised women: exploring the parallel processes of care provider interaction that contribute to women developing Post Traumatic Stress Disorder (PTSD) post childbirth. Midwifery.

Patton, M. Q. (2002). Qualitative research and evaluation methods. Thousand Oaks. Cal.: Sage Publications.

Pearson, M. (2018). Educating student midwives about compassion: A critical reflection. British Journal of Midwifery, 26(4), 261-263.

Pelargos, P. E., Nagasawa, D. T., Lagman, C., Tenn, S., Demos, J. V., Lee, S. J., ... & Bari, A. (2017). Utilizing virtual and augmented reality for educational and clinical enhancements in neurosurgery. Journal of Clinical Neuroscience, 35, 1-4..

Petersen, A. R., & Bunton, R. (Eds.). (1997). Foucault, health and medicine. Psychology Press.

Piwek, L., Ellis, D. A., Andrews, S., & Joinson, A. (2016). The rise of consumer health wearables: promises and barriers. PLoS Medicine, 13(2), e1001953.

Platkin, C., Link, A. R., & Kwan, A. (2017). The Digital Revolution and Its Potential Impact on Detection and Treatment of Depressive Disorders. Public Health Perspectives on Depressive Disorders, 380.

Polit DF, Beck CT (2010) Generalization in quantitative and qualitative research: Myths and strategies. Int J Nurs Studies. 47 (11): 1451-1458. 10.1016/j.ijnurstu.2010.06.004

Popay, J.M., Porroche-Escudero, A., Sadler, G. and Simpson, S. (2017). The CLAHRC NWC health inequalities assessment toolkit Version 3. The national institute of health research collaboration for leadership in applied health research and care North West coast and Lancaster University.

Power, A. (2016). Midwifery in the 21st century: Are students prepared for the challenge? British Journal of Midwifery, 24(1), 66-68.

Prasad, P. (2017). Crafting qualitative research: Beyond positivist traditions. Routledge.

Pratt-Eriksson, D., Bergbom, I., & Lyckhage, E. D. (2014). Don't ask don't tell: Battered Women living in Sweden encounter with healthcare personnel and their experience of the care given. International journal of qualitative studies on health and well-being, 9(1), 23166.

Price, S., & Mitchell, M. (2004). Teenagers' experiences of the maternity services. Evidence-Based Midwifery, 2(2), 66-71.

Psarros, A. (2014). Women's Voices on Health. Addressing Barriers to Accessing Primary Care. Pg 4.

PTSD. (n.d.) Collins English Dictionary – Complete and Unabridged, 12th Edition 2014. (1991, 1994, 1998, 2000, 2003, 2006, 2007, 2009, 2011, 2014). Retrieved April 25 2019 from https://www.thefreedictionary.com/PTSD

Public Health England (2014) UCL Institute for Health Equity. 'Local action on health inequalities: evidence papers'. Available at: www.gov.uk/government/publications/local-action-on-healthinequalities-evidence-papers. Last accessed 22/02/2016

Puffett, N. (2018). Mapping children's centre cuts. Children and Young People Now, 2018(6), 15-15.

Racine L, Proctor P, Jewell LM (2012) Putting the world as classroom: an application of the inequalities imagination model in nursing and health education. J Transcult Nurs. 2012;23:90–9.

Raine, R., Cartwright, M., Richens, Y., Mahamed, Z., & Smith, D. (2010). A qualitative study of women's experiences of communication in antenatal care: identifying areas for action. Maternal and Child Health Journal, 14(4), 590-599.

Rattray, J., & Jones, M. C. (2007). Essential elements of questionnaire design and development. Journal of clinical nursing, 16(2), 234-243.

Rayment-Jones, H., Murrells, T., & Sandall, J. (2015). An investigation of the relationship between the caseload model of midwifery for socially disadvantaged women and childbirth outcomes using routine data–a retrospective, observational study. *Midwifery*, *31*(4), 409-417.

Rayment-Jones, H., Butler, E., Miller, C., Nay, C., & O'Dowd, J. (2017). A multisite audit to assess how women with complex social factors access and engage with maternity services. Midwifery, 52, 71-77.

Reay, D. (2002). Class, authenticity and the transition to higher education for mature students. The Sociological Review, 50(3), 398-418.

Redshaw, M., & Heikkilä, K. (2010). Delivered With Care. A National Survey of Women's Experience of Maternity Care 2010.

Reed, R., Sharman, R., & Inglis, C. (2017). Women's descriptions of childbirth trauma relating to care provider actions and interactions. BMC pregnancy and childbirth, 17(1), 21.

Reese, R. M. (2019). Designing towards empathy using VR/iVR: Applying best practices to enhance student experience. In Society for Information Technology & Teacher Education International Conference (pp. 434-440). Association for the Advancement of Computing in Education (AACE).

Reid, M. (2011). The impact of traumatic delivery on the mother–infant relationship. Infant Observation, 14(2), 117-128.

Reitmanova, S., & Gustafson, D. L. (2008). “They can’t understand it”: maternity health and care needs of immigrant Muslim women in St. John’s, Newfoundland. Maternal and Child Health Journal, 12(1), 101-111.

Renedo, A., & Marston, C. (2011). Healthcare professionals' representations of ‘patient and public involvement’and creation of ‘public participant’identities: Implications for the development of inclusive and bottom-up community participation initiatives. *Journal of Community & Applied Social Psychology*, *21*(3), 268-280.

Renfrew, M. J., McFadden, A., Bastos, M. H., Campbell, J., Channon, A. A., Cheung, N. F., ... & McCormick, F. (2014). Midwifery and quality care: findings from a new evidence-informed framework for maternal and newborn care. The Lancet, 384(9948), 1129-1145.

Ritchie, J., & Lewis, J. (2003). *Qualitative research practice: A guide for social science students and researchers*. London: Sage.

Ritchie, J., Lewis, J., Nicholls, C. M., & Ormston, R. (Eds.). (2013). Qualitative research practice: A guide for social science students and researchers. sage.

Ritchie, J., Spencer, L., Bryman, A., & Burgess, R. G. (1994). Analysing qualitative data.

Rizzo, A. S., & Shilling, R. (2017). Clinical virtual reality tools to advance the prevention, assessment, and treatment of PTSD. European journal of psychotraumatology, 8(sup5), 1414560.

Robb, Y., McInery, D., & Hollins Martin, C. J. (2013). Exploration of the experiences of young mothers seeking and accessing health services. Journal of Reproductive and Infant Psychology, 31(4), 399-412.

Roberts, A., Marshall, L., & Charlesworth, A. (2012). A Decade of Austerity?: The Funding Pressures Facing the NHS from 2010/11 to 2021/22. London: Nuffield Trust.

Roberts, M. and Ion, R. (2014) A critical consideration of systemic moral catastrophes in modern health care systems: an Arendtian perspective. Nurse Educ. Today, 34, pp.673-675.

Robertson, R., Wenzel, L., Thompson, J., & Charles, A. (2017). Understanding NHS financial pressures. How are they affecting patient care.

Robinson, J. (2001). Demand and supply in maternity care. British Journal of Midwifery, 9(8), 510-510.

Rosenberger, C. (2014). Beyond empathy: Developing critical consciousness through service learning. In Integrating service learning and multicultural education in colleges and universities (pp. 39-60). Routledge.

Ross-Davie M, Elliot S, Sakara A, Green L (2006). A Public Health Role In Perinatal Mental Health: Are Midwives Ready? British Journal of Midwifery: 14:6 330-334.

Rowan C, McCourt C, Bick D. (2010). Provision of Perinatal Mental Health Services in two English Strategic Health Authorities: Views and perspectives of the multi-professional team. Evidence Based Midwifery 8(3): 98-106

Royal College of Midwives. (2016). Why midwives leave–revisited.

Russell, J., Greenhalgh, T., Lewis, H., MacKenzie, I., Maskrey, N., Montgomery, J., & O'Donnell, C. (2013). Addressing the 'postcode lottery'in local resource allocation decisions: a framework for clinical commissioning groups. Journal of the Royal Society of Medicine, 106(4), 120-123.

Rutter, D., Francis, J. Coren, E. & Fisher, M. (2010). SCIE Systematic Research Review: guidelines (2nd Edn.) London: Social Care Institute for Excellence.

Sadler, G. R., Lee, H. C., Lim, R. S. H., & Fullerton, J. (2010). Recruitment of hard-to-reach population subgroups via adaptations of the snowball sampling strategy. Nursing & health sciences, 12(3), 369-374

Sampson, E. (1993). Celebrating the other: A dialogic account of human nature. Hemel Hempstead: Harvester Wheatsheaf.

Sandall, J., Benoit, C., Wrede, S., Murray, S. F., van Teijlingen, E. R., & Westfall, R. (2009). Social service professional or market expert? Maternity care relations under neoliberal healthcare reform. Current Sociology, 57(4), 529-553.

Sandelowski, M. (2004). Using qualitative research. Qualitative health research, 14(10), 1366-1386.

Sandlin, J. A., & Milam, J. L. (2008). "Mixing pop (culture) and politics": Cultural resistance, culture jamming, and anti-consumption activism as critical public pedagogy. Curriculum inquiry, 38(3), 323-350.

Saunders, B., Sim, J., Kingstone, T., Baker, S., Waterfield, J., Bartlam, B., ... & Jinks, C. (2018). Saturation in qualitative research: exploring its conceptualization and operationalization. *Quality & quantity*, *52*(4), 1893-1907.

Scamell, M., & Hanley, T. (2017). Innovation in preregistration midwifery education: Web based interactive storytelling learning. Midwifery, 50, 93-98.

Schetter, C. D., & Tanner, L. (2012). Anxiety, depression and stress in pregnancy: implications for mothers, children, research, and practice. Current opinion in psychiatry, 25(2), 141.

Schoeller, F., Zenasni, F., Bertrand, P., Gerry, L. J., Jain, A., & Horowitz, A. H. (2018). Combining virtual reality and biofeedback to foster empathic abilities in humans. Frontiers in psychology, 9, 2741.

Schutz, A. (1962). Concept and theory formation in the social sciences. In Collected Papers I (pp. 48-66). Springer, Dordrecht.

Schwandt, T. A. (2000). Three epistemological stances for qualitative inquiry: Interpretivism, hermeneutics, and social constructionism. Handbook of qualitative research, 2, 189-213.

Schwandt, T. A., Lincoln, Y. S., & Guba, E. G. (2007). Judging interpretations: But is it rigorous? Trustworthiness and authenticity in naturalistic evaluation. New directions for evaluation, 2007(114), 11-25.

Schwartz-Shea, P., & Yanow, D. (2013). Interpretive research design: Concepts and processes. Routledge.

Scott-Samuel, A., Bambra, C., Collins, C., Hunter, D. J., McCartney, G., & Smith, K. (2014). The impact of Thatcherism on health and well-being in Britain. International Journal of Health Services, 44(1), 53-71.

Seng, J., & Taylor, J. (2015). Trauma informed care in the perinatal period. Dunedin Academic Press Ltd.

Serdar K, Kelly NR, Palmberg AA, Lydecker JA, Thornton L, Tully CE, Mazzeo SE. (2014) Comparing online and face-to-face dissonance-based eating disorder prevention. Eat Disord. 2014;22(3):244–260. doi: 10.1080/10640266.2013.874824

Shafiei, T., Small, R., & McLachlan, H. (2012). Women's views and experiences of maternity care: a study of immigrant Afghan women in Melbourne, Australia. Midwifery, 28(2), 198-203.

Sharma M and Romas J (2012) Theoretical foundations of health education and health promotion. 2nd ed. Sundbury, MA: Jones & Barlett Learning LLC,

Shingleton RM, Richards LK, Thompson-Brenner H. Using technology within the treatment of eating disorders: a clinical practice review. Psychotherapy (Chic) 2013 Dec;50(4):576–582. doi: 10.1037/a0031815.

Shor, I. (1992). Empowering Education: Critical Teaching for Social Change. Chicago and London: University of Chicago Press.

Shor, I., & Freire, P. (1987). What Is the “Dialogical Method” of Teaching? Journal of Education, 169, 11-31

Simkin P. (1991) Just another day in a woman’s life? Part I Women’s long-term perception of their first birth experience. Birth. 1991;18:203–10.

Simpson, M., & Catling, C. (2016). Understanding psychological traumatic birth experiences: A literature review. Women and Birth, 29(3), 203-207.

Siu, M. Y. (2018). Empathy in Nursing: Understanding Patient’s Experience through Digital Storytelling. SF Nurs Health J, 2(2), 2.

Smith, A. H. K., Dixon, A. L., & Page, L. A. (2009). Health-care professionals’ views about safety in maternity services: a qualitative study. Midwifery, 25(1), 21-31.

Soet, J., Brack, G., & Dilorio, C. (2003). Prevalence and predictors of women’s experience of psychological trauma during childbirth. Birth, 30, 36–46

Solnes Miltenburg, A., van Pelt, S., Meguid, T., & Sundby, J. (2018). Disrespect and abuse in maternity care: individual consequences of structural violence. Reproductive health matters, 26(53), 88-106.

Solorano D (2000) Teaching and social change: Reflections on a Freirean approach in a college classroom. In: Shor I and Pari C (eds) Education is politics. Critical teaching across differences, postsecondary. Portsmouth, NH: Boynton/Cook Publishers, Inc., 2000, pp.15–27

Sparkes A. (1992) The paradigms debate: An extended review and a celebration of difference. In: Sparkes A (ed.) Research in physical education and sport: Exploring alternative visions. Bristol, PA: The Falmer Press, 1992.

Speed, E., & Mannion, R. (2017). The rise of post-truth populism in pluralist liberal democracies: challenges for health policy. International journal of health policy and management, 6(5), 249.

Spencer, L., Ritchie, J., Lewis, J., & Dillon, L. (2003). Quality in qualitative evaluation: a framework for assessing research evidence.

Spidsberg, B. D. (2007). Vulnerable and strong–lesbian women encountering maternity care. Journal of Advanced Nursing, 60(5), 478-486.

Statista (2019) https://www.statista.com/statistics/274774/forecast-of-mobile-phone-users-worldwide/ Last accessed 26/5/2019

Steenkamp, M. M., Boasso, A. M., Nash, W. P., Larson, J. L., Lubin, R. E., & Litz, B. T. (2015). PTSD symptom presentation across the deployment cycle. Journal of affective disorders, 176, 87-94.

Stewart, C. P., Oaks, B. M., Laugero, K. D., Ashorn, U., Harjunmaa, U., Kumwenda, C., ... & Dewey, K. G. (2015). Maternal cortisol and stress are associated with birth outcomes, but are not affected by lipid-based nutrient supplements during pregnancy: an analysis of data from a randomized controlled trial in rural Malawi. BMC pregnancy and childbirth, 15(1), 346.

Sturgeon, D. (2014). The business of the NHS: The rise and rise of consumer culture and commodification in the provision of healthcare services. Critical social policy, 34(3), 405-416.

Sturgeon, D. (2014). The business of the NHS: The rise and rise of consumer culture and commodification in the provision of healthcare services. Critical social policy, 34(3), 405-416.

Su, Q., Zhang, H., Zhang, Y., Zhang, H., Ding, D., Zeng, J., ... & Li, H. (2015). Maternal stress in gestation: birth outcomes and stress-related hormone response of the neonates. Pediatrics & Neonatology, 56(6), 376-381.

Swain, D. V., & Swain, J. R. (1988). Film scriptwriting: a practical manual. Focal Press.

Tatarchevskiy, T. (2011). The 'popular'culture of internet activism. New Media & Society, 13(2), 297-313.

Taylor, E. W. (2017). Transformative learning theory. In Transformative Learning Meets Bildung (pp. 17-29). SensePublishers, Rotterdam.

Teal, C. R., & Street, R. L. (2009). Critical elements of culturally competent communication in the medical encounter: a review and model. Social science & medicine, 68(3), 533-543.

The Birth Trauma Association (2015) http://www.birthtraumaassociation.org.uk/publications/Post_Natal_PTSD.pdf

The Cochrane Collaboration (2011) http://handbook.cochrane.org/chapter_21/21_5_ethics_and_inequalities.htm last Accessed 28/03/2017

The Department of health (2012a) No health without mental health: implementation framework. The Department of Health. London. https://www.gov.uk/government/uploads/system/uploads/attachment_data/file/216870/No-Health-Without-Mental-Health-Implementation-Framework-Report-accessible-version.pdf. Last Accessed 28/03/2017

The Department of health (2012b) Recommendations to improve health of children and young people. The Department of Health. London. https://www.gov.uk/government/publications/independent-experts-set-out-recommendations-to-improve-children-and-young-people-s-health-results Last accessed 22/02/2016 Last Accessed 28/03/2017

The Department of health (2012b) Recommendations to improve health of children and young people. The Department of Health. London. https://www.gov.uk/government/publications/independent-experts-set-out-recommendations-to-improve-children-and-young-people-s-health-results Last accessed 22/02/2016 Last Accessed 28/03/2017

The Department of Health (2014*). Health inequalities: assessment of the fulfilment of health inequalities duties in 2014 to 2015* The Department of Health. London.

The National Institute of Health Research (2017) http://www.hiat.org.uk/introduction.html) Last Accessed 28/03/2017
The Royal College of Midwives (2016) Going Digital: Developing resources for women and families in maternity care. A better births summary. The Royal College of Midwives Pg 41-45

The World Health Organization (2017) (http://www.who.int/hia/about/glos/en/) Last Accessed 28/03/2017

Thesen, J. (2005). From oppression towards empowerment in clinical practice—offering doctors a model for reflection. Scandinavian Journal of Public Health, 33(66_suppl), 47-52.

Thomas J. & Harden A. (2008) Methods for the thematic synthesis of qualitative research in systematic reviews. BMC Medical Research Methodology. 8(45), 1–19. doi:10.1186/1471-2288-8- 45.

Thompson, N. (2015). Seeing power: Art and activism in the 21st century. Melville House.

Thomson G, Downe S. Widening the trauma discourse: the link between childbirth and experiences of abuse. J Psychosom Obstet Gynecol. 2008; 29(4):268–73

Thomson, G. M., & Downe, S. (2010). Changing the future to change the past: women's experiences of a positive birth following a traumatic birth experience. Journal of reproductive and infant psychology, 28(1), 102-112.

Thomson, G., & Downe, S. (2016). Emotions and support needs following a distressing birth: Scoping study with pregnant multigravida women in North-West England. Midwifery, 40, 32-39.

Thomson, G., & Schmied, V. (Eds.). (2017). Psychosocial Resilience and Risk in the Perinatal Period: Implications and Guidance for Professionals. Taylor & Francis.

Thomson, G., Beck, C., & Ayers, S. (2017). The ripple effects of a traumatic birth. Psychosocial Resilience and Risk in the Perinatal Period: Implications and Guidance for Professionals, 154.

Thomson, G., Dykes, F., Singh, G., Cawley, L., & Dey, P. (2013). A public health perspective of women's experiences of antenatal care: An exploration of insights from a community consultation. Midwifery, 29(3), 211-216.

Timmi, S. B. (1996). Race and colour in internal and external reality. British Journal of Psychotherapy, 13(2), 183–192.

Tong A, Flemming K, McInnes E, Oliver S, Craig J: Enhancing transparency in reporting the synthesis of qualitative research: ENTREQ. BMC Med Res Methodol. 2012, 12 (1): 181-10.1186/1471-2288-12-181.

Topol, E. (2012). The creative destruction of medicine: How the digital revolution will create better health care. Basic Books.

Tripp, N., Hainey, K., Liu, A., Poulton, A., Peek, M., Kim, J., & Nanan, R. (2014). An emerging model of maternity care: smartphone, midwife, doctor?. Women and Birth, 27(1), 64-67.

Tuck, S. G., Summers, A. C., Bowie, J., Fife-Stallworth, D., Alston, C., Hayes, S., & Alexander, S. (2017). B'More Fit for Healthy Babies: using trauma-informed care policies to improve maternal health in Baltimore city. Women's health issues, 27, S38-S45.

Turton, P., P. Hughes, et al. (2001). "Incidence, correlates and predictors of posttraumatic stress disorder in the pregnancy after stillbirth." British Journal of Psychiatry 178: 556-560.

Tyler, D. I. (2013). Revolting subjects: Social abjection and resistance in neoliberal Britain. Zed Books Ltd.

Tyler, I. (2008). "Chav mum chav scum" Class disgust in contemporary Britain. Feminist media studies, 8(1), 17-34.

Van Apeldoorn, B., & Overbeek, H. (2012). Introduction: The life course of the neoliberal project and the global crisis. In H. Overbeek & B. van Apeldoorn (Editors.), Neoliberalism in crisis (pp. 1–20). Basingstoke, UK: Palgrave Macmillan.

Van der Zande, I. S., van der Graaf, R., Oudijk, M. A., & Van Delden, J. J. (2017). Vulnerability of pregnant women in clinical research. *Journal of medical ethics*, *43*(10), 657-663.

Van Manen, M. (2016). Researching lived experience: Human science for an action sensitive pedagogy. Routledge.

Van Son, M., Verkerk, G., van der Hart, O., Komproe, I., & Pop, V. (2005). Prenatal depression, mode of delivery and perinatal dissociation as predictors of postpartum posttraumatic stress: an empirical study. Clinical Psychology & Psychotherapy: An International Journal of Theory & Practice, 12(4), 297-312.

Vasileiou, K., Barnett, J., Thorpe, S., & Young, T. (2018). Characterising and justifying sample size sufficiency in interview-based studies: systematic analysis of qualitative health research over a 15-year period. BMC medical research methodology, 18(1), 148. doi:10.1186/s12874-018-0594-7

Venugopal, R. (2015). Neoliberalism as concept. Economy and Society, 44(2), 165-187.

Vetter, N. (2003). Values in the NHS.

Vincent, C., & Amalberti, R. (2016). Seeing Safety Through the Patient's Eyes. In Safer Healthcare (pp. 39-46). Springer, Cham.

Visser, L. M., Benschop, Y. W., Bleijenbergh, I. L., & van Riel, A. C. (2018). Unequal Consumers: Consumerist healthcare technologies and their creation of new inequalities. Organization Studies, 0170840618772599.

Vossbeck-Elsebusch, A. N., Freisfeld, C., and Ehring, T. (2014). Predictors of posttraumatic stress symptoms following childbirth. BMC Psychiatry 14:200. doi: 10.1186/1471-244X-14-200

Wadmann, S., & Hoeyer, K. (2018). Dangers of the digital fit: Rethinking seamlessness and social sustainability in data-intensive healthcare. Big Data & Society, 5(1), 2053951717752964.

Waheed, W., Hughes-Morley, A., Woodham, A., Allen, G., & Bower, P. (2015). Overcoming barriers to recruiting ethnic minorities to mental health research: a typology of recruitment strategies. BMC psychiatry, 15(1), 101.

Wald, H. S., McFarland, J., & Markovina, I. (2018). Medical humanities in medical education and practice. Medical teacher, 1-5.

Wallerstein N and Bernstein E. (1988) Empowerment education: Freire’s ideas adapted to health education. Health Educ Behav 1988: 15: 379–394.

Wallerstein N. (1992) Powerlessness, empowerment, and health: Implications for health promotion programs. Am J Health Promot 1992; 6: 197–205

Walsh, D. J. (2010). Childbirth embodiment: problematic aspects of current understandings. Sociology of health & illness, 32(3), 486-501

Walsh, D., & Downe, S. (2005). Meta-synthesis method for qualitative research: a literature review. Journal of advanced nursing, 50(2), 204-211.

Walsh, D., & Downe, S. (2006). Appraising the quality of qualitative research. Midwifery, 22(2), 108-119.

Ward, D. J., Furber, C., Tierney, S., & Swallow, V. (2013). Using Framework Analysis in nursing research: a worked example. Journal of advanced nursing, 69(11), 2423-2431.

Ward, T. C. S., Mazul, M., Ngui, E. M., Bridgewater, F. D., & Harley, A. E. (2013). “You learn to go last”: Perceptions of prenatal care experiences among African-American women with limited incomes. Maternal and child health journal, 17(10), 1753-1759.

Watson, H., Harrop, D., Walton, E., Young, A., & Soltani, H. (2019). A systematic review of ethnic minority women’s experiences of perinatal mental health conditions and services in Europe. PloS one, 14(1), e0210587.

Watson, V. S. (2016). Re-Traumatization of Sexual Trauma in Women's Reproductive Health Care.

Weber, L., & Parra-Medina, D. (2003). Intersectionality and women’s health: Charting a path to eliminating health disparities. In Gender Perspectives on Health and Medicine (pp. 181-230). Emerald Group Publishing Limited.

Weiss PL, Sveistrup H, Rand D, Kizony R (2009) Video capture virtual reality: a decade of rehabilitation assessment and intervention. Phys Ther Rev; 14:307–321.

Wenzel, L., & Jabbal, J. (2016). User feedback in maternity services

West, M. (2016). Creating a Workplace Where NHS Staff Can Flourish. The King's Fund, London.

White, T., Matthey, S., Boyd, K., & Barnett, B. (2006). Postnatal depression and post-traumatic stress after childbirth: Prevalence, course and co-occurrence. Journal of Reproductive and Infant Psychology, 24(02), 107-120.

Whitehead, C. Selleger, V. Van de Kreeke, J. Hodges, B. (2014) The ‘missing person’ in roles-based competency models. ; A historical. Cross -national , contrastive case study. Med Education. 48. 785-95.

Wiederhold B,K (2016) VRMC: The Virtual Reality Medical Center [website] The potential for virtual reality to improve health care. 2006. [Accessed May 3, 2016]. http://www.iactor.eu/downloads/WP%20The%20Potential%20for%20VR%20to%20Improve%20Healthcare.pdf

Wiederhold M,D, Wiederhold B,K (2007) Virtual reality and interactive simulation for pain distraction. Pain Med. 2007;8:182–188.

Wijma, E. M., Veerbeek, M. A., Prins, M., Pot, A. M., & Willemse, B. M. (2018). A virtual reality intervention to improve the understanding and empathy for people with dementia in informal caregivers: results of a pilot study. Aging & mental health, 22(9), 1121-1129.

Wilkes, M., & Bligh, J. (1999). Evaluating educational interventions. Bmj, 318(7193), 1269-1272.

Williams, B. A. O. (2002). Truth & truthfulness: An essay in genealogy. Princeton University Press.

Wilmore, M., Rodger, D., Humphreys, S., Clifton, V. L., Dalton, J., Flabouris, M., & Skuse, A. (2015). How midwives tailor health information used in antenatal care. Midwifery, 31(1), 74-79.

Wilson, H. J. (2000) 'The myth of objectivity: Is medicine moving towards a social constructivist medical paradigm?' Family Practice. Vol 17, No 2, pp. 203-209.

Wilton, T., & Kaufmann, T. (2001). Lesbian mothers' experiences of maternity care in the UK. Midwifery, 17(3), 203-211.

Wolff, M., Wagner, M. J., Poznanski, S., Schiller, J., & Santen, S. (2015). Not another boring lecture: engaging learners with active learning techniques. The Journal of emergency medicine, 48(1), 85-93.

Womens Health and Quality Consortium (WHEC) (2013) Briefing: Enablers and barriers to wellbeing – experiences of BME women in Manchester, available at. http://www.whec.org.uk/wordpress/wp-content/uploads/downloads/2013/03/WHEC-briefing-local-roadshow-events-March-2013.pdf Last accessed 15/4/2016

World Health Organization. (2013). Global and regional estimates of violence against women. Prevalence and health effects of intimate partner violence and non-partner sexual violence. In *Global and regional estimates of violence against women. Prevalence and health effects of intimate partner violence and non-partner sexual violence*.

World Health Organisation WHO (2016) https://www.who.int/news-room/fact-sheets/detail/child-maltreatment Last accessed 20/06/2017

World Health Organization. (2018). WHO recommendations: intrapartum care for a positive childbirth experience.

Wright, E. M., Matthai, M. T., & Budhathoki, C. (2018). Midwifery Professional Stress and Its Sources: A Mixed-Methods Study. Journal of midwifery & women's health, 63(6), 660-667.

Yaldren, J., & Van Loo, M. (2018). Technology and inclusivity. British Journal of Cardiac Nursing, 13(2), 86-87.

Yildiz, P. D., Ayers, S., and Phillips, L. (2017). The prevalence of posttraumatic stress disorder in pregnancy and after birth: a systematic review and meta-analysis. J. Affect. Disord. 208, 634–645. doi: 10.1016/j.jad.2016.10.009

Yildiz, P., Ayers, S., & Phillips, L. (2018). Longitudinal trajectories of post-traumatic stress disorder (PTSD) after birth and associated risk factors. Journal of affective disorders, 229, 377-385

Young, R. A., & Collin, A. (2004). Introduction: Constructivism and social constructionism in the career field. Journal of vocational behavior, 64(3), 373-388.

Zambaldi, C. F., Cantilino, A., and Sougey, E. B. (2011). Bio-socio-demographic factors associated with post-traumatic stress disorder in a sample of postpartum Brazilian women. Arch. Womens Ment. Health 14, 435–439. doi: 10.1007/s00737 011-022
