# Supplementary material for: The design, delivery and evaluation of ‘Human Perspectives VR’: An immersive educational programme designed to raise awareness of contributory factors for a traumatic childbirth experience and PTSD
Source: PLoS One. 2022 Nov 2;17(11):e0276263. doi: 10.1371/journal.pone.0276263 (PMC9629609; doi:10.1371/journal.pone.0276263)
Supplement: S4 File — (DOCX) [file pone.0276263.s004.docx]

**Pre intervention questionnaire**

1. Please can you list any intrapartum factors that could contribute to a woman experiencing her birth as traumatic?

_______________________________________________________________________________

_______________________________________________________________________________

_______________________________________________________________________________

_______________________________________________________________________________

1. Please can you describe what Post-traumatic stress disorder (PTSD) is?

_______________________________________________________________________________

_______________________________________________________________________________

_______________________________________________________________________________

_______________________________________________________________________________

1. Please can you detail any specific risk factors that may make it more likely that a woman experiences birth trauma/PTSD following childbirth?

_______________________________________________________________________________

_______________________________________________________________________________

_______________________________________________________________________________

_______________________________________________________________________________

1. Please can you detail any/types of training you have received in relation to birth trauma/PTSD following childbirth.

_______________________________________________________________________________

_______________________________________________________________________________

_______________________________________________________________________________

_______________________________________________________________________________

1. Have you witnessed care practices that you feel may have contributed to a woman experiencing her birth as traumatic. Yes / No

If yes, please could you provide further details below:

_______________________________________________________________________________

_______________________________________________________________________________

_______________________________________________________________________________

_______________________________________________________________________________

1. Do you feel you have a good understanding of how birth trauma/PTSD onset following childbirth is caused (please circle the most appropriate response)

| **Strongly agree** | **Agree** | **Neither agree nor disagree** | **Disagree** | **Strongly disagree** |
| --- | --- | --- | --- | --- |

1. Do you feel able to recognise women at risk of birth trauma/PTSD onset (please circle the most appropriate response)?

| **Strongly agree** | **Agree** | **Neither agree nor disagree** | **Disagree** | **Strongly disagree** |
| --- | --- | --- | --- | --- |

1. Do you feel able to impact positively upon a woman’s birth experience?

| **Strongly agree** | **Agree** | **Neither agree nor disagree** | **Disagree** | **Strongly disagree** |
| --- | --- | --- | --- | --- |

1. Do you feel you have received sufficient training in relation to birth trauma/PTSD onset following childbirth (please circle the most appropriate response)?

| **Strongly agree** | **Agree** | **Neither agree nor disagree** | **Disagree** | **Strongly disagree** |
| --- | --- | --- | --- | --- |

If you have any further comments/issues in relation to identifying birth trauma/PTSD following childbirth and the level/types of training received in this area, please detail here:

_______________________________________________________________________________

_______________________________________________________________________________

_______________________________________________________________________________

_______________________________________________________________________________

**THANK YOU FOR YOUR PARTICIPATION. PLEASE RETURN THE COMPLETED QUESTIONNAIRE TO THE FACILITATOR OF THE SESSION BEFORE LEAVING.**

**Post intervention questionnaire**

1. Please can you list any intrapartum factors that could contribute to a woman experiencing her birth as traumatic?

_______________________________________________________________________________

_______________________________________________________________________________

_______________________________________________________________________________

_______________________________________________________________________________

1. Please can you describe what Post-traumatic stress disorder (PTSD) is?

_______________________________________________________________________________

_______________________________________________________________________________

_______________________________________________________________________________

_______________________________________________________________________________

1. Please can you detail any specific risk factors that may make it more likely that a woman experiences birth trauma/PTSD following childbirth?

_______________________________________________________________________________

_______________________________________________________________________________

_______________________________________________________________________________

_______________________________________________________________________________

1. Following the training how far do you feel able to understand how birth trauma / PTSD following childbirth can be caused (please circle the most appropriate response)?

| **Much more able** | **More able** | **Neither more or less able** | **Less able** | **Much more less able** |
| --- | --- | --- | --- | --- |

1. Following the training I feel I am more able to recognise women at risk of birth trauma/PTSD onset following childbirth (please circle the most appropriate response)?

| **Strongly agree** | **Agree** | **Neither agree nor disagree** | **Disagree** | **Strongly disagree** |
| --- | --- | --- | --- | --- |

1. Following the training I feel I am more able to make a positive impact on a woman’s birth experience?

| **Strongly agree** | **Agree** | **Neither agree nor disagree** | **Disagree** | **Strongly disagree** |
| --- | --- | --- | --- | --- |

1. Please could you indicate how useful the training programme is (by circling the most appropriate response) in terms of:
2. Raising awareness of birth trauma/PTSD following childbirth?

| **Very useful** | **Useful** | **Not very useful** | **Not at all useful** |
| --- | --- | --- | --- |

1. Raising awareness as to how to improve women’s birth experiences?

| **Very useful** | **Useful** | **Not very useful** | **Not at all useful** |
| --- | --- | --- | --- |

1. What did you find most useful about the training programme?

_______________________________________________________________________________

_______________________________________________________________________________

_______________________________________________________________________________

_______________________________________________________________________________

1. What did you find least useful about the training programme?

_______________________________________________________________________________

_______________________________________________________________________________

_______________________________________________________________________________

1. Do you have any thoughts on the use of VR?

_______________________________________________________________________________

_______________________________________________________________________________

_______________________________________________________________________________

1. If you have any suggestions on how the training could be improved or any other training required in this area, please provide suggestions below.

_______________________________________________________________________________

_______________________________________________________________________________

_______________________________________________________________________________

_______________________________________________________________________________

**THANK YOU FOR YOUR PARTICIPATION**
