# Supplementary material for: The design, delivery and evaluation of ‘Human Perspectives VR’: An immersive educational programme designed to raise awareness of contributory factors for a traumatic childbirth experience and PTSD
Source: PLoS One. 2022 Nov 2;17(11):e0276263. doi: 10.1371/journal.pone.0276263 (PMC9629609; doi:10.1371/journal.pone.0276263)
Supplement: Supplementary file 4 [file pone.0276263.s003.docx]

EMMA'S STORY

INT HOSPITAL BIRTHING ROOM DAY

A women (EMMA) is lying on a hospital bed in a patient gown, from Emma's POV (point of view) we see a bright clinical light shining in her eyes, the midwife moves it and we see the birthing room.

MIDWIFE
(Removing gloves) You're still 5cm. Same as the last time, you've got a stubborn little one in there haven't you? I'll have to inform the doctor, I'll be back in a min.

EMMA
What does that mean?

MIDWIFE
We like there to be some changes at each vaginal examination, it tells us things are moving along, if they aren't we need to find out why. There are things we can do to help so don't worry. I'll be back in a min with the doctor.

The midwife leaves the room, camera pans the birthing room from Emma's POV.

The midwife returns with the DOCTOR. They do not make eye contact with Emma and head for the end of the bed and stand reading her notes, next to the computer.

MIDWIFE (CONT'D)
(To doctor) There's been no progress sine the last VE, she's still 5cm, no decent of the fetal head. Not really coping with gas and air but refused an epidural and diamorphine because of a fear of needles. Her temperature was 37.8 On her last observations.

DOCTOR
The midwife has told me you haven't progressed in 4 hours since your last VE. This concerns me, your waters broke over 48 hours ago putting you at an increased risk of infection.
(MORE)

DOCTOR (CONT'D)
I know you wanted a natural birth without intervention but I feel we need to intervene and put you on a drip to speed things along. It's just a little needle in your hand attached to a drip, the midwife will be in control of the rate. Ideally we want to strengthen those contractions and hopefully see some changes at the next VE. Ill be back in 4 hours to see how you're getting on.

The Doctor leaves the room and the midwife starts to prepare the cannula (taking needles out of packaging) whilst addressing Emma.

MIDWIFE
I know you don't like needles but it's really important we get this needle in to give you the medication to help things progress. It will be fine, I'm really good at these, shouldn't take a minute or two, it'll be over before you know it.

The midwife has the cannula on a tray, ready.

EMMA
I feel like I want to push.

MIDWIFE
You're feeling pressure? It's the baby pressing against your back passage, you're baby is back to back so you feel it a lot more, just keep going on that gas and air. Deep breaths, through the pain, that's it, that's it, well done see. Use that gas and air whilst I put this cannula in your hand, take your mind off it.

The midwife puts the tourniquet on Emma's arm ready for the cannula, Emma's breathing gets faster and she tries to grab the midwifes arm/hand for reassurance, the midwife continues what she's doing.

MIDWIFE (CONT'D)
Emma love, you're going to just have o hold onto the side whilst I'm doing this, keep still as best you can.

We hear the sound of the baby's heart beat slowing down, the midwife stops what she is doing and adjusts the monitor on Emma's stomach whilst looking at the CTG machine.

EMMA
Is something wrong?

MIDWIFE
No, everything's fine.

The midwife leaves the room and returns with the doctor. They discuss the trace(on entering the room, viewer can't hear them) They stand over Emma's bed (either side).

DOCTOR
Hello, me again, this trace shows your baby is being a little naughty. (To midwife) that's a couple now, atypical, prolonged and variability is not great, how have movements been?

MIDWIFE
She's had plenty of movements, I was just trying to get the cannula in for the drip.

DOCTOR
Hold off the drip for now, I'd like to see variability improve, maybe some fluids. I may have to do a VE to confirm position and see how favorable her cervix is. Did you say she was 5cm at the last VE?

MIDWIFE
Yes,5, head high, anterior cervix felt oedematous.

The doctor goes to the cupboard to get gloves.

DOCTOR
Stirrups please.

MIDWIFE
The doctor is just going to do another vaginal examination to check baby's position and see what's happening with your cervix as baby is getting a little tired now. You're been in labour such a long time, I bet you're shattered!
(MORE)

MIDWIFE (CONT'D)
Ok Emma, we are just going to put your legs in these stirrups for the examination, it makes it a lot easier for the doctor to feel the baby's head, will only be a minute.

(Uncomfortable position for Emma) The doctor is at the end of the bed.

DOCTOR
Ok, ready, just relax.

The doctor does his examination, the midwife is stood at the monitor documenting what is happening.

MIDWIFE
(Looking at the monitor) Just keep breathing on that gas.

DOCTOR
Still 5 and the head is really high, I think we need to look at our next move, baby is getting a little tired. I'll speak to me consultant about a plan.

The doctor washes his hands at the sink, the midwife continues to document the CTG monitor, the doctor returns.

DOCTOR (CONT'D)
I've had a word with the consultant and due to your lengthy labour and lack of progress and your waters have been gone for over 48 hours, the consultant has decided that a caesarean section would be the best option. You gave it a go but these things happen. I'll prepare for theatre and see you in there. Ok? We shouldn't be long getting you in.

Doctor leaves the room.

The midwife begins to prepare Emma for theatre, she removes her legs from the stirrups and starts to ask her questions from the theatre checklist (we will have a list of questions on shoot)

MIDWIFE

If you would like someone to be with you then you're probably best calling them now as I imagine we will be in there in the next half hour.

The midwife will read the pre op questions to Emma (the questions will be provided on the day of the shoot)

MIDWIFE (CONT'D)

I'm just going to get changed for theatre, ill be back in a min.

We will see the empty room from Emma's POV for 20 seconds then fade out. THE END
